# Transcriptome analysis reveals microvascular endothelial cell-dependent pericyte differentiation

**DOI:** 10.1038/s41598-019-51838-x

**Published:** 2019-10-30

**Authors:** Maarten M. Brandt, Christian G. M. van Dijk, Ranganath Maringanti, Ihsan Chrifi, Rafael Kramann, Marianne C. Verhaar, Dirk J. Duncker, Michal Mokry, Caroline Cheng

**Affiliations:** 1 Experimental Cardiology, Department of Cardiology, Thoraxcenter, Erasmus MC, University Medical Center Rotterdam, Rotterdam, The Netherlands; 2 Department of Nephrology and Hypertension, Division of Internal Medicine and Dermatology, University Medical Center Utrecht, Utrecht, The Netherlands; 3 Division of Nephrology and Clinical Immunology, RWTH Aachen University Medical Faculty, RWTH Aachen University, Aachen, Germany; 4 Department of Internal Medicine, Nephrology and Transplantation, Erasmus University Medical Center, Rotterdam, The Netherlands; 5 Epigenomics facility, University Medical Center Utrecht, Utrecht, The Netherlands; 6 Regenerative Medicine Center Utrecht, University Medical Center Utrecht, Utrecht, The Netherlands

**Keywords:** Cell biology, Molecular biology

## Abstract

Microvascular homeostasis is strictly regulated, requiring close interaction between endothelial cells and pericytes. Here, we aimed to improve our understanding of how microvascular crosstalk affects pericytes. Human-derived pericytes, cultured in absence, or presence of human endothelial cells, were studied by RNA sequencing. Compared with mono-cultured pericytes, a total of 6704 genes were differentially expressed in co-cultured pericytes. Direct endothelial contact induced transcriptome profiles associated with pericyte maturation, suppression of extracellular matrix production, proliferation, and morphological adaptation. *In vitro* studies confirmed enhanced pericyte proliferation mediated by endothelial-derived PDGFB and pericyte-derived HB-EGF and FGF2. Endothelial-induced PLXNA2 and ACTR3 upregulation also triggered pericyte morphological adaptation. Pathway analysis predicted a key role for TGFβ signaling in endothelial-induced pericyte differentiation, whereas the effect of signaling via gap- and adherens junctions was limited. We demonstrate that endothelial cells have a major impact on the transcriptional profile of pericytes, regulating endothelial-induced maturation, proliferation, and suppression of ECM production.

## Introduction

Complex organisms such as vertebrates rely on a well-functioning circulatory system to meet the body’s oxygen and nutrient demand, and to remove waste products. The circulatory system is composed of blood vessels, lined by a single layer of endothelial cells (ECs) on the luminal side. These ECs are surrounded by a basement membrane which they share with mural cells. In the microvasculature, these mural cells consist of pericytes^1^.

Maintaining microvascular homeostasis is a strictly regulated process, which requires close interplay between ECs and pericytes. Dysregulation of this comprehensive interaction is associated with the onset and progression of a variety of diseases^2^. Lack of pericytes compromises vascular integrity and causes leaky unstable vessels (e.g. in rapidly growing tumors)^3^, as well as highly proliferative endothelium (e.g. in diabetic retinopathy)^4^. Moreover, pericytes have previously been linked to pathological organ fibrosis^5^, though whether injury-induced stimulation, or loss of endothelial interaction drives this differentiation is poorly understood. Former studies on microvascular cross-talk provided valuable insights into the mechanisms involved in regulating vascular homeostasis. For instance, Platelet Derived Growth Factor Subunit B (PDGFB) secretion by ECs was shown to modulate pericyte proliferation and migration towards the endothelium^4^, whereas pericyte-derived Vascular Endothelial Growth Factor A (VEGFA) and Angiopoietin 1 secretion were reported to promote endothelial survival and maturation^6,7^. In addition to these paracrine interactions, ECs and pericytes also connect physically. At distinct places, the basement membrane separating the two cell types is interrupted, allowing the formation of direct connection sites called peg and socket contacts^8^. These contacts are highly enriched in gap- and adherens junctions, which provide a direct signaling route for ions, nutrients, metabolites, and secondary messengers^9^. Over the years, numerous studies have focused on the different aspects of signaling between these closely associated microvascular cells. However, since most emphasis was put on how pericytes affect endothelial behavior, only little is known about the consequence of this cross-talk for pericytes.

To gain a deeper understanding of the impact of vascular crosstalk on these critical, yet relatively underexposed, contributors of microvascular homeostasis, an RNA sequence- (RNAseq) based analysis was performed to compare the mRNA expression profiles of single cultured pericytes, with those of pericytes cultured in direct contact with endothelial cells. The results demonstrate that ECs have a major impact on the transcriptional profile of pericytes and provide functional evidence for endothelium-induced pericyte maturation, proliferation, and suppression of ECM expression.

## Results

### Endothelial cells markedly affect pericyte phenotype

**Figure 1:**
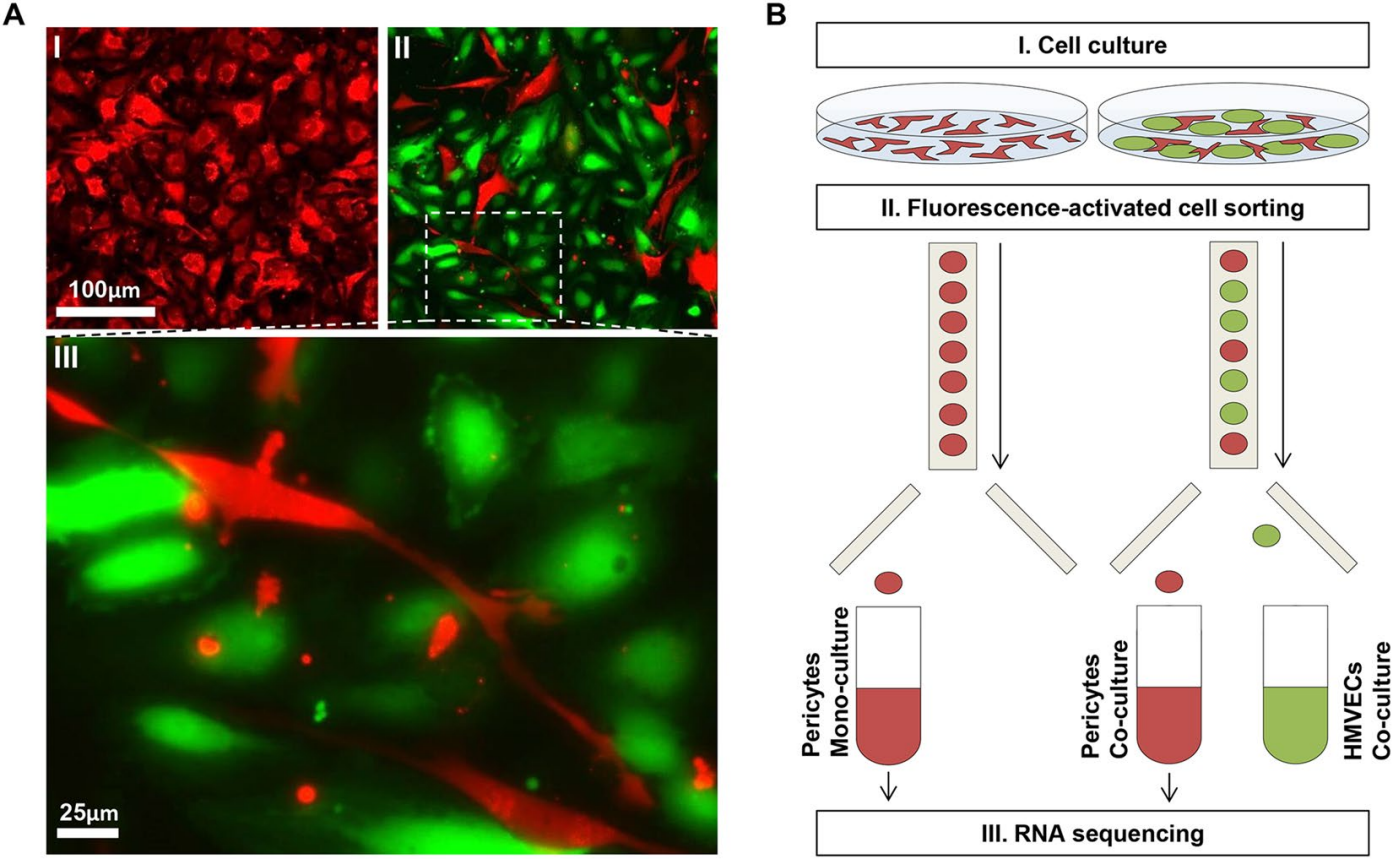
Expression profiles were generated from mono- and co-cultured pericytes via RNA sequencing. (**A**) Pericytes labeled with dsRED (red) were either cultured alone (I), or in direct contact with GFP-labeled HMVECs (green) (II). Magnified view of co-cultured cells clearly shows the elongated pericytes that appear to be in contact with multiple ECs (III). **(B)** Schematic overview of the experiments: Pericytes labeled with dsRED were cultured in a confluent layer, either alone, or in direct contact with GFP-labeled HMVECs for the duration of 20 hours (I). Hereafter, cells were trypsinized and sorted based on fluorescent signal (II), after which RNA was isolated from the pericytes for RNA sequencing (III).

**Figure 2:**
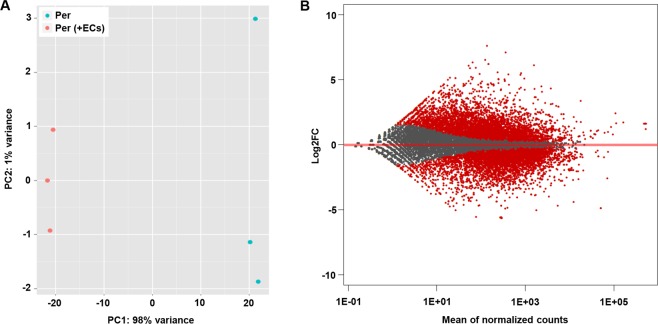
Endothelial cells have a major impact on the expression profile of pericytes. (**A**) PCA plot of expression profiles in single cultured pericytes (blue) and co-cultured pericytes (red), derived from 3 experimental replicates composed of single cultured and co-cultured pericytes. The x- and y-axes reflect the variance between the samples analyzed by RNAseq, in which most of the variance (98%) came from pericytes being cultured in absence or in presence of endothelial cells (x-axis), and where likely the experimental variation had only a limited contribution (y-axis). (**B**) Graphic display of differential gene expression (MA plot) in which log2FC is plotted against the mean of normalized counts. Red dots represent the 6704 differentially expressed genes (P adjusted < 0.05), gray dots represent non-differentially expressed genes.

To evaluate the impact of endothelial-pericyte interaction on pericyte behavior, discosoma sp. red- (dsRED) labeled pericytes were cultured in a confluent layer either alone, or in the presence of green fluorescent protein- (GFP) labeled human dermal microvascular endothelial cells (HMVECs), enabling direct contact between the two different cell types (Fig. 1A). Twenty hours post seeding, cells were trypsinized and sorted based on fluorescent signal, after which RNA was isolated and processed for RNA sequencing (Fig. 1B). A comparison of the transcription profile of single cultured pericytes and co-cultured pericytes in a principal component analysis (PCA) clearly illustrated the major effect of endothelial-pericyte crosstalk on pericytes (Fig. 2A). In total, 6704 genes were differentially expressed (P adjusted <0.05; Fig. 2B, Supplemental Table 3). Of these 6704 differentially expressed genes, 6081 were protein coding genes (almost one third of the estimated 19000 protein coding genes in the human genome)^10^, suggesting that direct contact with ECs dramatically affects pericyte’s transcriptomes.

### Direct contact with endothelium stimulates pericyte maturation

**Figure 3:**
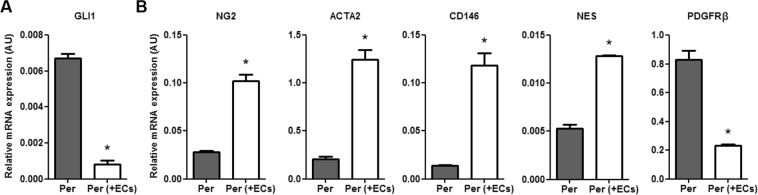
Direct contact with endothelium stimulates pericyte maturation. (**A**) QPCR results showing expression levels of mesenchymal transcription factor GLI1, and **(B)** pericyte markers NG2, ACTA2, CD146, NES, and PDGFRβ relative to RPLP0 and POLR2L in pericytes in monoculture (Per), and after 20 h in co-culture with HMVECs (Per + ECs). N = 3, *P < 0.05 compared to pericytes in monoculture. GLI1: Glioma-associated oncogene 1, NG2: Chondroitin Sulfate Proteoglycan 4, ACTA2: Alpha Smooth Muscle Actin 2, CD146: Melanoma Cell Adhesion Molecule, NES: Nestin, PDGFRβ: Platelet Derived Growth Factor Receptor Beta.

A newly formed endothelial network is initially unstable and requires support from recruited pericytes. These pericytes either migrate from adjacent vessels or differentiate from local mesenchymal stem cells^11^. Comparing expression profiles from single cultured pericytes with pericytes co-cultured with ECs clearly illustrated the importance of interaction with ECs in this differentiation process. Expression of Glioma-associated oncogene 1 (GLI1), a zinc finger type transcription factor and downstream effector of the Hedgehog signaling pathway that has been reported to be expressed in pericyte-like progenitors^12^, was significantly reduced in co-cultured pericytes (Fig. 3A). In contrast, well-validated pericyte markers, including Chondroitin Sulfate Proteoglycan 4 (CSPG4/NG2), Alpha Smooth Muscle Actin 2 (ACTA2), Melanoma Cell Adhesion Molecule (CD146), and Nestin (NES), were highly upregulated after direct interaction with ECs (Fig. 3B). Interestingly, Platelet Derived Growth Factor Receptor Beta (PDGFRβ), one of the most frequently used pericyte- and mesenchyme markers, was significantly downregulated in co-cultured pericytes (Fig. 3B).

### Endothelial signaling stimulates pericyte proliferation and survival

**Figure 4:**
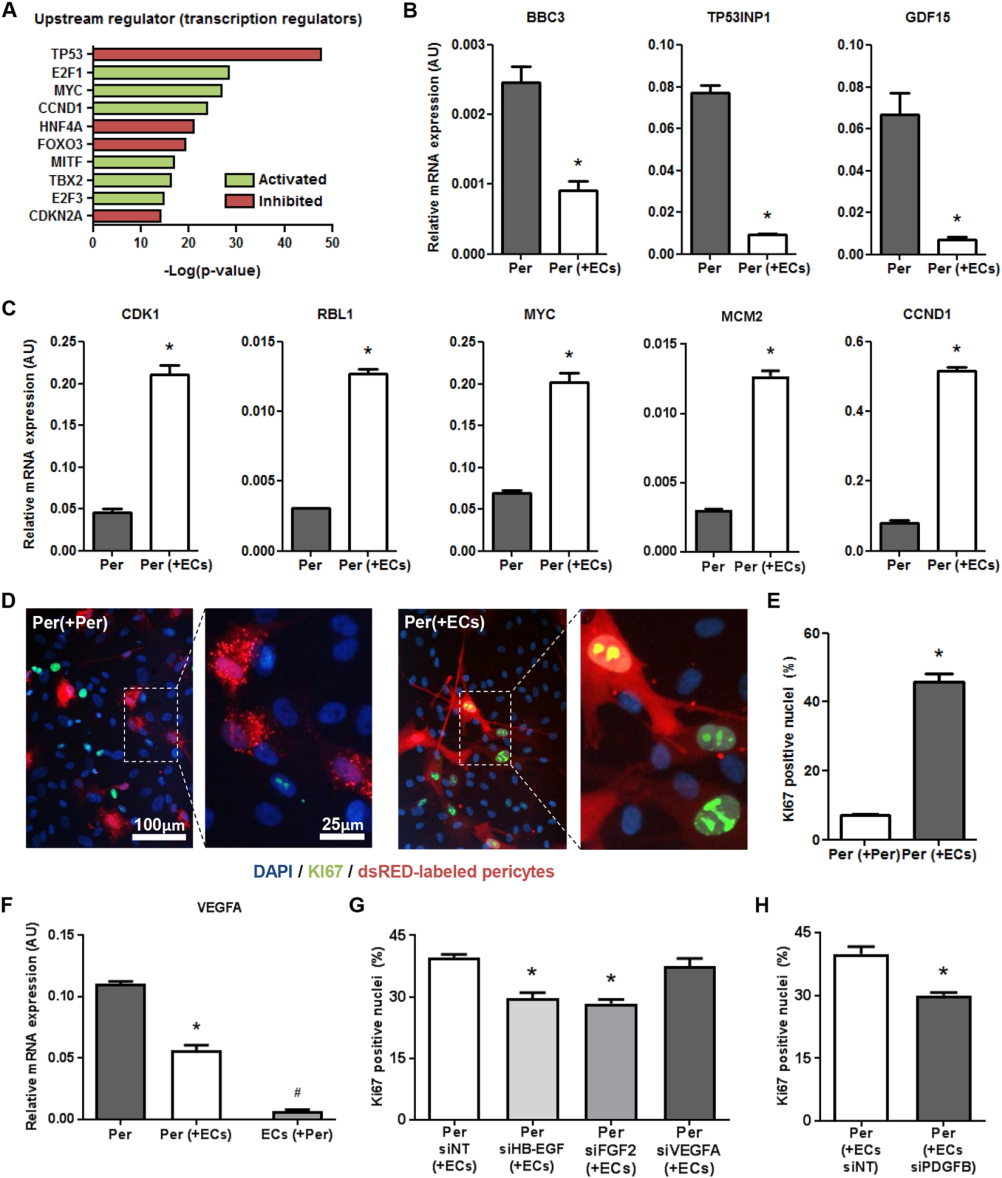
Endothelial signaling stimulates pericyte proliferation and survival. (**A)** IPA-derived prediction of activated or repressed transcription regulators in co-cultured pericytes compared with single cultured pericytes. Plotted is the −log(p-value) of overlap, in which green indicates predicted activation in co-culture versus predicted inhibition in red. **(B)** QPCR results showing expression levels of TP53 target genes BBC3, TP53INP1, and GDF15, as well as **(C)** E2F target genes CDK1, RBL1, MYC, MCM2 and CCND1 relative to RPLP0 and POLR2L in pericytes in monoculture (Per), and after 20 h in co-culture with HMVECs (Per + ECs). N = 3, *P < 0.05 compared to pericytes in monoculture. **(D)** Immunofluorescent Ki67 (green), and DAPI (blue) staining in dsRED-labeled pericytes, either cultured with unlabeled HMVECs (left) or with unlabeled pericytes (right). **(E)** Bar graph shows the quantified results of the Ki67 staining. Shown are the percentages of dsRED-labeled pericyte nuclei positive for the proliferation marker Ki67. N = 3, *P < 0.05 compared to dsRED-labeled pericytes in culture with unlabeled pericytes. **(F)** QPCR results showing expression levels of VEGFA in pericytes in monoculture (Per), and after 20 h in co-culture in both pericytes (Per + ECs) and HMVECs (ECs + Per). N = 3, *P < 0.05 compared to pericytes in monoculture and ECs in co-culture, ^#^P < 0.05 compared to pericytes in mono- and co-culture. **(G)** Bar graph showing the percentage of Ki67 positive nuclei in dsRED-labeled pericytes after knockdown of HB-EGF, FGF2, and VEGFA in pericytes, and **(H)** PDGFB in ECs. N = 4, *P < 0.05 compared to siNT-treated condition. BBC3: BCL2 Binding Component 3, TP53INP1: Tumor Protein P53 Inducible Nuclear Protein 1, GDF15: Growth Differentiation Factor 15, CDK1: Cyclin Dependent Kinase 1, RBL1: RB Transcriptional Corepressor Like 1, MYC: MYC Proto-Oncogene, MCM2: Minichromosome Maintenance Complex Component 2, CCND1: Cyclin D1, VEGFA: Vascular Endothelial Growth Factor A, NT: non-targeting, HB-EGF: Heparin-binding EGF-like growth factor, FGF2: Fibroblast Growth Factor 2.

Prior studies showed that pericyte-endothelial interaction had a profound positive effect on endothelial survival and maturation^13^. Interestingly, studying the upstream transcription factors of the differentially expressed genes using QIAGEN’s Ingenuity Pathway Analysis (IPA) led to the suggestion that direct interaction with ECs also stimulated pericyte proliferation and survival (Fig. 4A), which corresponds with functional findings reported previously^14^. Expression levels of 330 Tumor Protein 53- (TP53) dependent target genes in co-cultured pericytes (Supplemental Table 4), including BCL2 Binding Component 3 (BBC3), Tumor Protein P53 Inducible Nuclear Protein 1 (TP53INP1), and Growth Differentiation Factor 15 (GDF15), suggested suppressed activity of this pro-apoptotic and cell cycle inhibiting factor (Fig. 4B). Vice versa, the activity of proliferation-stimulating transcription factors E2F1 and E2F3 appeared to be enhanced in co-cultured pericytes. Expression of 139 E2F target genes, including Cyclin Dependent Kinase 1 (CDK1), Retinoblastoma Transcriptional Corepressor Like 1 (RBL1), Proto-Oncogene C-Myc (MYC), Minichromosome Maintenance Complex Component 2 (MCM2), and Cyclin D1 (CCND1), was significantly enhanced after direct interaction with ECs (Fig. 4C, Supplemental Table 5). To functionally validate the impact of endothelial contact on pericyte proliferation, we performed immunostaining for the proliferation (G1/S/G2M) marker Ki67 in dsRED-labeled pericytes, cultured in a confluent layer either with unlabeled HMVECs, or with unlabeled pericytes (Fig. 4D). In line with the observed transcriptional adaptations in co-cultured pericytes, quantification of the percentage of Ki67-positive nuclei in dsRED labeled pericytes clearly illustrated a significantly increased pericyte proliferation during co-culture with endothelium as compared with mono-cultured pericytes (Fig. 4E). It was previously reported that pericyte proliferation could be stimulated by VEGFA^15^, potentially via an autocrine signaling loop resulting from endothelium-induced upregulation of VEGFA expression in pericytes. In the present study however, a significant downregulation of VEGFA was observed in co-cultured pericytes, though the expression was still higher than in co-cultured ECs (Fig. 4F). To evaluate the involvement of pericyte-derived VEGFA in pericyte proliferation, as well as that of potent growth factors Heparin-binding EGF-like growth factor (HB-EGF) and fibroblast growth factor 2 (FGF2), which in contrast to VEGFA were upregulated in co-cultured pericytes, short interference RNA- (siRNA) mediated knockdown was performed in dsRED-labeled pericytes followed by coculture with ECs and Ki-67 immunostaining (Supplemental Fig. 1A,C). Quantification of the percentage of Ki67-positive nuclei in dsRED labeled pericytes revealed that knockdown of VEGFA had no effect on pericyte proliferation, whereas knockdown of HB-EGF and FGF2 significantly reduced the proliferative response of pericytes in co-culture with pericytes (Fig. 4G). Similarly, endothelial knockdown of PDGFB, a well-known pericyte mitogen that was upregulated in co-cultured ECs (Supplemental Fig. 1B,D), significantly reduced proliferation of pericytes in co-culture with ECs (Fig. 4H).

### Direct endothelial contact triggers outgrowth of pericyte projections

**Figure 5:**
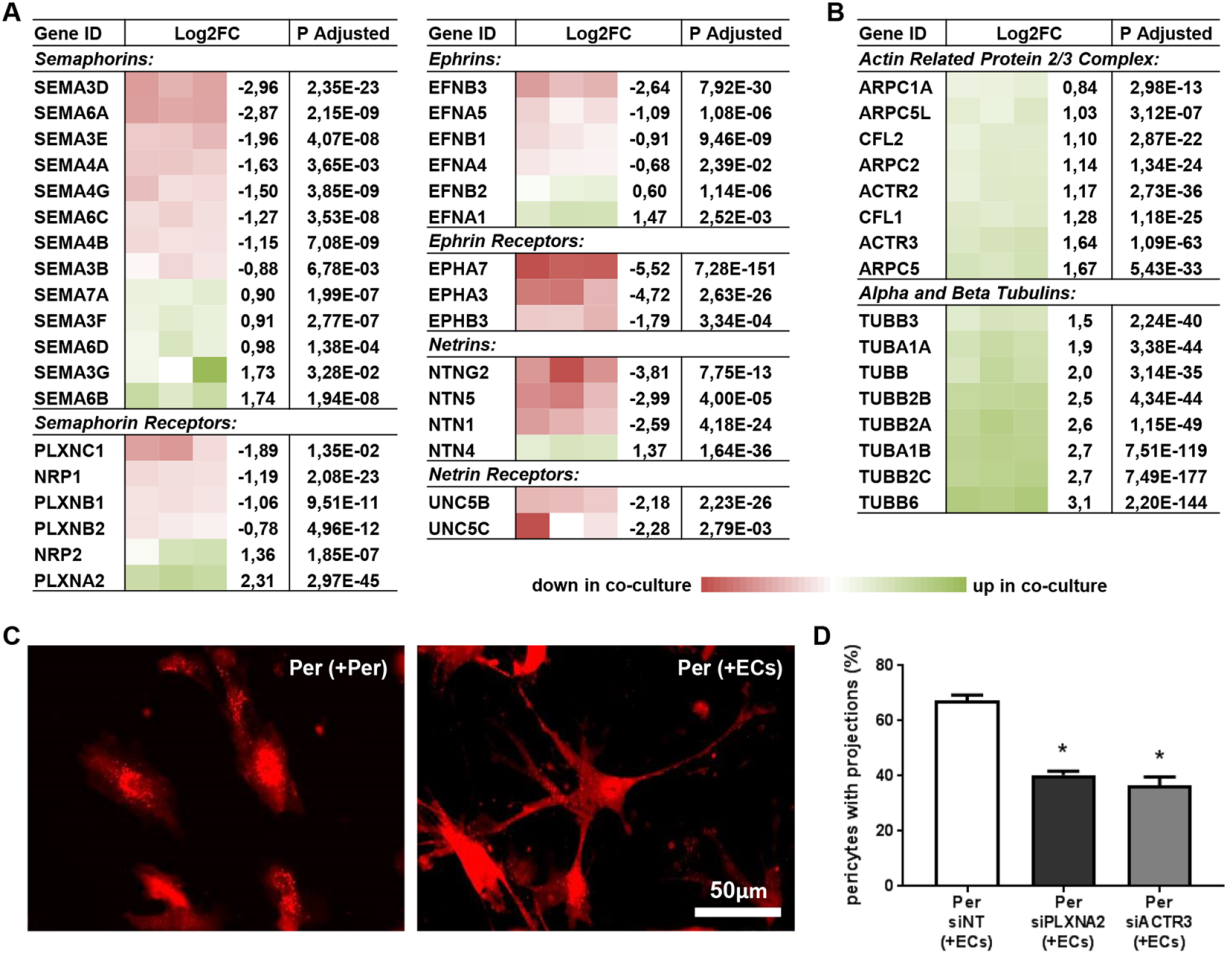
Direct endothelial contact triggers outgrowth of pericyte projections. (**A)** Schematic presentation of RNAseq data for differentially expressed Semaphorins, Ephrins, Netrins, and their respective receptors, as well as **(B)** subunits of the Actin Related Protein 2/3 Complex and Tubulins, which were all listed by IPA in a group of 200 differentially expressed genes involved in projection outgrowth. Shown is a color-based representation of the log2FC in each of the 3 individual experiments (red is downregulated in co-culture, green is upregulated in co-culture), followed by the average log2FC and the adjusted p-value. **(C)** Representative fluorescence microscopy images of dsRED-labeled pericytes in co-culture with unlabeled pericytes (left), or with unlabeled HMVECs (right). **(D)** Bar graph showing the percentage of dsRED-labeled pericytes with outgrowth of projections after knockdown of PLXNA2 and ACTR3 in pericytes. N = 4, *P < 0.05 compared to siNT-treated condition. NT: non-targeting, PLXNA2: Plexin A2, ACTR3: Actin Related Protein 3.

Mature pericytes have a highly specialized morphology that is distinctly different from other microvascular cells. They align their nuclei with the endothelium and extent thin processes along and around the capillaries. These elongated projections allow individual pericytes to contact and communicate with multiple ECs and were recently shown to have an active role in maintaining normal cerebral microvascular lumen diameter^16^. These thin processes show morphological similarities with axons growing from neurons. Growing axons have a highly dynamic structure at the peripheral tip, called the growth cone, which senses the environment for attracting or repelling guiding cues. Following these cues, actin remodeling at the leading edge guides the growth cone, followed by a polymerizing and elongating bundle of microtubules^17^. Interestingly, an exceptionally high number of genes involved in this process was differentially expressed in co-cultured pericytes, as identified by IPA (Supplemental Table 6). Among these differentially expressed genes was a variety of different growth cone-guiding molecules, including Semaphorins (SEMA), Ephrins (EFN), Netrins (NTN), and their respective receptors (Fig. 5A). Many Alpha and Beta Tubulins (TUBA and TUBB, respectively), composing the core of the growing projections, were upregulated in co-cultured pericytes (Fig. 5B). Similarly, enhanced expression was observed for Cofilins (CFL) and all but one Actin Related Protein 2/3 Complex subunits (ARPC), which regulate actin polymerization required for growth cone dynamics (Fig. 5B)^18^. To verify whether these transcriptional adaptations were associated with morphological changes that resemble outgrowth of pericyte projections, dsRED-labeled pericytes were again cultured in a confluent layer for the duration of 20 h, either in combination with unlabeled HMVECs, or with unlabeled pericytes followed by fluorescent microscopy imaging. Interestingly, pericytes in direct contact with ECs had a completely different morphology, indeed forming extensive projections (Fig. 5C). To study whether the differentially expressed growth cone-guiding molecules could in fact be involved in the observed morphological adaptation in pericytes, a proof-of-principle approach was used in which Plexin A2 (PLXNA2) and Actin Related Protein 3 (ACTR3), both well expressed and highly upregulated in co-cultured pericytes (Supplemental Fig. 2A), were knocked down (Supplemental Fig. 2B). After culturing these siRNA-transfected pericytes in a confluent layer with HMVECs for the duration of 20 h, the percentage of protrusion-forming pericytes was quantified using fluorescence microscopy. Knockdown of both PLXNA2 and ACTR3 significantly reduced the relative number of pericytes with projections (Fig. 5D), substantiating the idea that direct interaction with ECs triggered a transcriptional response in pericytes necessary for morphological maturation.

### Endothelial interaction suppresses overall ECM production in pericytes

**Figure 6:**
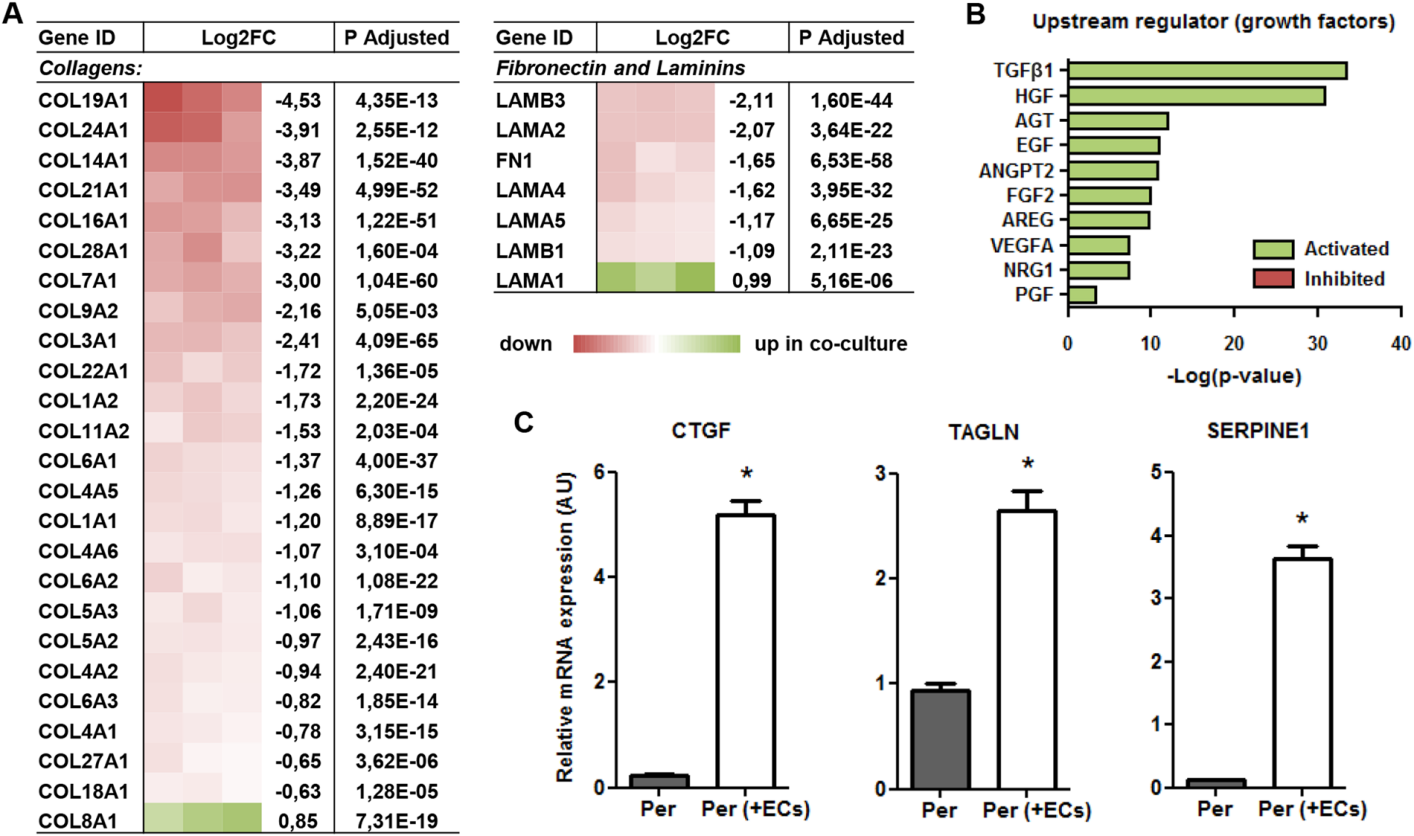
Endothelial interaction suppresses overall ECM production in pericytes. (**A**) Schematic presentation of RNAseq data for differentially expressed Collagens, Laminins, and Fibronectin. Shown is a color-based representation of the log2FC in each of the 3 individual experiments (red is downregulated in co-culture, green is upregulated in co-culture), followed by the average log2FC and the adjusted p-value. **(B)** IPA-derived prediction of the most activated or repressed growth factors in co-cultured pericytes compared with single cultured pericytes. Plotted is the −log(p-value) of overlap, in which green indicates predicted activation in co-culture versus predicted inhibition in red. (**C**) QPCR results showing expression levels of TGFβ target genes CTGF, TAGLN, and SERPINE1 relative to RPLP0 and POLR2L in pericytes in monoculture (Per), and after 20 h in co-culture with HMVECs (Per + ECs). N = 3, *P < 0.05 compared to pericytes in monoculture. CTGF: Connective Tissue Growth Factor, TAGLN: Transgelin, SERPINE1: Plasminogen Activator Inhibitor 1.

Recent studies showed that pericytes, detaching from the capillaries, not only leave the microvascular endothelium in a vulnerable position, but at the same time might undergo differentiation into ECM-producing cells themselves^5,19^. Whether this differentiation is a consequence of disrupted mutual cross-talk, or involves other extravascular signaling routes is not fully understood. Interestingly, the expression of 24 Collagen subtypes was significantly suppressed in pericytes co-cultured with endothelial cells, compared with mono-cultured pericytes (Fig. 6A). Moreover, a variety of other ECM components, including Fibronectin 1 (FN1), and Alpha- and Beta Laminins (LAMA and LAMB, respectively) was transcriptionally downregulated in co-cultured pericytes (Fig. 6A). This suppressed ECM expression in co-cultured pericytes might argue that differentiation into a fibrotic phenotype is indeed inhibited by direct interaction with ECs. Several studies have suggested that pericyte differentiation towards ECM producing cells depends on enhanced Transforming Growth Factor Beta (TGFβ) signaling^20,21^. However, in line with findings from many other studies (reviewed by Gaengel *et al*.)^22^, our findings indicated that pericytes in contact with ECs have much higher activation of TGFβ signaling than pericytes lacking interaction with ECs. In fact, based on 315 differentially expressed genes, IPA considered TGFβ1 (overlap p-value 3.56E-34) as the most active upstream growth factor in co-cultured pericytes (Fig. 6B, Supplemental Table 7). Other upregulated genes in co-cultured pericytes beside ACTA2, included Connective Tissue Growth Factor (CTGF), Smooth Muscle Protein 22 Alpha (TAGLN), and Plasminogen Activator Inhibitor 1 (SERPINE1), all of which are well-known TGFβ target genes (Fig. 6C). These findings illustrate that, even in the presence of activated TGFβ signaling, ECM excretion by pericytes is suppressed when in contact with endothelium.

### Destabilizing key gap- and adherens junctions has no effect on transcriptional adaptation

**Figure 7:**
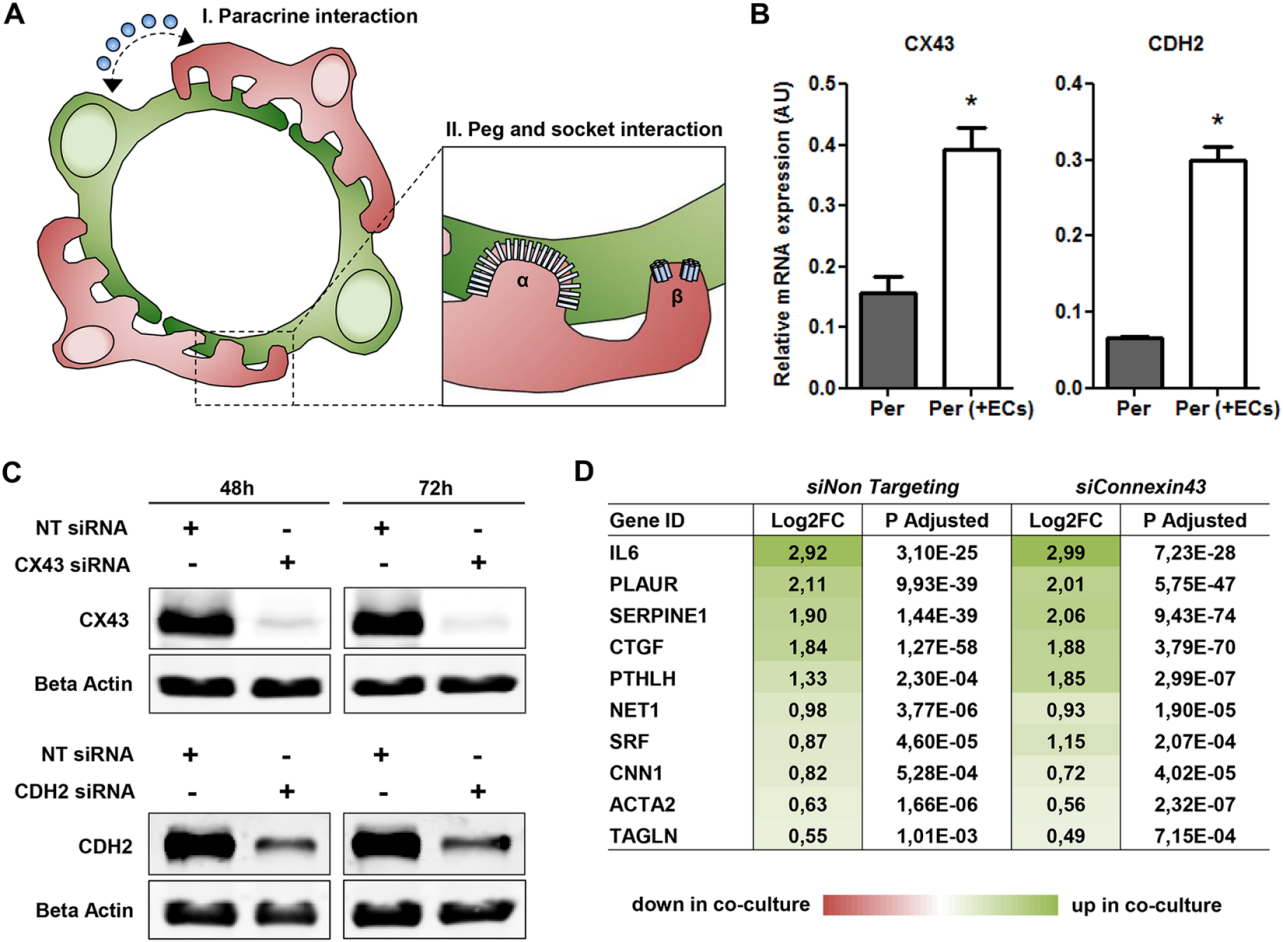
Destabilizing key gap- and adherens junctions does not affect transcriptional adaptation. (**A**) Pericytes (red) and ECs (green) can either interact via paracrine interaction (I) or via direct interaction at peg and socket contacts (II). These contacts are enriched in adherens junction protein CDH (α) and gap junction protein CX43 (β). **(B)** QPCR results showing expression levels of CX43 and CDH2 relative to RPLP0 and POLR2L in pericytes in monoculture (Per), and after 20 h in co-culture with HMVECs (Per + ECs). N = 3, *P < 0.05 compared to pericytes in monoculture. **(C)** Representative Western blots of CX43, CDH2, and β-actin levels in pericyte lysates at 48 h and 72 h post-transfection (full length blots in Supplemental Fig. 3A,B). N = 4. **(D)** Schematic presentation of RNAseq data for differentially expressed TGFβ target genes. Shown is a color-based representation of the log2FC (red is downregulated in co-culture, green is upregulated in co-culture), followed by the adjusted p-value. N = 3. NT: non-targeting, CX43: Connexin 43, CDH2: N-Cadherin.

In the mature microvasculature, pericytes and ECs form direct connections called peg and socket interactions. These sites are enriched in CDH2 and CX43 adherens- and gap junctions, respectively, which provide a direct signaling route for ions, nutrients, metabolites, and secondary messengers, acting complementary to paracrine signaling routes (Fig. 7A). This direct signaling was previously reported to play a key role in a particular aspect of endothelium-induced mural cell differentiation^23^. Interestingly in the present study, both CDH2 and CX43 were significantly upregulated in co-cultured pericytes, as validated by qPCR, suggesting enhanced requirement and thus signaling via these direct contacts in co-cultured pericytes (Fig. 7B). This raised the question to what extent this signaling was mandatory for pericyte differentiation. To address this question, we took a similar approach as listed in Fig. 1, but pericytes were now treated with non-targeting (NT) siRNA, CHD2-targeting siRNA, or CX43-targeting siRNA, to disrupt the main gap- and adherens junctions, followed by RNA sequencing. Knockdown efficiency was validated by Western blot for both CDH2 and CX43 (Fig. 7C, full length blots in Supplemental Fig. 3A,B). Expression profiles of co-cultured pericytes treated with NT siRNA were compared with that of co-cultured pericytes with either CDH2- or CX43 knockdown. It was observed that, besides siRNA-mediated downregulation of CDH2 and CX43, only KRT8 was differentially expressed in co-cultured pericytes with suppressed CX43 expression compared with co-cultured pericytes treated with NT siRNA (Log2FC: 0.54, adjusted p-value: 2.43E-02). KRT8 however was also upregulated in single cultured pericytes after CX43 knockdown (Log2FC: -0.52, adjusted p-value: 1.28E-02), indicating this was not related to destabilized interaction among ECs and pericytes. This also implicates that, in contrast to what has previously been reported, lack of CX43 expression thus did not block TGFβ-induced mural cell maturation. In co-cultured pericytes treated with CX43-targeting siRNA, an evident upregulation was still observed for known TGFβ target genes CTGF, ACTA2, TAGLN, and SERPINE1 (Fig. 7D).

## Discussion

The main findings of the current study are: (1) Interaction with ECs drives pericyte differentiation and maturation. (2) ECs stimulate pericyte proliferation and survival. (3) Direct endothelial contact stimulates the outgrowth of pericyte projections, and (4) represses overall ECM production in these perivascular cells. (5) Key gap- and adherens junctions genes CX43 and CDH2 do not appear to be involved in endothelium-induced differentiation of pericytes.

Pericytes have previously been described to be multipotent cells^24,25^. Thus, Crisan *et al*. reported that pericytes from multiple human organs, isolated using a combination of surface markers (including NG2, CD146, and PDGFRβ), were myogenic, osteogenic, chondrogenic, and adipogenic^26^. In light of these findings, it is not unexpected that direct interaction with ECs, resembling the physiological microvascular situation, triggers a defined adaptation in gene expression in these highly plastic cells when compared with single cultured pericytes. At the same time, these findings also align with studies showing that disease-induced loss of EC-pericyte interaction has a major impact on pericyte behavior. Among the differentially expressed genes in co-cultured pericytes there was a variety of well-known pericyte markers. The enhanced transcription of these markers was consistent with previous findings showing that endothelial contact with pericytes or mesenchymal progenitor cells plays a major role in their differentiation towards mature pericytes^12,27^. Remarkably, besides all upregulated pericyte markers, one of the most frequently used pericyte markers, PDGFRβ, was downregulated in pericytes after direct contact with ECs. PDGFRβ is a membrane-bound receptor with high affinity for PDGF subunits B and D^28^. PDGFB is abundantly expressed in ECs, and during vascular development, endothelial-derived PDGFB triggers pericyte proliferation and migration towards a newly formed vessel to effectuate pericyte-induced vascular stabilization^29^. Noteworthy, PDGFRβ expression is highly dependent on cell cycle activation, in a way that actively dividing cells have suppressed expression of the receptor^30^. The transcription factor MYC, actively involved in cell cycle progression, was identified as a key repressor of the PDGFRβ promoter^31,32^. Co-cultured pericytes were validated by Ki67 immunostaining to be actively proliferating, and not only was their expression of MYC upregulated almost threefold, analysis using IPA also suggested active transcriptional modulation by MYC in co-cultured pericytes (overlap p-value 1.03E-27). It is therefore likely that, among all the upregulated pericyte markers, the observed proliferative response in pericytes after direct contact with ECs triggers the – perhaps somewhat counterintuitive – suppression of PDGFRβ.

The enhanced proliferation in pericytes after contact with ECs supports the work of Tarallo *et al*.^14^. Using cell culture inserts, on which pericytes and ECs were cultured on either side, they found that ECs enhanced the number of pericytes, albeit after a relatively long incubation of 8 days. Taking into consideration that TGFβ was confirmed by IPA to be the most active upstream growth factor inducing the observed transcriptional response, the increased proliferation was remarkable as TGFβ has been reported in multiple different studies to have an inhibiting effect on this process^33,34^. This implies that other mitogenic factors must have compensated for the TGFβ-induced proliferation stop. VEGFA was previously found to directly induce proliferation in pericytes^15^. However, in contrast to the findings of Darland *et al*., who demonstrated that ECs induced VEGFA expression in pericytes^35^, a significant downregulation of VEGFA was found in co-cultured pericytes in the present study. In combination with the significantly lower expression of VEGFA in ECs, it therefore seemed unlikely that the enhanced proliferation in co-cultured pericytes was VEGFA-dependent. Moreover, several other mitogens, that were either highly expressed in ECs or showed elevated transcription in pericytes after interaction with ECs, have been reported for pericytes, including the aforementioned PDGFB, FGF2 and HB-EGF^36–38^. Both FGF2 and HB-EGF were significantly upregulated in pericytes co-cultured with ECs, and based on the observed transcriptional response IPA also listed these factors among the most active upstream growth factors (overlap p-values 9.53E-11 and 1.02E-11, respectively). Interestingly, siRNA-mediated knockdown of HB-EGF and FGF2 in pericytes, as well as PDGFB in HMVECs, significantly reduced Ki67 positivity of co-cultured pericytes, whereas levels of Ki67 were not affected in VEGFA siRNA-treated pericytes. These findings substantiate that, besides paracrine stimulation with the well-documented endothelial-derived PDGFB, direct contact with ECs triggers the transcription of these particular growth factors that could stimulate pericyte proliferation in an autocrine fashion.

The endothelium-induced differentiation also involved morphological adaptations. Pericytes cultured in the absence of ECs had a somewhat bipolar elongated shape, whereas pericytes cultured in the presence of ECs were characterized by long and thin projections, a morphology that resembles the physiological shape of mature pericytes, which project finger-like extension around the capillary wall. The observed morphological differences were accompanied by transcriptional adaption of particular genes. Individually, these genes can be involved in various cellular processes. Upregulation of tubulin isoforms for instance, is also observed during distinct phases of the cell cycle and migration^39^. The ARP2/3 complex, in concert with CFLs, has been shown to be involved in the dynamics of lamellipodia growth^40^, whereas many growth cone-guiding molecules have also been shown to be involved in vascular patterning, mostly affecting endothelial tip cell behavior. For example, signaling via the NRP receptor stimulates growth cone-collapse in the nervous systems in response to SEMAs^41^, yet they appear to induce endothelial tip-cell extension and vessel sprouting in the vascular system in response to VEGF^42^. Similarly, signaling of NTNs via UNC-5 Netrin Receptors (UNC5), downregulated in co-cultured pericytes, was shown to have a repulsive effect on endothelial branching^43,44^, and Eph receptors and EFNs were reported to be involved in segregation of ECs with distinct arterial- or venous fates^45^. However, the fact that in co-cultured pericytes developing long cellular protrusions, 200 genes (overlap p-value 6.78E-13) associated with axonal guidance were differentially expressed, suggested that these signaling molecules may also have a profound effect on pericyte behavior. The hypothesized activation of growth cone-guiding in pericytes upon endothelial interaction was substantiated by the finding that siRNA-mediated downregulation of PLXNA2 and ACTR3, both upregulated in co-cultured pericytes, significantly reduced the relative number of pericytes with projections. The reduction of projection outgrowth after knockdown of ACTR3 was not unexpected considering its role in onset of actin filament formation^46^. However, PLXNs generally transduce the inhibitory effect of SEMAs on axon outgrowth^47^, and consequently, loss of PLXNA2 was expected to actually stimulate outgrowth of pericyte projections. In ECs and U87MG glioblastoma cells, however, it was demonstrated that stimulation with SEMA3B and SEMA6A only in presence of PLXNA2 induced localized disassembly of the actin cytoskeleton and focal adhesion points, followed by cell contraction and the appearance of thin projections that remain attached to the substrate around the contracted cell^48^, yielding a morphological resemblance with the co-cultured pericytes. To date, little is known about the growth cone-guiding signaling pathways in pericytes, yet the associated morphological changes may be of great importance for vascular integrity, as was recently shown in the adult mouse brain, where local ablation of pericytes provoked resident pericytes to extend the tips of their projections to cover the endothelium and to restore local vascular function^16^.

The present study further provides evidence for an endothelium-induced suppression of overall ECM production in pericytes. This finding partially contrasts with the study of Stratman *et al*., in which basement membrane ECM expression was investigated in single-cultured and co-cultured human umbilical vein ECs and bovine pericytes in a collagen matrix^49^. Similar to our findings, they observed a defined downregulation of FN1 in pericytes cultured in the presence of ECs, yet a variety of other ECM molecules, including COL4A1 and COL4A2, demonstrated increased transcription levels in co-cultured pericytes. These apparent inconsistencies in transcriptional response are likely the result of differences in experimental approach, however, since we found that Glyceraldehyde-3-Phosphate Dehydrogenase, used by Stratman *et al*. as reference gene, was differentially expressed in co-cultured pericytes, it is difficult to draw definite conclusions. The findings on ECM expression do however appear to be in line with the many studies demonstrating that, upon organ injury, pericytes detach and migrate from the endothelium and differentiate into ECM-producing cells^5,12,19,50^. The observed suppression of ECM production in co-cultured pericytes, however, went paradoxically along with enhanced TGFβ signaling. This enhanced TGFβ signaling itself is not unexpected, as several studies demonstrated that direct contact of pericytes with ECs triggers the activation of latent TGFβ, thereby inducing a swift activation of TGFβ-mediated signaling^51^. The paradox lies in the fact that TGFβ is well known for its role in transforming pericytes into ECM-producing fibroblasts^52^. These findings thus illustrate that, even in the presence of activated TGFβ signaling, ECM production by pericytes is suppressed when in contact with endothelium, implying that either the actual level of TGFβ determines healthy or pathological pericyte differentiation, or that a thus far unknown endothelial-derived cue must be counteracting TGFβ-induced differentiation of pericytes into fibrotic cells.

Beside secreted signaling molecules, such as VEGFA and PDGFB, ECs and pericytes also interact in a more direct physical manner via peg and socket contacts, which are enriched in CX43 and CDH2^8,9^. In the present study, the expression of these molecules was suppressed in pericytes to assess if, and which, transcriptional adaptations in pericytes upon co-culture with ECs were regulated via these direct contacts. Unexpectedly, transcriptional comparison of co-cultured pericytes treated with NT siRNA and co-cultured pericytes with suppressed CX43 or CDH2 expression did not reveal significant differences. Hirschi *et al*. demonstrated that CX43 deficient mesenchymal cells lost their ability to activate latent TGFβ, and as a result were unable to differentiate into mature mural cells^23^. This is in contrast to our study where we did not observe any effect on TGFβ-mediated transcription in pericytes with suppressed CX43 expression. Our results suggest that endothelium-induced pericyte differentiation is not mediated by key gap- and adherens junctions CX43 and CDH2.

When exposing cells to a particular stimulus, a transcriptional response usually develops within hours. However, part of this response may depend on the formation of physical interactions that could require more than a few hours to develop. This led us to extend the incubation time to 20 h, which is well beyond the moment at which we could optically perceive morphological adaptation in the form of protrusions from pericytes extending to multiple ECs. It is hard to determine if at that particular moment communication between both cell types used the full spectrum of interaction channels, but it was long enough to induce a considerable response and it enabled us to verify that the observed TGFβ-mediated differentiation did not depend on CX43 presence. Using a set of more than 200 brain mural cell-enriched genes, identified by He *et al*.^53^, we also performed a gene set enrichment analysis (GSEA) on the RNAseq dataset to evaluate whether the presence of ECs pushed the pericytes into a phenotype resembling that of freshly isolated mature pericytes^54^. This GSEA illustrated that there was an enrichment of mature brain mural cell markers among the genes upregulated in pericytes cultured in the presence of ECs (Supplemental Fig. 4), substantiating the translatability of these findings, and further illustrating the endothelial-dependency of pericyte maturation.

In conclusion, the present study provides important evidence for pericyte differentiation upon interaction with ECs, by showing endothelium-induced pericyte maturation and proliferation, and suppression of ECM expression. However, functional gap- and adherens junctions do not appear to be involved in this process.

## Methods

### Cell culture

HMVECs (Lonza) and human brain-derived pericytes (Sciencell) were cultured on 0.1% gelatin coated plates in EGM-2MV medium (EBM2 medium supplemented with EGM-2MV bullet kit; Lonza) and DMEM (supplemented with 10% fetal calf serum (FCS); Lonza), respectively, in 5% CO_2_ at 37 °C. HMVECs were transduced with a lentiviral GFP construct and pericytes with a lentiviral dsRED construct. Experiments were performed with cells at passage 4–6. CDH2, CX43, HB-EGF, FGF2 and VEGFA, PLXN2A, ACTR3 knockdown in pericytes, and PDGFB knockdown in HMVECs was achieved by cell transfection with a pool containing 4 targeting siRNA sequences (Dharmacon) in a final concentration of 100 nM. Control cells were transfected with a pool of 4 non-targeting siRNA sequences (Dharmacon) in a final concentration of 100 nM. Target sequences are listed in Supplemental Table 1.

### Fluorescence-activated cell sorting

GFP-labeled HMVECs and dsRED-labeled pericytes were seeded in a confluent layer (4 million cells) on 10 cm dishes, either in co-culture at a 5:1 (HMVECs:pericytes) ratio, or in single culture (Fig. 1A). Both single cultures and co-cultures were cultured on EBM supplemented with 5% FCS for 6 h, followed by 20 h on EBM supplemented with 0.5% FCS. For fluorescence activated cell sorting (FACS), cells were trypsinized, washed once in cold PBS and suspended in cold sterile filtered PBS containing 2% bovine serum albumin. GFP-labeled HMVECs and dsRED-labeled pericytes were separately sorted in cold FCS based on their fluorescent signal. Subsequently, cells were washed once in cold PBS, lysed in RNA lysis buffer and stored at −80 °C (Fig. 1B).

### RNA sequencing

RNA sequencing was done as previously described^55^. Briefly, sequencing libraries were made from poly-adenylated RNA using the Rapid Directional RNA-Seq Kit (NEXTflex) and sequenced on Illumina NextSeq500 to produce single-end 75 base long reads (Utrecht Sequencing Facility). Reads were aligned to the human reference genome GRCh37 using STAR version2.4.2a. Read groups were added to the compressed binary version of the sequence alignment file (*.bam) files using Picard’s “AddOrReplaceReadGroups” tool (v1.98). The bam files were sorted with Sambamba v0.4.5, and transcript abundances were quantified with HTSeq-count version 0.6.1p117 using the union mode. Subsequently, reads per kilobase of transcript per million reads sequenced were calculated with edgeR’s rpkm() function. Differentially expressed genes in the transcriptome data were identified using the DESeq. 2 package with standard settings^56^. PCA was performed in R, using the plotPCA() command. Reads were normalized for sequencing depth and rlog transformed.

### Quantitative PCR and Western blot analysis

RNA was reverse transcribed into cDNA using SensiFast cDNA synthesis kit (Bioline). Gene expression was assessed by qPCR using SensiFast SYBR & Fluorecein kit (Bioline) and primers as listed in Supplemental Table 2. Expression levels are relative to the housekeeping gene Ribosomal Phosphoprotein P0 (RPLP0) and RNA Polymerase II Subunit L (POLR2L). For assessment of protein levels, cells were lysed in cold NP-40 lysis buffer (150 mM NaCl, 1.0% NP-40, 50 mM Tris, pH 8.0) supplemented with 1 mM β-glycerolphosphate, 1 mM PMSF, 10 mM NaF, 1 mM NaOV, and protease inhibitor cocktail (Roche). Total protein concentration was quantified by Pierce^®^ BCA Protein Assay Kit (Thermo Scientific) as a loading control. Lysates were denaturated in Laemmli buffer (60 mM Tris pH 6.8, 2% SDS, 10% glycerol, 5% β-mercaptoethanol, 0.01% bromophenol blue) at 90 °C for 5 min followed by electrophoresis on a 10% SDS-PAGE gel (Biorad). Subsequently, proteins were transferred to a nitrocellulose membrane (Pierce) and incubated for 1 hour in PBS with 5% non-fat milk, followed by incubation with rabbit anti-CX43 and rabbit anti-N-cadherin (CST) according to manufacturer’s description. Protein bands were visualized with the Li-Cor detection system (Westburg).

### Immunofluorescent staining

Pericytes labeled with dsRED were seeded in a confluent layer (50000 cells) in 96 wells plates in a 1:5 ratio, either with unlabeled HMVECs, or with unlabeled pericytes. After seeding, the cells were cultured on EBM supplemented with 5% FCS for 6 h, followed by 20 h on EBM supplemented with 0.5% FCS. After 20 h in culture, cells were fixed for 20 min in 2% paraformaldehyde and blocked for 60 min in PBS containing 5% bovine serum albumin (Sigma) and 0.3% Triton X-100 (Sigma). Hereafter, 100 μl PBS containing 1% BSA, 0.3% Triton X-100, and 1:400 rabbit anti-Ki67 antibody (CST) was added per well, followed by a 16 h incubation at 4 °C. After incubation with the primary antibody, Ki67 was stained with an Alexa Fluor 594-labeled secondary antibody (Invitrogen), dissolved (1:200) in PBS containing 1% BSA and 0.3% Triton X-100 for 2 h at room temperature. Vectashield with DAPI (Brunschwig) was applied to the fixed cell cultures, followed by imaging using fluorescence microscopy. Analysis of Ki67 positive pericyte nuclei was averaged per individual experiment from at least 5 random image fields in ImageJ using the Cell Counter plugin.

### Pathway analysis

RNAseq results were analyzed using IPA. IPA was used to study upstream regulators (growth factors and transcriptional regulators) of differentially expressed genes. P-values were calculated based on a right-tailed Fisher Exact Test, calculated by IPA.

### Statistics

Data are presented as means ± SEM. Groups were compared by students t-test (two-tailed) or 1-way ANOVA followed by Tukey post hoc test when appropriate. Statistical significance was accepted when p < 0.05.

## Supplementary information

Supplementary figures

Supplementary tables

## Data Availability

The data that support the findings of this study are available from the corresponding author upon reasonable request.
