# Supplementary material for: Transcriptome analysis reveals microvascular endothelial cell-dependent pericyte differentiation
Source: Sci Rep. 2019 Oct 30;9:15586. doi: 10.1038/s41598-019-51838-x (PMC6821775; doi:10.1038/s41598-019-51838-x)
Supplement: Supplementary file 1 — Supplementary figures [file 41598_2019_51838_MOESM1_ESM.docx]

**Transcriptome analysis reveals microvascular endothelial cell-dependent pericyte differentiation**

Maarten M. Brandt^1^, Christian G.M. van Dijk^2^, Ranganath Maringanti^1^, Ihsan Chrifi^1^, Rafael Kramann^3,4^, Marianne C. Verhaar^2^, Dirk J. Duncker^1^, Michal Mokry^5,6^, Caroline Cheng^1,2^.

*^1^Experimental Cardiology, Department of Cardiology, Thoraxcenter, Erasmus MC, University Medical Center Rotterdam, Rotterdam, The Netherlands; ^2^Department of Nephrology and Hypertension, Division of Internal Medicine and Dermatology, University Medical Center Utrecht, Utrecht, The Netherlands; ^3^Division of Nephrology and Clinical Immunology, RWTH Aachen University Medical Faculty, RWTH Aachen University, Aachen, Germany; ^4^Department of Internal Medicine, Nephrology and Transplantation, Erasmus University Medical Center, Rotterdam, The Netherlands; ^5^Epigenomics facility, University Medical Center Utrecht, Utrecht; ^6^Regenerative Medicine Center Utrecht, University Medical Center Utrecht, Utrecht; The Netherlands*

**Corresponding author:**

Caroline Cheng, PhD

University Medical Center Utrecht

PO Box 85500, 3508 GA Utrecht, The Netherlands

T: +31 (0)-88-7557329

E-mail: K.L.Cheng-2@umcutrecht.nl

**Supplemental figures**

**
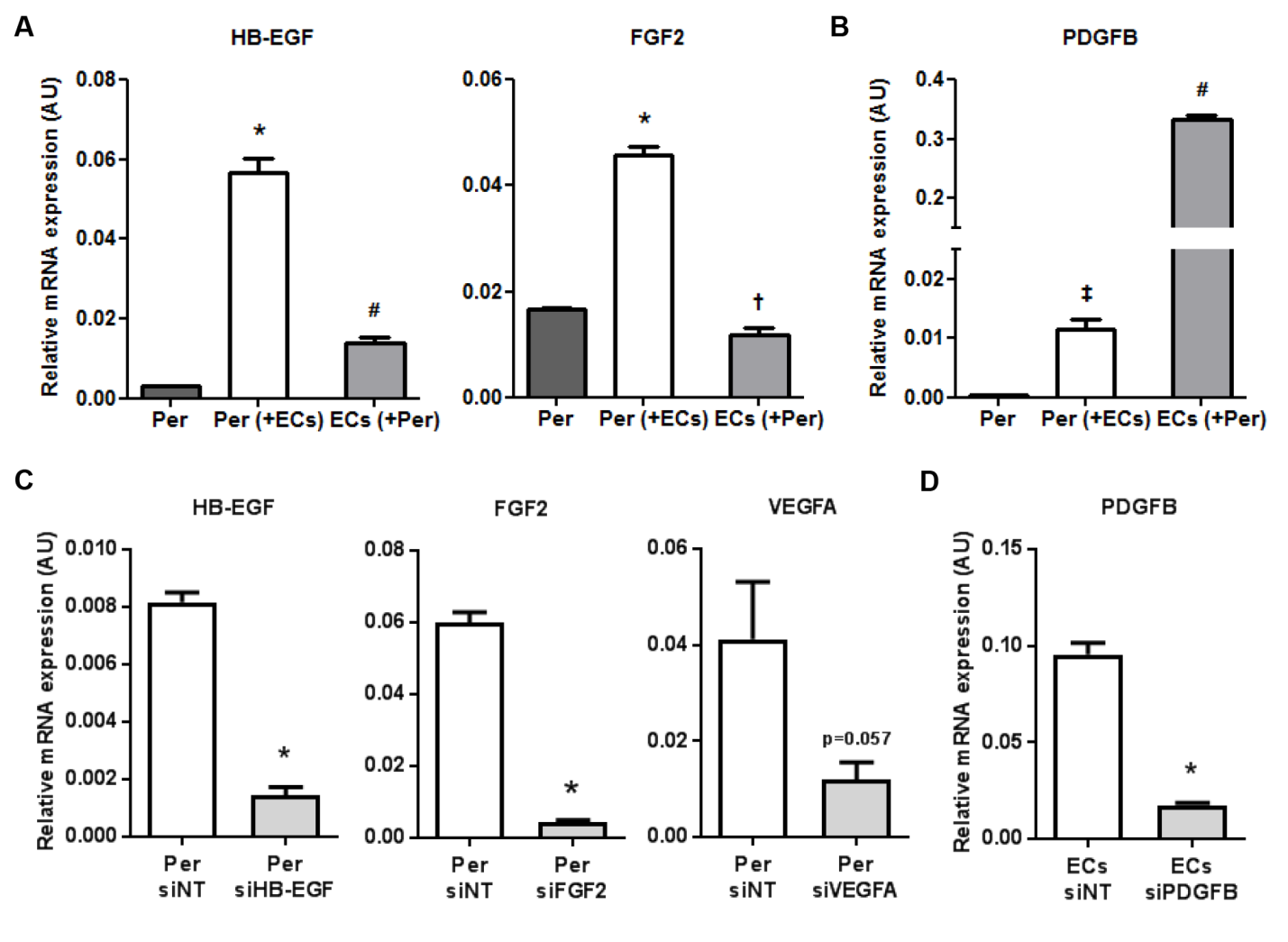
**

**Supplemental figure 1: Endothelial PDGFB, and pericyte HB-EGF and FGF2, are upregulated in co-cultured cells and were, like VEGFA, downregulated by siRNA. (A)** QPCR results showing expression levels of HB-EGF, FGF2, and PDGFB relative to RPLP0 and POLR2L in mono- and co-cultured pericytes and co-cultured ECs. N=3, * P<0.05 compared to pericytes in monoculture and ECs in co-culture, ^#^ P<0.05 compared to pericytes in mono- and co-culture, ^‡^ P<0.05 compared to ECs in co-culture, ^†^ P<0.05 compared to pericytes in co-culture. **(C)** QPCR results showing expression levels of HB-EGF, FGF2 and VEGFA in pericytes, as well as **(D)** PDGFB in ECs relative to RPLP0 and POLR2L. N=3-4, * P<0.05 compared to siNT-treated control.

**
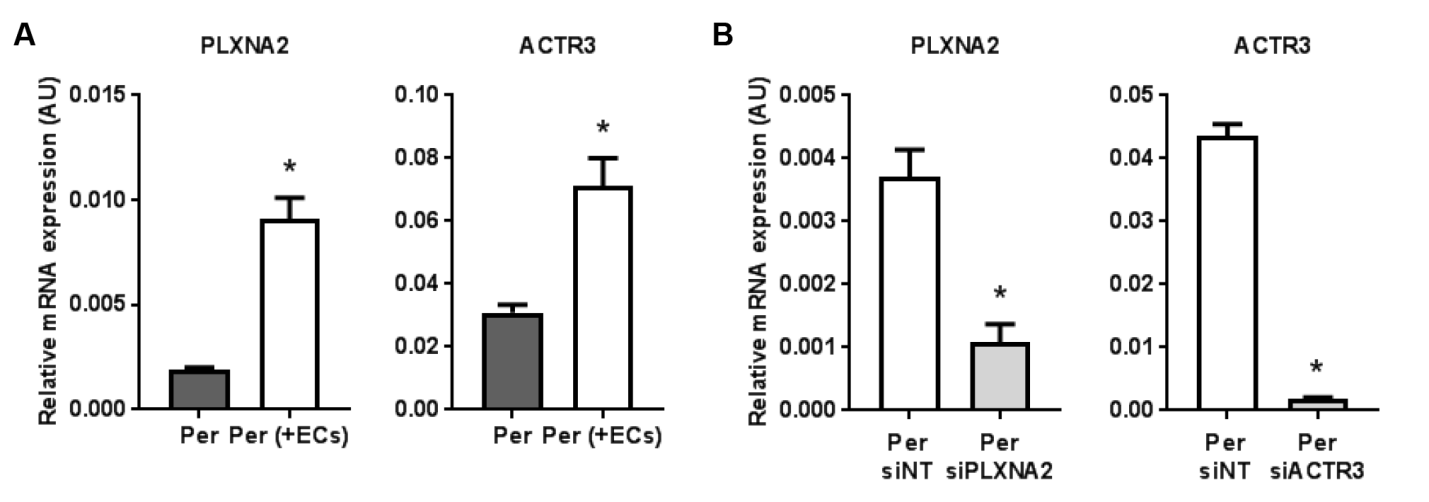
Supplemental figure 2: PLXNA2 and ACTR3 are upregulated in co-cultured pericytes and were downregulated by siRNA. (A)** QPCR results showing expression levels of PLXNA2 and ACTR3 relative to RPLP0 and POLR2L in mono- and co-cultured pericytes. N=3, * P<0.05 compared to pericytes in monoculture. **(B)** QPCR results showing expression levels of PLXNA2 and ACTR3 relative to RPLP0 and POLR2L in siRNA treated pericytes. N=3-4, * P<0.05 compared to siNT-treated control.


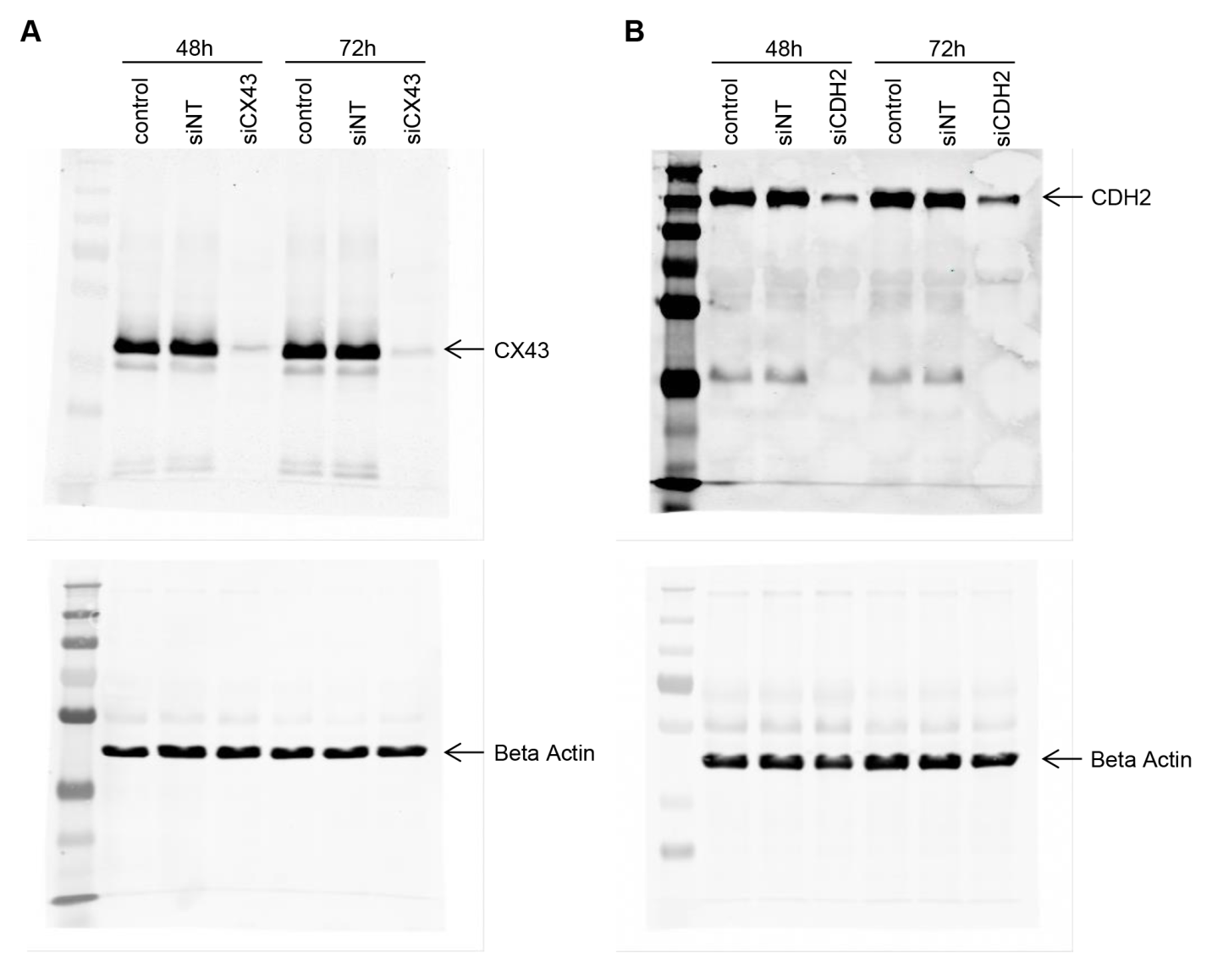


**Supplemental figure 3: Full length Western blots showing expression levels of CX43 and CDH2. (A)** Full length Western blots of CX43 and β-actin in untreated controls, non-targeting siRNA treated- (siNT) and CX43-targeting siRNA treated pericyte, 48h and 72h post-transfection. **(B)** Full length Western blots of CDH2 and β-actin in untreated controls, non-targeting siRNA treated- (siNT) and CDH2-targeting siRNA treated pericyte, 48h and 72h post-transfection.


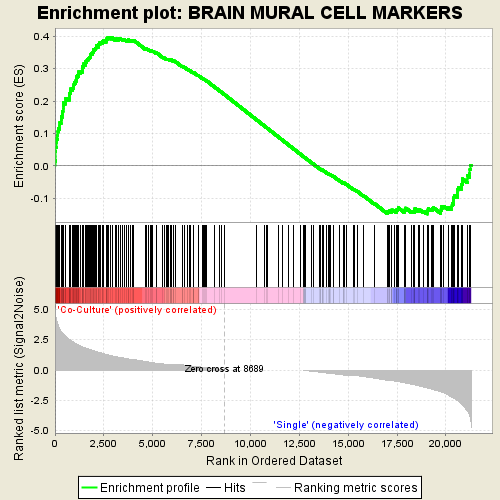


**Supplemental figure 4: Gene set enrichment analysis (GSEA).** Enrichment analysis of markers from freshly isolated mature mural cells in the RNAseq dataset illustrates enrichment of these markers in genes upregulated in pericytes when cultured in presence of ECs. Normalized enrichment score: 1.146, nominal p-value: 0.119, FDR q value: 0.235 (q<0.25 is generally considered as significant in GSEA).
