# Supplementary material for: Transcriptome analysis reveals microvascular endothelial cell-dependent pericyte differentiation
Source: Sci Rep. 2019 Oct 30;9:15586. doi: 10.1038/s41598-019-51838-x (PMC6821775; doi:10.1038/s41598-019-51838-x)
Supplement: Supplementary file 2 — Supplementary tables [file 41598_2019_51838_MOESM2_ESM.docx]

**Transcriptome analysis reveals microvascular endothelial cell-dependent pericyte differentiation**

Maarten M. Brandt^1^, Christian G.M. van Dijk^2^, Ranganath Maringanti^1^, Ihsan Chrifi^1^, Rafael Kramann^3,4^, Marianne C. Verhaar^2^, Dirk J. Duncker^1^, Michal Mokry^5,6^, Caroline Cheng^1,2^.

*^1^Experimental Cardiology, Department of Cardiology, Thoraxcenter, Erasmus MC, University Medical Center Rotterdam, Rotterdam, The Netherlands; ^2^Department of Nephrology and Hypertension, Division of Internal Medicine and Dermatology, University Medical Center Utrecht, Utrecht, The Netherlands; ^3^Division of Nephrology and Clinical Immunology, RWTH Aachen University Medical Faculty, RWTH Aachen University, Aachen, Germany; ^4^Department of Internal Medicine, Nephrology and Transplantation, Erasmus University Medical Center, Rotterdam, The Netherlands; ^5^Epigenomics facility, University Medical Center Utrecht, Utrecht; ^6^Regenerative Medicine Center Utrecht, University Medical Center Utrecht, Utrecht; The Netherlands*

**Corresponding author:**

Caroline Cheng, PhD

University Medical Center Utrecht

PO Box 85500, 3508 GA Utrecht, The Netherlands

T: +31 (0)-88-7557329

E-mail: K.L.Cheng-2@umcutrecht.nl

**Supplemental Table 1:** siRNA sequences used for knockdown experiments

| Target gene | Target sequence |
| --- | --- |
| Non-targeting | UGGUUUACAUGUCGACUAA |
|  | UGGUUUACAUGUUGUGUGA |
|  | UGGUUUACAUGUUUUCUGA |
|  | UGGUUUACAUGUUUUCCUA |
| CDH2 | GUGCAACAGUAUACGUUAA |
|  | GGACCCAGAUCGAUAUAUG |
|  | CAUAGUAGCUAAUCUAACU |
|  | GACAGCCUCUUCUCAAUGU |
| CX43 | CAGUCUGCCUUUCGUUGUA |
|  | UGACAAGGUUCAAGCCUAC |
|  | GUACAUCUAUGGAUUCAGC |
|  | GAACCUACAUCAUCAGUAU |
| PDGFB | CCGAGGAGCUUUAUGAGAU |
|  | GAAGAAGGAGCCUGGGUUC |
|  | GCAAGCACCGGAAAUUCAA |
|  | GGGCCGAGUUGGACCUGAA |
| HB-EGF | GAAAAUCGCUUAUAUACCU |
|  | UGAAGUUGGGCAUGACUAA |
|  | GGACCCAUGUCUUCGGAAA |
|  | GGAGAAUGCAAAUAUGUGA |
| FGF2 | CUAAAUGUGUUACGGAUGA |
|  | UCAAAGGAGUGUGUGCUAA |
|  | GCUAAGAGCUGAUUUUAAU |
|  | GAUGGAAGAUUACUGGCUU |
| VEGFA | GCAGAAUCAUCACGAAGUG |
|  | CAACAAAUGUGAAUGCAGA |
|  | GGAGUACCCUGAUGAGAUC |
|  | GAUCAAACCUCACCAAGGC |
| PLXNA2 | GGUCAAUGAGUGAGAUCGU |
|  | CUGGGAACCUAAUCGAUUU |
|  | CCAGCCAAGUUUCGUCAUA |
|  | ACUAUGAGCUACACAGCGA |
| ACTR3 | GCAGUAAAGGAGCGCUAUA |
|  | GUGAUUGGCAGCUGUAUUA |
|  | GGAAUUGAGUGGUGGUAGA |
|  | GCCAAAACCUAUUGAUGUA |

**Supplemental Table 2:** Primer sequences used for (q)PCR

| Gene | Sense primer sequence | Antisense primer sequence |
| --- | --- | --- |
| RPLP0 | CAGATTGGCTACCCAACTGTT | GGAAGGTGTAATCCGTCTCCAC |
| POLR2L | TCACTTGTGGCAAGATCGTCG | GGGTGCATAATTGAGCAGCTTC |
| GLI1 | TCCTACCAGAGTCCCAAGTTTC | GCCCTATGTGAAGCCCTATTT |
| NG2 | ACCTTCAACTACAGGGCACAAG | AGGACATTGGTGAGGACAGG |
| ACTA2 | ACTGAGCGTGGCTATTCCTTC | CAGGCAACTCGTAACTCTTCTC |
| CD146 | CTGCTGAGTGAACCACAGGA | CACCTGGCCTGTCTCTTCTC |
| NES | CCTGGGAAAGGGAGAGTACC | GATTCAGCTCTGCCTCATCC |
| PDGFRB | GAGGAATCCCTCACCCTCTC | GGGTATATGGCCTTGCTTCA |
| BBC3 | GACCTCAACGCACAGTACGAG | AGGAGTCCCATGATGAGATTGT |
| T53INP1 | TTCCTCCAACCAAGAACCAG | GATGCCGGTAAACAGGAAAA |
| GDF15 | CTCCAGATTCCGAGAGTTGC | AGAGATACGCAGGTGCAGGT |
| CDK1 | CTGGGGTCAGCTCGTTACTC | AGGCTTCCTGGTTTCCATTT |
| RBL1 | ATACGACTTGGCGAATCAGG | GAGCGCTTCTTGGTGTAAGG |
| MYC | CTCCTGGCAAAAGGTCAGAG | TCGGTTGTTGCTGATCTGTC |
| MCM2 | ATTTCGTCCTGGGTCCTTTCT | CTGGTTTCACCTCCTGGTTCT |
| CCND1 | GTCCATGCGGAAGATCGTCG | TCTCCTTCATCTTAGAGGCCACG |
| PDGFB | CCCCACACTCCACTCTGATT | GCCCTGGCCTCTAGTCTTCT |
| FGF2 | AGAGCGACCCTCACATCAAG | TCGTTTCAGTGCCACATACC |
| HB-EGF | GGTGGTGCTGAAGCTCTTTC | GCTTGTGGCTTGGAGGATAA |
| VEGFA | CATCCAATCGAGACCCTGG | ATGTGCTGGCCTTGGTGAG |
| PLXNA2 | AAAGGAAGAGGACCCCAGAA | GACAGCAAGCACAGAAGCAC |
| ACTR3 | CTGGGTTGCGGAAGTGATAG | TGTATACCCCGTGCCACAGT |
| CTGF | CCGTACTCCCAAAATCTCCA | GTAATGGCAGGCACAGGTCT |
| TAGLN | AAGAATGATGGGCACTACCG | ACTGATGATCTGCCGAGGTC |
| SERPINE1 | TCTTTGGTGAAGGGTCTGCT | TTGAATCTGCTGCTGGGTTT |
| CX43 | TGGATTCAGCTTGAGTGCTG | GGTCGCTCTTTCCCTTAACC |
| CDH2 | GACAATGCCCCTCAAGTGTT | CCATTAAGCCGAGTGATGGT |

**Supplemental Table 3:** Differentially expressed genes in co-cultured versus mono-cultured pericytes

| Gene ID | Refseq ID | Log2FC | P Adjusted |
| --- | --- | --- | --- |
| SERPINE1 | NM_000602 | -4,86 | 0,00E+00 |
| HHIP | NM_022475 | -5,02 | 2,06E-262 |
| NPTX1 | NM_002522 | 4,50 | 1,60E-244 |
| CYR61 | NM_001554 | -4,68 | 9,92E-222 |
| CTGF | NM_001901 | -4,40 | 9,96E-203 |
| ABCA8 | NM_007168 | 5,08 | 1,47E-186 |
| PLTP | NM_182676 | 3,66 | 1,16E-178 |
| ADAMTS15 | NM_139055 | 4,08 | 2,90E-178 |
| TUBB2C | NM_006088 | -2,75 | 7,49E-177 |
| ANLN | NM_018685 | -3,60 | 8,26E-175 |
| DHCR24 | NM_014762 | -3,38 | 2,43E-171 |
| INHBA | NM_002192 | -3,37 | 1,41E-158 |
| ODZ4 | NM_001098816 | 5,38 | 1,09E-152 |
| PVR | NM_001135768 | -3,21 | 2,00E-151 |
| EPHA7 | NM_004440 | 5,52 | 7,28E-151 |
| LRRC17 | NM_001031692 | 3,45 | 2,06E-149 |
| CRIM1 | NM_016441 | -2,97 | 1,63E-146 |
| SVIL | NM_021738 | 3,16 | 1,84E-146 |
| SLC16A3 | NM_001042423 | -3,83 | 8,36E-146 |
| TUBB6 | NM_032525 | -3,11 | 2,20E-144 |
| SLC7A5 | NM_003486 | -3,11 | 5,26E-143 |
| RGS4 | NM_001113381 | -2,78 | 1,16E-138 |
| PLA2R1 | NM_007366 | 4,40 | 1,51E-129 |
| IGDCC4 | NM_020962 | 3,99 | 4,03E-123 |
| CCND1 | NM_053056 | -2,53 | 4,67E-123 |
| VGLL4 | NM_001128219 | 2,51 | 1,49E-121 |
| TUBA1B | NM_006082 | -2,72 | 7,51E-119 |
| CEP55 | NM_018131 | -3,38 | 1,04E-117 |
| CALCOCO1 | NM_020898 | 2,81 | 1,89E-117 |
| RRM2 | NM_001165931 | -3,12 | 1,11E-116 |
| ARSJ | NM_024590 | -2,63 | 3,09E-116 |
| NCEH1 | NM_001146277 | -3,33 | 4,94E-116 |
| PRC1 | NM_003981 | -2,69 | 1,53E-114 |
| CCNA2 | NM_001237 | -3,10 | 8,01E-114 |
| ANKRD1 | NM_014391 | -4,90 | 2,02E-112 |
| DIAPH3 | NM_001042517 | -3,40 | 9,21E-112 |
| EMILIN2 | NM_032048 | -3,05 | 1,14E-106 |
| ADD3 | NM_019903 | 2,51 | 1,07E-102 |
| ADAMTS1 | NM_006988 | -3,08 | 4,71E-102 |
| ID3 | NM_002167 | -2,78 | 2,57E-101 |
| KPNA2 | NM_002266 | -2,38 | 1,22E-100 |
| HGF | NM_000601 | 4,92 | 4,67E-100 |
| CTSC | NM_148170 | -3,00 | 2,08E-99 |
| C14orf4 | NM_024496 | 2,75 | 2,08E-99 |
| MYBL2 | NM_002466 | -2,83 | 2,40E-99 |
| SMTN | NM_006932 | -2,66 | 3,75E-99 |
| OLFML2A | NM_182487 | 3,46 | 3,75E-99 |
| DCN | NM_133503 | 2,35 | 8,27E-98 |
| SGK493 | NM_138370 | 3,41 | 2,24E-96 |
| RCAN1 | NM_203418 | -2,87 | 3,92E-96 |
| TXNIP | NM_006472 | 2,32 | 4,04E-96 |
| PGPEP1 | NM_017712 | 3,79 | 2,83E-94 |
| SCG2 | NM_003469 | 4,63 | 3,42E-94 |
| CRYAB | NM_001885 | -3,36 | 6,59E-94 |
| SATB1 | NM_002971 | 3,30 | 7,83E-94 |
| KIF23 | NM_004856 | -2,90 | 9,37E-94 |
| RAB3B | NM_002867 | -4,30 | 9,92E-94 |
| MXRA5 | NM_015419 | 5,40 | 1,12E-93 |
| CORO1C | NM_014325 | -1,86 | 1,99E-93 |
| FBLN2 | NM_001004019 | 2,30 | 8,19E-93 |
| UBE2S | NM_014501 | -2,93 | 2,00E-92 |
| KRT7 | NM_005556 | -3,98 | 2,17E-92 |
| MARCH4 | NM_020814 | -4,85 | 2,56E-92 |
| CDC20 | NM_001255 | -2,83 | 5,12E-92 |
| ISYNA1 | NM_016368 | 2,47 | 4,22E-91 |
| PLK1 | NM_005030 | -2,99 | 6,81E-91 |
| LMNB2 | NM_032737 | -2,56 | 9,64E-91 |
| CYP1B1 | NM_000104 | 5,04 | 1,34E-90 |
| RAI14 | NM_001145522 | -2,17 | 3,31E-90 |
| ANKH | NM_054027 | 3,21 | 1,30E-89 |
| H2AFX | NM_002105 | -2,14 | 1,76E-89 |
| MCM10 | NM_018518 | -4,67 | 5,39E-89 |
| KANK2 | NM_001136191 | 2,21 | 3,35E-88 |
| ARHGAP28 | NM_001010000 | 4,19 | 3,77E-88 |
| FGF1 | NR_026695 | -5,60 | 1,43E-87 |
| DLC1 | NM_006094 | -2,25 | 3,01E-87 |
| YPEL3 | NM_031477 | 3,65 | 5,88E-87 |
| TYMS | NM_001071 | -2,57 | 8,67E-87 |
| WHSC1 | NM_133331 | -2,24 | 1,96E-86 |
| DDX21 | NM_004728 | -2,21 | 3,91E-86 |
| SHCBP1 | NM_024745 | -3,77 | 4,90E-86 |
| PRRT2 | NM_145239 | 3,75 | 3,61E-85 |
| CSPG4 | NM_001897 | -1,98 | 3,23E-84 |
| BUB1B | NM_001211 | -3,03 | 2,28E-83 |
| CPZ | NM_001014448 | 2,73 | 3,24E-83 |
| AURKA | NM_198433 | -2,99 | 8,40E-83 |
| SRGN | NM_002727 | -2,25 | 1,08E-82 |
| FOXM1 | NM_202002 | -2,69 | 1,59E-82 |
| PKP1 | NM_001005337 | -3,47 | 1,93E-82 |
| KIFC1 | NM_002263 | -3,00 | 3,69E-82 |
| AKAP2 | NM_001004065 | -1,88 | 8,53E-82 |
| DTL | NM_016448 | -3,72 | 8,95E-82 |
| PALM2-AKAP2 | NM_007203 | -1,88 | 1,18E-81 |
| BNIP3 | NM_004052 | -2,28 | 1,47E-81 |
| ITGA6 | NM_001079818 | -3,35 | 4,86E-81 |
| ATAD2 | NM_014109 | -2,72 | 1,70E-80 |
| CLSPN | NM_022111 | -3,49 | 1,87E-80 |
| TPCN1 | NM_001143819 | 2,37 | 2,60E-80 |
| TMPO | NM_003276 | -2,55 | 2,99E-80 |
| ECT2 | NM_018098 | -2,66 | 3,56E-80 |
| C14orf132 | NR_023938 | 2,42 | 8,02E-80 |
| ZIC2 | NM_007129 | 2,56 | 1,44E-79 |
| ARHGAP11A | NM_014783 | -2,57 | 3,35E-79 |
| HK2 | NM_000189 | -2,37 | 3,35E-79 |
| CCNB1 | NM_031966 | -2,80 | 1,92E-78 |
| UHRF1 | NM_013282 | -3,41 | 4,91E-78 |
| ABCA9 | NM_080283 | 5,59 | 6,19E-78 |
| MAF | NM_005360 | 4,00 | 2,19E-77 |
| RACGAP1 | NM_013277 | -2,32 | 2,86E-77 |
| C12orf75 | NM_001145199 | -2,85 | 4,22E-77 |
| NCAPG2 | NM_017760 | -2,63 | 1,70E-76 |
| CCDC99 | NM_017785 | -2,56 | 6,08E-76 |
| ABCA1 | NM_005502 | 4,22 | 7,52E-76 |
| MMP11 | NM_005940 | 3,43 | 1,54E-75 |
| CTPS | NM_001905 | -2,71 | 1,89E-75 |
| LRRC59 | NM_018509 | -1,84 | 2,24E-75 |
| G0S2 | NM_015714 | 4,28 | 2,94E-75 |
| MCM5 | NM_006739 | -2,46 | 3,59E-75 |
| TPM3 | NM_001043351 | -1,97 | 5,51E-75 |
| PLOD2 | NM_182943 | -1,98 | 1,37E-74 |
| PDGFRA | NM_006206 | 2,31 | 1,96E-74 |
| BUB1 | NM_004336 | -2,84 | 3,77E-74 |
| MAN1A1 | NM_005907 | 2,62 | 6,65E-74 |
| ARRDC3 | NM_020801 | 2,56 | 9,69E-74 |
| PFKP | NM_002627 | -2,22 | 6,55E-73 |
| CSRP1 | NM_004078 | -1,99 | 2,23E-72 |
| EZR | NM_001111077 | -2,47 | 7,26E-72 |
| CDCA8 | NM_018101 | -3,13 | 7,50E-72 |
| CORO2B | NM_006091 | 2,67 | 8,71E-72 |
| MCM4 | NM_182746 | -2,23 | 9,98E-72 |
| ABCC1 | NM_019862 | 1,88 | 2,93E-71 |
| ZNF503 | NM_032772 | 2,76 | 3,86E-71 |
| LRIG1 | NM_015541 | 2,07 | 9,95E-71 |
| PPL | NM_002705 | 7,11 | 2,10E-70 |
| RRM1 | NM_001033 | -2,16 | 2,25E-70 |
| ACAN | NM_001135 | -3,89 | 3,43E-70 |
| CENPE | NM_001813 | -2,41 | 3,87E-70 |
| LDLR | NM_000527 | -2,45 | 4,41E-70 |
| EIF4EBP1 | NM_004095 | -2,17 | 5,40E-70 |
| NP | NM_000270 | -2,67 | 5,57E-70 |
| ZCCHC24 | NM_153367 | 2,05 | 5,94E-70 |
| WDR62 | NM_001083961 | -2,74 | 1,58E-69 |
| KIF4A | NM_012310 | -2,70 | 4,05E-69 |
| ISLR | NM_005545 | 2,92 | 6,63E-69 |
| MAPK10 | NM_002753 | 4,56 | 7,81E-69 |
| EGR3 | NM_004430 | 4,58 | 1,15E-68 |
| IGFBP5 | NM_000599 | 3,38 | 2,33E-68 |
| RGMB | NM_001012761 | -1,87 | 5,31E-68 |
| DDAH1 | NM_012137 | -2,92 | 1,19E-67 |
| CDC6 | NM_001254 | -3,36 | 1,63E-67 |
| KLHL24 | NM_017644 | 3,39 | 1,64E-66 |
| ERCC2 | NM_000400 | -2,16 | 3,72E-66 |
| HSPD1 | NM_002156 | -1,70 | 3,73E-66 |
| HBEGF | NM_001945 | -4,04 | 5,98E-66 |
| SPAG5 | NM_006461 | -2,70 | 1,34E-65 |
| PKMYT1 | NM_182687 | -2,50 | 1,84E-65 |
| COL3A1 | NM_000090 | 2,41 | 4,09E-65 |
| HN1 | NM_001002033 | -2,32 | 4,62E-65 |
| ST5 | NM_005418 | 2,05 | 4,78E-65 |
| ENO1 | NM_001428 | -2,05 | 7,18E-65 |
| CCBE1 | NM_133459 | -3,25 | 1,84E-64 |
| LDHA | NM_001135239 | -1,95 | 5,40E-64 |
| C17orf103 | NM_152914 | 2,53 | 5,58E-64 |
| FOSB | NM_006732 | -4,37 | 9,26E-64 |
| ACTR3 | NM_005721 | -1,64 | 1,09E-63 |
| KLF6 | NR_027653 | -1,88 | 1,45E-63 |
| NCAPH | NM_015341 | -3,00 | 1,55E-63 |
| MCAM | NM_006500 | -2,98 | 2,25E-63 |
| H2AFZ | NM_002106 | -2,43 | 2,92E-63 |
| PLXDC1 | NM_020405 | 4,12 | 2,92E-63 |
| EHD4 | NM_139265 | -2,65 | 8,06E-63 |
| TP53INP1 | NM_033285 | 2,85 | 8,69E-63 |
| SORBS2 | NM_001145675 | -2,78 | 9,99E-63 |
| TPX2 | NM_012112 | -2,57 | 1,25E-62 |
| BMF | NM_033503 | 3,23 | 1,75E-62 |
| PCMTD1 | NM_052937 | 2,57 | 2,37E-62 |
| LRP1 | NM_002332 | 2,13 | 3,20E-62 |
| ACTG2 | NM_001615 | -2,63 | 5,87E-62 |
| KIF11 | NM_004523 | -2,31 | 1,11E-61 |
| PARP14 | NM_017554 | 2,31 | 3,89E-61 |
| TRIP13 | NM_004237 | -2,93 | 7,22E-61 |
| TGFB3 | NM_003239 | 3,58 | 7,83E-61 |
| FADS2 | NM_004265 | -2,68 | 8,81E-61 |
| PTPRS | NM_002850 | 1,93 | 9,05E-61 |
| COL7A1 | NM_000094 | 3,00 | 1,04E-60 |
| AURKB | NM_004217 | -2,80 | 1,22E-60 |
| ZNF608 | NM_020747 | 3,06 | 1,32E-60 |
| SQSTM1 | NM_001142299 | 1,77 | 1,73E-60 |
| C6orf132 | NM_001164446 | -3,06 | 2,15E-60 |
| GTSE1 | NM_016426 | -2,78 | 2,88E-60 |
| E2F1 | NM_005225 | -2,93 | 3,15E-60 |
| BIRC5 | NM_001012271 | -2,82 | 3,76E-60 |
| BCL2L11 | NM_138621 | 3,40 | 4,96E-60 |
| ABCE1 | NM_001040876 | -1,77 | 7,03E-60 |
| SKA3 | NM_145061 | -3,52 | 7,73E-60 |
| ITGB4 | NM_001005731 | 5,03 | 1,27E-59 |
| MEG3 | NR_003531 | 2,36 | 1,72E-59 |
| FSTL3 | NM_005860 | -2,38 | 2,09E-59 |
| PGK1 | NM_000291 | -2,07 | 2,49E-59 |
| KIAA1524 | NM_020890 | -2,74 | 3,12E-59 |
| NEK7 | NM_133494 | -1,77 | 3,18E-59 |
| CCT5 | NM_012073 | -1,97 | 3,30E-59 |
| TK1 | NM_003258 | -2,59 | 1,59E-58 |
| DNAJB4 | NM_007034 | -2,24 | 1,93E-58 |
| TCF19 | NM_007109 | -3,06 | 3,57E-58 |
| KITLG | NM_000899 | -1,96 | 3,57E-58 |
| FKBP4 | NM_002014 | -1,76 | 3,57E-58 |
| HNRNPAB | NM_031266 | -2,02 | 4,72E-58 |
| TSC22D1 | NM_006022 | 1,95 | 6,01E-58 |
| FN1 | NM_054034 | 1,65 | 6,53E-58 |
| FLNC | NM_001127487 | -1,89 | 1,05E-57 |
| HBP1 | NM_012257 | 2,05 | 1,05E-57 |
| SSR3 | NM_007107 | -1,48 | 1,43E-57 |
| NEDD9 | NM_006403 | -3,69 | 3,28E-57 |
| PFAS | NM_012393 | -2,15 | 3,52E-57 |
| HJURP | NM_018410 | -2,87 | 4,45E-57 |
| ZWINT | NM_032997 | -2,65 | 5,23E-57 |
| CENPF | NM_016343 | -2,03 | 6,52E-57 |
| CDCA5 | NM_080668 | -2,52 | 7,64E-57 |
| TUBA1C | NM_032704 | -2,64 | 8,52E-57 |
| RFWD3 | NM_018124 | -2,16 | 1,22E-56 |
| VEGFC | NM_005429 | -2,44 | 1,23E-56 |
| PDE5A | NM_033430 | 2,65 | 1,27E-56 |
| MCM7 | NM_182776 | -1,96 | 1,39E-56 |
| WDR1 | NM_005112 | -1,54 | 1,39E-56 |
| CA11 | NM_001217 | 4,76 | 1,71E-56 |
| GDF15 | NM_004864 | 3,13 | 1,89E-56 |
| CFH | NM_000186 | 2,01 | 2,43E-56 |
| VEGFA | NM_001025369 | 1,82 | 3,99E-56 |
| VWF | NM_000552 | -3,62 | 5,88E-56 |
| MELK | NM_014791 | -3,16 | 6,14E-56 |
| C9orf140 | NM_178448 | -2,57 | 1,14E-55 |
| ANKRD10 | NM_017664 | 2,18 | 1,88E-55 |
| SSC5D | NM_001144950 | 2,67 | 1,88E-55 |
| YWHAH | NM_003405 | -1,80 | 2,11E-55 |
| UAP1 | NM_003115 | -2,04 | 2,11E-55 |
| FLNB | NM_001164319 | -1,72 | 2,83E-55 |
| DEPDC1 | NM_001114120 | -2,74 | 3,14E-55 |
| TRO | NM_001039705 | 2,21 | 5,94E-55 |
| ATG7 | NM_001136031 | 1,99 | 6,46E-55 |
| FGF5 | NM_004464 | -2,31 | 7,08E-55 |
| PBK | NM_018492 | -3,08 | 8,32E-55 |
| CDH2 | NM_001792 | -2,19 | 8,60E-55 |
| KIF18B | NM_001080443 | -2,50 | 9,19E-55 |
| NCAPG | NM_022346 | -2,33 | 9,19E-55 |
| PTPN13 | NM_006264 | 1,89 | 1,55E-54 |
| CCNF | NM_001761 | -2,18 | 1,72E-54 |
| ASS1 | NM_000050 | 2,94 | 1,92E-54 |
| DUSP5 | NM_004419 | -1,81 | 2,76E-54 |
| MXD4 | NM_006454 | 2,09 | 4,05E-54 |
| CENPN | NM_001100624 | -2,41 | 9,69E-54 |
| CBLN3 | NM_001039771 | 3,43 | 1,25E-53 |
| NUAK2 | NM_030952 | -3,48 | 1,59E-53 |
| KIF2C | NM_006845 | -2,43 | 1,69E-53 |
| CPT1A | NM_001876 | 2,41 | 1,88E-53 |
| DUSP1 | NM_004417 | -1,78 | 1,92E-53 |
| DHCR7 | NM_001360 | -2,82 | 2,14E-53 |
| FAM110B | NM_147189 | 3,20 | 2,67E-53 |
| NEXN | NM_144573 | -2,35 | 2,72E-53 |
| TRIM22 | NM_006074 | 2,12 | 5,29E-53 |
| CDK2 | NM_052827 | -2,17 | 1,55E-52 |
| CDCA3 | NM_031299 | -2,58 | 1,64E-52 |
| ACOT7 | NM_181864 | -2,12 | 2,20E-52 |
| ACLY | NM_001096 | -1,67 | 2,25E-52 |
| GAA | NM_001079804 | 2,17 | 2,35E-52 |
| FST | NM_006350 | -3,45 | 2,79E-52 |
| AFAP1L1 | NM_001146337 | -3,15 | 3,68E-52 |
| PDGFRB | NM_002609 | 1,75 | 4,30E-52 |
| CSE1L | NM_001316 | -1,94 | 4,89E-52 |
| COL21A1 | NM_030820 | 3,49 | 4,99E-52 |
| LGALS3 | NR_003225 | 2,39 | 5,45E-52 |
| EIF5A | NM_001143761 | -1,71 | 5,69E-52 |
| FAM83D | NM_030919 | -2,56 | 8,79E-52 |
| COL16A1 | NM_001856 | 3,13 | 1,22E-51 |
| EMP1 | NM_001423 | -2,18 | 1,29E-51 |
| ACTN1 | NM_001102 | -1,79 | 1,41E-51 |
| DLGAP5 | NM_014750 | -2,37 | 2,21E-51 |
| NDRG1 | NM_001135242 | 1,59 | 2,46E-51 |
| ITM2C | NM_030926 | 1,53 | 2,83E-51 |
| SGOL2 | NM_001160033 | -2,36 | 3,17E-51 |
| SLC6A6 | NM_003043 | 2,46 | 3,28E-51 |
| FEN1 | NM_004111 | -2,58 | 5,47E-51 |
| BIRC2 | NM_001166 | -1,67 | 6,19E-51 |
| HSP90AA1 | NM_005348 | -1,90 | 6,74E-51 |
| SLC19A1 | NM_194255 | -2,31 | 7,27E-51 |
| CDCA7 | NM_031942 | -2,80 | 9,00E-51 |
| NFATC4 | NM_001136022 | 2,24 | 9,65E-51 |
| FAM64A | NM_019013 | -3,09 | 1,02E-50 |
| UBA7 | NM_003335 | 2,85 | 1,24E-50 |
| KRT80 | NM_001081492 | -3,09 | 1,57E-50 |
| MAPRE1 | NM_012325 | -1,57 | 1,75E-50 |
| CMKLR1 | NM_001142343 | 2,36 | 2,19E-50 |
| CLIP3 | NM_015526 | 3,18 | 2,58E-50 |
| ITGA11 | NM_001004439 | 2,00 | 2,59E-50 |
| ADAMTS10 | NM_030957 | 2,31 | 3,37E-50 |
| BAI2 | NM_001703 | 2,31 | 4,35E-50 |
| NBL1 | NM_182744 | 2,93 | 6,10E-50 |
| PDIA4 | NM_004911 | -1,42 | 6,59E-50 |
| CCNB2 | NM_004701 | -2,51 | 6,87E-50 |
| FOSL1 | NM_005438 | -2,91 | 7,91E-50 |
| TTLL3 | NM_001025930 | 2,60 | 8,87E-50 |
| MAFF | NM_012323 | -2,47 | 8,91E-50 |
| PBX1 | NM_002585 | 2,92 | 9,43E-50 |
| SMC4 | NM_005496 | -1,75 | 9,58E-50 |
| SOX4 | NM_003107 | 1,91 | 1,06E-49 |
| TUBB2A | NM_001069 | -2,65 | 1,15E-49 |
| FAM101B | NM_182705 | -2,23 | 1,26E-49 |
| NOLC1 | NM_004741 | -1,73 | 1,62E-49 |
| ETV1 | NM_004956 | 3,26 | 1,81E-49 |
| DNMT1 | NM_001130823 | -1,58 | 1,96E-49 |
| PAQR4 | NM_152341 | -2,12 | 3,09E-49 |
| SALL2 | NM_005407 | 2,24 | 3,64E-49 |
| SLC6A15 | NM_182767 | 3,19 | 4,33E-49 |
| MAD2L1 | NM_002358 | -2,79 | 5,24E-49 |
| IKBIP | NM_153687 | -1,94 | 5,47E-49 |
| HMGB2 | NM_001130689 | -1,96 | 1,60E-48 |
| OXTR | NM_000916 | -3,19 | 2,72E-48 |
| BRIP1 | NM_032043 | -3,12 | 2,75E-48 |
| ARHGAP29 | NM_004815 | 1,60 | 2,75E-48 |
| KLF2 | NM_016270 | -3,18 | 3,41E-48 |
| GLT8D2 | NM_031302 | 3,09 | 4,96E-48 |
| AK3L1 | NM_001005353 | -2,64 | 6,58E-48 |
| TACC3 | NM_006342 | -2,61 | 7,38E-48 |
| SNHG3-RCC1 | NM_001048199 | -1,85 | 9,69E-48 |
| RCC1 | NM_001269 | -1,86 | 9,70E-48 |
| SESN3 | NM_144665 | 4,12 | 9,87E-48 |
| PDK2 | NM_002611 | 2,83 | 1,17E-47 |
| ACTA2 | NM_001613 | -2,51 | 1,46E-47 |
| MCM6 | NM_005915 | -2,01 | 1,91E-47 |
| MMP1 | NM_001145938 | -2,82 | 2,48E-47 |
| PPP1R13L | NM_001142502 | -2,05 | 3,39E-47 |
| FANCI | NM_018193 | -1,94 | 3,51E-47 |
| STIP1 | NM_006819 | -1,60 | 5,71E-47 |
| S1PR2 | NM_004230 | 2,42 | 6,43E-47 |
| NEURL2 | NM_080749 | 4,65 | 8,90E-47 |
| ABL2 | NM_007314 | -1,89 | 9,21E-47 |
| UBE2C | NM_181802 | -2,61 | 1,28E-46 |
| PNRC1 | NM_006813 | 2,09 | 1,33E-46 |
| SLC2A6 | NM_017585 | -2,69 | 1,82E-46 |
| SQLE | NM_003129 | -2,19 | 2,14E-46 |
| TXNDC5 | NM_030810 | -1,50 | 2,40E-46 |
| LMO7 | NM_015842 | -2,30 | 3,62E-46 |
| CASC5 | NM_144508 | -2,36 | 5,03E-46 |
| APOLD1 | NM_001130415 | -2,39 | 6,66E-46 |
| CDC2 | NM_001130829 | -2,12 | 7,95E-46 |
| DNAJC9 | NM_015190 | -2,47 | 9,64E-46 |
| DDR1 | NM_013993 | 1,72 | 1,28E-45 |
| DYRK1B | NM_004714 | 2,25 | 1,47E-45 |
| TRANK1 | NM_014831 | 3,31 | 2,02E-45 |
| HTRA1 | NM_002775 | 1,87 | 2,86E-45 |
| TPM1 | NM_001018006 | -1,73 | 2,97E-45 |
| PLXNA2 | NM_025179 | -2,31 | 2,97E-45 |
| ASF1B | NM_018154 | -2,36 | 3,53E-45 |
| GRIN2A | NM_001134407 | 6,22 | 3,58E-45 |
| SRM | NM_003132 | -1,71 | 3,90E-45 |
| MAP3K12 | NM_006301 | 1,82 | 5,04E-45 |
| C5orf4 | NM_032385 | 4,39 | 6,50E-45 |
| ERCC6L | NM_017669 | -3,26 | 7,36E-45 |
| NCAPD3 | NM_015261 | -1,79 | 9,61E-45 |
| ABCA7 | NM_019112 | 3,55 | 9,69E-45 |
| SMS | NM_004595 | -1,62 | 1,02E-44 |
| CCS | NM_005125 | 2,89 | 1,36E-44 |
| LAMB3 | NM_000228 | 2,11 | 1,60E-44 |
| AOX1 | NM_001159 | -2,69 | 1,86E-44 |
| NAV3 | NM_014903 | -2,68 | 2,91E-44 |
| TUBA1A | NM_006009 | -1,93 | 3,38E-44 |
| EXO1 | NM_130398 | -3,01 | 3,64E-44 |
| SLC6A9 | NM_001024845 | 1,96 | 4,10E-44 |
| TUBB2B | NM_178012 | -2,45 | 4,34E-44 |
| SMURF2 | NM_022739 | -2,11 | 4,46E-44 |
| CCND3 | NM_001760 | -1,88 | 5,39E-44 |
| KCND1 | NM_004979 | 3,56 | 1,95E-43 |
| HN1L | NM_144570 | -1,74 | 2,91E-43 |
| PAMR1 | NM_015430 | 2,52 | 2,97E-43 |
| NME1 | NM_198175 | -2,21 | 3,94E-43 |
| EIF4G1 | NM_004953 | -1,27 | 4,35E-43 |
| TIE1 | NM_005424 | -4,05 | 4,88E-43 |
| TRIM62 | NM_018207 | 2,16 | 4,90E-43 |
| ANP32E | NM_030920 | -1,70 | 5,24E-43 |
| ECM2 | NM_001393 | 2,99 | 7,99E-43 |
| JMY | NM_152405 | 2,31 | 8,25E-43 |
| SKA1 | NM_001039535 | -2,91 | 1,07E-42 |
| TNFAIP3 | NM_006290 | -2,45 | 1,12E-42 |
| STK32B | NM_018401 | -1,97 | 1,19E-42 |
| C15orf23 | NM_001142761 | -2,56 | 1,34E-42 |
| CKAP4 | NM_006825 | -1,37 | 1,47E-42 |
| SEC31B | NM_015490 | 2,81 | 1,79E-42 |
| TXNRD1 | NM_003330 | -1,45 | 1,96E-42 |
| KPNB1 | NM_002265 | -1,35 | 1,99E-42 |
| LOC100128191 | NR_027157 | -2,34 | 2,07E-42 |
| ADA | NM_000022 | 2,13 | 2,56E-42 |
| GEM | NM_005261 | 1,85 | 2,84E-42 |
| FERMT2 | NM_001135000 | -1,41 | 4,36E-42 |
| ITGA10 | NM_003637 | 1,71 | 5,65E-42 |
| EPR1 | NR_002219 | -2,84 | 5,73E-42 |
| PECAM1 | NM_000442 | -3,98 | 6,04E-42 |
| CDC45L | NM_003504 | -3,27 | 6,69E-42 |
| GJA1 | NM_000165 | -1,44 | 7,84E-42 |
| CSGALNACT1 | NM_001130518 | 4,14 | 7,98E-42 |
| INCENP | NM_001040694 | -1,83 | 8,07E-42 |
| MN1 | NM_002430 | 2,41 | 9,16E-42 |
| FJX1 | NM_014344 | -1,69 | 1,00E-41 |
| RAD18 | NM_020165 | -2,53 | 1,40E-41 |
| PAICS | NM_006452 | -1,82 | 1,51E-41 |
| LMNB1 | NM_005573 | -2,85 | 1,58E-41 |
| ALCAM | NM_001627 | -1,45 | 2,08E-41 |
| SGMS2 | NM_001136258 | -1,90 | 2,79E-41 |
| SYNCRIP | NM_001159674 | -1,33 | 4,19E-41 |
| RANGAP1 | NM_002883 | -2,04 | 4,23E-41 |
| TNFRSF12A | NM_016639 | -2,18 | 5,35E-41 |
| IFT140 | NM_014714 | 2,08 | 5,86E-41 |
| ENO2 | NM_001975 | -2,07 | 8,80E-41 |
| ETS1 | NM_005238 | -1,85 | 9,37E-41 |
| METTL7A | NM_014033 | 4,87 | 9,43E-41 |
| UGDH | NM_003359 | -1,58 | 9,48E-41 |
| MAN1C1 | NM_020379 | 4,73 | 1,03E-40 |
| DIAPH1 | NM_005219 | -1,62 | 1,07E-40 |
| FAM20C | NM_020223 | 1,57 | 1,10E-40 |
| MYO1B | NM_001130158 | -1,51 | 1,15E-40 |
| FAM102A | NM_001035254 | 1,96 | 1,30E-40 |
| VARS | NM_006295 | -1,60 | 1,34E-40 |
| CAMK2N1 | NM_018584 | 1,89 | 1,39E-40 |
| COL14A1 | NM_021110 | 3,87 | 1,52E-40 |
| CYCS | NM_018947 | -2,01 | 2,09E-40 |
| TUBB3 | NM_006086 | -1,46 | 2,24E-40 |
| MAP1B | NM_005909 | -1,93 | 2,27E-40 |
| MTUS1 | NM_020749 | 1,93 | 2,41E-40 |
| PA2G4 | NM_006191 | -1,76 | 2,64E-40 |
| PSAT1 | NM_058179 | -1,63 | 2,76E-40 |
| GATSL3 | NM_001037666 | 2,90 | 2,91E-40 |
| KIAA0020 | NM_014878 | -2,00 | 3,18E-40 |
| IDI1 | NM_004508 | -2,32 | 3,61E-40 |
| GNPNAT1 | NM_198066 | -1,68 | 3,67E-40 |
| MTHFR | NM_005957 | 2,23 | 3,87E-40 |
| PLAUR | NM_001005376 | -1,99 | 4,42E-40 |
| OXSR1 | NM_005109 | -1,58 | 5,51E-40 |
| RHOB | NM_004040 | -1,84 | 5,55E-40 |
| CKAP2L | NM_152515 | -2,91 | 6,44E-40 |
| ABHD4 | NM_022060 | 2,64 | 6,44E-40 |
| POLA2 | NM_002689 | -2,38 | 7,38E-40 |
| CDR2L | NM_014603 | -1,70 | 8,68E-40 |
| PTPRF | NM_130440 | -1,92 | 8,88E-40 |
| HNRNPR | NM_001102398 | -1,51 | 9,91E-40 |
| MRTO4 | NM_016183 | -2,14 | 1,06E-39 |
| POLE | NM_006231 | -1,75 | 1,14E-39 |
| KIFC2 | NM_145754 | 2,15 | 1,33E-39 |
| UBE2T | NM_014176 | -3,24 | 1,71E-39 |
| NCAPD2 | NM_014865 | -1,33 | 1,73E-39 |
| TGFBR3 | NM_003243 | 2,31 | 1,83E-39 |
| CTSF | NM_003793 | 2,57 | 1,85E-39 |
| MAPK8IP3 | NM_001040439 | 1,47 | 1,89E-39 |
| EBNA1BP2 | NM_001159936 | -1,88 | 2,26E-39 |
| GPATCH4 | NM_015590 | -2,32 | 2,50E-39 |
| PGAM1 | NM_002629 | -1,58 | 2,62E-39 |
| KNTC1 | NM_014708 | -2,25 | 2,71E-39 |
| MYBL1 | NM_001080416 | -2,94 | 2,93E-39 |
| UCK2 | NM_012474 | -2,17 | 2,96E-39 |
| AP1G2 | NM_003917 | 2,46 | 3,72E-39 |
| SLC9A3 | NM_004174 | 2,68 | 4,22E-39 |
| ATP10A | NM_024490 | -2,72 | 4,25E-39 |
| KIT | NM_001093772 | -3,03 | 4,51E-39 |
| SLC1A4 | NM_001135581 | -1,88 | 4,62E-39 |
| CMBL | NM_138809 | 2,55 | 5,31E-39 |
| CNN3 | NM_001839 | -1,32 | 5,36E-39 |
| MICA | NM_000247 | -1,76 | 5,61E-39 |
| NYNRIN | NM_025081 | 2,33 | 6,21E-39 |
| AKR1C1 | NM_001353 | 4,46 | 6,22E-39 |
| BRCA2 | NM_000059 | -2,80 | 8,18E-39 |
| PLIN2 | NM_001122 | 2,20 | 8,24E-39 |
| RAD51 | NM_002875 | -3,53 | 8,43E-39 |
| HNRNPH3 | NM_021644 | -1,50 | 1,02E-38 |
| LEPREL1 | NM_001134418 | -2,48 | 1,08E-38 |
| RFC2 | NM_002914 | -2,64 | 1,20E-38 |
| ZNF367 | NM_153695 | -3,28 | 1,30E-38 |
| DNM1 | NM_001005336 | 2,85 | 1,35E-38 |
| sep-11 | NM_018243 | -1,33 | 1,42E-38 |
| FLJ10357 | NM_018071 | 1,60 | 1,43E-38 |
| CIT | NM_007174 | -2,13 | 1,75E-38 |
| BTN3A3 | NM_006994 | 2,26 | 1,76E-38 |
| PLK4 | NM_014264 | -3,17 | 2,03E-38 |
| TRA2B | NM_004593 | -1,39 | 2,10E-38 |
| IFITM3 | NM_021034 | 1,82 | 2,14E-38 |
| ASPH | NM_032468 | -1,61 | 2,17E-38 |
| UBE2N | NM_003348 | -1,61 | 2,29E-38 |
| NCLN | NM_020170 | -1,96 | 2,54E-38 |
| FANCD2 | NM_033084 | -2,99 | 2,56E-38 |
| MYC | NM_002467 | -1,63 | 2,67E-38 |
| AMD1 | NM_001634 | -1,75 | 2,69E-38 |
| GINS1 | NM_021067 | -2,47 | 3,03E-38 |
| FAM113A | NM_022760 | 1,96 | 3,56E-38 |
| IVNS1ABP | NM_006469 | -1,71 | 3,62E-38 |
| CHAF1A | NM_005483 | -2,50 | 3,79E-38 |
| GRWD1 | NM_031485 | -1,86 | 7,97E-38 |
| CDC25A | NM_001789 | -3,06 | 7,99E-38 |
| NLGN2 | NM_020795 | 1,39 | 1,06E-37 |
| ALDH1B1 | NM_000692 | -1,90 | 1,17E-37 |
| IQGAP3 | NM_178229 | -2,25 | 1,33E-37 |
| ACAT2 | NM_005891 | -2,35 | 1,48E-37 |
| BCAR3 | NM_003567 | -2,24 | 1,66E-37 |
| TNC | NM_002160 | -1,64 | 1,66E-37 |
| CAP1 | NM_001105530 | -1,53 | 1,84E-37 |
| MESDC1 | NM_022566 | -1,91 | 1,89E-37 |
| C5orf23 | NM_024563 | -2,07 | 1,95E-37 |
| CTSD | NM_001909 | 1,34 | 2,12E-37 |
| CTSO | NM_001334 | 2,19 | 2,30E-37 |
| NARG1 | NM_057175 | -1,52 | 2,58E-37 |
| CPE | NM_001873 | 1,82 | 2,68E-37 |
| IPO5 | NM_002271 | -1,21 | 2,91E-37 |
| FADS1 | NM_013402 | -1,96 | 2,93E-37 |
| NOP2 | NM_001033714 | -1,60 | 3,25E-37 |
| LRRC8C | NM_032270 | -2,27 | 3,72E-37 |
| COL6A1 | NM_001848 | 1,37 | 4,00E-37 |
| CSDC2 | NM_014460 | 3,51 | 4,48E-37 |
| NUP205 | NM_015135 | -1,92 | 4,98E-37 |
| MET | NM_000245 | -2,69 | 5,17E-37 |
| ESPL1 | NM_012291 | -2,09 | 5,22E-37 |
| KIF14 | NM_014875 | -2,14 | 6,13E-37 |
| DBF4 | NM_006716 | -2,50 | 6,14E-37 |
| TOMM40 | NM_001128917 | -2,05 | 6,14E-37 |
| NPR3 | NM_000908 | -2,21 | 6,38E-37 |
| CD59 | NM_001127223 | -1,23 | 7,37E-37 |
| FBXO5 | NM_012177 | -2,09 | 7,65E-37 |
| USP1 | NM_003368 | -1,64 | 8,31E-37 |
| MCM2 | NM_004526 | -2,12 | 8,50E-37 |
| ENPP2 | NM_006209 | 2,38 | 9,45E-37 |
| TCP11L2 | NM_152772 | 3,61 | 9,53E-37 |
| SYDE1 | NM_033025 | -1,52 | 1,05E-36 |
| RPL22L1 | NM_001099645 | -2,25 | 1,08E-36 |
| SFTA1P | NR_027082 | -2,82 | 1,37E-36 |
| C13orf33 | NM_032849 | 1,52 | 1,61E-36 |
| NTN4 | NM_021229 | -1,37 | 1,64E-36 |
| C19orf66 | NM_018381 | 2,09 | 1,67E-36 |
| BOC | NM_033254 | 2,15 | 2,13E-36 |
| FAM107B | NM_031453 | -2,17 | 2,57E-36 |
| ACTR2 | NM_001005386 | -1,17 | 2,73E-36 |
| CCT2 | NM_006431 | -1,47 | 3,58E-36 |
| SFRS3 | NM_003017 | -1,80 | 4,13E-36 |
| GDF5 | NM_000557 | 2,57 | 4,59E-36 |
| CLU | NM_001831 | 1,65 | 4,82E-36 |
| SAE1 | NM_001145713 | -1,39 | 4,85E-36 |
| PAPSS2 | NM_001015880 | -1,51 | 5,24E-36 |
| GAP43 | NM_001130064 | 4,75 | 5,41E-36 |
| C15orf42 | NM_152259 | -2,34 | 5,52E-36 |
| LGALS3BP | NM_005567 | 1,14 | 6,81E-36 |
| UBE2L6 | NM_004223 | 2,84 | 7,65E-36 |
| TROAP | NM_005480 | -1,95 | 8,05E-36 |
| FMN2 | NM_020066 | -2,26 | 8,29E-36 |
| TMEM119 | NM_181724 | 6,53 | 8,71E-36 |
| CUL7 | NM_014780 | 1,60 | 9,99E-36 |
| TSPAN9 | NM_006675 | 1,94 | 1,08E-35 |
| WBP1 | NM_012477 | 1,67 | 1,12E-35 |
| SC4MOL | NM_006745 | -2,37 | 1,33E-35 |
| GINS4 | NM_032336 | -2,56 | 1,55E-35 |
| SLC25A32 | NM_030780 | -1,67 | 1,60E-35 |
| SLC1A5 | NM_001145145 | -1,42 | 1,66E-35 |
| ITM2B | NM_021999 | 1,63 | 1,91E-35 |
| ARHGDIA | NM_004309 | -1,41 | 1,96E-35 |
| SLC22A18 | NM_002555 | 2,51 | 2,15E-35 |
| CALM1 | NM_001166106 | -1,34 | 2,20E-35 |
| MRC2 | NM_006039 | 1,43 | 2,26E-35 |
| TCOF1 | NM_001008656 | -1,40 | 2,30E-35 |
| GLT25D1 | NM_024656 | -1,24 | 2,68E-35 |
| TUBB | NM_178014 | -1,99 | 3,14E-35 |
| RBM24 | NM_001143942 | -2,66 | 3,17E-35 |
| GJC1 | NM_001080383 | -1,56 | 3,39E-35 |
| SLC39A14 | NM_001128431 | -1,41 | 3,69E-35 |
| CRTC1 | NM_001098482 | 2,17 | 3,78E-35 |
| ZNF862 | NM_001099220 | 2,25 | 3,85E-35 |
| KIF20B | NM_016195 | -2,29 | 4,54E-35 |
| TMC4 | NM_144686 | 4,13 | 5,80E-35 |
| FAM38A | NM_001142864 | -1,35 | 6,05E-35 |
| FLT1 | NM_001159920 | -4,69 | 6,34E-35 |
| MKI67 | NM_002417 | -2,37 | 6,92E-35 |
| PPIF | NM_005729 | -1,85 | 7,00E-35 |
| ENDOD1 | NM_015036 | -1,32 | 7,31E-35 |
| LOX | NM_002317 | -2,43 | 9,20E-35 |
| HSPA5 | NM_005347 | -1,53 | 1,04E-34 |
| TIMELESS | NM_003920 | -1,67 | 1,20E-34 |
| AXL | NM_001699 | -1,54 | 1,27E-34 |
| ROBO4 | NM_019055 | -3,87 | 1,37E-34 |
| PI15 | NM_015886 | 7,61 | 1,39E-34 |
| PEA15 | NM_003768 | -1,62 | 1,52E-34 |
| BBS1 | NM_024649 | 2,07 | 1,68E-34 |
| TGM2 | NM_004613 | -2,48 | 1,77E-34 |
| RAN | NM_006325 | -1,68 | 1,89E-34 |
| HMGCS1 | NM_002130 | -2,37 | 2,16E-34 |
| FREQ | NM_014286 | -1,34 | 2,24E-34 |
| NISCH | NM_007184 | 1,37 | 2,37E-34 |
| LTBP4 | NM_001042545 | 2,36 | 2,42E-34 |
| BHLHE41 | NM_030762 | 2,76 | 2,63E-34 |
| ORAI3 | NM_152288 | 2,48 | 3,91E-34 |
| DUSP7 | NM_001947 | -1,63 | 4,03E-34 |
| VDAC1 | NM_003374 | -1,23 | 4,81E-34 |
| KALRN | NM_007064 | -2,47 | 5,00E-34 |
| LSAMP | NM_002338 | 3,21 | 5,72E-34 |
| GRN | NM_002087 | 1,42 | 6,32E-34 |
| RRS1 | NM_015169 | -2,11 | 6,83E-34 |
| CLEC14A | NM_175060 | -4,67 | 7,93E-34 |
| ZWILCH | NR_003105 | -1,92 | 9,12E-34 |
| SIPA1L3 | NM_015073 | -1,77 | 9,96E-34 |
| PRR5L | NM_024841 | -2,29 | 1,32E-33 |
| SECTM1 | NM_003004 | 5,44 | 1,34E-33 |
| ORC6L | NM_014321 | -2,75 | 1,78E-33 |
| SREBF1 | NM_001005291 | -1,88 | 1,99E-33 |
| EIF4A1 | NM_001416 | -1,20 | 1,99E-33 |
| ADAMTS14 | NM_139155 | 2,66 | 2,19E-33 |
| CCNG2 | NM_004354 | 1,87 | 2,43E-33 |
| ELOVL6 | NM_024090 | -2,19 | 2,55E-33 |
| CKAP2 | NM_001098525 | -1,85 | 2,75E-33 |
| PRKAG2 | NM_024429 | -1,88 | 2,83E-33 |
| FAM49A | NM_030797 | 4,62 | 2,84E-33 |
| PDLIM5 | NM_001011513 | -1,39 | 2,94E-33 |
| DRD1 | NM_000794 | 3,76 | 3,19E-33 |
| PSMD2 | NM_002808 | -1,31 | 3,34E-33 |
| MMP19 | NM_002429 | 1,98 | 4,51E-33 |
| DEK | NM_001134709 | -1,39 | 4,51E-33 |
| GPNMB | NM_001005340 | 2,52 | 4,79E-33 |
| TEAD4 | NM_003213 | -1,57 | 5,06E-33 |
| BAZ1A | NM_013448 | -1,43 | 5,14E-33 |
| GNB1 | NM_002074 | -1,07 | 5,43E-33 |
| ARPC5 | NM_005717 | -1,67 | 5,43E-33 |
| TOP2A | NM_001067 | -1,51 | 7,65E-33 |
| KIF20A | NM_005733 | -2,15 | 7,72E-33 |
| CDT1 | NM_030928 | -1,85 | 8,16E-33 |
| MCM3 | NM_002388 | -2,27 | 8,60E-33 |
| BTN3A1 | NM_007048 | 2,19 | 9,40E-33 |
| AHRR | NM_020731 | 1,64 | 9,74E-33 |
| KCNQ5 | NM_019842 | -3,21 | 9,76E-33 |
| RND3 | NM_005168 | -1,57 | 1,02E-32 |
| CENPJ | NM_018451 | -2,37 | 1,27E-32 |
| PCDHGB7 | NM_032101 | 1,97 | 1,46E-32 |
| CD93 | NM_012072 | -2,11 | 1,49E-32 |
| CCNYL1 | NM_001142300 | -1,61 | 2,01E-32 |
| UBASH3B | NM_032873 | -1,54 | 2,09E-32 |
| MICALL1 | NM_033386 | -1,42 | 2,36E-32 |
| GSG2 | NM_031965 | -2,75 | 2,85E-32 |
| LRRFIP1 | NM_001137553 | -1,35 | 2,85E-32 |
| TTK | NM_003318 | -2,00 | 2,91E-32 |
| BNC2 | NM_017637 | 1,86 | 3,08E-32 |
| FGD4 | NM_139241 | -2,23 | 3,12E-32 |
| SELENBP1 | NM_003944 | 2,78 | 3,59E-32 |
| GPC6 | NM_005708 | 2,10 | 3,62E-32 |
| CDCA2 | NM_152562 | -2,48 | 3,84E-32 |
| BMPR1B | NM_001203 | 3,19 | 3,91E-32 |
| LAMA4 | NM_001105207 | 1,62 | 3,95E-32 |
| C5orf41 | NM_153607 | 2,86 | 4,85E-32 |
| SIPA1L2 | NM_020808 | 2,35 | 5,03E-32 |
| NUF2 | NM_145697 | -2,50 | 6,05E-32 |
| MMP2 | NM_001127891 | 1,68 | 6,39E-32 |
| TPI1 | NR_027483 | -1,54 | 7,24E-32 |
| H6PD | NM_004285 | 1,82 | 7,58E-32 |
| POM121L9P | NR_003714 | 3,30 | 8,89E-32 |
| CPT1C | NM_001136052 | 1,93 | 9,28E-32 |
| PHLDA1 | NM_007350 | 1,21 | 9,76E-32 |
| AK2 | NM_013411 | -1,28 | 1,13E-31 |
| SPEG | NM_005876 | 1,68 | 1,18E-31 |
| POLD1 | NM_002691 | -1,75 | 1,41E-31 |
| RRAS2 | NM_012250 | -1,81 | 1,46E-31 |
| IFITM1 | NM_003641 | 4,12 | 1,57E-31 |
| CYP51A1 | NM_001146152 | -1,88 | 1,59E-31 |
| STRA6 | NM_022369 | 2,49 | 1,66E-31 |
| NBR1 | NM_031858 | 1,22 | 1,85E-31 |
| STAT2 | NM_005419 | 1,95 | 1,89E-31 |
| TOPBP1 | NM_007027 | -1,55 | 2,00E-31 |
| NUP88 | NM_002532 | -1,61 | 2,09E-31 |
| FBXO44 | NM_033182 | 1,98 | 2,16E-31 |
| HSPA9 | NM_004134 | -1,12 | 2,17E-31 |
| EZH1 | NM_001991 | 1,87 | 2,54E-31 |
| CLK1 | NR_027856 | 1,57 | 2,63E-31 |
| NUDCD1 | NM_001128211 | -2,13 | 2,92E-31 |
| FLJ90757 | NR_026857 | 2,09 | 3,14E-31 |
| DDX39 | NM_005804 | -1,69 | 3,28E-31 |
| MXD3 | NM_001142935 | -1,53 | 3,36E-31 |
| DTX3 | NM_178502 | 2,29 | 3,43E-31 |
| RHBDF1 | NM_022450 | 1,35 | 3,50E-31 |
| AFAP1 | NM_198595 | -1,36 | 4,15E-31 |
| GEFT | NM_182947 | 2,60 | 4,15E-31 |
| CHAF1B | NM_005441 | -2,30 | 4,51E-31 |
| SMC2 | NM_001042550 | -1,59 | 5,16E-31 |
| DSP | NM_004415 | -1,19 | 5,38E-31 |
| FTL | NM_000146 | 1,27 | 5,97E-31 |
| PLCH2 | NM_014638 | 2,00 | 6,00E-31 |
| ING4 | NM_001127582 | 2,24 | 7,69E-31 |
| PCMTD2 | NM_001104925 | 2,06 | 7,96E-31 |
| LY6K | NM_001160354 | -2,49 | 8,57E-31 |
| FDPS | NM_001135822 | -2,01 | 8,72E-31 |
| MTHFD1 | NM_005956 | -1,64 | 9,95E-31 |
| STX1B | NM_052874 | 3,10 | 1,00E-30 |
| EMX2OS | NR_002791 | 3,05 | 1,07E-30 |
| SRF | NM_003131 | -1,55 | 1,13E-30 |
| THBS1 | NM_003246 | -2,05 | 1,14E-30 |
| HMGB1 | NM_002128 | -1,22 | 1,16E-30 |
| LYAR | NM_017816 | -2,30 | 1,22E-30 |
| AGPAT5 | NM_018361 | -1,55 | 1,53E-30 |
| PODXL | NM_001018111 | -2,09 | 1,53E-30 |
| EFEMP1 | NM_001039348 | -2,73 | 1,59E-30 |
| CDKN1A | NM_000389 | 1,28 | 1,77E-30 |
| RFTN2 | NM_144629 | 4,25 | 1,96E-30 |
| MYO1E | NM_004998 | -1,74 | 2,00E-30 |
| PDK1 | NM_002610 | -1,90 | 2,15E-30 |
| RDH11 | NM_016026 | -1,39 | 2,18E-30 |
| IMPDH1 | NM_183243 | -1,55 | 2,38E-30 |
| C11orf82 | NM_145018 | -3,10 | 2,81E-30 |
| GMNN | NM_015895 | -2,40 | 3,01E-30 |
| KIAA0182 | NM_001134473 | 1,66 | 3,33E-30 |
| POLQ | NM_199420 | -2,69 | 3,34E-30 |
| SSPN | NM_005086 | 2,07 | 3,37E-30 |
| RANBP1 | NM_002882 | -1,98 | 3,52E-30 |
| SNRPB | NM_003091 | -1,52 | 3,74E-30 |
| CKS1B | NM_001826 | -2,06 | 4,43E-30 |
| ODC1 | NM_002539 | -1,66 | 4,62E-30 |
| DLAT | NM_001931 | -1,54 | 4,68E-30 |
| WDHD1 | NM_007086 | -2,23 | 4,78E-30 |
| FAM8A1 | NM_016255 | 1,91 | 4,85E-30 |
| SPATA5 | NM_145207 | -2,34 | 5,49E-30 |
| TMEM26 | NM_178505 | 5,93 | 5,83E-30 |
| CCRN4L | NM_012118 | -2,54 | 5,85E-30 |
| PPAT | NM_002703 | -1,94 | 6,30E-30 |
| CTSA | NM_001127695 | 1,41 | 6,76E-30 |
| FAM166A | NM_001001710 | -2,55 | 6,77E-30 |
| STMN1 | NM_005563 | -1,84 | 7,15E-30 |
| KIF22 | NM_007317 | -1,96 | 7,43E-30 |
| LRIG3 | NM_001136051 | 1,95 | 7,87E-30 |
| EFNB3 | NM_001406 | 2,64 | 7,92E-30 |
| BAZ2B | NM_013450 | 1,97 | 8,45E-30 |
| CSAD | NM_015989 | 2,37 | 9,77E-30 |
| KBTBD11 | NM_014867 | 2,05 | 9,92E-30 |
| KIAA1377 | NM_020802 | 2,85 | 1,10E-29 |
| HAPLN3 | NM_178232 | -1,87 | 1,13E-29 |
| MAP2K3 | NM_002756 | -1,52 | 1,19E-29 |
| NEK2 | NM_002497 | -2,43 | 1,32E-29 |
| PTTG1 | NM_004219 | -2,35 | 1,45E-29 |
| SFRS2 | NM_003016 | -1,57 | 1,49E-29 |
| GRPR | NM_005314 | -5,54 | 1,61E-29 |
| ASPM | NM_018136 | -1,97 | 1,66E-29 |
| EPHX1 | NM_001136018 | 1,87 | 2,01E-29 |
| IDUA | NM_000203 | 2,00 | 2,05E-29 |
| GPR176 | NM_007223 | -1,33 | 2,24E-29 |
| HSPH1 | NM_006644 | -1,30 | 2,40E-29 |
| IPO4 | NM_024658 | -1,29 | 2,43E-29 |
| PDCD11 | NM_014976 | -1,46 | 2,50E-29 |
| INSIG1 | NM_005542 | -2,40 | 3,10E-29 |
| URB2 | NM_014777 | -1,96 | 3,10E-29 |
| PSD4 | NM_012455 | 2,97 | 3,22E-29 |
| SNRPA | NM_004596 | -1,56 | 3,68E-29 |
| SH3BP2 | NM_003023 | 1,32 | 3,78E-29 |
| POP7 | NM_005837 | -1,80 | 3,93E-29 |
| PTK7 | NM_152881 | 1,22 | 4,04E-29 |
| BTBD19 | NM_001136537 | 2,26 | 5,58E-29 |
| NSUN2 | NM_017755 | -1,51 | 5,86E-29 |
| KIF15 | NM_020242 | -2,34 | 5,95E-29 |
| ERO1L | NM_014584 | -1,42 | 6,31E-29 |
| GSN | NM_001127662 | 1,19 | 6,47E-29 |
| BCAM | NM_005581 | 1,83 | 6,52E-29 |
| NFKBIL2 | NM_013432 | -1,66 | 6,52E-29 |
| LOC728661 | NM_001110781 | 1,36 | 6,68E-29 |
| CDV3 | NM_017548 | -1,10 | 6,89E-29 |
| PCDH10 | NM_032961 | -2,03 | 7,07E-29 |
| EIF2S1 | NM_004094 | -1,24 | 7,43E-29 |
| ILF3 | NM_001137673 | -1,13 | 7,44E-29 |
| STARD4 | NM_139164 | -2,22 | 7,73E-29 |
| PCDHGC5 | NM_018929 | 1,24 | 8,83E-29 |
| NUMA1 | NM_006185 | 1,13 | 8,89E-29 |
| ADAM33 | NM_025220 | 2,07 | 9,52E-29 |
| SLC7A1 | NM_003045 | -1,79 | 1,06E-28 |
| SNRPD1 | NM_006938 | -1,92 | 1,07E-28 |
| PCDHGA10 | NM_018913 | 1,24 | 1,16E-28 |
| ACVRL1 | NM_000020 | -3,30 | 1,22E-28 |
| GREM1 | NM_013372 | -1,98 | 1,23E-28 |
| PTGES3 | NM_006601 | -1,16 | 1,37E-28 |
| TIMP2 | NM_003255 | 1,02 | 1,50E-28 |
| NF2 | NM_181828 | -1,17 | 1,65E-28 |
| LRP5 | NM_002335 | 1,48 | 1,72E-28 |
| PGP | NM_001042371 | -1,71 | 1,74E-28 |
| BDNF | NM_001143814 | -2,50 | 1,80E-28 |
| ITGA5 | NM_002205 | -1,21 | 1,84E-28 |
| IFIT2 | NM_001547 | 3,07 | 1,96E-28 |
| RFC3 | NM_002915 | -2,83 | 2,10E-28 |
| ARL6IP1 | NM_015161 | -1,15 | 2,22E-28 |
| KDR | NM_002253 | -3,44 | 2,33E-28 |
| LYVE1 | NM_006691 | -2,74 | 2,37E-28 |
| SIGMAR1 | NM_005866 | -1,48 | 2,50E-28 |
| KIF18A | NM_031217 | -2,21 | 2,61E-28 |
| MASTL | NM_032844 | -1,90 | 2,68E-28 |
| UCN2 | NM_033199 | 2,44 | 2,75E-28 |
| VDR | NM_001017535 | 2,23 | 3,00E-28 |
| PSAP | NM_001042465 | 1,41 | 3,02E-28 |
| ZER1 | NM_006336 | 1,36 | 3,18E-28 |
| CCT6A | NM_001762 | -1,19 | 3,24E-28 |
| RUVBL2 | NM_006666 | -1,46 | 3,50E-28 |
| PCDHGA7 | NM_018920 | 1,23 | 3,67E-28 |
| CKAP5 | NM_014756 | -1,11 | 4,20E-28 |
| GINS2 | NM_016095 | -2,98 | 4,78E-28 |
| FBXL17 | NM_001163315 | 1,77 | 5,21E-28 |
| SH3RF1 | NM_020870 | -1,26 | 5,48E-28 |
| PSD3 | NM_015310 | 1,70 | 5,72E-28 |
| MCM8 | NM_032485 | -2,10 | 6,31E-28 |
| ZIC1 | NM_003412 | 1,47 | 6,38E-28 |
| BRCA1 | NR_027676 | -2,12 | 6,53E-28 |
| PCDHGA6 | NM_018919 | 1,23 | 6,87E-28 |
| HMGCR | NM_000859 | -1,76 | 7,07E-28 |
| PCDHGA3 | NM_018916 | 1,22 | 7,15E-28 |
| TSR1 | NM_018128 | -1,25 | 7,18E-28 |
| SNX9 | NM_016224 | 1,23 | 8,06E-28 |
| CD97 | NM_078481 | -1,26 | 8,30E-28 |
| TBC1D1 | NM_015173 | -1,41 | 8,37E-28 |
| NOL6 | NM_022917 | -1,31 | 9,32E-28 |
| MIAT | NR_003491 | 4,32 | 9,57E-28 |
| NRAS | NM_002524 | -1,35 | 9,88E-28 |
| NIP7 | NM_016101 | -1,78 | 1,03E-27 |
| PCDHGA4 | NM_018917 | 1,22 | 1,05E-27 |
| GSPT1 | NM_002094 | -1,25 | 1,10E-27 |
| HNRNPH1 | NM_005520 | -1,35 | 1,18E-27 |
| VOPP1 | NM_030796 | -1,25 | 1,21E-27 |
| PLA2G6 | NM_003560 | 2,17 | 1,23E-27 |
| SLC22A17 | NM_016609 | 1,72 | 1,25E-27 |
| NDC80 | NM_006101 | -2,17 | 1,26E-27 |
| GPRC5A | NM_003979 | -2,18 | 1,28E-27 |
| ORC1L | NM_004153 | -2,75 | 1,31E-27 |
| FOXC1 | NM_001453 | 1,76 | 1,31E-27 |
| SLC27A1 | NM_198580 | 1,60 | 1,32E-27 |
| VLDLR | NM_003383 | -2,44 | 1,40E-27 |
| PCDHGA9 | NM_018921 | 1,22 | 1,43E-27 |
| FOXO4 | NM_005938 | 2,88 | 1,46E-27 |
| PCDHGC3 | NM_032403 | 1,22 | 1,50E-27 |
| AKR1C3 | NM_003739 | 4,08 | 1,72E-27 |
| PCDHGB3 | NM_018924 | 1,22 | 1,80E-27 |
| EXOSC9 | NM_001034194 | -1,95 | 1,82E-27 |
| CCNL2 | NM_001039577 | 1,18 | 1,87E-27 |
| ITGA4 | NM_000885 | -1,64 | 2,09E-27 |
| PCDHGA2 | NM_018915 | 1,22 | 2,15E-27 |
| PCDHGB2 | NM_018923 | 1,22 | 2,22E-27 |
| PCDHGB6 | NM_018926 | 1,23 | 2,28E-27 |
| PCDHGB1 | NM_018922 | 1,22 | 2,33E-27 |
| LRP10 | NM_014045 | 1,18 | 2,48E-27 |
| ITPKB | NM_002221 | 1,90 | 2,52E-27 |
| PCDHGB4 | NM_003736 | 1,19 | 2,73E-27 |
| LXN | NM_020169 | 2,10 | 2,84E-27 |
| GTPBP4 | NM_012341 | -1,90 | 3,26E-27 |
| PCDHGA5 | NM_018918 | 1,22 | 3,35E-27 |
| PCDHGC4 | NM_018928 | 1,22 | 3,38E-27 |
| PCDHGA1 | NM_018912 | 1,22 | 3,48E-27 |
| HMMR | NM_001142557 | -2,33 | 3,56E-27 |
| PTGDS | NM_000954 | 3,70 | 3,58E-27 |
| PRPS1 | NM_002764 | -1,94 | 3,59E-27 |
| GRPEL1 | NM_025196 | -1,85 | 3,63E-27 |
| PCOLCE | NM_002593 | 1,38 | 3,75E-27 |
| TINAGL1 | NM_022164 | -2,25 | 4,02E-27 |
| KIAA1671 | NM_001145206 | 1,75 | 4,41E-27 |
| APOBEC3B | NM_004900 | -2,33 | 4,55E-27 |
| LRP8 | NM_001018054 | -2,20 | 4,71E-27 |
| NUP50 | NM_153645 | -1,34 | 4,92E-27 |
| PRELID1 | NM_013237 | -1,52 | 5,31E-27 |
| PCDHGA8 | NM_032088 | 1,22 | 6,00E-27 |
| C11orf87 | NM_207645 | 3,18 | 6,03E-27 |
| NUP188 | NM_015354 | -1,52 | 6,23E-27 |
| C10orf119 | NM_024834 | -1,33 | 7,08E-27 |
| MAK16 | NM_032509 | -1,52 | 7,43E-27 |
| NR2F2 | NM_001145157 | 1,08 | 7,53E-27 |
| LPHN1 | NM_014921 | 1,58 | 7,79E-27 |
| CENPK | NM_022145 | -2,77 | 8,08E-27 |
| FAM38B | NM_022068 | 1,58 | 8,32E-27 |
| MST1 | NM_020998 | 3,95 | 9,06E-27 |
| E2F7 | NM_203394 | -1,49 | 9,10E-27 |
| GPI | NM_000175 | -1,09 | 9,65E-27 |
| PCBP3 | NM_001130141 | 2,83 | 1,02E-26 |
| VRK1 | NM_003384 | -2,77 | 1,23E-26 |
| WSB2 | NM_018639 | -1,26 | 1,24E-26 |
| ERMAP | NM_001017922 | 2,17 | 1,24E-26 |
| LMOD1 | NM_012134 | 2,35 | 1,24E-26 |
| PFKFB3 | NM_004566 | -1,92 | 1,25E-26 |
| STIL | NM_001048166 | -2,42 | 1,33E-26 |
| KIAA1370 | NM_019600 | 2,45 | 1,36E-26 |
| KIAA1755 | NM_001029864 | 1,98 | 1,38E-26 |
| PCDHGA11 | NM_018914 | 1,17 | 1,51E-26 |
| C4orf46 | NM_001008393 | -1,89 | 1,53E-26 |
| SLC7A8 | NM_012244 | 5,03 | 1,61E-26 |
| ZHX2 | NM_014943 | 1,83 | 1,90E-26 |
| ANK3 | NM_020987 | 2,65 | 2,06E-26 |
| SELI | NM_033505 | -1,65 | 2,22E-26 |
| UNC5B | NM_170744 | 2,18 | 2,23E-26 |
| ACACA | NM_198836 | -1,63 | 2,26E-26 |
| SETBP1 | NM_015559 | 1,83 | 2,31E-26 |
| PPRC1 | NM_015062 | -1,28 | 2,39E-26 |
| ATP6V1C2 | NM_001039362 | -1,33 | 2,40E-26 |
| SOX9 | NM_000346 | 1,83 | 2,41E-26 |
| DAZAP1 | NM_018959 | -1,20 | 2,44E-26 |
| RNF144B | NM_182757 | -3,12 | 2,44E-26 |
| CALHM2 | NM_015916 | 1,48 | 2,53E-26 |
| IL11 | NM_000641 | -1,66 | 2,55E-26 |
| EPHA3 | NM_005233 | 4,72 | 2,63E-26 |
| DCTPP1 | NM_024096 | -1,88 | 3,06E-26 |
| SDK1 | NM_152744 | 1,98 | 3,11E-26 |
| ELL2 | NM_012081 | -1,61 | 3,17E-26 |
| GABBR1 | NM_001470 | 1,72 | 3,20E-26 |
| FSCN1 | NM_003088 | -1,06 | 3,22E-26 |
| TMEM8B | NM_001042589 | 2,23 | 3,27E-26 |
| IGFBP4 | NM_001552 | 1,80 | 3,34E-26 |
| DFNB31 | NM_015404 | 2,16 | 3,39E-26 |
| PPM1H | NM_020700 | 2,32 | 3,54E-26 |
| MXI1 | NM_005962 | 1,95 | 3,59E-26 |
| GNB4 | NM_021629 | -1,15 | 4,13E-26 |
| DUSP2 | NM_004418 | 3,50 | 4,92E-26 |
| MCC | NM_002387 | 2,14 | 5,28E-26 |
| MAML3 | NM_018717 | 1,80 | 5,48E-26 |
| THOC4 | NM_005782 | -2,01 | 5,74E-26 |
| KPNA4 | NM_002268 | -1,17 | 5,78E-26 |
| ATAD3A | NM_018188 | -1,79 | 6,01E-26 |
| CD44 | NM_001001392 | -1,12 | 6,16E-26 |
| HAS2 | NM_005328 | -1,92 | 6,57E-26 |
| SPON2 | NM_001128325 | -1,77 | 6,76E-26 |
| AMT | NM_000481 | 3,21 | 7,43E-26 |
| MZF1 | NM_198055 | 1,79 | 7,65E-26 |
| NRXN3 | NM_001105250 | -2,63 | 7,66E-26 |
| PSMD14 | NM_005805 | -1,72 | 7,67E-26 |
| LDB2 | NM_001290 | 2,03 | 7,74E-26 |
| NEO1 | NM_002499 | 1,95 | 8,48E-26 |
| SPARC | NM_003118 | 1,00 | 8,59E-26 |
| MAP2K1 | NM_002755 | -1,42 | 8,80E-26 |
| POP1 | NM_001145860 | -2,22 | 9,51E-26 |
| KSR1 | NM_014238 | 1,86 | 1,07E-25 |
| MLLT11 | NM_006818 | -1,32 | 1,14E-25 |
| MLF1IP | NM_024629 | -2,16 | 1,16E-25 |
| WISP2 | NM_003881 | 5,23 | 1,18E-25 |
| CFL1 | NM_005507 | -1,28 | 1,18E-25 |
| HSF4 | NM_001040667 | 3,32 | 1,33E-25 |
| ARHGDIB | NM_001175 | -2,61 | 1,46E-25 |
| APBB3 | NM_133172 | 1,76 | 1,49E-25 |
| CYBRD1 | NM_001127383 | 1,60 | 1,53E-25 |
| DKC1 | NM_001142463 | -1,71 | 1,56E-25 |
| BRIX1 | NM_018321 | -1,75 | 1,83E-25 |
| SPTLC3 | NM_018327 | 1,74 | 1,90E-25 |
| MYBBP1A | NM_014520 | -1,39 | 1,93E-25 |
| STK17A | NM_004760 | -1,61 | 1,95E-25 |
| SUV39H1 | NM_003173 | -1,96 | 1,96E-25 |
| ATP2A2 | NM_001681 | -1,08 | 1,99E-25 |
| CHST3 | NM_004273 | -1,22 | 2,02E-25 |
| ATP6V1G2 | NM_138282 | 2,82 | 2,10E-25 |
| YWHAG | NM_012479 | -1,35 | 2,13E-25 |
| ZBED4 | NM_014838 | -1,30 | 2,13E-25 |
| JMJD6 | NM_001081461 | -1,89 | 2,26E-25 |
| CDCA4 | NM_017955 | -1,69 | 2,53E-25 |
| SHISA2 | NM_001007538 | 1,30 | 2,64E-25 |
| OXR1 | NM_018002 | 1,29 | 2,92E-25 |
| RNF182 | NM_001165032 | -2,07 | 3,04E-25 |
| GRB10 | NM_001001550 | -1,12 | 3,65E-25 |
| NUPL1 | NM_014089 | -1,69 | 3,76E-25 |
| GBX2 | NM_001485 | -4,04 | 3,89E-25 |
| WDR4 | NM_018669 | -2,34 | 3,90E-25 |
| PCDHGB5 | NM_018925 | 1,17 | 4,49E-25 |
| GYS1 | NM_002103 | -1,67 | 4,87E-25 |
| ZBTB46 | NM_025224 | 2,30 | 4,89E-25 |
| CCDC3 | NM_031455 | -1,89 | 5,24E-25 |
| PDCD1LG2 | NM_025239 | -3,01 | 5,34E-25 |
| YPEL1 | NM_013313 | 1,77 | 5,77E-25 |
| CTDSP2 | NM_005730 | 1,08 | 6,32E-25 |
| FLCN | NM_144997 | 1,64 | 6,40E-25 |
| RHOC | NM_175744 | -1,07 | 6,57E-25 |
| LAMA5 | NM_005560 | 1,17 | 6,65E-25 |
| FAM171B | NM_177454 | 1,94 | 7,70E-25 |
| PPP1R1B | NM_032192 | 6,35 | 7,82E-25 |
| TFDP1 | NM_007111 | -1,34 | 8,33E-25 |
| HCN2 | NM_001194 | 1,83 | 9,03E-25 |
| POLR2D | NM_004805 | -1,40 | 9,06E-25 |
| TAGLN2 | NM_003564 | -1,00 | 9,27E-25 |
| INSR | NM_000208 | 1,71 | 9,60E-25 |
| C1RL | NM_016546 | 2,53 | 1,01E-24 |
| CENPI | NM_006733 | -3,35 | 1,04E-24 |
| CHTF18 | NM_022092 | -1,54 | 1,04E-24 |
| C16orf7 | NM_004913 | 1,67 | 1,04E-24 |
| RBM43 | NM_198557 | 2,72 | 1,07E-24 |
| MTP18 | NM_016498 | -2,17 | 1,13E-24 |
| PDIA6 | NM_005742 | -1,31 | 1,16E-24 |
| ALDH3A2 | NM_001031806 | 1,31 | 1,16E-24 |
| TRPS1 | NM_014112 | 1,62 | 1,20E-24 |
| HNRNPD | NM_031369 | -1,24 | 1,32E-24 |
| ARPC2 | NM_005731 | -1,14 | 1,34E-24 |
| FAM111B | NM_001142703 | -2,44 | 1,34E-24 |
| GLO1 | NM_006708 | -1,17 | 1,36E-24 |
| CYB5B | NM_030579 | -1,28 | 1,45E-24 |
| GLIS1 | NM_147193 | 1,97 | 1,45E-24 |
| RRP15 | NM_016052 | -1,81 | 1,48E-24 |
| WDTC1 | NM_015023 | 1,32 | 1,56E-24 |
| CDK6 | NM_001259 | -1,22 | 1,76E-24 |
| HMOX1 | NM_002133 | 2,09 | 1,78E-24 |
| IL34 | NM_152456 | 5,17 | 1,93E-24 |
| LBR | NM_002296 | -1,52 | 1,96E-24 |
| RRP12 | NM_015179 | -1,46 | 1,96E-24 |
| SFRS18 | NM_015491 | 1,28 | 2,06E-24 |
| COL1A2 | NM_000089 | 1,73 | 2,20E-24 |
| RELT | NM_152222 | -2,19 | 2,20E-24 |
| WDR43 | NM_015131 | -1,35 | 2,46E-24 |
| TMEM2 | NM_013390 | -1,50 | 2,48E-24 |
| ADAMTS13 | NM_139025 | 3,28 | 2,69E-24 |
| EHD1 | NM_006795 | -1,34 | 2,82E-24 |
| C11orf41 | NM_012194 | -1,71 | 2,84E-24 |
| CDKN3 | NM_005192 | -2,25 | 2,98E-24 |
| BCAS3 | NM_017679 | 1,90 | 3,05E-24 |
| MICB | NM_005931 | -1,75 | 3,21E-24 |
| SLC1A1 | NM_004170 | -1,59 | 3,26E-24 |
| KIFC3 | NM_001130099 | -1,32 | 3,32E-24 |
| PCYOX1 | NM_016297 | 1,10 | 3,35E-24 |
| PRDX1 | NM_181697 | -1,16 | 3,38E-24 |
| MANF | NM_006010 | -1,81 | 3,62E-24 |
| TIAM1 | NM_003253 | 1,73 | 3,66E-24 |
| KLHL4 | NM_019117 | 2,54 | 3,86E-24 |
| SPSB3 | NM_080861 | 1,66 | 3,87E-24 |
| SFRS1 | NM_006924 | -1,21 | 3,99E-24 |
| SLC9A5 | NM_004594 | 1,91 | 4,02E-24 |
| C12orf53 | NM_153685 | 2,43 | 4,15E-24 |
| NOP56 | NR_027700 | -1,46 | 4,18E-24 |
| NTN1 | NM_004822 | 2,59 | 4,18E-24 |
| POLR1A | NM_015425 | -1,40 | 4,30E-24 |
| HMGB3 | NM_005342 | -1,78 | 4,49E-24 |
| MMP14 | NM_004995 | 1,24 | 4,49E-24 |
| SOD3 | NM_003102 | 1,94 | 5,36E-24 |
| IL8 | NM_000584 | -4,09 | 5,49E-24 |
| OBSL1 | NM_015311 | 1,79 | 6,18E-24 |
| PRR11 | NM_018304 | -2,23 | 6,24E-24 |
| RUVBL1 | NM_003707 | -1,46 | 6,78E-24 |
| RECQL4 | NM_004260 | -1,61 | 6,96E-24 |
| PLD3 | NM_001031696 | 1,32 | 7,27E-24 |
| XBP1 | NM_005080 | -1,04 | 7,31E-24 |
| FAM131B | NM_001031690 | 1,85 | 7,66E-24 |
| PLEKHA4 | NM_020904 | 2,45 | 7,68E-24 |
| MGP | NM_000900 | 2,04 | 7,71E-24 |
| SFPQ | NM_005066 | -1,19 | 8,14E-24 |
| GOLGB1 | NM_004487 | 1,24 | 8,18E-24 |
| SLC9A3R1 | NM_004252 | 1,43 | 8,38E-24 |
| CCDC137 | NM_199287 | -1,73 | 8,63E-24 |
| XRCC2 | NM_005431 | -2,41 | 8,65E-24 |
| C10orf26 | NM_017787 | 1,25 | 8,81E-24 |
| PIK3R2 | NM_005027 | 1,27 | 8,98E-24 |
| ENC1 | NM_003633 | -1,57 | 9,00E-24 |
| MTERFD2 | NR_028051 | 2,08 | 9,58E-24 |
| SCHIP1 | NM_014575 | -1,78 | 9,78E-24 |
| CACYBP | NM_001007214 | -1,59 | 1,05E-23 |
| NPTXR | NM_014293 | 1,86 | 1,08E-23 |
| MFSD2A | NM_001136493 | -4,31 | 1,10E-23 |
| HAUS6 | NM_017645 | -1,67 | 1,16E-23 |
| FH | NM_000143 | -1,26 | 1,28E-23 |
| ESCO2 | NM_001017420 | -2,88 | 1,41E-23 |
| MAP2 | NM_002374 | -2,06 | 1,49E-23 |
| TTC3 | NM_003316 | 1,08 | 1,50E-23 |
| BEX4 | NM_001080425 | 1,81 | 1,52E-23 |
| SPTLC2 | NM_004863 | -1,35 | 1,61E-23 |
| SKA2 | NM_182620 | -1,31 | 1,62E-23 |
| ARF6 | NM_001663 | -1,17 | 1,66E-23 |
| SBDS | NM_016038 | -1,31 | 1,67E-23 |
| CDKN2B | NM_004936 | 1,78 | 1,68E-23 |
| SASH1 | NM_015278 | -1,07 | 1,68E-23 |
| CD248 | NM_020404 | 1,11 | 1,70E-23 |
| CYB5D2 | NR_023347 | 2,02 | 1,71E-23 |
| POLE3 | NR_027261 | -1,29 | 1,73E-23 |
| ARSA | NM_001085427 | 1,60 | 1,75E-23 |
| ARHGEF3 | NM_019555 | 2,85 | 1,77E-23 |
| PELI2 | NM_021255 | 1,72 | 1,86E-23 |
| PCDHGA12 | NM_003735 | 1,07 | 1,90E-23 |
| NEDD1 | NM_001135176 | -1,56 | 1,90E-23 |
| PCDH1 | NM_032420 | -2,50 | 1,97E-23 |
| ACSL4 | NM_022977 | -1,14 | 2,00E-23 |
| TUBG1 | NM_001070 | -1,57 | 2,08E-23 |
| NRP1 | NM_003873 | 1,19 | 2,08E-23 |
| LAMB1 | NM_002291 | 1,09 | 2,11E-23 |
| EDNRA | NR_028596 | 1,51 | 2,18E-23 |
| CNN1 | NM_001299 | -2,17 | 2,25E-23 |
| SEMA3D | NM_152754 | 2,96 | 2,35E-23 |
| LOC651250 | NR_027418 | 1,25 | 2,42E-23 |
| XRCC5 | NM_021141 | -0,97 | 2,43E-23 |
| C16orf88 | NM_001012991 | -1,60 | 2,52E-23 |
| FRMD6 | NM_001042481 | -1,08 | 2,52E-23 |
| TMEM97 | NM_014573 | -1,60 | 2,53E-23 |
| USP14 | NM_005151 | -1,15 | 2,66E-23 |
| UTP20 | NM_014503 | -1,62 | 2,74E-23 |
| NSDHL | NM_001129765 | -1,94 | 2,80E-23 |
| GBE1 | NM_000158 | -1,15 | 2,91E-23 |
| SAMD4A | NM_001161577 | -1,60 | 2,92E-23 |
| TCEA2 | NM_198723 | 2,20 | 3,01E-23 |
| SMC1A | NM_006306 | -1,22 | 3,25E-23 |
| ZNF521 | NM_015461 | 1,68 | 3,42E-23 |
| IP6K2 | NR_027438 | 1,73 | 3,60E-23 |
| MMRN2 | NM_024756 | -2,95 | 3,64E-23 |
| N4BP2L2 | NM_033111 | 1,96 | 3,64E-23 |
| HAT1 | NM_003642 | -1,46 | 4,11E-23 |
| PPP2CB | NM_001009552 | -1,12 | 4,11E-23 |
| SMAD7 | NM_005904 | 1,25 | 4,13E-23 |
| ARAP1 | NM_015242 | 1,17 | 4,30E-23 |
| ICAM2 | NM_001099789 | -3,83 | 4,52E-23 |
| TSC2 | NM_001077183 | 1,04 | 4,77E-23 |
| KIAA0895L | NM_001040715 | 1,59 | 5,25E-23 |
| SLC8A1 | NM_001112802 | -1,49 | 6,76E-23 |
| AHSA2 | NM_152392 | 1,86 | 7,69E-23 |
| SPNS2 | NM_001124758 | -1,53 | 7,75E-23 |
| PPP2CA | NM_002715 | -1,00 | 7,75E-23 |
| HYOU1 | NM_001130991 | -1,34 | 7,79E-23 |
| DNAJB11 | NM_016306 | -1,36 | 7,89E-23 |
| FANCA | NM_000135 | -1,42 | 8,20E-23 |
| PNO1 | NM_020143 | -2,03 | 9,03E-23 |
| GABARAPL1 | NM_031412 | 1,94 | 9,41E-23 |
| PSMC3IP | NM_016556 | -1,96 | 9,89E-23 |
| IFRD2 | NM_006764 | -1,77 | 1,06E-22 |
| FIGNL1 | NM_001042762 | -1,96 | 1,07E-22 |
| COL6A2 | NM_001849 | 1,10 | 1,08E-22 |
| C7orf41 | NM_152793 | 1,71 | 1,09E-22 |
| ACCS | NM_032592 | 2,63 | 1,10E-22 |
| JUN | NM_002228 | 1,12 | 1,14E-22 |
| PRRT1 | NM_030651 | 3,18 | 1,57E-22 |
| RASSF1 | NM_170713 | -1,76 | 1,62E-22 |
| OGT | NM_181673 | 1,12 | 1,74E-22 |
| PARP1 | NM_001618 | -1,15 | 1,86E-22 |
| CENPM | NM_024053 | -2,43 | 1,87E-22 |
| BYSL | NM_004053 | -1,89 | 1,94E-22 |
| KCTD20 | NM_173562 | -1,19 | 2,00E-22 |
| GPR177 | NM_001002292 | 1,04 | 2,33E-22 |
| CRABP2 | NM_001878 | 2,67 | 2,48E-22 |
| PLCE1 | NM_016341 | 1,61 | 2,51E-22 |
| BICD2 | NM_001003800 | -1,19 | 2,60E-22 |
| CFL2 | NM_138638 | -1,09 | 2,71E-22 |
| EZH2 | NM_152998 | -1,71 | 2,73E-22 |
| PTPLA | NM_014241 | -2,77 | 2,98E-22 |
| GATAD2A | NM_017660 | -1,08 | 2,99E-22 |
| HEXA | NM_000520 | 1,17 | 3,19E-22 |
| DHX37 | NM_032656 | -1,65 | 3,44E-22 |
| IMP4 | NM_033416 | -1,40 | 3,45E-22 |
| LAMA2 | NM_000426 | 2,07 | 3,64E-22 |
| OGFOD1 | NM_018233 | -1,38 | 3,76E-22 |
| WDR76 | NM_024908 | -2,08 | 3,89E-22 |
| PTX3 | NM_002852 | -2,01 | 3,90E-22 |
| SBDSP | NR_024110 | -1,48 | 4,59E-22 |
| PLEKHB2 | NM_001100623 | -1,01 | 4,68E-22 |
| CALM2 | NM_001743 | -1,31 | 4,76E-22 |
| BUB3 | NM_004725 | -1,13 | 4,81E-22 |
| FARSA | NM_004461 | -1,26 | 5,02E-22 |
| TNK2 | NM_001010938 | 1,23 | 5,15E-22 |
| RRP1B | NM_015056 | -1,18 | 5,39E-22 |
| DKK1 | NM_012242 | -4,06 | 5,47E-22 |
| PC | NM_000920 | 1,52 | 5,66E-22 |
| GNL2 | NM_013285 | -1,33 | 5,77E-22 |
| ERRFI1 | NM_018948 | 1,24 | 5,79E-22 |
| CHML | NM_001821 | -1,29 | 6,05E-22 |
| RXRA | NM_002957 | 1,09 | 6,15E-22 |
| KATNAL1 | NM_032116 | -1,62 | 6,35E-22 |
| C14orf80 | NM_001134875 | -2,62 | 6,40E-22 |
| BAG2 | NM_004282 | -1,53 | 6,55E-22 |
| HSPE1 | NM_002157 | -1,83 | 6,83E-22 |
| ABCA2 | NM_001606 | 1,14 | 6,93E-22 |
| ZFYVE1 | NM_021260 | 1,38 | 8,44E-22 |
| FUS | NM_004960 | -1,29 | 8,53E-22 |
| GOLGA8B | NR_027410 | 1,14 | 8,92E-22 |
| GSTA4 | NM_001512 | 1,84 | 1,04E-21 |
| PSME3 | NM_005789 | -1,21 | 1,05E-21 |
| XPO6 | NM_015171 | -0,96 | 1,06E-21 |
| SLC44A1 | NM_080546 | 1,48 | 1,08E-21 |
| OLFM2 | NM_058164 | 1,70 | 1,08E-21 |
| BTG3 | NM_001130914 | -1,51 | 1,19E-21 |
| PDGFB | NM_002608 | -3,30 | 1,19E-21 |
| DHX15 | NM_001358 | -1,11 | 1,19E-21 |
| C1QTNF6 | NM_031910 | 1,63 | 1,22E-21 |
| HSP90B1 | NM_003299 | -1,14 | 1,23E-21 |
| WNT5B | NM_032642 | -1,76 | 1,36E-21 |
| PIGZ | NM_025163 | 2,79 | 1,39E-21 |
| CHST2 | NM_004267 | 1,62 | 1,41E-21 |
| RBL1 | NM_183404 | -2,16 | 1,46E-21 |
| KIAA0754 | NM_015038 | -1,63 | 1,46E-21 |
| PGAM4 | NM_001029891 | -1,52 | 1,49E-21 |
| NOP58 | NM_015934 | -1,34 | 1,53E-21 |
| DNAJC4 | NM_005528 | 1,57 | 1,55E-21 |
| PTPN11 | NM_002834 | -0,97 | 1,57E-21 |
| FTH1 | NM_002032 | 1,37 | 1,69E-21 |
| PLCB3 | NM_000932 | -1,44 | 1,71E-21 |
| MTHFD2 | NM_006636 | -1,00 | 1,72E-21 |
| FARSB | NM_005687 | -1,90 | 1,81E-21 |
| AKAP12 | NM_005100 | -1,13 | 2,00E-21 |
| EFCAB4A | NM_173584 | 2,36 | 2,21E-21 |
| CDC42EP3 | NM_006449 | -1,49 | 2,30E-21 |
| POLM | NM_013284 | 1,52 | 2,37E-21 |
| ESAM | NM_138961 | -3,04 | 2,40E-21 |
| COL4A2 | NM_001846 | 0,94 | 2,40E-21 |
| PUS7 | NM_019042 | -1,52 | 2,40E-21 |
| PAG1 | NM_018440 | -1,67 | 2,62E-21 |
| HS1BP3 | NM_022460 | 1,74 | 2,84E-21 |
| MGLL | NM_007283 | -1,36 | 2,86E-21 |
| RASL12 | NM_016563 | 3,97 | 2,97E-21 |
| VAMP1 | NM_199245 | 1,91 | 2,99E-21 |
| HPS1 | NM_000195 | 1,10 | 3,04E-21 |
| PCSK4 | NM_017573 | 3,22 | 3,28E-21 |
| ITGA3 | NM_005501 | -1,03 | 3,31E-21 |
| PCNA | NM_182649 | -1,56 | 3,53E-21 |
| IL6 | NM_000600 | -4,19 | 3,54E-21 |
| EIF4E | NM_001968 | -1,45 | 3,78E-21 |
| CCDC81 | NM_021827 | -3,20 | 4,06E-21 |
| ERLIN1 | NM_006459 | -1,18 | 4,26E-21 |
| FLVCR2 | NM_017791 | 3,62 | 4,29E-21 |
| MYO15B | NR_003587 | 4,72 | 4,31E-21 |
| NUSAP1 | NM_016359 | -2,06 | 4,32E-21 |
| NQO1 | NM_001025434 | 1,11 | 4,41E-21 |
| CAPS | NM_004058 | 2,47 | 4,57E-21 |
| SUPT16H | NM_007192 | -1,05 | 4,67E-21 |
| FAT4 | NM_024582 | 1,89 | 5,07E-21 |
| CLPTM1L | NM_030782 | -1,09 | 5,12E-21 |
| IQSEC2 | NM_001111125 | 2,09 | 5,17E-21 |
| AKR1B10 | NM_020299 | 3,38 | 5,35E-21 |
| C1QBP | NM_001212 | -1,39 | 5,86E-21 |
| GADD45B | NM_015675 | -1,45 | 6,12E-21 |
| SLC25A22 | NM_024698 | -1,19 | 6,14E-21 |
| EIF3J | NM_003758 | -1,13 | 6,15E-21 |
| VGLL3 | NM_016206 | -1,60 | 6,21E-21 |
| WDR51A | NM_001161581 | -2,09 | 6,66E-21 |
| SGSM2 | NM_014853 | 1,09 | 8,19E-21 |
| AMIGO2 | NM_001143668 | -1,32 | 8,20E-21 |
| TOP3A | NM_004618 | -1,34 | 8,71E-21 |
| CCPG1 | NM_004748 | 1,08 | 8,98E-21 |
| TRIM59 | NM_173084 | -1,56 | 9,15E-21 |
| DDX11 | NM_152438 | -1,50 | 9,69E-21 |
| ZMAT3 | NM_152240 | 1,81 | 9,76E-21 |
| NEK9 | NM_033116 | 1,15 | 1,00E-20 |
| TCERG1 | NM_001040006 | -1,15 | 1,03E-20 |
| RAD54L | NM_003579 | -1,84 | 1,05E-20 |
| PPIL1 | NM_016059 | -1,70 | 1,07E-20 |
| RNF126 | NM_194460 | -1,24 | 1,09E-20 |
| NUDT15 | NM_018283 | -1,65 | 1,14E-20 |
| EEF1E1 | NM_004280 | -1,86 | 1,15E-20 |
| PHF21A | NM_016621 | 1,64 | 1,16E-20 |
| EIF5B | NM_015904 | -0,98 | 1,18E-20 |
| LUM | NM_002345 | 2,45 | 1,33E-20 |
| CYTH3 | NM_004227 | -0,99 | 1,37E-20 |
| SLC48A1 | NM_017842 | 1,79 | 1,39E-20 |
| SPC25 | NM_020675 | -2,82 | 1,41E-20 |
| G3BP1 | NM_198395 | -1,26 | 1,46E-20 |
| OBFC1 | NM_024928 | 1,67 | 1,48E-20 |
| CHSY3 | NM_175856 | -1,73 | 1,49E-20 |
| PLK1S1 | NM_001163023 | 2,39 | 1,66E-20 |
| FAM98A | NM_015475 | -1,16 | 1,73E-20 |
| LMBRD1 | NM_018368 | 1,63 | 1,80E-20 |
| LOC728402 | NR_027338 | -1,42 | 1,84E-20 |
| ABHD14B | NM_001146314 | 1,35 | 1,91E-20 |
| MYADM | NM_001020818 | -1,44 | 1,92E-20 |
| ENOPH1 | NM_021204 | -1,29 | 1,97E-20 |
| PTPRJ | NM_002843 | -1,67 | 2,02E-20 |
| FAM46B | NM_052943 | -2,71 | 2,25E-20 |
| BLM | NM_000057 | -2,05 | 2,28E-20 |
| VASH1 | NM_014909 | 1,91 | 2,28E-20 |
| ABTB1 | NM_172027 | 1,70 | 2,33E-20 |
| NLN | NM_020726 | -1,52 | 2,38E-20 |
| MPP4 | NM_033066 | -2,72 | 2,39E-20 |
| SPSB1 | NM_025106 | 1,57 | 2,52E-20 |
| KCNAB2 | NM_172130 | 3,53 | 2,55E-20 |
| TBRG4 | NM_004749 | -1,35 | 2,57E-20 |
| ECH1 | NM_001398 | 1,63 | 2,62E-20 |
| RFC4 | NM_002916 | -2,04 | 2,62E-20 |
| PIK3IP1 | NM_052880 | 3,59 | 2,78E-20 |
| BTG1 | NM_001731 | 1,29 | 2,89E-20 |
| POLL | NM_013274 | 1,48 | 2,93E-20 |
| CDH5 | NM_001795 | -4,06 | 3,01E-20 |
| THBS3 | NM_007112 | 1,35 | 3,17E-20 |
| JUNB | NM_002229 | 1,32 | 3,18E-20 |
| LTBP1 | NM_001166264 | 0,93 | 3,22E-20 |
| STAB1 | NM_015136 | -1,96 | 3,33E-20 |
| MTMR11 | NM_001145862 | 1,59 | 3,36E-20 |
| ULK1 | NM_003565 | 1,00 | 3,54E-20 |
| FAR1 | NM_032228 | -1,14 | 3,54E-20 |
| NUP107 | NM_020401 | -1,47 | 3,55E-20 |
| XRCC6 | NM_001469 | -0,96 | 3,64E-20 |
| LTBP3 | NM_001130144 | 1,44 | 3,64E-20 |
| PARP10 | NM_032789 | 1,69 | 3,85E-20 |
| ICMT | NM_012405 | -1,10 | 3,95E-20 |
| LOC91316 | NR_024448 | 1,98 | 4,34E-20 |
| FAM176A | NM_032181 | -1,78 | 4,43E-20 |
| C17orf72 | NM_001164257 | -3,37 | 4,62E-20 |
| DDX18 | NM_006773 | -1,27 | 4,66E-20 |
| BCL6 | NM_001130845 | 1,47 | 4,67E-20 |
| KIAA0101 | NM_014736 | -1,70 | 4,68E-20 |
| C10orf72 | NM_001031746 | 1,96 | 4,76E-20 |
| CAV2 | NM_198212 | -1,08 | 4,78E-20 |
| SFRS7 | NM_001031684 | -1,72 | 4,85E-20 |
| ATP1B1 | NM_001677 | -1,42 | 4,87E-20 |
| WWOX | NM_016373 | 2,59 | 4,88E-20 |
| ICAM1 | NM_000201 | -2,74 | 5,08E-20 |
| STAC | NM_003149 | -2,09 | 5,51E-20 |
| TMX2 | NR_026593 | -1,15 | 5,68E-20 |
| SERBP1 | NM_001018068 | -1,03 | 5,72E-20 |
| DOT1L | NM_032482 | -0,97 | 5,73E-20 |
| ABCF2 | NM_007189 | -1,03 | 5,84E-20 |
| ZNF436 | NM_001077195 | 1,46 | 5,96E-20 |
| NUP35 | NM_138285 | -2,08 | 6,67E-20 |
| SPATA20 | NM_022827 | 1,57 | 6,82E-20 |
| PLDN | NM_012388 | -1,07 | 6,99E-20 |
| C6orf167 | NM_198468 | -1,74 | 7,50E-20 |
| NCRNA00174 | NR_026873 | 2,60 | 7,64E-20 |
| SYNGR1 | NM_004711 | 1,87 | 8,44E-20 |
| MAP2K5 | NM_002757 | 1,51 | 8,58E-20 |
| BDH2 | NM_020139 | 2,05 | 9,79E-20 |
| TOMM5 | NM_001134484 | -1,67 | 9,85E-20 |
| C9orf16 | NM_024112 | 1,38 | 1,07E-19 |
| ETF1 | NM_004730 | -1,00 | 1,08E-19 |
| PHF17 | NM_199320 | -1,36 | 1,10E-19 |
| TGFB2 | NM_003238 | -1,65 | 1,12E-19 |
| C13orf37 | NM_001071775 | -1,83 | 1,14E-19 |
| MSTP2 | NR_027504 | 4,69 | 1,17E-19 |
| SACS | NM_014363 | -1,33 | 1,18E-19 |
| SEC23B | NM_032985 | -1,30 | 1,19E-19 |
| MRPL17 | NM_022061 | -1,21 | 1,21E-19 |
| NOP16 | NM_016391 | -2,35 | 1,26E-19 |
| RRP9 | NM_004704 | -1,94 | 1,27E-19 |
| DHX9 | NM_001357 | -1,01 | 1,27E-19 |
| MANBA | NM_005908 | 1,49 | 1,33E-19 |
| GLIPR1 | NM_006851 | -1,72 | 1,43E-19 |
| NUP155 | NM_153485 | -1,48 | 1,51E-19 |
| TLN1 | NM_006289 | -1,02 | 1,60E-19 |
| GPR162 | NM_019858 | 2,29 | 1,60E-19 |
| QSOX2 | NM_181701 | -1,23 | 1,66E-19 |
| NXT1 | NM_013248 | -1,68 | 1,73E-19 |
| CCT3 | NM_001008883 | -1,08 | 1,74E-19 |
| PIGW | NM_178517 | -2,01 | 1,75E-19 |
| SLC4A3 | NM_005070 | 1,88 | 1,91E-19 |
| ZEB1 | NR_024286 | 1,57 | 2,02E-19 |
| TMEM63A | NM_014698 | 1,35 | 2,09E-19 |
| TRIM58 | NM_015431 | -2,23 | 2,17E-19 |
| VCAN | NM_001126336 | -1,12 | 2,25E-19 |
| SFXN5 | NM_144579 | 1,70 | 2,29E-19 |
| SQRDL | NM_021199 | 1,45 | 3,07E-19 |
| ZNF217 | NM_006526 | -1,25 | 3,15E-19 |
| SAT1 | NM_002970 | 1,89 | 3,20E-19 |
| AEBP1 | NM_001129 | 1,18 | 3,77E-19 |
| LTA4H | NM_000895 | 1,19 | 3,78E-19 |
| AKR1C2 | NM_205845 | 4,54 | 3,79E-19 |
| GLI1 | NM_005269 | 2,68 | 3,88E-19 |
| CYP26B1 | NM_019885 | -3,83 | 3,91E-19 |
| ASB1 | NM_001040445 | -1,34 | 4,13E-19 |
| LHFP | NM_005780 | 1,45 | 4,71E-19 |
| CHKB-CPT1B | NR_027928 | 1,79 | 4,82E-19 |
| LIPG | NM_006033 | -4,25 | 5,11E-19 |
| AHDC1 | NM_001029882 | 1,21 | 5,36E-19 |
| CPD | NM_001304 | 1,03 | 5,42E-19 |
| IDH2 | NM_002168 | -1,12 | 6,08E-19 |
| CSTF2 | NM_001325 | -1,64 | 6,09E-19 |
| TENC1 | NM_015319 | 1,37 | 6,12E-19 |
| BEGAIN | NM_020836 | 4,59 | 6,85E-19 |
| MYO18A | NM_078471 | 1,28 | 6,89E-19 |
| RAD51AP1 | NM_001130862 | -2,15 | 6,89E-19 |
| CLIC1 | NM_001288 | -1,13 | 6,98E-19 |
| RNF103 | NM_005667 | 1,38 | 7,01E-19 |
| P4HA1 | NM_000917 | -1,04 | 7,27E-19 |
| COL8A1 | NM_020351 | -0,85 | 7,31E-19 |
| CHD1L | NM_004284 | -1,36 | 7,34E-19 |
| FBXL8 | NM_018378 | 2,89 | 7,49E-19 |
| GMFB | NM_004124 | -1,05 | 8,15E-19 |
| NICN1 | NM_032316 | 1,74 | 8,47E-19 |
| WDR19 | NM_025132 | 1,65 | 8,72E-19 |
| HAUS8 | NM_033417 | -2,58 | 9,25E-19 |
| KIAA1908 | NR_027329 | 1,88 | 9,25E-19 |
| DDX17 | NM_001098504 | 0,94 | 9,50E-19 |
| PSRC1 | NM_001032290 | -1,60 | 9,54E-19 |
| GRAMD4 | NM_015124 | 1,61 | 9,64E-19 |
| PPP3CA | NM_001130692 | 1,02 | 9,65E-19 |
| IL32 | NM_001012636 | -3,20 | 9,77E-19 |
| CNDP2 | NM_018235 | -1,39 | 9,86E-19 |
| IGFBP3 | NM_000598 | 1,23 | 1,05E-18 |
| NHS | NM_198270 | 1,96 | 1,05E-18 |
| EXOSC8 | NM_181503 | -1,70 | 1,06E-18 |
| IER5L | NM_203434 | 1,26 | 1,06E-18 |
| PNPT1 | NM_033109 | -1,41 | 1,06E-18 |
| TNFRSF10D | NM_003840 | -0,98 | 1,10E-18 |
| SLC2A12 | NM_145176 | 2,03 | 1,12E-18 |
| STARD10 | NM_006645 | 2,29 | 1,12E-18 |
| ABHD8 | NM_024527 | 2,13 | 1,15E-18 |
| C14orf145 | NM_152446 | -2,38 | 1,15E-18 |
| SEH1L | NM_031216 | -1,34 | 1,15E-18 |
| C20orf117 | NM_080627 | -1,34 | 1,20E-18 |
| REEP4 | NM_025232 | -1,53 | 1,34E-18 |
| CACNA1C | NM_001129831 | 1,56 | 1,35E-18 |
| OSBPL7 | NM_145798 | 1,62 | 1,36E-18 |
| SH3PXD2A | NM_014631 | 1,07 | 1,40E-18 |
| MYO1C | NM_033375 | -0,94 | 1,44E-18 |
| YPEL5 | NM_001127399 | 1,17 | 1,45E-18 |
| SH2D5 | NM_001103161 | -4,09 | 1,46E-18 |
| VASP | NM_003370 | -1,48 | 1,46E-18 |
| ANGEL1 | NM_015305 | 1,56 | 1,47E-18 |
| TMEM106B | NM_001134232 | 1,36 | 1,59E-18 |
| GNE | NM_001128227 | -1,28 | 1,67E-18 |
| DPP9 | NM_139159 | -0,99 | 1,72E-18 |
| KLC4 | NM_201523 | 1,72 | 1,72E-18 |
| ZNF425 | NM_001001661 | 2,73 | 1,78E-18 |
| PTGIS | NM_000961 | 2,43 | 1,81E-18 |
| BCL6B | NM_181844 | -4,63 | 1,82E-18 |
| ID1 | NM_002165 | -1,45 | 1,83E-18 |
| SDK2 | NM_001144952 | 2,83 | 1,85E-18 |
| IL1R1 | NM_000877 | 1,71 | 2,03E-18 |
| CIRH1A | NM_032830 | -1,44 | 2,04E-18 |
| GMPPB | NM_021971 | -1,72 | 2,12E-18 |
| NOC3L | NM_022451 | -1,49 | 2,35E-18 |
| ELAVL1 | NM_001419 | -1,11 | 2,49E-18 |
| DENND4C | NM_017925 | 1,37 | 2,49E-18 |
| ILF2 | NM_004515 | -1,08 | 2,57E-18 |
| NPC1 | NM_000271 | -1,17 | 2,69E-18 |
| RNF145 | NM_144726 | -1,06 | 2,96E-18 |
| ATAD5 | NM_024857 | -2,11 | 3,10E-18 |
| SYNGAP1 | NM_006772 | 1,54 | 3,18E-18 |
| STRAP | NM_007178 | -0,87 | 3,28E-18 |
| SEZ6L2 | NM_201575 | 1,97 | 3,29E-18 |
| AFF3 | NM_002285 | 1,48 | 3,51E-18 |
| RBBP8 | NM_002894 | -1,55 | 3,67E-18 |
| PNPLA7 | NM_001098537 | 3,14 | 3,68E-18 |
| FZD1 | NM_003505 | 1,23 | 3,71E-18 |
| C1orf63 | NM_020317 | 1,51 | 3,73E-18 |
| SERTAD2 | NM_014755 | -1,30 | 3,80E-18 |
| CX3CL1 | NM_002996 | 4,75 | 3,83E-18 |
| KIF21B | NM_017596 | -1,69 | 4,11E-18 |
| GNL3 | NM_206826 | -1,29 | 4,11E-18 |
| SMARCA4 | NM_001128846 | -0,91 | 4,11E-18 |
| ROBO1 | NM_001145845 | 1,04 | 4,13E-18 |
| NOP10 | NM_018648 | -1,15 | 4,51E-18 |
| EFHC1 | NM_018100 | 1,75 | 4,55E-18 |
| AHSA1 | NM_012111 | -1,26 | 4,62E-18 |
| CRAT | NR_028048 | 1,14 | 4,65E-18 |
| SMOX | NM_175839 | 1,44 | 4,74E-18 |
| OSR1 | NM_145260 | 1,67 | 4,74E-18 |
| OGDH | NM_002541 | -0,95 | 4,83E-18 |
| SRPK1 | NM_003137 | -1,10 | 4,95E-18 |
| ZNF259 | NM_003904 | -1,29 | 4,96E-18 |
| YRDC | NM_024640 | -1,59 | 5,10E-18 |
| NOL11 | NM_015462 | -1,34 | 5,53E-18 |
| PRMT1 | NM_198319 | -1,02 | 5,55E-18 |
| SYNJ2 | NM_003898 | 1,21 | 5,99E-18 |
| LYNX1 | NM_177457 | 1,62 | 6,01E-18 |
| ZNF423 | NM_015069 | 1,41 | 6,74E-18 |
| NCL | NM_005381 | -1,30 | 6,83E-18 |
| NRGN | NM_001126181 | -1,90 | 7,20E-18 |
| NARS | NM_004539 | -0,93 | 7,33E-18 |
| SLC7A6 | NM_003983 | -1,17 | 7,69E-18 |
| PPIL5 | NM_152329 | -2,05 | 7,76E-18 |
| PKN3 | NM_013355 | -1,83 | 8,09E-18 |
| NEIL3 | NM_018248 | -2,49 | 8,34E-18 |
| DAXX | NR_024517 | -1,05 | 8,75E-18 |
| EPB41L1 | NM_177996 | 1,40 | 8,84E-18 |
| DHFR | NM_000791 | -1,41 | 9,03E-18 |
| TTLL12 | NM_015140 | -1,23 | 9,47E-18 |
| GNA11 | NM_002067 | -1,12 | 9,47E-18 |
| SUSD5 | NM_015551 | -1,07 | 9,77E-18 |
| EFHD2 | NM_024329 | -1,25 | 9,77E-18 |
| CLSTN3 | NM_014718 | 1,94 | 1,01E-17 |
| DOCK6 | NM_020812 | 1,29 | 1,06E-17 |
| THOP1 | NM_003249 | -1,72 | 1,06E-17 |
| LHPP | NM_022126 | 2,24 | 1,07E-17 |
| DAPK3 | NM_001348 | -1,19 | 1,07E-17 |
| ICOSLG | NM_015259 | 2,97 | 1,09E-17 |
| IRF1 | NM_002198 | 1,59 | 1,13E-17 |
| NUP85 | NM_024844 | -1,37 | 1,14E-17 |
| BTN3A2 | NM_007047 | 1,53 | 1,17E-17 |
| KIF5B | NM_004521 | -0,85 | 1,18E-17 |
| STK36 | NM_015690 | 1,25 | 1,22E-17 |
| NMT2 | NM_004808 | -1,27 | 1,22E-17 |
| HECA | NM_016217 | 1,23 | 1,23E-17 |
| DBI | NM_001079862 | -1,50 | 1,25E-17 |
| TRMT6 | NM_015939 | -1,69 | 1,28E-17 |
| PLAG1 | NM_002655 | 1,66 | 1,29E-17 |
| SSB | NM_003142 | -0,93 | 1,29E-17 |
| NFIB | NM_005596 | 1,19 | 1,29E-17 |
| LETM1 | NM_012318 | -1,06 | 1,32E-17 |
| ACPL2 | NM_152282 | 1,64 | 1,35E-17 |
| RAB23 | NM_016277 | -1,15 | 1,36E-17 |
| NIPA2 | NM_030922 | -1,15 | 1,37E-17 |
| MEIS3 | NM_020160 | 2,46 | 1,39E-17 |
| WDR3 | NM_006784 | -1,28 | 1,39E-17 |
| RSL1D1 | NM_015659 | -0,93 | 1,51E-17 |
| DENND4A | NM_005848 | 1,52 | 1,53E-17 |
| PLCD3 | NM_133373 | 1,23 | 1,56E-17 |
| PDK4 | NM_002612 | 3,95 | 1,56E-17 |
| TPM4 | NM_003290 | -0,98 | 1,58E-17 |
| PLAGL1 | NM_001080951 | 1,40 | 1,65E-17 |
| S1PR1 | NM_001400 | -2,92 | 1,66E-17 |
| TBC1D17 | NM_024682 | 1,35 | 1,68E-17 |
| GAB2 | NM_012296 | 1,35 | 1,69E-17 |
| PAN2 | NM_001166279 | 1,32 | 1,75E-17 |
| VWA5A | NM_001130142 | 3,33 | 1,79E-17 |
| APOL6 | NM_030641 | 2,03 | 1,80E-17 |
| RAB8A | NM_005370 | -1,08 | 1,98E-17 |
| IRAK1 | NM_001025242 | -0,92 | 1,98E-17 |
| SOX11 | NM_003108 | -1,50 | 2,00E-17 |
| ZNF581 | NM_016535 | 1,42 | 2,01E-17 |
| CCDC86 | NM_024098 | -0,95 | 2,02E-17 |
| FAM126A | NM_032581 | -1,03 | 2,02E-17 |
| TEK | NM_000459 | -3,26 | 2,08E-17 |
| MAFK | NM_002360 | -1,42 | 2,10E-17 |
| ZNF251 | NM_138367 | 1,40 | 2,11E-17 |
| FDFT1 | NM_004462 | -1,33 | 2,24E-17 |
| TNFRSF1A | NM_001065 | 0,99 | 2,34E-17 |
| HEXDC | NM_173620 | 1,96 | 2,36E-17 |
| PKM2 | NM_002654 | -0,94 | 2,41E-17 |
| MICAL3 | NM_001136004 | -1,54 | 2,43E-17 |
| RNPS1 | NM_080594 | -1,11 | 2,54E-17 |
| ZMYND19 | NM_138462 | -1,38 | 2,61E-17 |
| MARCH8 | NM_001002265 | 1,53 | 2,70E-17 |
| TDG | NM_003211 | -1,20 | 2,74E-17 |
| RNF26 | NM_032015 | -1,10 | 2,80E-17 |
| RNASEH2A | NM_006397 | -1,98 | 2,86E-17 |
| MMP16 | NM_005941 | -1,04 | 2,95E-17 |
| POLD2 | NM_006230 | -1,06 | 3,04E-17 |
| IFT80 | NM_020800 | 1,47 | 3,04E-17 |
| SCARB1 | NM_005505 | -1,55 | 3,11E-17 |
| BMS1 | NM_014753 | -1,03 | 3,14E-17 |
| PKP4 | NM_001005476 | -1,16 | 3,18E-17 |
| ZNF783 | NR_015357 | 2,06 | 3,20E-17 |
| HMCN1 | NM_031935 | 1,26 | 3,26E-17 |
| PSMC3 | NM_002804 | -1,26 | 3,28E-17 |
| CBX7 | NM_175709 | 2,47 | 3,29E-17 |
| PHB | NM_002634 | -1,16 | 3,41E-17 |
| VPS37D | NM_001077621 | 2,89 | 3,46E-17 |
| MICALL2 | NM_182924 | 1,16 | 3,70E-17 |
| C10orf58 | NM_032333 | 1,50 | 3,71E-17 |
| DUSP10 | NM_007207 | 1,93 | 3,71E-17 |
| ARHGEF15 | NM_173728 | -3,94 | 3,84E-17 |
| NUP93 | NM_014669 | -1,34 | 3,93E-17 |
| GNG11 | NM_004126 | 1,34 | 3,94E-17 |
| EXOSC3 | NM_016042 | -1,61 | 3,99E-17 |
| ANGPTL2 | NM_012098 | 1,81 | 4,24E-17 |
| SENP3 | NM_015670 | -0,96 | 4,40E-17 |
| SRRT | NM_001128852 | -0,98 | 4,46E-17 |
| SLC5A6 | NM_021095 | -1,31 | 4,59E-17 |
| NT5DC3 | NM_001031701 | -1,43 | 4,69E-17 |
| MBNL1 | NM_207296 | -1,25 | 4,72E-17 |
| CABC1 | NM_020247 | 1,68 | 4,79E-17 |
| DNAH1 | NM_015512 | 2,38 | 4,96E-17 |
| MALAT1 | NR_002819 | 1,24 | 4,97E-17 |
| FAM63A | NM_018379 | 1,71 | 5,02E-17 |
| NECAB3 | NM_031231 | 2,06 | 5,02E-17 |
| PSMD1 | NM_002807 | -0,95 | 5,11E-17 |
| LIMD1 | NM_014240 | -0,99 | 5,24E-17 |
| SIPA1L1 | NM_015556 | 1,19 | 5,31E-17 |
| NCOA2 | NM_006540 | 1,53 | 5,66E-17 |
| DTYMK | NM_012145 | -1,83 | 5,74E-17 |
| DCAF8 | NR_028104 | 1,07 | 5,74E-17 |
| LOC100129550 | NR_024618 | 2,67 | 5,95E-17 |
| FBXO32 | NM_058229 | 4,02 | 6,12E-17 |
| SUV39H2 | NM_024670 | -1,94 | 6,72E-17 |
| GATS | NR_028038 | 2,38 | 6,73E-17 |
| KCTD10 | NM_031954 | -0,95 | 6,82E-17 |
| MMRN1 | NM_007351 | -4,00 | 6,83E-17 |
| ZBTB44 | NM_014155 | 1,31 | 6,94E-17 |
| PTRF | NM_012232 | -1,52 | 6,98E-17 |
| D2HGDH | NM_152783 | 1,54 | 7,17E-17 |
| PTEN | NM_000314 | 0,94 | 7,19E-17 |
| SCNN1D | NM_001130413 | 2,72 | 7,35E-17 |
| PAK1IP1 | NM_017906 | -1,66 | 7,78E-17 |
| MPRIP | NM_201274 | -0,90 | 7,89E-17 |
| DCBLD2 | NM_080927 | -0,87 | 7,90E-17 |
| CBFA2T2 | NM_005093 | 1,26 | 8,04E-17 |
| TRIM2 | NM_001130067 | 1,54 | 8,26E-17 |
| ARNTL2 | NM_020183 | -2,39 | 8,54E-17 |
| COL1A1 | NM_000088 | 1,20 | 8,89E-17 |
| ERI1 | NM_153332 | -1,44 | 8,94E-17 |
| PFDN1 | NM_002622 | -1,07 | 8,94E-17 |
| UCHL5 | NM_015984 | -1,66 | 8,98E-17 |
| NFIA | NM_005595 | 1,52 | 9,08E-17 |
| GALNT1 | NM_020474 | -0,90 | 9,11E-17 |
| TMEM109 | NM_024092 | -1,00 | 9,20E-17 |
| PKNOX2 | NM_022062 | 1,47 | 9,46E-17 |
| IDH3A | NM_005530 | -1,19 | 9,72E-17 |
| CTF1 | NM_001330 | 2,12 | 9,75E-17 |
| LOC283070 | NR_027322 | 1,91 | 9,75E-17 |
| CERCAM | NM_016174 | 1,04 | 9,80E-17 |
| PRKDC | NM_001081640 | -1,07 | 9,94E-17 |
| C6orf173 | NM_001012507 | -2,47 | 1,00E-16 |
| PALM | NM_001040134 | 1,55 | 1,02E-16 |
| HSPA8 | NM_153201 | -0,77 | 1,03E-16 |
| SIX5 | NM_175875 | 1,33 | 1,10E-16 |
| GPR116 | NM_001098518 | -3,33 | 1,10E-16 |
| C2orf69 | NM_153689 | -1,49 | 1,10E-16 |
| RHPN1 | NM_052924 | 3,00 | 1,12E-16 |
| ATP9A | NM_006045 | 1,27 | 1,21E-16 |
| UBE2K | NM_005339 | -1,13 | 1,23E-16 |
| ADARB1 | NM_015833 | -1,08 | 1,23E-16 |
| IFIT3 | NM_001549 | 1,93 | 1,24E-16 |
| RNASEL | NM_021133 | 1,69 | 1,25E-16 |
| SLC16A4 | NM_004696 | 2,40 | 1,49E-16 |
| TIPARP | NM_015508 | 1,27 | 1,54E-16 |
| PPAN | NM_020230 | -1,38 | 1,55E-16 |
| TIMM17A | NM_006335 | -1,34 | 1,58E-16 |
| KIAA0513 | NM_014732 | 1,39 | 1,60E-16 |
| MPHOSPH6 | NM_005792 | -2,22 | 1,62E-16 |
| GALNT10 | NM_198321 | -0,90 | 1,76E-16 |
| C15orf39 | NM_015492 | -1,06 | 1,77E-16 |
| MYOCD | NM_153604 | -1,45 | 1,80E-16 |
| FXYD5 | NR_028406 | -1,21 | 1,81E-16 |
| PACSIN2 | NM_007229 | -1,27 | 1,82E-16 |
| SLC27A4 | NM_005094 | -1,10 | 1,85E-16 |
| KCNE3 | NM_005472 | 3,05 | 1,86E-16 |
| CHEK1 | NM_001274 | -1,62 | 1,90E-16 |
| PHF19 | NM_001009936 | -1,74 | 1,91E-16 |
| PPP1R14B | NM_138689 | -1,28 | 2,07E-16 |
| WDR45 | NM_001029896 | 1,28 | 2,08E-16 |
| ATP8B1 | NM_005603 | -1,10 | 2,08E-16 |
| NEAT1 | NR_028272 | 1,84 | 2,12E-16 |
| ZNF446 | NM_017908 | 1,66 | 2,26E-16 |
| EIF5AL1 | NM_001099692 | -1,58 | 2,31E-16 |
| ACAD11 | NM_032169 | 1,29 | 2,35E-16 |
| COL5A2 | NM_000393 | 0,97 | 2,43E-16 |
| MIB2 | NM_080875 | 1,49 | 2,43E-16 |
| WSB1 | NM_015626 | 0,98 | 2,51E-16 |
| IFI16 | NM_005531 | 1,08 | 2,53E-16 |
| INO80C | NM_194281 | -2,05 | 2,54E-16 |
| GNS | NM_002076 | 0,82 | 2,63E-16 |
| KBTBD10 | NM_006063 | 4,15 | 2,68E-16 |
| TMEM130 | NM_001134450 | 2,93 | 2,71E-16 |
| DNTTIP2 | NM_014597 | -1,09 | 2,73E-16 |
| GTF2IRD2 | NM_173537 | 2,50 | 2,77E-16 |
| AKT1 | NM_001014432 | -0,83 | 2,79E-16 |
| PRRT3 | NM_207351 | 1,90 | 2,95E-16 |
| HERC2P2 | NR_002824 | 1,05 | 3,05E-16 |
| IRF9 | NM_006084 | 1,40 | 3,08E-16 |
| FAM171A1 | NM_001010924 | -1,19 | 3,10E-16 |
| HNRNPA3 | NM_194247 | -0,99 | 3,10E-16 |
| NCBP1 | NM_002486 | -1,05 | 3,11E-16 |
| NUDC | NM_006600 | -1,44 | 3,20E-16 |
| CEP78 | NM_032171 | -1,59 | 3,26E-16 |
| TMEM132B | NM_052907 | 1,88 | 3,30E-16 |
| ACSS3 | NM_024560 | 2,49 | 3,37E-16 |
| POLR1E | NM_022490 | -1,26 | 3,41E-16 |
| CUL9 | NM_015089 | 1,37 | 3,41E-16 |
| HARS | NM_002109 | -1,07 | 3,53E-16 |
| XRCC3 | NM_001100118 | -1,40 | 3,59E-16 |
| GFPT2 | NM_005110 | 1,76 | 3,59E-16 |
| MGC87042 | NM_207342 | -2,33 | 3,69E-16 |
| S1PR3 | NM_005226 | 1,40 | 3,79E-16 |
| PFN1 | NM_005022 | -1,19 | 3,81E-16 |
| SUMO3 | NM_006936 | -0,91 | 4,01E-16 |
| DARS2 | NM_018122 | -1,55 | 4,02E-16 |
| CYHR1 | NM_032687 | 1,88 | 4,19E-16 |
| PDXP | NM_020315 | -1,43 | 4,32E-16 |
| GTF2IP1 | NR_002206 | 1,10 | 4,33E-16 |
| LOC100093631 | NR_003580 | 1,09 | 4,36E-16 |
| NBAS | NM_015909 | 1,10 | 4,48E-16 |
| RBM12 | NM_006047 | -0,99 | 4,50E-16 |
| NAT10 | NM_024662 | -1,09 | 4,52E-16 |
| CTSL1 | NM_145918 | 1,12 | 4,53E-16 |
| DOK5 | NM_018431 | -1,74 | 4,61E-16 |
| TBX18 | NM_001080508 | 1,27 | 4,81E-16 |
| GALE | NM_001127621 | -1,67 | 4,84E-16 |
| STEAP3 | NM_182915 | 1,17 | 4,89E-16 |
| C5orf45 | NM_001017987 | 1,61 | 4,89E-16 |
| EBP | NM_006579 | -1,84 | 4,94E-16 |
| LSG1 | NM_018385 | -1,23 | 5,00E-16 |
| ATHL1 | NM_025092 | 1,35 | 5,15E-16 |
| RMI1 | NM_024945 | -1,67 | 5,16E-16 |
| HDAC2 | NM_001527 | -0,95 | 5,38E-16 |
| ZIC4 | NM_032153 | 1,74 | 5,39E-16 |
| UBE2H | NM_003344 | 1,06 | 5,45E-16 |
| RHEB | NM_005614 | -1,14 | 5,57E-16 |
| KCTD18 | NM_152387 | 1,56 | 5,65E-16 |
| C20orf27 | NM_001039140 | -1,30 | 5,68E-16 |
| PSMD7 | NM_002811 | -0,95 | 5,94E-16 |
| CDC25C | NM_001790 | -2,03 | 6,07E-16 |
| CENPL | NM_033319 | -1,96 | 6,20E-16 |
| C1orf104 | NM_001039517 | -1,73 | 6,42E-16 |
| FOXH1 | NM_003923 | 2,21 | 6,86E-16 |
| DPF1 | NM_001135156 | -2,66 | 6,91E-16 |
| KDM5B | NM_006618 | 0,89 | 7,15E-16 |
| MTHFD1L | NM_015440 | -1,03 | 7,29E-16 |
| C12orf11 | NM_018164 | -1,16 | 7,47E-16 |
| C18orf54 | NM_173529 | -1,81 | 7,52E-16 |
| THRA | NM_003250 | 1,61 | 7,55E-16 |
| PACS2 | NM_015197 | 0,94 | 7,58E-16 |
| TARS | NM_152295 | -1,02 | 7,59E-16 |
| TMEM80 | NM_001042463 | 1,67 | 7,69E-16 |
| C3orf59 | NM_178496 | -1,53 | 7,84E-16 |
| MRPL3 | NM_007208 | -0,98 | 7,85E-16 |
| LENG8 | NM_052925 | 1,15 | 8,00E-16 |
| FGD5 | NM_152536 | -3,35 | 8,06E-16 |
| EME1 | NM_001166131 | -2,04 | 8,27E-16 |
| FUBP1 | NM_003902 | -1,39 | 8,43E-16 |
| PCDH18 | NM_019035 | 1,08 | 8,48E-16 |
| C19orf48 | NM_199249 | -1,22 | 8,58E-16 |
| WDR52 | NM_001164496 | 2,23 | 8,65E-16 |
| PWP1 | NM_007062 | -1,04 | 8,70E-16 |
| FSTL1 | NM_007085 | -0,71 | 8,74E-16 |
| XPC | NM_004628 | 1,28 | 8,77E-16 |
| TTF2 | NM_003594 | -1,47 | 8,78E-16 |
| UBE2E3 | NM_182678 | 1,07 | 9,02E-16 |
| ANXA6 | NM_001155 | -0,96 | 9,17E-16 |
| ZBTB2 | NM_020861 | -1,41 | 9,50E-16 |
| RECQL | NM_032941 | -1,12 | 9,62E-16 |
| CALR | NM_004343 | -1,04 | 9,67E-16 |
| SLC40A1 | NM_014585 | 3,37 | 9,67E-16 |
| BARD1 | NM_000465 | -2,32 | 9,94E-16 |
| LRP4 | NM_002334 | 1,10 | 1,00E-15 |
| PSMD12 | NM_002816 | -1,27 | 1,04E-15 |
| KARS | NM_001130089 | -1,01 | 1,05E-15 |
| GINS3 | NM_001126129 | -2,32 | 1,10E-15 |
| DONSON | NM_017613 | -1,34 | 1,11E-15 |
| TARDBP | NM_007375 | -1,10 | 1,11E-15 |
| ZDHHC1 | NM_013304 | 1,93 | 1,12E-15 |
| PTGFRN | NM_020440 | 1,01 | 1,13E-15 |
| LOC647979 | NR_027451 | 0,91 | 1,15E-15 |
| IFI44L | NM_006820 | 4,11 | 1,18E-15 |
| EXOSC2 | NM_014285 | -1,45 | 1,19E-15 |
| CAMK2D | NM_172128 | 0,83 | 1,21E-15 |
| PCOLCE2 | NM_013363 | -2,06 | 1,29E-15 |
| MKRN1 | NM_013446 | 1,12 | 1,32E-15 |
| TMEM48 | NM_018087 | -1,85 | 1,35E-15 |
| LOC646471 | NR_024498 | 1,84 | 1,36E-15 |
| LOC253039 | NR_024408 | 1,61 | 1,38E-15 |
| CIRBP | NM_001280 | 1,34 | 1,44E-15 |
| NUP153 | NM_005124 | -1,11 | 1,45E-15 |
| APOBEC3F | NM_145298 | 2,34 | 1,57E-15 |
| KRT18 | NM_199187 | -1,38 | 1,59E-15 |
| ZNF469 | NM_001127464 | -1,17 | 1,63E-15 |
| CREB3L1 | NM_052854 | -1,09 | 1,63E-15 |
| HEATR1 | NM_018072 | -1,08 | 1,65E-15 |
| SLC25A24 | NM_013386 | -1,12 | 1,65E-15 |
| MVD | NM_002461 | -1,74 | 1,65E-15 |
| ALDH2 | NM_000690 | 1,37 | 1,66E-15 |
| LOC100272228 | NR_027456 | 2,76 | 1,70E-15 |
| MEX3D | NM_203304 | -1,28 | 1,71E-15 |
| RGL1 | NM_015149 | 1,41 | 1,71E-15 |
| TRPC6 | NM_004621 | -1,43 | 1,81E-15 |
| AP1M1 | NM_032493 | -0,96 | 1,84E-15 |
| OPRL1 | NM_000913 | 2,46 | 1,85E-15 |
| PURB | NM_033224 | -1,16 | 1,94E-15 |
| SMAP2 | NM_022733 | -1,12 | 1,95E-15 |
| ALS2CR8 | NM_024744 | 2,88 | 1,96E-15 |
| AP1S3 | NM_001039569 | -2,17 | 2,03E-15 |
| CSF1 | NM_000757 | 1,24 | 2,08E-15 |
| STX2 | NM_194356 | -1,11 | 2,19E-15 |
| SPCS3 | NM_021928 | -0,87 | 2,20E-15 |
| ADHFE1 | NM_144650 | 4,27 | 2,27E-15 |
| PPFIBP2 | NM_003621 | 1,40 | 2,32E-15 |
| AGRN | NM_198576 | 1,04 | 2,36E-15 |
| LRRC56 | NM_198075 | 3,47 | 2,39E-15 |
| CDC123 | NM_006023 | -1,04 | 2,40E-15 |
| CHRNA5 | NM_000745 | -2,82 | 2,41E-15 |
| GCC2 | NM_181453 | 1,25 | 2,41E-15 |
| CD320 | NM_016579 | -1,67 | 2,44E-15 |
| GART | NM_175085 | -1,03 | 2,55E-15 |
| CYTH2 | NM_004228 | 1,26 | 2,56E-15 |
| ITGA2 | NM_002203 | 2,02 | 2,59E-15 |
| CENPA | NM_001809 | -2,09 | 2,61E-15 |
| BEND3 | NM_001080450 | -1,54 | 2,76E-15 |
| VSIG10L | NM_001163922 | 2,36 | 2,79E-15 |
| KIAA0090 | NM_015047 | -1,00 | 2,83E-15 |
| GAS2L1 | NM_152237 | -1,03 | 2,88E-15 |
| CAT | NM_001752 | 1,30 | 2,92E-15 |
| FGF16 | NM_003868 | -3,26 | 2,94E-15 |
| U2AF2 | NM_007279 | -0,85 | 2,97E-15 |
| MYO19 | NM_025109 | -1,03 | 3,06E-15 |
| RBM5 | NM_005778 | 0,89 | 3,08E-15 |
| TMEM201 | NM_001010866 | -1,30 | 3,15E-15 |
| C4orf14 | NM_032313 | 1,12 | 3,15E-15 |
| COL4A1 | NM_001845 | 0,78 | 3,15E-15 |
| TBKBP1 | NM_014726 | 1,63 | 3,32E-15 |
| PGM2 | NM_018290 | -1,23 | 3,36E-15 |
| ADCK4 | NM_001142555 | 1,58 | 3,40E-15 |
| HIST2H2BE | NM_003528 | 2,44 | 3,40E-15 |
| PDZRN3 | NM_015009 | 1,26 | 3,43E-15 |
| ADCY3 | NM_004036 | 1,09 | 3,43E-15 |
| CKS2 | NM_001827 | -2,23 | 3,70E-15 |
| USP20 | NM_001008563 | 1,19 | 3,81E-15 |
| GIGYF1 | NM_022574 | 1,23 | 4,09E-15 |
| C18orf55 | NM_014177 | -1,57 | 4,12E-15 |
| ZNF238 | NM_006352 | -1,18 | 4,14E-15 |
| ZNF516 | NM_014643 | 1,26 | 4,18E-15 |
| C1R | NM_001733 | 1,87 | 4,22E-15 |
| ARHGEF6 | NM_004840 | 1,24 | 4,23E-15 |
| ACTG1 | NM_001614 | -0,96 | 4,28E-15 |
| MALL | NM_005434 | -2,37 | 4,34E-15 |
| THBS2 | NM_003247 | 0,97 | 4,34E-15 |
| UMPS | NM_000373 | -1,54 | 4,77E-15 |
| RGAG4 | NM_001024455 | 1,14 | 4,84E-15 |
| EIF4G2 | NM_001042559 | -0,89 | 4,88E-15 |
| ENOSF1 | NM_001126123 | -1,59 | 4,91E-15 |
| RP2 | NM_006915 | -1,41 | 5,00E-15 |
| TUBGCP6 | NM_020461 | 1,00 | 5,02E-15 |
| MVK | NM_001114185 | -1,85 | 5,04E-15 |
| LRRC27 | NM_030626 | 2,28 | 5,06E-15 |
| SH3BP4 | NM_014521 | -1,02 | 5,31E-15 |
| RPA1 | NM_002945 | -1,09 | 5,33E-15 |
| HSPB8 | NM_014365 | -1,10 | 5,45E-15 |
| NES | NM_006617 | -1,28 | 5,63E-15 |
| E2F3 | NM_001949 | -1,23 | 5,76E-15 |
| PLEKHM1P | NR_024386 | 1,73 | 5,76E-15 |
| ZNF37B | NR_026777 | 1,50 | 5,90E-15 |
| DDX12 | NM_004400 | -1,49 | 6,15E-15 |
| SEC1 | NR_004401 | 3,86 | 6,16E-15 |
| APOC1 | NM_001645 | 4,08 | 6,27E-15 |
| COL4A5 | NM_033380 | 1,26 | 6,30E-15 |
| MSN | NM_002444 | -1,06 | 6,57E-15 |
| MBP | NM_001025100 | 1,39 | 6,57E-15 |
| PUS1 | NM_001002019 | -1,51 | 6,57E-15 |
| PWP2 | NM_005049 | -1,31 | 6,83E-15 |
| RPN1 | NM_002950 | -0,79 | 6,92E-15 |
| C1QTNF1 | NM_030968 | 2,28 | 7,25E-15 |
| CES2 | NM_003869 | 1,08 | 7,29E-15 |
| VPS41 | NM_014396 | 1,02 | 7,29E-15 |
| DCLK1 | NM_004734 | 2,05 | 7,56E-15 |
| LARP4B | NM_015155 | -0,92 | 7,57E-15 |
| CCNE2 | NM_057749 | -2,29 | 7,65E-15 |
| CDR2 | NM_001802 | -1,16 | 7,74E-15 |
| GEMIN4 | NM_015721 | -1,19 | 7,91E-15 |
| PGM2L1 | NM_173582 | 1,39 | 8,03E-15 |
| KIAA1407 | NM_020817 | 2,76 | 8,07E-15 |
| RQCD1 | NM_005444 | -1,41 | 8,10E-15 |
| NUCKS1 | NM_022731 | -0,79 | 8,19E-15 |
| CTSK | NM_000396 | 1,65 | 8,40E-15 |
| DBP | NM_001352 | 3,78 | 8,52E-15 |
| FLJ32065 | NR_026903 | 2,07 | 8,68E-15 |
| DYNLL2 | NM_080677 | -1,26 | 8,70E-15 |
| KCNT2 | NM_198503 | 2,47 | 8,94E-15 |
| TMEM184A | NM_001097620 | -1,34 | 9,20E-15 |
| ATP1B3 | NM_001679 | -0,90 | 9,42E-15 |
| GOLGA8A | NR_027409 | 0,95 | 9,63E-15 |
| ACSF2 | NM_025149 | 2,04 | 9,82E-15 |
| FTSJ1 | NM_177439 | -1,04 | 1,05E-14 |
| LBH | NM_030915 | -1,46 | 1,10E-14 |
| HPRT1 | NM_000194 | -1,53 | 1,12E-14 |
| GIMAP4 | NM_018326 | -4,49 | 1,13E-14 |
| ASAP3 | NM_017707 | 1,83 | 1,14E-14 |
| HSD11B1L | NM_198707 | 2,47 | 1,15E-14 |
| C16orf59 | NM_025108 | -1,82 | 1,15E-14 |
| USP10 | NM_005153 | -1,02 | 1,18E-14 |
| SHMT1 | NM_004169 | -1,75 | 1,18E-14 |
| PSMD3 | NM_002809 | -0,86 | 1,20E-14 |
| DLG4 | NM_001128827 | 0,94 | 1,21E-14 |
| ALAD | NM_000031 | 1,07 | 1,21E-14 |
| DNAJC18 | NM_152686 | 1,48 | 1,22E-14 |
| SLC7A2 | NM_001008539 | -1,38 | 1,23E-14 |
| SOCS1 | NM_003745 | -2,75 | 1,28E-14 |
| GBP1 | NM_002053 | -1,49 | 1,28E-14 |
| ACTL6A | NM_177989 | -1,22 | 1,28E-14 |
| NR4A2 | NM_006186 | 3,12 | 1,29E-14 |
| NOC2L | NM_015658 | -1,26 | 1,37E-14 |
| RB1CC1 | NM_001083617 | 0,90 | 1,37E-14 |
| KLC1 | NM_005552 | -0,90 | 1,37E-14 |
| RAD54B | NM_012415 | -2,13 | 1,40E-14 |
| TIMM23 | NM_006327 | -1,06 | 1,43E-14 |
| C14orf139 | NR_026779 | 1,63 | 1,47E-14 |
| ANXA1 | NM_000700 | -0,94 | 1,48E-14 |
| CCT7 | NM_001009570 | -1,16 | 1,51E-14 |
| PART1 | NR_028509 | 3,30 | 1,51E-14 |
| PIM1 | NM_002648 | -1,32 | 1,55E-14 |
| DNALI1 | NM_003462 | 1,77 | 1,56E-14 |
| RAPGEF3 | NM_006105 | 1,83 | 1,61E-14 |
| BOK | NM_032515 | -1,05 | 1,63E-14 |
| CUX1 | NM_181500 | 1,08 | 1,66E-14 |
| PRPF4 | NM_004697 | -1,53 | 1,67E-14 |
| GSTM2 | NM_000848 | 2,10 | 1,71E-14 |
| HNRNPU | NM_004501 | -0,98 | 1,71E-14 |
| BIN1 | NM_139346 | 1,02 | 1,72E-14 |
| RCOR3 | NM_001136223 | 1,28 | 1,73E-14 |
| POLR3G | NM_006467 | -2,03 | 1,74E-14 |
| SOX18 | NM_018419 | -2,95 | 1,74E-14 |
| RBM14 | NM_006328 | -1,06 | 1,80E-14 |
| KIAA0913 | NM_015037 | 0,85 | 1,80E-14 |
| GABARAP | NM_007278 | 0,89 | 1,82E-14 |
| COL6A3 | NM_057166 | 0,82 | 1,85E-14 |
| DCK | NM_000788 | -1,32 | 1,85E-14 |
| PTPRU | NM_133177 | 1,76 | 1,86E-14 |
| MKI67IP | NM_032390 | -1,12 | 1,87E-14 |
| SLBP | NM_006527 | -1,23 | 1,87E-14 |
| PLS3 | NM_005032 | -0,88 | 1,95E-14 |
| MLEC | NM_014730 | -0,82 | 1,95E-14 |
| PPIA | NM_021130 | -0,82 | 2,01E-14 |
| C5orf30 | NM_033211 | -1,43 | 2,02E-14 |
| ACBD4 | NM_024722 | 2,71 | 2,05E-14 |
| GNPDA1 | NM_005471 | 1,17 | 2,05E-14 |
| SLC3A2 | NM_001012661 | -1,16 | 2,08E-14 |
| PDIA3 | NM_005313 | -0,79 | 2,09E-14 |
| CXorf36 | NM_176819 | -3,27 | 2,13E-14 |
| TCP1 | NM_030752 | -1,18 | 2,14E-14 |
| ARMC6 | NM_033415 | -1,26 | 2,33E-14 |
| UHRF2 | NM_152896 | 1,27 | 2,44E-14 |
| YWHAZ | NM_145690 | -0,96 | 2,48E-14 |
| GPX4 | NM_002085 | 1,05 | 2,57E-14 |
| GAS1 | NM_002048 | 2,00 | 2,64E-14 |
| TNFRSF14 | NM_003820 | 2,20 | 2,66E-14 |
| USP31 | NM_020718 | -1,40 | 2,70E-14 |
| WDR36 | NM_139281 | -0,98 | 2,74E-14 |
| C1S | NM_201442 | 2,70 | 2,74E-14 |
| CEP68 | NM_015147 | 1,06 | 2,74E-14 |
| RANBP3L | NM_001161429 | 5,13 | 2,82E-14 |
| CARD10 | NM_014550 | 1,44 | 2,86E-14 |
| SLC6A7 | NM_014228 | 4,61 | 2,86E-14 |
| GIMAP6 | NM_024711 | -3,67 | 2,87E-14 |
| RRN3 | NM_018427 | -1,08 | 2,92E-14 |
| KDELC2 | NM_153705 | -1,11 | 2,97E-14 |
| HDAC9 | NM_014707 | -2,69 | 3,05E-14 |
| TRPV2 | NM_016113 | -1,10 | 3,17E-14 |
| USP13 | NM_003940 | -1,08 | 3,18E-14 |
| GPN3 | NM_016301 | -1,97 | 3,19E-14 |
| SSH3 | NM_017857 | 1,57 | 3,33E-14 |
| SNRNP40 | NM_004814 | -1,24 | 3,43E-14 |
| G2E3 | NM_017769 | -1,29 | 3,53E-14 |
| SSBP2 | NM_012446 | 1,92 | 3,54E-14 |
| SLC17A9 | NM_022082 | -1,94 | 3,64E-14 |
| RNF219 | NM_024546 | -1,48 | 3,67E-14 |
| BGN | NM_001711 | 1,05 | 3,67E-14 |
| RAB27B | NM_004163 | 2,83 | 3,70E-14 |
| HSPA2 | NM_021979 | -2,18 | 3,70E-14 |
| CLCN7 | NM_001114331 | 1,17 | 3,71E-14 |
| SMPD4 | NM_017751 | -1,05 | 3,87E-14 |
| CDKN1C | NM_001122630 | 3,30 | 3,87E-14 |
| CALU | NM_001219 | -0,89 | 3,91E-14 |
| RGL2 | NR_028387 | 1,15 | 4,00E-14 |
| WRAP53 | NM_018081 | -1,57 | 4,03E-14 |
| POLR3K | NM_016310 | -1,83 | 4,04E-14 |
| BAG5 | NM_001015048 | -1,08 | 4,08E-14 |
| P2RX6 | NM_005446 | 4,12 | 4,34E-14 |
| TWISTNB | NM_001002926 | -1,24 | 4,61E-14 |
| VCL | NM_014000 | -0,96 | 4,67E-14 |
| KLHL22 | NM_032775 | 1,39 | 4,91E-14 |
| QPRT | NM_014298 | 2,07 | 4,95E-14 |
| PITPNB | NM_012399 | -0,83 | 5,02E-14 |
| AZIN1 | NM_015878 | -0,85 | 5,22E-14 |
| MYL12A | NM_006471 | -1,27 | 5,24E-14 |
| STRA13 | NM_144998 | -1,98 | 5,31E-14 |
| MAPRE3 | NM_012326 | 2,10 | 5,39E-14 |
| XPO1 | NM_003400 | -0,77 | 5,46E-14 |
| MIA3 | NM_198551 | 1,04 | 5,54E-14 |
| SLC16A1 | NM_001166496 | -0,96 | 5,57E-14 |
| LUC7L3 | NM_016424 | 0,94 | 5,71E-14 |
| MYCT1 | NM_025107 | -3,46 | 5,75E-14 |
| GFM1 | NM_024996 | -1,10 | 5,76E-14 |
| SLC26A11 | NM_001166347 | 1,60 | 6,20E-14 |
| ROBO3 | NM_022370 | 1,37 | 6,22E-14 |
| FAM72A | NM_001123168 | -2,02 | 6,25E-14 |
| PYROXD2 | NM_032709 | 1,79 | 6,26E-14 |
| RAB3IL1 | NM_013401 | 2,12 | 6,27E-14 |
| VPS39 | NM_015289 | 0,88 | 6,31E-14 |
| NUP62 | NM_012346 | -1,16 | 6,49E-14 |
| MRPS12 | NM_033363 | -1,27 | 6,52E-14 |
| RTN4 | NM_207521 | -1,01 | 6,72E-14 |
| BZW1L1 | NR_026584 | -0,90 | 6,75E-14 |
| CYS1 | NM_001037160 | 3,76 | 6,92E-14 |
| NDRG4 | NM_020465 | 1,60 | 7,03E-14 |
| KIAA0430 | NM_014647 | 1,07 | 7,24E-14 |
| SLC7A14 | NM_020949 | 2,76 | 7,26E-14 |
| CD109 | NM_001159587 | 1,19 | 7,43E-14 |
| DEPDC1B | NM_018369 | -1,78 | 7,48E-14 |
| CHKB | NM_005198 | 1,71 | 7,63E-14 |
| CHD6 | NM_032221 | 0,98 | 7,65E-14 |
| LTB4R | NM_181657 | 1,70 | 7,65E-14 |
| PPP1R16B | NM_015568 | -3,60 | 7,67E-14 |
| RFC5 | NM_001130113 | -1,42 | 7,74E-14 |
| C3orf45 | NM_153215 | -1,62 | 7,77E-14 |
| FAT1 | NM_005245 | -0,97 | 7,79E-14 |
| LOC728554 | NR_003615 | -1,26 | 7,80E-14 |
| CS | NM_004077 | -0,88 | 8,14E-14 |
| RNF44 | NM_014901 | 1,05 | 8,18E-14 |
| NASP | NM_172164 | -1,19 | 8,20E-14 |
| SMAGP | NM_001031628 | -1,57 | 8,31E-14 |
| UBL3 | NM_007106 | 1,03 | 8,36E-14 |
| FANCM | NM_020937 | -1,77 | 8,42E-14 |
| AP1B1 | NM_001166019 | -0,85 | 8,46E-14 |
| LRRC40 | NM_017768 | -1,47 | 8,67E-14 |
| NPLOC4 | NM_017921 | -0,77 | 8,82E-14 |
| TTTY15 | NR_001545 | -1,59 | 8,85E-14 |
| PLOD3 | NM_001084 | -0,87 | 9,09E-14 |
| SLC35B4 | NM_032826 | -1,20 | 9,13E-14 |
| GPSM2 | NM_013296 | -1,38 | 9,24E-14 |
| SORT1 | NM_002959 | -0,98 | 9,24E-14 |
| CBX1 | NM_006807 | -1,05 | 9,31E-14 |
| POLI | NM_007195 | 1,53 | 9,36E-14 |
| FAM43A | NM_153690 | 1,17 | 9,71E-14 |
| ADRM1 | NM_007002 | -0,93 | 9,98E-14 |
| C18orf19 | NM_001098801 | -1,33 | 1,01E-13 |
| RNF144A | NM_014746 | 1,74 | 1,01E-13 |
| EDN1 | NM_001955 | -4,06 | 1,03E-13 |
| ATP13A3 | NM_024524 | -0,89 | 1,06E-13 |
| EXD3 | NM_017820 | 3,04 | 1,09E-13 |
| PGAP3 | NM_033419 | 1,53 | 1,10E-13 |
| LUZP1 | NM_001142546 | -1,01 | 1,10E-13 |
| PTPN14 | NM_005401 | -1,37 | 1,12E-13 |
| C12orf48 | NM_017915 | -1,83 | 1,14E-13 |
| CD3EAP | NM_012099 | -1,02 | 1,14E-13 |
| SMO | NM_005631 | 1,16 | 1,14E-13 |
| RECQL5 | NM_004259 | 1,36 | 1,14E-13 |
| SFXN1 | NM_022754 | -0,95 | 1,17E-13 |
| PLD1 | NM_002662 | 1,66 | 1,18E-13 |
| AGBL5 | NM_001035507 | 1,09 | 1,21E-13 |
| RARA | NM_001024809 | 1,01 | 1,22E-13 |
| C3orf52 | NM_024616 | -2,83 | 1,23E-13 |
| C17orf53 | NM_024032 | -1,80 | 1,25E-13 |
| GABPB1 | NM_002041 | -1,52 | 1,26E-13 |
| CHAC1 | NM_024111 | -1,37 | 1,27E-13 |
| PRIM2 | NM_000947 | -1,68 | 1,31E-13 |
| BCAR1 | NM_014567 | -0,88 | 1,32E-13 |
| GRINL1A | NR_027390 | 1,00 | 1,39E-13 |
| RAB32 | NM_006834 | -1,33 | 1,40E-13 |
| SULT1B1 | NM_014465 | -5,05 | 1,42E-13 |
| DDX60 | NM_017631 | 1,79 | 1,44E-13 |
| DACH2 | NM_001139514 | 2,50 | 1,48E-13 |
| SLC16A2 | NM_006517 | 1,48 | 1,55E-13 |
| INO80B | NM_031288 | 1,59 | 1,56E-13 |
| LMF1 | NM_022773 | 1,54 | 1,58E-13 |
| RARRES3 | NM_004585 | 4,21 | 1,60E-13 |
| NCOA3 | NM_006534 | 1,02 | 1,61E-13 |
| LASP1 | NM_006148 | -0,84 | 1,64E-13 |
| NPEPL1 | NM_024663 | 1,23 | 1,71E-13 |
| SERTAD4 | NM_019605 | 1,58 | 1,71E-13 |
| HEATR3 | NM_182922 | -1,50 | 1,75E-13 |
| SH3TC2 | NM_024577 | -3,14 | 1,79E-13 |
| CAMK1G | NM_020439 | 3,07 | 1,80E-13 |
| LIG1 | NM_000234 | -1,14 | 1,88E-13 |
| GLIS2 | NM_032575 | 1,12 | 1,93E-13 |
| UTP15 | NM_032175 | -1,56 | 1,94E-13 |
| C11orf48 | NM_024099 | -1,24 | 1,94E-13 |
| TICAM2 | NM_021649 | -1,39 | 1,95E-13 |
| UBA2 | NM_005499 | -0,82 | 2,00E-13 |
| MBTD1 | NM_017643 | 1,38 | 2,02E-13 |
| UTP3 | NM_020368 | -1,17 | 2,04E-13 |
| TNRC18 | NM_001080495 | 1,00 | 2,08E-13 |
| LENG9 | NM_198988 | 1,26 | 2,09E-13 |
| HCFC1R1 | NM_001002017 | 1,34 | 2,10E-13 |
| ADAMTS18 | NM_199355 | -4,72 | 2,24E-13 |
| ITPR3 | NM_002224 | -1,06 | 2,26E-13 |
| IQCE | NM_152558 | 1,13 | 2,29E-13 |
| VCP | NM_007126 | -1,10 | 2,35E-13 |
| TXNDC12 | NM_015913 | -0,99 | 2,37E-13 |
| NPC2 | NM_006432 | 1,05 | 2,44E-13 |
| MARCKSL1 | NM_023009 | -0,85 | 2,46E-13 |
| UBQLN1 | NM_053067 | -0,91 | 2,53E-13 |
| AHCTF1 | NM_015446 | -1,08 | 2,61E-13 |
| PSMC4 | NM_006503 | -1,00 | 2,61E-13 |
| SNRPA1 | NM_003090 | -1,73 | 2,65E-13 |
| EWSR1 | NM_001163285 | -0,77 | 2,66E-13 |
| SIAE | NM_170601 | 1,35 | 2,66E-13 |
| PAPLN | NM_173462 | 2,10 | 2,75E-13 |
| LOC642852 | NR_026943 | 0,87 | 2,80E-13 |
| MAN2B1 | NM_000528 | 0,96 | 2,82E-13 |
| ABHD15 | NM_198147 | 1,47 | 2,83E-13 |
| SURF4 | NM_033161 | -0,73 | 2,83E-13 |
| PPA1 | NM_021129 | -1,24 | 2,85E-13 |
| MPDU1 | NM_004870 | -1,14 | 2,95E-13 |
| UGCG | NM_003358 | -1,28 | 2,97E-13 |
| ARPC1A | NM_006409 | -0,84 | 2,98E-13 |
| GLI4 | NM_138465 | 1,92 | 2,98E-13 |
| C4B | NM_001002029 | 2,32 | 3,06E-13 |
| C4A | NM_007293 | 2,32 | 3,06E-13 |
| TMEM129 | NM_138385 | 1,16 | 3,14E-13 |
| CCDC21 | NM_022778 | -1,19 | 3,26E-13 |
| FBXO45 | NM_001105573 | -1,22 | 3,32E-13 |
| C3orf18 | NM_016210 | 2,10 | 3,40E-13 |
| JAG1 | NM_000214 | -0,74 | 3,43E-13 |
| SMARCC2 | NM_139067 | 0,90 | 3,51E-13 |
| TTLL4 | NM_014640 | -1,19 | 3,53E-13 |
| ATIC | NM_004044 | -0,93 | 3,54E-13 |
| MTAP | NM_002451 | -1,00 | 3,56E-13 |
| RAPH1 | NM_213589 | -1,18 | 3,57E-13 |
| C10orf41 | NR_024421 | 3,14 | 3,57E-13 |
| ABCF1 | NM_001025091 | -0,82 | 3,60E-13 |
| ZNF641 | NM_152320 | 2,08 | 3,60E-13 |
| PFKFB2 | NM_006212 | 2,00 | 3,62E-13 |
| GTF2IRD2B | NM_001003795 | 2,36 | 3,64E-13 |
| MTA2 | NM_004739 | -0,82 | 3,66E-13 |
| UACA | NM_018003 | 0,71 | 3,72E-13 |
| ID4 | NM_001546 | -1,44 | 3,81E-13 |
| LRRC32 | NM_001128922 | -1,12 | 3,85E-13 |
| ZYX | NM_003461 | -0,84 | 3,90E-13 |
| C9orf7 | NM_017586 | 1,74 | 3,93E-13 |
| C6orf153 | NM_033112 | -1,28 | 3,93E-13 |
| SLC2A11 | NM_030807 | 2,22 | 3,99E-13 |
| REXO1 | NM_020695 | -0,83 | 4,05E-13 |
| ANPEP | NM_001150 | 0,91 | 4,08E-13 |
| GPR124 | NM_032777 | 1,03 | 4,12E-13 |
| ARRB1 | NM_004041 | 1,69 | 4,14E-13 |
| TESK2 | NM_007170 | 2,07 | 4,14E-13 |
| GIPC1 | NM_202494 | -0,92 | 4,21E-13 |
| SLC25A42 | NM_178526 | 1,73 | 4,22E-13 |
| IRS2 | NM_003749 | 1,13 | 4,29E-13 |
| COL19A1 | NM_001858 | 4,53 | 4,35E-13 |
| EIF1AX | NM_001412 | -1,07 | 4,50E-13 |
| SLC27A3 | NM_024330 | 1,90 | 4,54E-13 |
| HNRNPL | NM_001005335 | -0,93 | 4,66E-13 |
| PANK3 | NM_024594 | -1,01 | 4,67E-13 |
| PLEKHG4 | NM_001129728 | 1,02 | 4,68E-13 |
| CPAMD8 | NM_015692 | 3,85 | 4,73E-13 |
| ACAD10 | NM_001136538 | 1,44 | 4,79E-13 |
| ANGPT2 | NM_001147 | 1,86 | 4,84E-13 |
| TMEM33 | NM_018126 | -0,94 | 4,94E-13 |
| CAP2 | NM_006366 | -1,76 | 5,08E-13 |
| C15orf52 | NM_207380 | -1,04 | 5,20E-13 |
| AEN | NM_022767 | -1,03 | 5,22E-13 |
| EXOC4 | NM_021807 | 0,88 | 5,41E-13 |
| RSU1 | NM_012425 | -1,45 | 5,66E-13 |
| C2CD2L | NM_014807 | -1,08 | 5,68E-13 |
| CLN6 | NM_017882 | -1,43 | 5,79E-13 |
| PCSK7 | NM_004716 | -0,95 | 5,82E-13 |
| ISCU | NM_014301 | 1,13 | 5,85E-13 |
| LHX6 | NM_014368 | -3,36 | 5,93E-13 |
| TNFRSF19 | NM_148957 | 2,10 | 5,98E-13 |
| SSRP1 | NM_003146 | -1,04 | 6,12E-13 |
| B2M | NM_004048 | 0,83 | 6,13E-13 |
| MLLT6 | NM_005937 | 0,95 | 6,17E-13 |
| PABPC1L | NM_001124756 | 1,28 | 6,42E-13 |
| AFF1 | NM_005935 | 1,26 | 6,44E-13 |
| WDR5 | NM_052821 | -1,02 | 6,50E-13 |
| PRMT5 | NM_001039619 | -1,00 | 6,52E-13 |
| CTNNAL1 | NM_003798 | -1,03 | 6,76E-13 |
| GAB1 | NM_207123 | 1,49 | 6,76E-13 |
| VMAC | NM_001017921 | 2,12 | 6,95E-13 |
| UBR5 | NM_015902 | 0,86 | 7,03E-13 |
| TRMT61A | NM_152307 | -1,40 | 7,07E-13 |
| ELFN2 | NM_052906 | 2,18 | 7,27E-13 |
| SLC30A10 | NM_018713 | 5,19 | 7,28E-13 |
| TANC2 | NM_025185 | -1,31 | 7,35E-13 |
| HELLS | NM_018063 | -1,92 | 7,36E-13 |
| DCAF15 | NM_138353 | -1,01 | 7,62E-13 |
| ATOH8 | NM_032827 | -1,47 | 7,69E-13 |
| NTNG2 | NM_032536 | 3,81 | 7,75E-13 |
| STBD1 | NM_003943 | -1,84 | 8,14E-13 |
| PRPF19 | NM_014502 | -0,84 | 8,43E-13 |
| ATP5B | NM_001686 | -0,68 | 8,60E-13 |
| GPR137 | NM_020155 | 1,10 | 8,66E-13 |
| BZW1 | NM_014670 | -0,92 | 9,01E-13 |
| B4GALT5 | NM_004776 | -0,88 | 9,16E-13 |
| FIBIN | NM_203371 | 3,49 | 9,18E-13 |
| SH3GLB2 | NM_020145 | 0,93 | 9,29E-13 |
| LOC90110 | NR_026953 | 1,96 | 9,44E-13 |
| HIF1A | NM_181054 | -1,05 | 9,60E-13 |
| C13orf1 | NM_020456 | -1,45 | 9,67E-13 |
| TJP2 | NM_004817 | -0,80 | 9,70E-13 |
| GLI2 | NM_005270 | -1,37 | 1,01E-12 |
| ARMCX1 | NM_016608 | 1,09 | 1,01E-12 |
| PGAP1 | NM_024989 | 1,32 | 1,01E-12 |
| EXOSC10 | NM_002685 | -0,88 | 1,02E-12 |
| NAPEPLD | NM_001122838 | 1,42 | 1,05E-12 |
| CAV1 | NM_001753 | -0,92 | 1,06E-12 |
| TSPAN18 | NM_130783 | -2,57 | 1,06E-12 |
| UBE3C | NM_014671 | -0,78 | 1,06E-12 |
| CLK4 | NM_020666 | 1,77 | 1,06E-12 |
| NUAK1 | NM_014840 | -1,24 | 1,06E-12 |
| MUC20 | NM_152673 | 4,40 | 1,07E-12 |
| SETD8 | NM_020382 | -1,04 | 1,07E-12 |
| NIPAL2 | NM_024759 | 1,62 | 1,12E-12 |
| DEGS1 | NM_003676 | -0,86 | 1,15E-12 |
| DCUN1D5 | NM_032299 | -1,20 | 1,15E-12 |
| ZNF598 | NM_178167 | -0,83 | 1,15E-12 |
| TDP1 | NM_018319 | -1,37 | 1,17E-12 |
| PIP5K1A | NM_001135638 | -0,91 | 1,22E-12 |
| TRIM66 | NM_014818 | 1,93 | 1,22E-12 |
| ADAMTS16 | NM_139056 | 1,50 | 1,27E-12 |
| TFEB | NM_007162 | 2,19 | 1,29E-12 |
| M6PR | NM_002355 | -0,94 | 1,32E-12 |
| PARP2 | NM_005484 | -1,23 | 1,33E-12 |
| C21orf45 | NM_018944 | -1,88 | 1,37E-12 |
| FANCG | NM_004629 | -1,32 | 1,39E-12 |
| NOP14 | NM_003703 | -1,00 | 1,40E-12 |
| IGBP1 | NM_001551 | 1,12 | 1,43E-12 |
| SNHG1 | NR_003098 | -0,95 | 1,43E-12 |
| SH3BP5 | NM_004844 | 1,10 | 1,45E-12 |
| DHRS1 | NM_138452 | 1,77 | 1,45E-12 |
| LARP1 | NM_033551 | -0,84 | 1,48E-12 |
| JMJD1C | NM_004241 | 1,35 | 1,50E-12 |
| CCNG1 | NM_004060 | 0,97 | 1,53E-12 |
| RIMS3 | NM_014747 | 2,82 | 1,63E-12 |
| NR1H3 | NM_005693 | 2,31 | 1,63E-12 |
| FAM60A | NM_001135811 | -1,26 | 1,67E-12 |
| ZKSCAN1 | NM_003439 | 1,06 | 1,69E-12 |
| TMEM59 | NM_004872 | 1,04 | 1,70E-12 |
| GTF2IRD2P | NR_002164 | 2,53 | 1,78E-12 |
| ATXN3 | NM_001164775 | 1,41 | 1,78E-12 |
| SGOL1 | NM_001012409 | -2,37 | 1,79E-12 |
| DNAJA1 | NM_001539 | -1,04 | 1,79E-12 |
| FANCB | NM_001018113 | -3,14 | 1,83E-12 |
| BBS2 | NM_031885 | 1,11 | 1,89E-12 |
| TMEM165 | NM_018475 | -0,89 | 1,89E-12 |
| SMC3 | NM_005445 | -0,81 | 1,91E-12 |
| APBA2 | NM_005503 | -1,15 | 1,91E-12 |
| LRFN4 | NM_024036 | -1,00 | 1,94E-12 |
| FAM168B | NM_001009993 | -0,76 | 1,96E-12 |
| PDCD4 | NM_014456 | 1,01 | 1,97E-12 |
| ANKRD28 | NM_015199 | -0,85 | 2,01E-12 |
| IFIH1 | NM_022168 | 3,30 | 2,02E-12 |
| FNIP1 | NM_001008738 | 1,07 | 2,08E-12 |
| DCLRE1B | NM_022836 | -1,25 | 2,16E-12 |
| GRAMD3 | NM_001146322 | -1,33 | 2,23E-12 |
| PDE12 | NM_177966 | -1,14 | 2,26E-12 |
| BRI3BP | NM_080626 | -2,13 | 2,28E-12 |
| JMJD7-PLA2G4B | NR_015346 | 1,41 | 2,28E-12 |
| POMP | NM_015932 | -1,10 | 2,29E-12 |
| KRT34 | NM_021013 | -4,22 | 2,35E-12 |
| SMC6 | NM_024624 | -0,95 | 2,35E-12 |
| CALCOCO2 | NM_005831 | 0,89 | 2,37E-12 |
| SH3BP1 | NM_018957 | 1,16 | 2,41E-12 |
| PCNXL2 | NM_014801 | 1,73 | 2,41E-12 |
| EDA2R | NM_021783 | 2,09 | 2,43E-12 |
| SMARCD3 | NM_003078 | 1,03 | 2,52E-12 |
| COL24A1 | NM_152890 | 3,91 | 2,55E-12 |
| RPL7L1 | NM_198486 | -1,15 | 2,55E-12 |
| KIAA1841 | NM_001129993 | -1,76 | 2,79E-12 |
| FLNA | NM_001110556 | -0,89 | 2,83E-12 |
| RNF213 | NM_020914 | 0,87 | 2,85E-12 |
| CDRT4 | NM_173622 | 2,39 | 2,90E-12 |
| ZBTB22 | NM_005453 | 1,25 | 2,91E-12 |
| BBC3 | NM_014417 | 1,85 | 2,95E-12 |
| C7orf60 | NM_152556 | 1,34 | 2,99E-12 |
| RRP7A | NM_015703 | -1,39 | 3,04E-12 |
| PFDN2 | NM_012394 | -1,29 | 3,06E-12 |
| KCNJ8 | NM_004982 | -2,34 | 3,11E-12 |
| NIN | NM_182944 | -0,81 | 3,18E-12 |
| PNPLA2 | NM_020376 | 0,89 | 3,26E-12 |
| KIAA1715 | NM_030650 | -1,02 | 3,30E-12 |
| KCNMA1 | NM_002247 | 1,09 | 3,30E-12 |
| ARHGAP17 | NM_001006634 | -1,09 | 3,30E-12 |
| ATP5G1 | NM_001002027 | -1,30 | 3,40E-12 |
| MUSTN1 | NM_205853 | 3,45 | 3,41E-12 |
| MARCH6 | NM_005885 | 0,85 | 3,41E-12 |
| BOLA3 | NM_212552 | -1,70 | 3,48E-12 |
| HIGD1A | NM_001099669 | -1,13 | 3,48E-12 |
| PARP9 | NM_031458 | 1,61 | 3,53E-12 |
| TMEM104 | NM_017728 | -1,01 | 3,54E-12 |
| SLC43A3 | NM_017611 | -1,81 | 3,63E-12 |
| DBNDD2 | NM_001048226 | -1,31 | 3,70E-12 |
| ABHD12 | NM_001042472 | -0,90 | 3,70E-12 |
| GSTT1 | NM_000853 | 1,27 | 3,72E-12 |
| GDI1 | NM_001493 | -0,75 | 3,72E-12 |
| NUPR1 | NM_001042483 | 1,79 | 3,76E-12 |
| ZCCHC5 | NM_152694 | -4,77 | 3,77E-12 |
| RDH10 | NM_172037 | 1,66 | 3,84E-12 |
| LTV1 | NM_032860 | -1,38 | 3,96E-12 |
| LDB1 | NM_003893 | 0,97 | 4,00E-12 |
| RAD23B | NM_002874 | -0,82 | 4,06E-12 |
| ZNF185 | NM_007150 | -1,38 | 4,07E-12 |
| PLEKHM1 | NR_027782 | 0,98 | 4,10E-12 |
| TMEM175 | NM_032326 | 1,08 | 4,10E-12 |
| HLA-B | NM_005514 | 0,93 | 4,13E-12 |
| NAT13 | NM_025146 | -0,82 | 4,16E-12 |
| PRPS2 | NM_002765 | -1,20 | 4,18E-12 |
| C1orf198 | NM_001136494 | -1,12 | 4,20E-12 |
| ERG | NM_182918 | -1,95 | 4,20E-12 |
| LCA5 | NM_001122769 | 1,92 | 4,34E-12 |
| IARS | NM_013417 | -0,66 | 4,35E-12 |
| TCEB3 | NM_003198 | -0,83 | 4,48E-12 |
| FLJ35220 | NM_173627 | 1,99 | 4,48E-12 |
| ENTPD7 | NM_020354 | -1,07 | 4,50E-12 |
| C4orf34 | NM_174921 | 1,11 | 4,51E-12 |
| PSMG1 | NM_203433 | -1,34 | 4,58E-12 |
| BCAT1 | NM_005504 | -0,71 | 4,63E-12 |
| PLOD1 | NM_000302 | -0,69 | 4,66E-12 |
| TPPP3 | NM_016140 | -2,37 | 4,76E-12 |
| ANKRD52 | NM_173595 | -0,73 | 4,94E-12 |
| PLXNB2 | NM_012401 | 0,78 | 4,96E-12 |
| DSCC1 | NM_024094 | -2,22 | 4,98E-12 |
| PSMD8 | NM_002812 | -0,80 | 5,09E-12 |
| VDAC2 | NM_003375 | -0,99 | 5,18E-12 |
| CHPF | NM_024536 | 0,91 | 5,21E-12 |
| DDX41 | NM_016222 | -0,95 | 5,33E-12 |
| SAMD9L | NM_152703 | 1,61 | 5,35E-12 |
| EIF4B | NM_001417 | 1,04 | 5,50E-12 |
| TFRC | NM_003234 | -0,94 | 5,52E-12 |
| TTC14 | NM_001042601 | 1,08 | 5,70E-12 |
| TIMM50 | NM_001001563 | -1,12 | 5,70E-12 |
| PINK1 | NM_032409 | 0,92 | 5,78E-12 |
| CCDC88A | NM_001135597 | -0,93 | 5,78E-12 |
| LOC100129034 | NR_027406 | 0,80 | 5,92E-12 |
| GGH | NM_003878 | -1,45 | 6,00E-12 |
| LRDD | NM_145886 | 1,33 | 6,03E-12 |
| CCDC159 | NM_001080503 | 2,37 | 6,04E-12 |
| GEN1 | NM_001130009 | -1,30 | 6,06E-12 |
| CDX1 | NM_001804 | 3,17 | 6,06E-12 |
| ROBO2 | NM_001128929 | 1,32 | 6,10E-12 |
| MAGED2 | NM_177433 | 0,96 | 6,12E-12 |
| CXCL1 | NM_001511 | -2,89 | 6,21E-12 |
| SH3BGRL | NM_003022 | 1,13 | 6,35E-12 |
| MYL12B | NM_033546 | -0,88 | 6,55E-12 |
| IFFO1 | NM_001039670 | 0,91 | 6,57E-12 |
| STX17 | NM_017919 | 1,18 | 6,60E-12 |
| LETMD1 | NM_015416 | 1,17 | 6,72E-12 |
| ZNF385A | NM_001130967 | 1,57 | 6,73E-12 |
| METTL13 | NM_001007239 | -1,13 | 6,79E-12 |
| PNMAL1 | NM_018215 | 1,28 | 6,93E-12 |
| RAP1GAP2 | NM_015085 | -1,32 | 7,09E-12 |
| COQ2 | NM_015697 | -1,72 | 7,11E-12 |
| DSEL | NM_032160 | -0,88 | 7,11E-12 |
| PTN | NM_002825 | 1,52 | 7,15E-12 |
| PHF15 | NM_015288 | 1,17 | 7,21E-12 |
| GSTCD | NM_024751 | -1,61 | 7,21E-12 |
| AP1S2 | NM_003916 | -1,02 | 7,31E-12 |
| CNNM2 | NM_199076 | 1,41 | 7,31E-12 |
| PHLDA2 | NM_003311 | -0,80 | 7,32E-12 |
| DIRAS2 | NM_017594 | 5,03 | 7,32E-12 |
| MCCC1 | NM_020166 | 1,52 | 7,52E-12 |
| ARPC4 | NM_001024959 | -0,91 | 7,61E-12 |
| UBE2G1 | NM_003342 | -0,88 | 7,74E-12 |
| ST6GALNAC6 | NM_013443 | 0,76 | 7,90E-12 |
| RIOK1 | NM_031480 | -1,19 | 7,92E-12 |
| MSRB3 | NM_001031679 | -0,92 | 7,94E-12 |
| DAK | NM_015533 | 1,26 | 8,01E-12 |
| MTMR9L | NR_026850 | 1,80 | 8,08E-12 |
| DHX33 | NM_020162 | -1,21 | 8,13E-12 |
| TAGLN | NM_001001522 | -1,27 | 8,25E-12 |
| SGSH | NM_000199 | 1,04 | 8,25E-12 |
| LOC339535 | NR_015407 | 2,44 | 8,39E-12 |
| IFI27 | NM_001130080 | -2,72 | 8,39E-12 |
| SAMD10 | NM_080621 | 2,25 | 8,39E-12 |
| MYO7B | NM_001080527 | -3,45 | 8,53E-12 |
| EHMT2 | NM_006709 | 0,80 | 8,61E-12 |
| CAPN3 | NM_024344 | 3,41 | 8,67E-12 |
| HNRPDL | NM_031372 | -0,70 | 8,93E-12 |
| GOLIM4 | NM_014498 | -0,76 | 8,95E-12 |
| HNRNPF | NM_001098208 | -0,74 | 9,09E-12 |
| YWHAE | NR_024058 | -0,70 | 9,18E-12 |
| BTBD6 | NM_033271 | -1,18 | 9,23E-12 |
| MNT | NM_020310 | 0,91 | 9,25E-12 |
| FAM116B | NM_001001794 | 2,46 | 9,26E-12 |
| RUFY3 | NM_014961 | 1,40 | 9,28E-12 |
| CHORDC1 | NM_001144073 | -1,04 | 9,32E-12 |
| SRFBP1 | NM_152546 | -1,70 | 9,54E-12 |
| PDSS1 | NM_014317 | -2,02 | 9,91E-12 |
| KRR1 | NM_007043 | -1,25 | 9,93E-12 |
| PTPRB | NM_001109754 | -2,86 | 1,01E-11 |
| LOC146880 | NR_027487 | 1,84 | 1,03E-11 |
| R3HDM2 | NM_014925 | 1,29 | 1,04E-11 |
| MOBKL2B | NM_024761 | -2,14 | 1,04E-11 |
| PDCL3 | NM_024065 | -1,52 | 1,05E-11 |
| DISP1 | NM_032890 | 1,85 | 1,05E-11 |
| KCTD5 | NM_018992 | -1,11 | 1,05E-11 |
| PSMC1 | NM_002802 | -0,88 | 1,06E-11 |
| SUFU | NM_016169 | 1,11 | 1,06E-11 |
| CDC7 | NM_001134419 | -1,40 | 1,07E-11 |
| HSP90AB1 | NM_007355 | -0,87 | 1,08E-11 |
| SLC39A11 | NM_001159770 | 1,55 | 1,08E-11 |
| TOMM34 | NM_006809 | -0,99 | 1,10E-11 |
| ANKRD24 | NM_133475 | 4,26 | 1,11E-11 |
| FGF2 | NM_002006 | -1,23 | 1,11E-11 |
| MTFR1 | NM_014637 | -1,20 | 1,12E-11 |
| KIAA0664 | NM_015229 | -0,92 | 1,12E-11 |
| SNHG3 | NR_002909 | -1,01 | 1,14E-11 |
| IGF2BP2 | NM_006548 | -0,87 | 1,16E-11 |
| OSBPL5 | NM_020896 | 0,96 | 1,16E-11 |
| KANK1 | NM_153186 | -1,22 | 1,18E-11 |
| EVL | NM_016337 | 1,13 | 1,19E-11 |
| EML3 | NM_153265 | 1,06 | 1,20E-11 |
| BCCIP | NM_078468 | -1,16 | 1,20E-11 |
| LARP4 | NM_199190 | -1,08 | 1,28E-11 |
| SRXN1 | NM_080725 | -0,94 | 1,30E-11 |
| MESDC2 | NM_015154 | -1,17 | 1,30E-11 |
| ANKRD32 | NM_032290 | -1,71 | 1,31E-11 |
| MRPL12 | NM_002949 | -1,33 | 1,31E-11 |
| ANGPT1 | NM_001146 | 2,14 | 1,32E-11 |
| C9orf100 | NM_032818 | -1,24 | 1,32E-11 |
| COPS3 | NM_003653 | -0,97 | 1,35E-11 |
| PTBP1 | NM_175847 | -1,02 | 1,35E-11 |
| C16orf61 | NM_020188 | -1,42 | 1,37E-11 |
| CIR1 | NM_004882 | 1,18 | 1,37E-11 |
| ME2 | NM_002396 | -1,06 | 1,41E-11 |
| MEGF10 | NM_032446 | 1,36 | 1,46E-11 |
| NUP98 | NM_139131 | -1,15 | 1,47E-11 |
| PFDN6 | NM_014260 | -1,52 | 1,47E-11 |
| UBE4B | NM_006048 | 0,82 | 1,47E-11 |
| GGT7 | NM_178026 | 1,11 | 1,48E-11 |
| NOM1 | NM_138400 | -1,29 | 1,50E-11 |
| DAAM2 | NM_015345 | -1,23 | 1,51E-11 |
| PDE4DIP | NM_014644 | 1,08 | 1,51E-11 |
| PLCD4 | NM_032726 | 1,95 | 1,52E-11 |
| BOP1 | NM_015201 | -1,23 | 1,59E-11 |
| GYPC | NM_002101 | 1,06 | 1,62E-11 |
| BTG2 | NM_006763 | 1,34 | 1,63E-11 |
| C1orf112 | NM_018186 | -1,75 | 1,64E-11 |
| FNDC5 | NM_153756 | 3,19 | 1,65E-11 |
| N4BP3 | NM_015111 | -4,05 | 1,65E-11 |
| FKBP14 | NM_017946 | -0,93 | 1,66E-11 |
| ADAMTS2 | NM_014244 | -0,93 | 1,66E-11 |
| C5orf43 | NM_001048249 | -0,91 | 1,73E-11 |
| WDR77 | NM_024102 | -1,36 | 1,74E-11 |
| UGGT1 | NR_027671 | -0,73 | 1,74E-11 |
| FDXR | NM_004110 | 1,63 | 1,75E-11 |
| AIMP2 | NM_006303 | -0,98 | 1,77E-11 |
| LYPLA1 | NM_006330 | -1,05 | 1,78E-11 |
| GNAI3 | NM_006496 | -0,80 | 1,83E-11 |
| ZNF187 | NM_001111039 | 1,34 | 1,86E-11 |
| CAD | NM_004341 | -0,90 | 1,86E-11 |
| TSC22D4 | NM_030935 | 0,81 | 1,87E-11 |
| PGF | NM_002632 | -1,54 | 1,87E-11 |
| RRAGB | NM_006064 | 1,76 | 1,89E-11 |
| ZNF554 | NM_001102651 | 2,12 | 1,91E-11 |
| PSMD11 | NM_002815 | -1,11 | 1,91E-11 |
| TK2 | NM_004614 | 0,99 | 1,93E-11 |
| RAD21 | NM_006265 | -0,79 | 1,99E-11 |
| LOC678655 | NR_015382 | 1,98 | 1,99E-11 |
| HSPA13 | NM_006948 | -0,97 | 2,00E-11 |
| RCBTB2 | NM_001268 | 1,40 | 2,03E-11 |
| EIF4A2 | NM_001967 | 0,90 | 2,09E-11 |
| KCNMB1 | NM_004137 | -2,28 | 2,09E-11 |
| C19orf54 | NM_198476 | 1,23 | 2,09E-11 |
| C8orf55 | NM_016647 | -1,01 | 2,14E-11 |
| PALM2 | NM_001037293 | -2,07 | 2,18E-11 |
| C18orf56 | NM_001012716 | -2,23 | 2,19E-11 |
| C19orf63 | NM_206538 | 0,84 | 2,19E-11 |
| ARMCX2 | NM_177949 | 0,72 | 2,22E-11 |
| EDIL3 | NM_005711 | 1,15 | 2,22E-11 |
| HEATR7A | NM_032450 | 1,34 | 2,27E-11 |
| PPAN-P2RY11 | NM_001040664 | -1,11 | 2,28E-11 |
| ENAH | NM_018212 | -0,77 | 2,28E-11 |
| KCTD12 | NM_138444 | -0,97 | 2,40E-11 |
| NACC1 | NM_052876 | -0,82 | 2,40E-11 |
| CEP170 | NM_001042405 | -1,10 | 2,51E-11 |
| CAMKK1 | NM_032294 | 1,86 | 2,55E-11 |
| RNASE1 | NM_002933 | -4,30 | 2,58E-11 |
| AKR7A2 | NM_003689 | 1,29 | 2,61E-11 |
| METTL11A | NM_014064 | -1,00 | 2,61E-11 |
| IER3 | NM_003897 | -1,09 | 2,63E-11 |
| C9orf25 | NM_147202 | -1,04 | 2,67E-11 |
| PHRF1 | NM_020901 | -0,96 | 2,70E-11 |
| ESYT1 | NM_015292 | -0,80 | 2,72E-11 |
| PLXNA4 | NM_020911 | 1,62 | 2,84E-11 |
| EAPP | NM_018453 | 1,31 | 2,95E-11 |
| EIF2C2 | NM_012154 | -1,04 | 2,96E-11 |
| FOXN3 | NM_005197 | 0,88 | 2,97E-11 |
| SR140 | NM_001080415 | -0,84 | 2,98E-11 |
| ALDOA | NM_000034 | -0,76 | 3,02E-11 |
| TIPIN | NM_017858 | -1,85 | 3,10E-11 |
| DHX58 | NM_024119 | 3,88 | 3,17E-11 |
| SCAMP5 | NM_138967 | 2,24 | 3,19E-11 |
| GLTP | NM_016433 | -1,00 | 3,21E-11 |
| DYNC2H1 | NM_001080463 | 1,39 | 3,26E-11 |
| RBM28 | NM_001166135 | -1,25 | 3,42E-11 |
| SEC14L2 | NM_012429 | 1,12 | 3,43E-11 |
| FLT3LG | NM_001459 | 2,19 | 3,43E-11 |
| HIVEP3 | NM_024503 | -1,97 | 3,43E-11 |
| C10orf2 | NM_001163812 | -1,32 | 3,46E-11 |
| C1orf144 | NM_015609 | -0,76 | 3,46E-11 |
| CXXC5 | NM_016463 | 0,94 | 3,47E-11 |
| MEX3B | NM_032246 | -1,13 | 3,49E-11 |
| NNMT | NM_006169 | -2,84 | 3,53E-11 |
| IPO7 | NM_006391 | -0,84 | 3,54E-11 |
| TMEM92 | NM_153229 | 2,41 | 3,54E-11 |
| KIAA1279 | NM_015634 | -0,93 | 3,58E-11 |
| DDX10 | NM_004398 | -0,93 | 3,66E-11 |
| RPS29 | NM_001030001 | 1,51 | 3,71E-11 |
| BMX | NM_203281 | -4,37 | 3,88E-11 |
| PTMA | NM_001099285 | -0,97 | 3,93E-11 |
| C16orf80 | NM_013242 | -1,10 | 3,94E-11 |
| IGFBP7 | NM_001553 | 0,75 | 3,97E-11 |
| ADIPOR2 | NM_024551 | -0,84 | 3,98E-11 |
| TGFBR1 | NM_004612 | -0,92 | 4,11E-11 |
| LPPR4 | NM_001166252 | -1,21 | 4,13E-11 |
| DR1 | NM_001938 | -0,77 | 4,14E-11 |
| CDC2L6 | NM_015076 | 0,92 | 4,31E-11 |
| MRPL15 | NM_014175 | -1,09 | 4,32E-11 |
| B3GALT2 | NM_003783 | -3,34 | 4,36E-11 |
| MTMR6 | NM_004685 | -0,86 | 4,37E-11 |
| C16orf89 | NM_152459 | 4,01 | 4,41E-11 |
| C20orf24 | NM_199483 | -1,11 | 4,42E-11 |
| KIAA0195 | NM_014738 | 0,84 | 4,48E-11 |
| ZNF449 | NM_152695 | 1,24 | 4,50E-11 |
| HSPA4 | NM_002154 | -0,82 | 4,54E-11 |
| DVL1 | NM_004421 | -0,80 | 4,59E-11 |
| NIPAL3 | NM_020448 | -0,93 | 4,67E-11 |
| LOC387763 | NM_001145033 | 1,59 | 4,67E-11 |
| B4GALNT4 | NM_178537 | 1,66 | 4,75E-11 |
| CRBN | NM_016302 | 1,37 | 4,83E-11 |
| TES | NM_015641 | -0,78 | 4,85E-11 |
| EMG1 | NM_006331 | -1,56 | 5,02E-11 |
| UNG | NM_080911 | -1,23 | 5,03E-11 |
| PYROXD1 | NM_024854 | -1,04 | 5,13E-11 |
| C11orf84 | NM_138471 | -1,08 | 5,14E-11 |
| ILK | NM_001014795 | -0,83 | 5,21E-11 |
| EIF4H | NM_022170 | -0,63 | 5,23E-11 |
| TSPYL2 | NM_022117 | 0,94 | 5,25E-11 |
| GPRASP2 | NM_001004051 | 1,41 | 5,28E-11 |
| TACSTD2 | NM_002353 | -3,66 | 5,41E-11 |
| NDST1 | NM_001543 | -0,81 | 5,44E-11 |
| LOC642846 | NR_024374 | -1,50 | 5,44E-11 |
| ARRDC4 | NM_183376 | 1,28 | 5,56E-11 |
| PCSK9 | NM_174936 | -3,61 | 5,65E-11 |
| B3GNT1 | NM_006876 | 1,03 | 5,78E-11 |
| PVRL3 | NM_015480 | -1,12 | 5,87E-11 |
| EIF3B | NM_001037283 | -1,04 | 5,92E-11 |
| GABRE | NM_004961 | 1,21 | 6,11E-11 |
| SGIP1 | NM_032291 | 1,31 | 6,45E-11 |
| ARHGAP11B | NM_001039841 | -2,31 | 6,48E-11 |
| OLFML1 | NM_198474 | 1,69 | 6,48E-11 |
| POLR1B | NM_001137604 | -1,05 | 6,49E-11 |
| PEX6 | NM_000287 | 1,26 | 6,52E-11 |
| HDAC11 | NM_024827 | 1,35 | 6,54E-11 |
| KCTD7 | NM_153033 | 1,12 | 6,64E-11 |
| CNTNAP1 | NM_003632 | 0,71 | 6,71E-11 |
| PRICKLE2 | NM_198859 | 1,14 | 6,85E-11 |
| MYOF | NM_133337 | -0,72 | 6,90E-11 |
| TMED2 | NM_006815 | -0,71 | 6,92E-11 |
| CACNA1H | NM_001005407 | 0,96 | 7,00E-11 |
| IGFBP1 | NM_000596 | -0,91 | 7,04E-11 |
| SLC36A1 | NM_078483 | 0,86 | 7,04E-11 |
| CHUK | NM_001278 | -1,13 | 7,13E-11 |
| S100A16 | NM_080388 | -0,85 | 7,16E-11 |
| CMIP | NM_030629 | -0,87 | 7,21E-11 |
| LOC388692 | NR_027002 | 2,13 | 7,24E-11 |
| IL1B | NM_000576 | -4,14 | 7,34E-11 |
| NMI | NM_004688 | 1,62 | 7,40E-11 |
| C13orf18 | NM_025113 | 2,90 | 7,43E-11 |
| PARP6 | NM_020214 | 1,13 | 7,43E-11 |
| CHCHD3 | NM_017812 | -0,95 | 7,49E-11 |
| COPS6 | NM_006833 | -0,89 | 7,67E-11 |
| NFKBIL1 | NM_001144962 | 1,31 | 8,01E-11 |
| DNAJC11 | NM_018198 | -0,94 | 8,09E-11 |
| NUCB1 | NM_006184 | 0,65 | 8,15E-11 |
| JARID2 | NM_004973 | 1,00 | 8,18E-11 |
| ATRX | NM_138270 | 0,79 | 8,27E-11 |
| TMX1 | NM_030755 | -0,93 | 8,33E-11 |
| RNF152 | NM_173557 | -1,59 | 8,39E-11 |
| KCTD9 | NM_017634 | -1,07 | 8,44E-11 |
| CCDC15 | NM_025004 | -2,10 | 8,51E-11 |
| SLC25A15 | NM_014252 | -1,25 | 8,62E-11 |
| SOAT1 | NM_003101 | -1,10 | 8,62E-11 |
| GGT5 | NM_004121 | 0,89 | 8,66E-11 |
| TIMP3 | NM_000362 | -0,90 | 8,82E-11 |
| PLA2G15 | NM_012320 | -1,05 | 8,85E-11 |
| RHOA | NM_001664 | -0,66 | 8,97E-11 |
| RBM8A | NM_005105 | -1,12 | 8,97E-11 |
| CENPV | NM_181716 | -1,63 | 9,13E-11 |
| HHLA3 | NM_001031693 | 1,87 | 9,18E-11 |
| NOTCH4 | NM_004557 | -2,32 | 9,45E-11 |
| GARS | NM_002047 | -0,74 | 9,46E-11 |
| APLN | NM_017413 | -3,27 | 9,50E-11 |
| PLXNB1 | NM_001130082 | 1,05 | 9,51E-11 |
| NBEA | NM_015678 | 2,30 | 9,58E-11 |
| RABGGTB | NM_004582 | -1,17 | 9,58E-11 |
| LAPTM5 | NM_006762 | -4,58 | 9,65E-11 |
| NPEPPS | NM_006310 | 0,73 | 9,83E-11 |
| C6orf150 | NM_138441 | -1,90 | 9,94E-11 |
| TAF5L | NM_001025247 | -1,22 | 9,94E-11 |
| C21orf34 | NR_027791 | 3,06 | 1,03E-10 |
| IMP3 | NM_018285 | -1,16 | 1,04E-10 |
| LOC654342 | NR_027238 | -1,47 | 1,06E-10 |
| CFB | NM_001710 | 2,58 | 1,06E-10 |
| GPT2 | NM_001142466 | -1,05 | 1,07E-10 |
| RNF24 | NM_007219 | 0,99 | 1,08E-10 |
| C9orf172 | NM_001080482 | 1,77 | 1,08E-10 |
| CENPH | NM_022909 | -1,72 | 1,09E-10 |
| HIC1 | NM_001098202 | 1,60 | 1,11E-10 |
| SOX12 | NM_006943 | 0,87 | 1,11E-10 |
| IL17RC | NM_153461 | 1,33 | 1,12E-10 |
| B3GNT9 | NM_033309 | 1,07 | 1,13E-10 |
| CNOT7 | NM_054026 | -0,94 | 1,16E-10 |
| LOC349114 | NR_026999 | 1,70 | 1,16E-10 |
| DACT3 | NM_145056 | 2,02 | 1,16E-10 |
| C7 | NM_000587 | 4,56 | 1,18E-10 |
| RNF4 | NM_002938 | -0,88 | 1,18E-10 |
| PYCRL | NM_023078 | -1,18 | 1,22E-10 |
| PPM1M | NM_144641 | 1,28 | 1,22E-10 |
| SLC4A2 | NM_003040 | -0,77 | 1,23E-10 |
| C1orf135 | NM_024037 | -2,32 | 1,27E-10 |
| PPP5C | NM_006247 | -0,85 | 1,27E-10 |
| PLEKHA5 | NM_019012 | 1,45 | 1,30E-10 |
| HOOK2 | NM_013312 | 1,64 | 1,30E-10 |
| DNAJA3 | NM_001135110 | -0,79 | 1,30E-10 |
| IPO11 | NM_016338 | -1,12 | 1,30E-10 |
| SLC25A37 | NM_016612 | 1,05 | 1,30E-10 |
| SCN2A | NM_021007 | 2,25 | 1,30E-10 |
| CDC42EP5 | NM_145057 | 1,76 | 1,31E-10 |
| SSBP4 | NM_001009998 | 1,06 | 1,31E-10 |
| CUGBP2 | NM_001083591 | -1,17 | 1,32E-10 |
| C11orf49 | NM_001003676 | 0,98 | 1,33E-10 |
| KCNE4 | NM_080671 | 1,03 | 1,38E-10 |
| CNN2 | NM_004368 | -0,98 | 1,44E-10 |
| PDE1C | NM_005020 | -1,06 | 1,48E-10 |
| MARCH9 | NM_138396 | 1,41 | 1,48E-10 |
| SOX7 | NM_031439 | -2,62 | 1,49E-10 |
| ZNF33B | NM_006955 | 1,45 | 1,52E-10 |
| FER1L4 | NR_024377 | 1,65 | 1,53E-10 |
| TMEM178 | NM_152390 | 3,97 | 1,53E-10 |
| LPCAT4 | NM_153613 | -0,89 | 1,58E-10 |
| RFFL | NM_057178 | 1,03 | 1,62E-10 |
| UNC13D | NM_199242 | 3,25 | 1,62E-10 |
| LARS | NM_020117 | -0,73 | 1,64E-10 |
| OIP5 | NM_007280 | -2,10 | 1,66E-10 |
| CBX5 | NM_012117 | -0,93 | 1,67E-10 |
| WWC1 | NM_001161661 | -2,33 | 1,68E-10 |
| USP30 | NM_032663 | 1,39 | 1,68E-10 |
| DSN1 | NM_024918 | -1,34 | 1,69E-10 |
| TBC1D2 | NM_018421 | -1,15 | 1,70E-10 |
| ITIH4 | NM_002218 | 3,77 | 1,70E-10 |
| MSTP9 | NR_002729 | 3,48 | 1,71E-10 |
| PGCP | NM_016134 | 0,92 | 1,71E-10 |
| B4GALT2 | NM_001005417 | -0,84 | 1,74E-10 |
| LIMS2 | NR_027823 | -0,93 | 1,75E-10 |
| TTL | NM_153712 | -0,84 | 1,81E-10 |
| KLF9 | NM_001206 | 1,27 | 1,81E-10 |
| SERINC1 | NM_020755 | 0,69 | 1,83E-10 |
| GPR126 | NM_020455 | -2,17 | 1,85E-10 |
| L3MBTL | NM_032107 | 2,27 | 1,85E-10 |
| SREBF2 | NM_004599 | -0,76 | 1,85E-10 |
| SHROOM3 | NM_020859 | -1,38 | 1,86E-10 |
| SCFD2 | NM_152540 | -1,32 | 1,89E-10 |
| ERH | NM_004450 | -0,92 | 1,91E-10 |
| JAK3 | NM_000215 | 2,35 | 1,92E-10 |
| PMM2 | NM_000303 | -0,90 | 1,96E-10 |
| FAM193B | NR_024019 | 0,84 | 2,00E-10 |
| ITGB8 | NM_002214 | 3,34 | 2,00E-10 |
| RNASEH1 | NM_002936 | -1,14 | 2,01E-10 |
| COBLL1 | NM_014900 | -1,26 | 2,02E-10 |
| VPS4A | NM_013245 | -0,72 | 2,02E-10 |
| EFTUD2 | NM_001142605 | -0,71 | 2,03E-10 |
| LOC84740 | NR_026892 | -1,55 | 2,03E-10 |
| WDR48 | NM_020839 | 0,86 | 2,03E-10 |
| GEMIN5 | NM_015465 | -0,99 | 2,08E-10 |
| CLPP | NM_006012 | -0,95 | 2,09E-10 |
| FXN | NM_181425 | -1,49 | 2,12E-10 |
| ZNF302 | NM_018443 | 1,22 | 2,13E-10 |
| KIAA0556 | NM_015202 | 1,14 | 2,13E-10 |
| CCDC120 | NM_033626 | 1,21 | 2,15E-10 |
| DDX51 | NM_175066 | -0,89 | 2,16E-10 |
| SDC3 | NM_014654 | 0,98 | 2,26E-10 |
| SIDT2 | NM_001040455 | 1,19 | 2,26E-10 |
| ADRB2 | NM_000024 | -2,44 | 2,27E-10 |
| UHMK1 | NM_175866 | -1,27 | 2,29E-10 |
| THSD4 | NM_024817 | -1,12 | 2,31E-10 |
| NR4A3 | NM_006981 | 2,49 | 2,31E-10 |
| STAT6 | NM_003153 | 0,65 | 2,36E-10 |
| SFRP1 | NM_003012 | -1,63 | 2,37E-10 |
| LRP12 | NM_001135703 | -0,95 | 2,39E-10 |
| SNRPC | NM_003093 | -1,02 | 2,40E-10 |
| ATAD3B | NM_031921 | -1,40 | 2,46E-10 |
| NUP43 | NM_198887 | -0,91 | 2,46E-10 |
| STON1 | NM_006873 | 1,50 | 2,52E-10 |
| ABCD1 | NM_000033 | 1,18 | 2,54E-10 |
| C5orf34 | NM_198566 | -2,03 | 2,55E-10 |
| NT5DC2 | NM_001134231 | -0,75 | 2,56E-10 |
| LARP1B | NM_032239 | -1,42 | 2,56E-10 |
| STX16 | NM_001134772 | 0,70 | 2,57E-10 |
| GJD3 | NM_152219 | 1,71 | 2,64E-10 |
| CELSR2 | NM_001408 | 1,18 | 2,66E-10 |
| DNM1L | NM_012063 | -0,77 | 2,68E-10 |
| REEP2 | NM_016606 | 1,35 | 2,70E-10 |
| GSTM4 | NR_024538 | 1,27 | 2,71E-10 |
| FOXL1 | NM_005250 | -0,82 | 2,72E-10 |
| NPAS2 | NM_002518 | 1,52 | 2,74E-10 |
| MSH6 | NM_000179 | -0,89 | 2,74E-10 |
| RNPC3 | NM_017619 | 1,59 | 2,74E-10 |
| DHX16 | NM_003587 | -0,84 | 2,77E-10 |
| IDH1 | NM_005896 | -1,01 | 2,79E-10 |
| SOCS5 | NM_014011 | 0,76 | 2,80E-10 |
| KY | NM_178554 | 2,78 | 2,83E-10 |
| PLSCR4 | NM_020353 | 1,48 | 2,97E-10 |
| RNF112 | NM_007148 | 3,74 | 2,97E-10 |
| YPEL4 | NM_145008 | 3,89 | 3,03E-10 |
| CLASP2 | NM_015097 | -0,85 | 3,04E-10 |
| TIAF1 | NM_004740 | 1,45 | 3,07E-10 |
| AK5 | NM_174858 | -1,65 | 3,11E-10 |
| CCDC102A | NM_033212 | 1,62 | 3,17E-10 |
| UBE2V2 | NM_003350 | -0,91 | 3,19E-10 |
| PDE3A | NM_000921 | -0,76 | 3,23E-10 |
| GPD2 | NM_000408 | -0,87 | 3,28E-10 |
| LSM4 | NM_012321 | -0,93 | 3,33E-10 |
| GPR180 | NM_180989 | -1,21 | 3,34E-10 |
| FAM36A | NM_198076 | -0,92 | 3,38E-10 |
| ZNF528 | NM_032423 | 1,66 | 3,39E-10 |
| PCYT2 | NM_002861 | -1,03 | 3,39E-10 |
| TSPAN15 | NM_012339 | -3,43 | 3,40E-10 |
| WDR74 | NM_018093 | -1,18 | 3,41E-10 |
| TMEM11 | NR_024547 | -1,05 | 3,42E-10 |
| PLEKHA1 | NM_001001974 | 1,08 | 3,42E-10 |
| ZMYM5 | NM_001142684 | 1,52 | 3,42E-10 |
| FBXO25 | NM_183420 | 1,43 | 3,43E-10 |
| EIF5 | NM_001969 | -0,65 | 3,46E-10 |
| STOM | NM_004099 | -0,96 | 3,50E-10 |
| ELOVL1 | NM_022821 | -1,23 | 3,51E-10 |
| TSSK3 | NM_052841 | 2,28 | 3,55E-10 |
| HMBS | NM_000190 | -1,51 | 3,56E-10 |
| PUF60 | NM_001136033 | -0,81 | 3,56E-10 |
| ZCCHC14 | NM_015144 | 0,86 | 3,63E-10 |
| ASAH1 | NM_001127505 | 0,90 | 3,72E-10 |
| FTSJ2 | NM_013393 | -1,08 | 3,78E-10 |
| ALPK1 | NM_001102406 | 2,20 | 3,82E-10 |
| PPME1 | NM_016147 | -0,89 | 3,83E-10 |
| ODF2 | NM_002540 | -0,75 | 3,86E-10 |
| C2orf68 | NM_001013649 | 1,04 | 3,86E-10 |
| SAMD14 | NM_174920 | 1,84 | 3,87E-10 |
| TMCO7 | NM_024562 | -1,35 | 3,89E-10 |
| EI24 | NM_004879 | -0,69 | 3,91E-10 |
| VPS11 | NM_021729 | 1,10 | 3,92E-10 |
| XPO5 | NM_020750 | -0,89 | 3,94E-10 |
| CHD3 | NM_005852 | 0,69 | 3,95E-10 |
| C9orf125 | NM_032342 | 0,97 | 3,97E-10 |
| LSS | NM_001001438 | -1,04 | 3,98E-10 |
| SDF2L1 | NM_022044 | -1,65 | 3,99E-10 |
| EPDR1 | NM_017549 | -1,13 | 3,99E-10 |
| C21orf7 | NM_020152 | -1,51 | 4,06E-10 |
| FEM1A | NM_018708 | -0,75 | 4,06E-10 |
| CYTIP | NM_004288 | 3,60 | 4,13E-10 |
| NRBP2 | NM_178564 | 0,92 | 4,17E-10 |
| ZNF358 | NM_018083 | 1,04 | 4,24E-10 |
| WDR12 | NM_018256 | -1,02 | 4,36E-10 |
| YIF1B | NM_033557 | -0,92 | 4,55E-10 |
| MRPL35 | NM_016622 | -0,94 | 4,56E-10 |
| HNRNPK | NM_031262 | -0,77 | 4,57E-10 |
| SAP30L | NM_001131063 | 0,94 | 4,58E-10 |
| ABR | NM_001159746 | 0,64 | 4,64E-10 |
| KLHL18 | NM_025010 | -0,91 | 4,67E-10 |
| TBC1D8 | NM_001102426 | 1,34 | 4,69E-10 |
| NGF | NM_002506 | -2,32 | 4,70E-10 |
| NDEL1 | NM_030808 | -0,90 | 4,70E-10 |
| ELAC2 | NM_018127 | -0,79 | 4,71E-10 |
| TRAPPC6A | NM_024108 | 2,39 | 4,91E-10 |
| FAM72B | NM_001100910 | -2,01 | 5,07E-10 |
| PIP4K2C | NM_001146258 | 1,18 | 5,13E-10 |
| ADAMTS5 | NM_007038 | -1,27 | 5,15E-10 |
| C12orf30 | NM_024953 | -1,17 | 5,17E-10 |
| NHP2 | NM_017838 | -1,12 | 5,24E-10 |
| FADD | NM_003824 | -0,92 | 5,30E-10 |
| LOC729678 | NR_027183 | 1,08 | 5,31E-10 |
| SPATA6 | NM_019073 | 2,22 | 5,31E-10 |
| HSD17B14 | NM_016246 | 2,21 | 5,37E-10 |
| UBXN2B | NM_001077619 | 1,50 | 5,38E-10 |
| CHCHD8 | NM_016565 | -1,09 | 5,42E-10 |
| PABPC1 | NM_002568 | 0,67 | 5,42E-10 |
| LAPTM4A | NM_014713 | 0,84 | 5,45E-10 |
| FLJ42875 | NR_015440 | 2,94 | 5,57E-10 |
| OGFRL1 | NM_024576 | -1,98 | 5,62E-10 |
| TTBK2 | NM_173500 | 1,60 | 5,63E-10 |
| FUCA2 | NM_032020 | -0,79 | 5,69E-10 |
| PIGA | NM_002641 | -1,48 | 5,84E-10 |
| LRRC8D | NM_018103 | -1,10 | 5,89E-10 |
| RRM2B | NM_015713 | 1,01 | 5,89E-10 |
| ZNF828 | NM_001164145 | -0,81 | 5,93E-10 |
| TRIM41 | NM_033549 | 0,88 | 5,99E-10 |
| CD164 | NM_001142404 | 0,64 | 6,08E-10 |
| ZNF277 | NM_021994 | 1,46 | 6,09E-10 |
| SH2B1 | NM_015503 | 0,97 | 6,12E-10 |
| FRAT1 | NM_005479 | 2,88 | 6,13E-10 |
| TRAK1 | NM_014965 | -0,79 | 6,22E-10 |
| CCT8 | NM_006585 | -1,05 | 6,25E-10 |
| SLC46A3 | NM_181785 | 2,30 | 6,37E-10 |
| MDGA1 | NM_153487 | 1,30 | 6,41E-10 |
| LOC338799 | NR_002809 | 1,52 | 6,42E-10 |
| GCNT1 | NM_001097636 | -1,85 | 6,44E-10 |
| C9orf9 | NM_018956 | 2,20 | 6,61E-10 |
| RNGTT | NM_003800 | -1,10 | 6,61E-10 |
| TSC1 | NM_001162427 | 0,89 | 6,70E-10 |
| POFUT1 | NM_015352 | -0,73 | 6,70E-10 |
| KIAA1107 | NM_015237 | 2,84 | 6,70E-10 |
| SH3YL1 | NM_015677 | 1,53 | 6,73E-10 |
| AGPS | NM_003659 | -0,91 | 6,78E-10 |
| C9orf130 | NR_023390 | 1,62 | 6,80E-10 |
| GRK6 | NM_002082 | -1,05 | 6,81E-10 |
| PLD2 | NM_002663 | 0,98 | 6,84E-10 |
| ZP1 | NM_207341 | 2,71 | 6,89E-10 |
| PIP4K2A | NM_005028 | -0,85 | 6,90E-10 |
| EPB41L5 | NM_020909 | 1,09 | 6,95E-10 |
| SLC14A1 | NM_001128588 | 4,68 | 6,95E-10 |
| UTP14A | NM_006649 | -0,96 | 7,03E-10 |
| C14orf106 | NM_018353 | -1,18 | 7,08E-10 |
| DNAJB1 | NM_006145 | -0,74 | 7,26E-10 |
| TTYH2 | NM_032646 | 2,47 | 7,30E-10 |
| SNRPD3 | NM_004175 | -0,97 | 7,48E-10 |
| HNRNPM | NM_031203 | -1,08 | 7,53E-10 |
| SOX5 | NM_006940 | 3,72 | 7,83E-10 |
| NUDT4 | NM_019094 | 0,71 | 7,97E-10 |
| SLC24A1 | NM_004727 | 1,35 | 8,02E-10 |
| C9orf89 | NM_032310 | 1,03 | 8,04E-10 |
| KIAA0831 | NM_014924 | 1,05 | 8,06E-10 |
| DRP2 | NM_001939 | 2,05 | 8,14E-10 |
| ABCC3 | NM_003786 | 2,47 | 8,15E-10 |
| TMEM150A | NM_001031738 | 1,20 | 8,17E-10 |
| CDCA7L | NM_001127370 | -1,07 | 8,18E-10 |
| KLRK1 | NM_007360 | 4,38 | 8,20E-10 |
| CCNE1 | NM_057182 | -1,85 | 8,29E-10 |
| FUCA1 | NM_000147 | 1,49 | 8,29E-10 |
| FAM54A | NM_001099286 | -2,31 | 8,34E-10 |
| BDKRB2 | NM_000623 | 3,92 | 8,45E-10 |
| MAFB | NM_005461 | 2,33 | 8,53E-10 |
| KLC3 | NM_177417 | -2,13 | 8,55E-10 |
| LMTK3 | NM_001080434 | 2,98 | 8,63E-10 |
| PIGV | NM_017837 | 1,55 | 8,72E-10 |
| TSHZ1 | NM_005786 | 0,96 | 8,73E-10 |
| UBE2I | NM_194259 | -0,76 | 8,77E-10 |
| MYL5 | NM_002477 | 2,10 | 8,84E-10 |
| CARS2 | NM_024537 | -1,01 | 8,92E-10 |
| NRK | NM_198465 | -2,15 | 8,98E-10 |
| ABCC5 | NM_005688 | 1,01 | 9,03E-10 |
| USP19 | NM_006677 | 0,81 | 9,10E-10 |
| PLEKHH2 | NM_172069 | 2,06 | 9,17E-10 |
| WWC2 | NM_024949 | -0,86 | 9,21E-10 |
| PSMB3 | NM_002795 | -0,86 | 9,45E-10 |
| TPP1 | NM_000391 | 1,31 | 9,46E-10 |
| SPPL2B | NM_001077238 | 0,89 | 9,55E-10 |
| RUSC2 | NM_014806 | -0,88 | 9,61E-10 |
| FOXN2 | NM_002158 | -1,46 | 9,73E-10 |
| SLC35F2 | NM_017515 | -1,79 | 9,77E-10 |
| AHCYL2 | NM_001130720 | 1,24 | 9,94E-10 |
| RHPN2 | NM_033103 | -1,63 | 1,00E-09 |
| TNKS | NM_003747 | 0,96 | 1,02E-09 |
| HSPA1A | NM_005345 | -0,95 | 1,02E-09 |
| AMY2B | NM_020978 | 2,58 | 1,02E-09 |
| RFNG | NM_002917 | 0,86 | 1,03E-09 |
| FOXP2 | NM_148898 | 2,15 | 1,03E-09 |
| TMEM47 | NM_031442 | -0,80 | 1,05E-09 |
| CDC27 | NM_001256 | -0,85 | 1,05E-09 |
| NUDT4P1 | NR_002212 | 0,71 | 1,05E-09 |
| RDH5 | NM_002905 | 1,59 | 1,08E-09 |
| MRVI1 | NM_001098579 | 1,23 | 1,08E-09 |
| PPP3CC | NM_005605 | -1,00 | 1,10E-09 |
| KIAA0586 | NM_014749 | -1,14 | 1,13E-09 |
| CDH6 | NM_004932 | 1,06 | 1,13E-09 |
| SASS6 | NM_194292 | -1,55 | 1,14E-09 |
| ZNF76 | NM_003427 | 1,10 | 1,18E-09 |
| DOPEY2 | NM_005128 | -1,09 | 1,18E-09 |
| RXRB | NM_021976 | 0,82 | 1,19E-09 |
| FAM175A | NM_139076 | 1,07 | 1,19E-09 |
| P4HTM | NM_177938 | 1,32 | 1,20E-09 |
| POLD3 | NM_006591 | -1,29 | 1,21E-09 |
| SELS | NM_018445 | -1,04 | 1,22E-09 |
| CD82 | NM_002231 | 1,23 | 1,22E-09 |
| FAT2 | NM_001447 | 2,54 | 1,22E-09 |
| CAB39L | NM_030925 | -1,10 | 1,22E-09 |
| SEC61G | NM_014302 | -1,00 | 1,23E-09 |
| TMEM194A | NM_015257 | -0,98 | 1,25E-09 |
| PML | NM_033238 | 0,87 | 1,25E-09 |
| HRAS | NM_005343 | -1,31 | 1,26E-09 |
| EDEM1 | NM_014674 | -0,72 | 1,26E-09 |
| TBC1D3B | NM_001001417 | 1,98 | 1,27E-09 |
| RNF208 | NM_031297 | 2,30 | 1,28E-09 |
| MSH2 | NM_000251 | -0,95 | 1,28E-09 |
| HPGD | NR_027332 | 3,89 | 1,29E-09 |
| SNX22 | NM_024798 | -0,64 | 1,31E-09 |
| MARS | NM_004990 | -0,82 | 1,31E-09 |
| GLRX3 | NM_006541 | -0,89 | 1,32E-09 |
| CHAC2 | NM_001008708 | -2,70 | 1,32E-09 |
| FBL | NM_001436 | -1,05 | 1,37E-09 |
| CCDC154 | NM_001143980 | 3,93 | 1,38E-09 |
| POLR3A | NM_007055 | -0,80 | 1,43E-09 |
| VAMP2 | NM_014232 | 0,98 | 1,43E-09 |
| POLG | NM_001126131 | -0,71 | 1,43E-09 |
| FAM155A | NM_001080396 | 1,16 | 1,48E-09 |
| RAB11B | NM_004218 | 0,75 | 1,48E-09 |
| POLE2 | NM_002692 | -2,49 | 1,50E-09 |
| MEF2A | NM_001130926 | 0,86 | 1,51E-09 |
| CDH11 | NM_001797 | 0,63 | 1,54E-09 |
| LATS2 | NM_014572 | -0,90 | 1,54E-09 |
| ATM | NM_138292 | 1,07 | 1,55E-09 |
| ZMYM2 | NM_197968 | 0,80 | 1,56E-09 |
| TIA1 | NM_022173 | 0,78 | 1,59E-09 |
| TNFAIP8 | NM_014350 | 1,02 | 1,59E-09 |
| C9orf40 | NM_017998 | -1,38 | 1,60E-09 |
| CITED4 | NM_133467 | 1,84 | 1,63E-09 |
| MFHAS1 | NM_004225 | -1,07 | 1,65E-09 |
| C17orf56 | NM_144679 | 0,97 | 1,70E-09 |
| COL5A3 | NM_015719 | 1,06 | 1,71E-09 |
| MYCBP | NM_012333 | -1,25 | 1,72E-09 |
| ZC3H6 | NM_198581 | 1,72 | 1,74E-09 |
| SYT11 | NM_152280 | 0,76 | 1,74E-09 |
| PGAM5 | NM_138575 | -1,59 | 1,76E-09 |
| DDX52 | NM_152300 | -0,96 | 1,79E-09 |
| KIAA0892 | NM_015329 | 0,78 | 1,80E-09 |
| ALDH5A1 | NM_001080 | 1,22 | 1,84E-09 |
| SPATA18 | NM_145263 | 2,09 | 1,85E-09 |
| C22orf46 | NM_001142964 | 1,08 | 1,87E-09 |
| RCC2 | NM_001136204 | -0,73 | 1,89E-09 |
| ZNF181 | NM_001145665 | 1,55 | 1,93E-09 |
| ARID4A | NM_023000 | 1,25 | 1,96E-09 |
| BBS9 | NM_198428 | 1,25 | 2,02E-09 |
| SLC35B1 | NM_005827 | -0,94 | 2,04E-09 |
| APBB1 | NM_145689 | 0,93 | 2,09E-09 |
| ERMP1 | NM_024896 | 1,14 | 2,13E-09 |
| H2AFV | NM_012412 | -0,85 | 2,15E-09 |
| SEMA6A | NM_020796 | 2,87 | 2,15E-09 |
| GLTSCR2 | NM_015710 | 0,84 | 2,21E-09 |
| C11orf24 | NM_022338 | -0,98 | 2,22E-09 |
| CASC4 | NM_138423 | 0,67 | 2,23E-09 |
| TRIM4 | NM_033017 | 0,93 | 2,23E-09 |
| SF3B1 | NM_012433 | 0,61 | 2,24E-09 |
| CCDC58 | NM_001017928 | -1,72 | 2,27E-09 |
| NIF3L1 | NM_021824 | -1,12 | 2,27E-09 |
| GUCY1B3 | NM_000857 | 1,35 | 2,27E-09 |
| WDR78 | NM_024763 | 2,58 | 2,32E-09 |
| KLHDC2 | NM_014315 | 1,02 | 2,32E-09 |
| DISC1 | NM_001164540 | 1,50 | 2,35E-09 |
| MORF4L2 | NM_001142424 | -0,66 | 2,36E-09 |
| SLC38A2 | NM_018976 | 0,69 | 2,37E-09 |
| RPS6KA4 | NM_003942 | -0,99 | 2,37E-09 |
| FAM196B | NM_001129891 | -1,69 | 2,39E-09 |
| SMARCA2 | NM_003070 | 0,88 | 2,42E-09 |
| YBX1 | NM_004559 | -0,76 | 2,42E-09 |
| TBX15 | NM_152380 | 1,40 | 2,44E-09 |
| MYCBP2 | NM_015057 | 1,08 | 2,45E-09 |
| NOTCH1 | NM_017617 | 0,72 | 2,48E-09 |
| SNAI1 | NM_005985 | 1,47 | 2,49E-09 |
| CNRIP1 | NM_015463 | -0,92 | 2,49E-09 |
| HYAL2 | NM_033158 | -1,03 | 2,52E-09 |
| RAB36 | NM_004914 | 1,19 | 2,53E-09 |
| CDC37 | NM_007065 | -0,68 | 2,53E-09 |
| PIN1 | NM_006221 | -0,83 | 2,55E-09 |
| NHP2L1 | NM_005008 | -0,80 | 2,56E-09 |
| SEMA5B | NM_001031702 | 3,12 | 2,59E-09 |
| NIPSNAP1 | NM_003634 | 0,85 | 2,63E-09 |
| EIF2B3 | NM_001166588 | -1,16 | 2,65E-09 |
| NINJ1 | NM_004148 | 1,09 | 2,66E-09 |
| PTGS1 | NM_080591 | 1,01 | 2,67E-09 |
| GEMIN8 | NM_001042479 | 1,44 | 2,71E-09 |
| CELSR1 | NM_014246 | 1,50 | 2,71E-09 |
| STAT3 | NM_213662 | 0,64 | 2,73E-09 |
| TMBIM1 | NM_022152 | -0,71 | 2,75E-09 |
| CC2D1A | NM_017721 | 0,93 | 2,76E-09 |
| B3GALTL | NM_194318 | -1,13 | 2,76E-09 |
| SNPH | NM_014723 | 1,12 | 2,76E-09 |
| GATA2 | NM_032638 | -2,60 | 2,81E-09 |
| EGLN1 | NM_022051 | -0,83 | 2,92E-09 |
| ANKRD40 | NM_052855 | -0,90 | 2,95E-09 |
| ST7 | NM_021908 | -1,36 | 2,96E-09 |
| EBF1 | NM_024007 | 1,03 | 3,01E-09 |
| CDC42 | NM_001791 | -0,63 | 3,02E-09 |
| EGR2 | NM_000399 | 3,02 | 3,04E-09 |
| KLRC3 | NM_002261 | 4,53 | 3,08E-09 |
| C1orf213 | NM_138479 | 2,00 | 3,08E-09 |
| ZNF154 | NM_001085384 | 1,31 | 3,10E-09 |
| FAM160B2 | NM_022749 | 0,72 | 3,14E-09 |
| TCF25 | NM_014972 | 0,81 | 3,17E-09 |
| SETDB1 | NM_012432 | 0,86 | 3,21E-09 |
| SLC16A6 | NM_004694 | 3,31 | 3,22E-09 |
| MTCH2 | NM_014342 | -0,75 | 3,23E-09 |
| ZNF846 | NM_001077624 | 2,25 | 3,27E-09 |
| PARD3 | NM_019619 | 0,88 | 3,32E-09 |
| KIAA0406 | NM_014657 | -0,97 | 3,34E-09 |
| PLCL1 | NM_006226 | 2,54 | 3,39E-09 |
| SMG6 | NM_017575 | 0,75 | 3,40E-09 |
| SCLT1 | NM_144643 | -1,43 | 3,42E-09 |
| ATP6AP2 | NM_005765 | 0,75 | 3,44E-09 |
| SAAL1 | NM_138421 | -1,46 | 3,52E-09 |
| C15orf29 | NM_024713 | -1,32 | 3,52E-09 |
| GPRASP1 | NM_001099410 | 1,75 | 3,52E-09 |
| FUT11 | NM_173540 | -0,88 | 3,61E-09 |
| BAZ1B | NM_032408 | -0,67 | 3,62E-09 |
| TMTC3 | NM_181783 | -0,77 | 3,68E-09 |
| FBXL20 | NM_032875 | 1,40 | 3,74E-09 |
| ISG20L2 | NM_030980 | -0,83 | 3,79E-09 |
| SEMA4G | NM_017893 | 1,50 | 3,85E-09 |
| KPNA3 | NM_002267 | -0,87 | 3,86E-09 |
| C1orf126 | NR_027136 | 2,40 | 3,86E-09 |
| DYNC1LI1 | NM_016141 | -0,78 | 3,95E-09 |
| SLC26A6 | NM_001040454 | 0,99 | 3,96E-09 |
| SORCS2 | NM_020777 | 3,72 | 4,00E-09 |
| SLC2A10 | NM_030777 | 0,93 | 4,03E-09 |
| ZNF853 | NM_017560 | 1,67 | 4,14E-09 |
| NFIX | NM_002501 | 0,86 | 4,14E-09 |
| FUT8 | NM_178154 | 0,91 | 4,16E-09 |
| SAT2 | NM_133491 | 1,38 | 4,30E-09 |
| RPF1 | NM_025065 | -0,96 | 4,37E-09 |
| CFD | NM_001928 | 3,02 | 4,42E-09 |
| MUS81 | NM_025128 | -0,80 | 4,45E-09 |
| WAPAL | NM_015045 | -0,71 | 4,48E-09 |
| MPZL2 | NM_005797 | -3,69 | 4,49E-09 |
| RGMA | NM_001166283 | 2,78 | 4,49E-09 |
| BBS4 | NM_033028 | 1,50 | 4,49E-09 |
| ARMCX3 | NM_177948 | 0,87 | 4,50E-09 |
| QSOX1 | NM_001004128 | -0,90 | 4,55E-09 |
| SNX30 | NM_001012994 | 1,07 | 4,59E-09 |
| ZW10 | NM_004724 | -0,93 | 4,72E-09 |
| FAM83G | NM_001039999 | -1,23 | 4,76E-09 |
| CWF19L1 | NM_018294 | -1,13 | 4,81E-09 |
| RPL39L | NM_052969 | -1,42 | 4,87E-09 |
| DCAF5 | NM_003861 | 0,97 | 5,00E-09 |
| FGD1 | NM_004463 | 0,82 | 5,01E-09 |
| LOC619207 | NR_002934 | 2,67 | 5,02E-09 |
| SLC20A2 | NM_006749 | 0,70 | 5,04E-09 |
| HABP4 | NM_014282 | -1,08 | 5,07E-09 |
| TCF4 | NM_003199 | 1,13 | 5,09E-09 |
| ECSCR | NM_001077693 | -1,20 | 5,14E-09 |
| C14orf159 | NM_001102368 | 1,79 | 5,15E-09 |
| ANKRD9 | NM_152326 | -1,93 | 5,21E-09 |
| HSPB1 | NM_001540 | -0,91 | 5,22E-09 |
| ADAMTS6 | NM_197941 | -1,51 | 5,31E-09 |
| NUS1 | NM_138459 | -0,88 | 5,32E-09 |
| ZFP36L2 | NM_006887 | 0,71 | 5,34E-09 |
| RABGEF1 | NM_014504 | -0,81 | 5,48E-09 |
| WBP11 | NM_016312 | -1,06 | 5,49E-09 |
| MDH2 | NM_005918 | -0,69 | 5,57E-09 |
| CAMLG | NM_001745 | 1,00 | 5,69E-09 |
| MRPL24 | NM_024540 | -1,13 | 5,71E-09 |
| UTP11L | NM_016037 | -1,05 | 5,72E-09 |
| AMMECR1L | NM_031445 | -0,78 | 5,76E-09 |
| PTPRM | NM_002845 | 0,74 | 5,78E-09 |
| KIAA1191 | NM_001079684 | -0,75 | 5,89E-09 |
| PTPRK | NM_002844 | 0,71 | 5,90E-09 |
| ZNF211 | NM_198855 | 1,30 | 5,90E-09 |
| NPR2 | NM_003995 | 0,92 | 5,91E-09 |
| IFIT1 | NM_001548 | 2,25 | 5,94E-09 |
| PHF3 | NM_015153 | 0,73 | 5,95E-09 |
| C1orf85 | NM_144580 | 0,97 | 6,03E-09 |
| SLMO2 | NM_016045 | -0,88 | 6,10E-09 |
| NCOA1 | NM_003743 | 1,37 | 6,13E-09 |
| SEMA4B | NM_020210 | 1,15 | 6,17E-09 |
| FAM117B | NM_173511 | 1,29 | 6,23E-09 |
| FAM171A2 | NM_198475 | 1,10 | 6,24E-09 |
| KDM5D | NM_001146705 | 0,85 | 6,32E-09 |
| LOC646851 | NM_001013647 | 2,17 | 6,43E-09 |
| SGTA | NM_003021 | -0,85 | 6,46E-09 |
| S100A10 | NM_002966 | -0,80 | 6,48E-09 |
| CCDC40 | NM_017950 | 2,22 | 6,63E-09 |
| FYCO1 | NM_024513 | 0,84 | 6,70E-09 |
| WDR34 | NM_052844 | -1,06 | 6,76E-09 |
| CDK5RAP2 | NM_001011649 | -0,78 | 6,76E-09 |
| PLXDC2 | NM_032812 | 4,21 | 6,78E-09 |
| GATAD2B | NM_020699 | 0,80 | 6,79E-09 |
| UBA6 | NM_018227 | -0,72 | 6,84E-09 |
| ATRIP | NM_130384 | -1,35 | 6,86E-09 |
| IWS1 | NM_017969 | -0,77 | 6,88E-09 |
| KCNK15 | NM_022358 | 4,02 | 6,92E-09 |
| C9orf30 | NM_080655 | -0,95 | 6,94E-09 |
| TBX3 | NM_005996 | 1,55 | 6,97E-09 |
| SLC25A13 | NM_001160210 | -1,22 | 7,13E-09 |
| SLCO2A1 | NM_005630 | -2,31 | 7,31E-09 |
| ATP1B2 | NM_001678 | 3,19 | 7,36E-09 |
| SNRPF | NM_003095 | -1,59 | 7,39E-09 |
| PLA2G4A | NM_024420 | 1,31 | 7,51E-09 |
| PICALM | NM_001008660 | -0,83 | 7,55E-09 |
| BTN2A1 | NM_078476 | -0,82 | 7,55E-09 |
| OCEL1 | NM_024578 | 1,61 | 7,55E-09 |
| ANKLE2 | NM_015114 | -0,76 | 7,62E-09 |
| RAB1A | NM_004161 | -0,64 | 7,64E-09 |
| FIBCD1 | NM_032843 | 2,64 | 7,64E-09 |
| ELFN1 | NM_001128636 | -1,94 | 7,74E-09 |
| VIPR1 | NM_004624 | 2,70 | 7,75E-09 |
| RBM6 | NM_005777 | 0,75 | 7,81E-09 |
| MED12 | NM_005120 | 1,07 | 7,82E-09 |
| MCL1 | NM_021960 | -0,71 | 7,86E-09 |
| NHLRC3 | NM_001012754 | 1,33 | 7,90E-09 |
| TBC1D3 | NM_001123391 | 1,81 | 7,95E-09 |
| PRX | NM_020956 | 1,47 | 7,96E-09 |
| C10orf32 | NM_001136200 | 1,45 | 8,02E-09 |
| UFD1L | NM_005659 | -0,92 | 8,06E-09 |
| EEF2K | NM_013302 | 0,67 | 8,07E-09 |
| GTF2E1 | NM_005513 | -1,12 | 8,15E-09 |
| UXS1 | NM_025076 | -0,86 | 8,36E-09 |
| H19 | NR_002196 | -1,35 | 8,38E-09 |
| ATP13A2 | NM_001141974 | -0,81 | 8,47E-09 |
| GSTK1 | NM_001143679 | 0,78 | 8,47E-09 |
| CPT1B | NM_001145135 | 2,58 | 8,49E-09 |
| CLDN15 | NM_014343 | 1,51 | 8,59E-09 |
| ERCC1 | NM_001166049 | -0,64 | 8,66E-09 |
| TMEM219 | NM_001083613 | 0,91 | 8,66E-09 |
| PREX1 | NM_020820 | 0,83 | 8,79E-09 |
| IRF2 | NM_002199 | 1,15 | 8,80E-09 |
| SLC25A23 | NM_024103 | 1,29 | 8,81E-09 |
| ZFYVE28 | NM_020972 | 2,14 | 8,91E-09 |
| BMP6 | NM_001718 | -1,56 | 8,95E-09 |
| PSMD6 | NM_014814 | -0,77 | 9,17E-09 |
| DOLPP1 | NM_020438 | -1,10 | 9,19E-09 |
| C1orf113 | NM_024676 | -1,21 | 9,21E-09 |
| VBP1 | NM_003372 | -0,90 | 9,35E-09 |
| BSCL2 | NM_001130702 | 1,00 | 9,45E-09 |
| ADCY7 | NM_001114 | -1,13 | 9,46E-09 |
| EFNB1 | NM_004429 | 0,91 | 9,46E-09 |
| PLA2G12A | NM_030821 | 0,92 | 9,51E-09 |
| SHE | NM_001010846 | -3,36 | 9,52E-09 |
| HSPA1B | NM_005346 | -0,82 | 9,53E-09 |
| PAK4 | NM_005884 | -0,79 | 9,64E-09 |
| ENPP1 | NM_006208 | -1,00 | 9,67E-09 |
| SLITRK3 | NM_014926 | 2,04 | 9,75E-09 |
| SULT1C2 | NM_001056 | 4,16 | 1,00E-08 |
| FAM21C | NM_015262 | 0,82 | 1,01E-08 |
| DIMT1L | NM_014473 | -1,16 | 1,01E-08 |
| CDKN1B | NM_004064 | 0,77 | 1,01E-08 |
| PGAP2 | NR_027017 | 1,05 | 1,02E-08 |
| AKNA | NM_030767 | 1,02 | 1,03E-08 |
| CLPB | NM_030813 | -1,05 | 1,03E-08 |
| KIAA1109 | NM_015312 | 1,03 | 1,03E-08 |
| BZW2 | NM_014038 | -0,75 | 1,04E-08 |
| OSTF1 | NM_012383 | -1,12 | 1,05E-08 |
| SULT1C4 | NM_006588 | 3,27 | 1,06E-08 |
| KIF24 | NM_194313 | -1,52 | 1,06E-08 |
| MR1 | NM_001531 | 1,61 | 1,06E-08 |
| LOC100133091 | NR_029411 | 1,33 | 1,06E-08 |
| MRPL18 | NM_014161 | -0,96 | 1,07E-08 |
| PARK2 | NM_004562 | 3,79 | 1,09E-08 |
| C22orf36 | NM_207644 | 1,81 | 1,11E-08 |
| DSTN | NM_006870 | -0,62 | 1,11E-08 |
| TIMM10 | NM_012456 | -1,44 | 1,12E-08 |
| NTF3 | NM_001102654 | -1,86 | 1,12E-08 |
| SLC1A3 | NM_004172 | 2,51 | 1,13E-08 |
| PRDX3 | NM_006793 | -0,87 | 1,13E-08 |
| PEX1 | NM_000466 | 1,22 | 1,13E-08 |
| HEXIM2 | NM_144608 | 1,84 | 1,14E-08 |
| POLDIP2 | NM_015584 | -0,71 | 1,15E-08 |
| PSMB8 | NM_004159 | 1,21 | 1,16E-08 |
| DTNA | NM_032979 | -1,37 | 1,18E-08 |
| COLEC12 | NM_130386 | 2,16 | 1,18E-08 |
| ACOX2 | NM_003500 | 2,48 | 1,18E-08 |
| TXNL4A | NM_006701 | -0,83 | 1,18E-08 |
| CPXM1 | NM_019609 | 2,48 | 1,19E-08 |
| ZSWIM4 | NM_023072 | 1,52 | 1,20E-08 |
| REEP6 | NM_138393 | 1,98 | 1,21E-08 |
| MAP7D1 | NM_018067 | -0,93 | 1,23E-08 |
| CLCF1 | NM_001166212 | -1,01 | 1,23E-08 |
| CEND1 | NM_016564 | 1,73 | 1,23E-08 |
| SERPINH1 | NM_001235 | -0,73 | 1,24E-08 |
| SLFN5 | NM_144975 | 0,98 | 1,25E-08 |
| TBC1D3F | NM_032258 | 1,80 | 1,25E-08 |
| RRP1 | NM_003683 | -1,17 | 1,26E-08 |
| CCDC106 | NM_013301 | 1,17 | 1,28E-08 |
| DTX3L | NM_138287 | 0,98 | 1,28E-08 |
| IFITM2 | NM_006435 | 1,17 | 1,28E-08 |
| MAGI2 | NM_012301 | 1,75 | 1,29E-08 |
| SPRYD3 | NM_032840 | -0,84 | 1,29E-08 |
| ARHGEF2 | NM_001162383 | 0,64 | 1,29E-08 |
| SEPHS1 | NM_012247 | -0,78 | 1,29E-08 |
| SESN1 | NM_014454 | 1,40 | 1,31E-08 |
| SLC22A23 | NM_015482 | 1,18 | 1,31E-08 |
| PI4KA | NM_058004 | 0,69 | 1,32E-08 |
| GGCT | NM_024051 | -1,05 | 1,34E-08 |
| KIF13B | NM_015254 | 1,23 | 1,34E-08 |
| CHRFAM7A | NM_148911 | 2,99 | 1,35E-08 |
| CCDC115 | NM_032357 | 1,02 | 1,35E-08 |
| CCDC24 | NM_152499 | 1,95 | 1,36E-08 |
| LOC153684 | NR_015447 | 2,02 | 1,37E-08 |
| CORO6 | NM_032854 | 1,90 | 1,38E-08 |
| SLC31A1 | NM_001859 | -0,76 | 1,39E-08 |
| TSC22D2 | NM_014779 | -0,81 | 1,40E-08 |
| C5orf62 | NM_032947 | 1,46 | 1,41E-08 |
| C17orf96 | NM_001130677 | -1,65 | 1,41E-08 |
| SNX32 | NM_152760 | 4,12 | 1,41E-08 |
| C7orf40 | NR_003697 | -1,55 | 1,42E-08 |
| HNRNPA2B1 | NM_002137 | -0,81 | 1,42E-08 |
| ADAM9 | NM_003816 | -0,63 | 1,44E-08 |
| C5orf33 | NM_153013 | 0,93 | 1,44E-08 |
| UBR7 | NM_175748 | -0,78 | 1,46E-08 |
| TM4SF18 | NM_138786 | -2,76 | 1,48E-08 |
| SHC2 | NM_012435 | 1,90 | 1,48E-08 |
| CLSTN2 | NM_022131 | 1,23 | 1,49E-08 |
| SLC35E2 | NM_182838 | 1,61 | 1,49E-08 |
| FAM124B | NM_024785 | -2,97 | 1,52E-08 |
| DKK2 | NM_014421 | 1,28 | 1,54E-08 |
| DNMT3A | NM_175629 | 0,95 | 1,54E-08 |
| DARS | NM_001349 | -0,77 | 1,57E-08 |
| HEY2 | NM_012259 | 1,47 | 1,57E-08 |
| SPRED2 | NM_181784 | 1,12 | 1,59E-08 |
| RTN2 | NM_206901 | 1,64 | 1,60E-08 |
| SH2B3 | NM_005475 | -0,94 | 1,64E-08 |
| UNC84B | NM_015374 | -0,77 | 1,64E-08 |
| IL4R | NM_000418 | -0,68 | 1,65E-08 |
| C16orf58 | NM_022744 | 0,69 | 1,68E-08 |
| SUSD1 | NM_022486 | -1,26 | 1,73E-08 |
| SIAH2 | NM_005067 | -0,80 | 1,75E-08 |
| TNRC6C | NM_018996 | 1,05 | 1,76E-08 |
| F2RL1 | NM_005242 | 1,36 | 1,77E-08 |
| AGT | NM_000029 | 1,45 | 1,79E-08 |
| PTRH2 | NM_016077 | -1,40 | 1,80E-08 |
| NRP2 | NM_003872 | -1,19 | 1,82E-08 |
| SORBS3 | NM_005775 | -1,03 | 1,83E-08 |
| SLC38A1 | NM_030674 | -0,88 | 1,84E-08 |
| PINX1 | NM_017884 | -1,60 | 1,85E-08 |
| UBN2 | NM_173569 | 1,07 | 1,86E-08 |
| GALNT6 | NM_007210 | -2,82 | 1,86E-08 |
| MCTP1 | NM_001002796 | -2,94 | 1,87E-08 |
| ZNF785 | NM_152458 | 1,32 | 1,88E-08 |
| LIN9 | NM_173083 | -1,66 | 1,90E-08 |
| PDCD5 | NM_004708 | -1,12 | 1,90E-08 |
| TRAIP | NM_005879 | -1,74 | 1,92E-08 |
| SEMA6B | NM_032108 | -1,74 | 1,94E-08 |
| C13orf27 | NM_138779 | -1,74 | 1,97E-08 |
| KAT2B | NM_003884 | 1,16 | 1,98E-08 |
| PROS1 | NM_000313 | 1,60 | 2,00E-08 |
| GSTZ1 | NM_001513 | 1,45 | 2,00E-08 |
| ABCB10 | NM_012089 | -1,03 | 2,03E-08 |
| PPM1G | NM_177983 | -0,98 | 2,06E-08 |
| BAK1 | NM_001188 | -0,96 | 2,07E-08 |
| MT2A | NM_005953 | -0,92 | 2,07E-08 |
| PLA2G4B | NM_001114633 | 1,40 | 2,07E-08 |
| B3GNT2 | NM_006577 | -1,11 | 2,08E-08 |
| ASNS | NM_001673 | -0,71 | 2,09E-08 |
| UPK3B | NM_030570 | 3,88 | 2,10E-08 |
| TBCK | NM_033115 | 1,43 | 2,10E-08 |
| PRR16 | NM_016644 | -1,09 | 2,11E-08 |
| ACTN4 | NM_004924 | -0,87 | 2,11E-08 |
| FXR2 | NM_004860 | -0,68 | 2,12E-08 |
| RAPGEFL1 | NM_016339 | 1,55 | 2,13E-08 |
| CCDC57 | NM_198082 | 0,99 | 2,13E-08 |
| ZNF318 | NM_014345 | 0,74 | 2,14E-08 |
| ATP11C | NM_173694 | -0,75 | 2,14E-08 |
| KLHDC5 | NM_020782 | -0,73 | 2,18E-08 |
| HDHD1A | NM_012080 | -1,14 | 2,25E-08 |
| LZTS1 | NM_021020 | 0,78 | 2,27E-08 |
| RBCK1 | NM_031229 | 0,75 | 2,28E-08 |
| PBRM1 | NM_181042 | -0,63 | 2,29E-08 |
| RABEPK | NM_005833 | -0,97 | 2,29E-08 |
| TM7SF2 | NM_003273 | 1,98 | 2,32E-08 |
| DYRK2 | NM_006482 | -0,73 | 2,33E-08 |
| SRPR | NM_003139 | -0,66 | 2,34E-08 |
| THAP3 | NM_138350 | -0,79 | 2,35E-08 |
| CRKRS | NM_015083 | -0,80 | 2,36E-08 |
| DLK1 | NM_003836 | 3,72 | 2,39E-08 |
| MLLT3 | NM_004529 | 1,47 | 2,42E-08 |
| ADAM17 | NM_003183 | 0,80 | 2,47E-08 |
| TMED7-TICAM2 | NM_001164469 | -0,90 | 2,47E-08 |
| PLEKHM3 | NM_001080475 | 1,06 | 2,48E-08 |
| PSME4 | NM_014614 | -0,66 | 2,49E-08 |
| AKR7A3 | NM_012067 | 2,23 | 2,49E-08 |
| DEDD2 | NM_133328 | 1,01 | 2,51E-08 |
| MOBKL2C | NM_145279 | 1,38 | 2,56E-08 |
| MAP1LC3A | NM_032514 | 1,11 | 2,57E-08 |
| ADK | NM_006721 | -0,88 | 2,58E-08 |
| BID | NM_001196 | -1,06 | 2,62E-08 |
| GNG5 | NM_005274 | -0,74 | 2,68E-08 |
| ENTPD6 | NM_001247 | -0,73 | 2,70E-08 |
| HIST1H2AC | NM_003512 | 2,38 | 2,70E-08 |
| TMEM159 | NM_020422 | 1,26 | 2,71E-08 |
| RAB35 | NM_006861 | -0,68 | 2,71E-08 |
| DPH2 | NM_001384 | -0,96 | 2,74E-08 |
| AGXT2L2 | NM_153373 | 1,33 | 2,75E-08 |
| NEK8 | NM_178170 | 2,11 | 2,77E-08 |
| PPIB | NM_000942 | -0,66 | 2,78E-08 |
| EIF4EBP3 | NM_003732 | 1,77 | 2,78E-08 |
| VDAC3 | NM_001135694 | -0,92 | 2,79E-08 |
| LCTL | NM_207338 | -1,90 | 2,82E-08 |
| ZSCAN18 | NM_001145544 | 1,27 | 2,86E-08 |
| LCNL1 | NM_207510 | 4,05 | 2,92E-08 |
| PSMA7 | NM_002792 | -0,99 | 2,95E-08 |
| CLTB | NM_007097 | -0,84 | 2,97E-08 |
| PIK3R3 | NM_003629 | 0,99 | 2,98E-08 |
| MIF | NM_002415 | -0,78 | 2,99E-08 |
| VCAM1 | NM_001078 | 3,03 | 2,99E-08 |
| TATDN2 | NM_014760 | -0,67 | 3,00E-08 |
| FAM118A | NM_017911 | 0,87 | 3,03E-08 |
| C4orf43 | NM_018352 | -1,04 | 3,05E-08 |
| VAMP5 | NM_006634 | 1,28 | 3,05E-08 |
| IER3IP1 | NM_016097 | -0,87 | 3,06E-08 |
| ZNF575 | NM_174945 | 1,91 | 3,09E-08 |
| DCAF13 | NM_015420 | -1,02 | 3,11E-08 |
| TXNDC9 | NM_005783 | -1,02 | 3,20E-08 |
| SWAP70 | NM_015055 | -0,76 | 3,22E-08 |
| RICS | NM_001142685 | 1,17 | 3,23E-08 |
| FZD8 | NM_031866 | 1,19 | 3,25E-08 |
| ZNF517 | NM_213605 | 1,78 | 3,26E-08 |
| MARK4 | NM_031417 | 0,76 | 3,28E-08 |
| C10orf57 | NM_025125 | 1,09 | 3,34E-08 |
| PPP2R1B | NM_002716 | -0,99 | 3,36E-08 |
| DDX56 | NM_019082 | -0,74 | 3,37E-08 |
| TXNDC16 | NM_020784 | 1,26 | 3,38E-08 |
| STON1-GTF2A1L | NM_172311 | 1,56 | 3,39E-08 |
| RAD23A | NM_005053 | -0,62 | 3,49E-08 |
| SYS1-DBNDD2 | NR_003189 | -1,05 | 3,51E-08 |
| SLC38A5 | NM_033518 | -2,46 | 3,51E-08 |
| SEMA6C | NM_030913 | 1,27 | 3,53E-08 |
| CCDC18 | NM_206886 | -1,32 | 3,54E-08 |
| YME1L1 | NM_014263 | -0,60 | 3,61E-08 |
| DDB2 | NM_000107 | 0,97 | 3,62E-08 |
| C14orf93 | NM_021944 | 1,34 | 3,72E-08 |
| ACO1 | NM_002197 | -0,67 | 3,74E-08 |
| ACSS1 | NM_032501 | 1,93 | 3,75E-08 |
| SET | NM_001122821 | -0,77 | 3,77E-08 |
| ATAD3C | NM_001039211 | -1,75 | 3,78E-08 |
| C17orf51 | NM_001113434 | -0,74 | 3,81E-08 |
| ARF1 | NM_001024227 | -0,68 | 3,85E-08 |
| ZNF667 | NM_022103 | 1,61 | 3,86E-08 |
| LOC728448 | NR_003929 | 2,49 | 3,86E-08 |
| C12orf24 | NM_013300 | -1,37 | 3,89E-08 |
| CSRP2 | NM_001321 | -1,46 | 3,95E-08 |
| ABCA4 | NM_000350 | 2,46 | 3,97E-08 |
| NR4A1 | NM_002135 | -1,51 | 3,99E-08 |
| SENP7 | NM_001077203 | 1,09 | 3,99E-08 |
| KPNA1 | NR_026698 | -0,60 | 4,00E-08 |
| ZNF219 | NM_001101672 | 1,07 | 4,02E-08 |
| SEMA3E | NM_012431 | 1,96 | 4,07E-08 |
| PGM3 | NM_015599 | -0,78 | 4,07E-08 |
| MBD5 | NM_018328 | 1,58 | 4,07E-08 |
| GTF2I | NM_001518 | 0,62 | 4,08E-08 |
| TSPAN7 | NM_004615 | 2,13 | 4,13E-08 |
| PERP | NM_022121 | 0,81 | 4,14E-08 |
| APEX2 | NM_014481 | -0,96 | 4,18E-08 |
| CD302 | NM_014880 | 1,22 | 4,22E-08 |
| SF3A3 | NM_006802 | -0,73 | 4,22E-08 |
| GCOM1 | NM_001018091 | 0,93 | 4,28E-08 |
| PRIM1 | NM_000946 | -1,64 | 4,28E-08 |
| C17orf108 | NM_001076680 | 2,48 | 4,30E-08 |
| HSD17B7 | NM_016371 | -1,86 | 4,33E-08 |
| METTL1 | NM_005371 | -1,11 | 4,35E-08 |
| ZAK | NM_133646 | -0,72 | 4,37E-08 |
| CCBL2 | NM_001008661 | 0,96 | 4,43E-08 |
| ETNK2 | NM_018208 | 1,39 | 4,47E-08 |
| FOXO3 | NM_201559 | 0,68 | 4,49E-08 |
| F8 | NM_000132 | 2,66 | 4,50E-08 |
| TRAPPC9 | NM_001160372 | 0,92 | 4,53E-08 |
| NPHP3 | NM_153240 | 0,86 | 4,53E-08 |
| DCAF11 | NM_181357 | 0,82 | 4,54E-08 |
| TAX1BP3 | NM_014604 | -0,85 | 4,54E-08 |
| KIAA1614 | NM_020950 | 1,47 | 4,54E-08 |
| MYB | NM_001130173 | -3,80 | 4,55E-08 |
| FAM20B | NM_014864 | -0,65 | 4,57E-08 |
| RALGPS2 | NM_152663 | -1,03 | 4,57E-08 |
| EPB41 | NM_004437 | 1,15 | 4,57E-08 |
| NCOA5 | NM_020967 | -0,75 | 4,60E-08 |
| DNA2 | NM_001080449 | -1,37 | 4,60E-08 |
| ARF4 | NM_001660 | -0,68 | 4,61E-08 |
| WISP1 | NM_003882 | 2,02 | 4,71E-08 |
| USP7 | NM_003470 | -0,62 | 4,76E-08 |
| DUSP14 | NM_007026 | -0,84 | 4,83E-08 |
| ZC3H18 | NM_144604 | -0,70 | 4,84E-08 |
| TLCD2 | NM_001164407 | 1,68 | 4,92E-08 |
| PTP4A1 | NM_003463 | -0,73 | 4,92E-08 |
| UBAP2 | NM_018449 | -1,16 | 4,94E-08 |
| CRY2 | NM_021117 | 1,10 | 4,95E-08 |
| LGMN | NM_001008530 | -0,67 | 4,99E-08 |
| SLC25A29 | NM_001039355 | 0,84 | 5,00E-08 |
| SLC16A12 | NM_213606 | -2,51 | 5,02E-08 |
| STMN3 | NM_015894 | 1,06 | 5,02E-08 |
| COX4NB | NM_001142288 | -1,01 | 5,16E-08 |
| CDK5R1 | NM_003885 | -1,63 | 5,24E-08 |
| DOHH | NM_001145165 | -1,32 | 5,26E-08 |
| EMP3 | NM_001425 | -0,77 | 5,28E-08 |
| FAM172A | NM_001163417 | 0,78 | 5,28E-08 |
| RBBP7 | NM_002893 | -0,68 | 5,35E-08 |
| GLI3 | NM_000168 | 1,06 | 5,35E-08 |
| TMEM35 | NM_021637 | 1,78 | 5,44E-08 |
| TMEM100 | NM_018286 | 2,85 | 5,48E-08 |
| FEZ2 | NM_005102 | -0,79 | 5,57E-08 |
| FPGS | NM_001018078 | -0,79 | 5,58E-08 |
| RPE | NM_199229 | -0,79 | 5,59E-08 |
| ESF1 | NM_016649 | -1,20 | 5,63E-08 |
| PTPRE | NM_006504 | -2,62 | 5,73E-08 |
| SGTB | NM_019072 | -0,96 | 5,77E-08 |
| CDK3 | NM_001258 | 1,48 | 5,78E-08 |
| RARRES1 | NM_206963 | 2,78 | 5,82E-08 |
| ARHGAP6 | NM_013423 | 1,50 | 5,84E-08 |
| LITAF | NM_001136472 | 1,29 | 5,94E-08 |
| DYNLL1 | NM_001037495 | -0,77 | 5,97E-08 |
| KBTBD8 | NM_032505 | -2,38 | 5,98E-08 |
| DPM2 | NM_003863 | -0,85 | 5,98E-08 |
| PAK6 | NM_001128629 | -2,30 | 6,11E-08 |
| NBPF1 | NM_017940 | 0,76 | 6,13E-08 |
| TBC1D22A | NM_014346 | 0,76 | 6,18E-08 |
| RAP1B | NM_015646 | -0,64 | 6,21E-08 |
| FLJ45340 | NR_024368 | 1,01 | 6,27E-08 |
| ATP1A2 | NM_000702 | 3,99 | 6,31E-08 |
| YARS | NM_003680 | -0,65 | 6,32E-08 |
| ASXL3 | NM_030632 | 3,26 | 6,35E-08 |
| MRPL42 | NM_172177 | -1,01 | 6,38E-08 |
| CRIPAK | NM_175918 | 1,09 | 6,45E-08 |
| DBN1 | NM_004395 | -0,68 | 6,50E-08 |
| SYT14 | NM_001146264 | 2,35 | 6,61E-08 |
| WWC3 | NM_015691 | -0,67 | 6,70E-08 |
| PER3 | NM_016831 | 1,07 | 6,76E-08 |
| SYTL5 | NM_001163335 | 3,31 | 6,76E-08 |
| CTDSP1 | NM_021198 | 0,73 | 6,84E-08 |
| ATP2B4 | NM_001001396 | -0,76 | 6,86E-08 |
| RG9MTD1 | NM_017819 | -0,96 | 6,92E-08 |
| KIAA0895 | NM_015314 | 2,09 | 6,95E-08 |
| PLEKHA2 | NM_021623 | -0,80 | 7,03E-08 |
| E2F4 | NM_001950 | -0,66 | 7,06E-08 |
| MPHOSPH10 | NM_005791 | -0,77 | 7,06E-08 |
| TP53BP1 | NM_005657 | 0,67 | 7,06E-08 |
| SLC25A5 | NM_001152 | -0,65 | 7,13E-08 |
| QTRTD1 | NM_024638 | -1,12 | 7,50E-08 |
| RHBDL1 | NM_003961 | 2,35 | 7,58E-08 |
| MAGED4B | NM_030801 | 0,71 | 7,60E-08 |
| NDUFA4L2 | NM_020142 | 0,68 | 7,75E-08 |
| CCL2 | NM_002982 | -1,83 | 7,83E-08 |
| SLC25A10 | NM_012140 | -1,27 | 7,92E-08 |
| TSNAXIP1 | NM_018430 | 2,88 | 7,92E-08 |
| CEP72 | NM_018140 | -1,77 | 7,95E-08 |
| PDAP1 | NM_014891 | -0,88 | 8,04E-08 |
| C14orf79 | NM_174891 | 1,27 | 8,11E-08 |
| GOLT1B | NM_016072 | -0,70 | 8,15E-08 |
| RNF216L | NR_015449 | -0,89 | 8,16E-08 |
| TRIM5 | NM_033034 | -0,75 | 8,16E-08 |
| CAPZA1 | NM_006135 | -0,75 | 8,30E-08 |
| UBE2J1 | NM_016021 | -0,74 | 8,30E-08 |
| C20orf108 | NM_080821 | 1,00 | 8,30E-08 |
| MAGED4 | NM_001098800 | 0,71 | 8,31E-08 |
| MPDZ | NM_003829 | 0,69 | 8,46E-08 |
| ABCA10 | NM_080282 | 3,49 | 8,53E-08 |
| RAB7L1 | NM_001135663 | 0,94 | 8,54E-08 |
| ZDHHC16 | NM_198044 | -0,84 | 8,56E-08 |
| PPT1 | NM_000310 | -0,73 | 8,69E-08 |
| MGAT1 | NM_002406 | -0,60 | 8,89E-08 |
| DOPEY1 | NM_015018 | 0,94 | 8,94E-08 |
| EGFL7 | NM_201446 | -1,05 | 9,06E-08 |
| URB1 | NM_014825 | -0,74 | 9,08E-08 |
| JPH2 | NM_020433 | -1,98 | 9,14E-08 |
| CERKL | NM_001160277 | -1,58 | 9,15E-08 |
| CPSF2 | NM_017437 | -0,63 | 9,16E-08 |
| ELOVL5 | NM_021814 | -0,66 | 9,55E-08 |
| RAC2 | NM_002872 | 0,91 | 9,61E-08 |
| PSMC6 | NM_002806 | -0,85 | 9,74E-08 |
| GTF2A2 | NM_004492 | -0,96 | 9,80E-08 |
| THY1 | NM_006288 | -1,30 | 9,81E-08 |
| CLDN5 | NM_001130861 | -2,10 | 9,85E-08 |
| SH3D19 | NM_001128924 | 0,85 | 9,89E-08 |
| NEK11 | NM_001146003 | 1,86 | 9,94E-08 |
| FAM91A1 | NM_144963 | 0,88 | 1,00E-07 |
| DNAJC5 | NM_025219 | -0,61 | 1,00E-07 |
| UBXN6 | NM_025241 | 0,78 | 1,01E-07 |
| TNPO1 | NM_153188 | -0,65 | 1,01E-07 |
| PAQR6 | NM_024897 | 2,43 | 1,01E-07 |
| PCMT1 | NM_005389 | -0,75 | 1,02E-07 |
| RPF2 | NM_032194 | -1,24 | 1,02E-07 |
| MIIP | NM_021933 | -1,08 | 1,05E-07 |
| C14orf133 | NM_022067 | 0,87 | 1,06E-07 |
| HIATL1 | NM_032558 | -0,74 | 1,06E-07 |
| CYB561D1 | NM_001134404 | 0,91 | 1,06E-07 |
| AKAP8L | NM_014371 | 0,74 | 1,07E-07 |
| ADAM19 | NM_033274 | -0,64 | 1,07E-07 |
| APOL2 | NM_030882 | 0,91 | 1,07E-07 |
| KIRREL3 | NM_032531 | -2,13 | 1,09E-07 |
| NME3 | NM_002513 | 1,26 | 1,11E-07 |
| NAALADL2 | NM_207015 | 1,57 | 1,12E-07 |
| CLIP4 | NM_024692 | 1,00 | 1,12E-07 |
| TUBGCP3 | NM_006322 | -1,04 | 1,12E-07 |
| CCNI | NM_006835 | 0,72 | 1,12E-07 |
| HYLS1 | NM_001134793 | -1,29 | 1,13E-07 |
| DENND1A | NM_020946 | 0,82 | 1,13E-07 |
| DTNB | NM_021907 | 2,05 | 1,14E-07 |
| OTUD6B | NM_016023 | -1,10 | 1,15E-07 |
| ATXN7L1 | NM_138495 | 1,47 | 1,16E-07 |
| SLC41A3 | NM_017836 | 0,76 | 1,20E-07 |
| CALCRL | NM_005795 | 1,27 | 1,21E-07 |
| C19orf10 | NM_019107 | -1,24 | 1,21E-07 |
| POGK | NM_017542 | -0,73 | 1,21E-07 |
| LOC100133331 | NR_028327 | 1,30 | 1,21E-07 |
| SUZ12 | NM_015355 | -0,71 | 1,22E-07 |
| CREM | NM_182717 | 1,48 | 1,22E-07 |
| TBC1D4 | NM_014832 | -0,90 | 1,25E-07 |
| POLR3D | NM_001722 | -0,89 | 1,25E-07 |
| RCAN2 | NM_005822 | 1,85 | 1,25E-07 |
| RGL4 | NM_153615 | 1,70 | 1,26E-07 |
| PNMA2 | NM_007257 | -0,95 | 1,26E-07 |
| MYST4 | NM_012330 | 0,94 | 1,28E-07 |
| GPR81 | NM_032554 | 2,18 | 1,28E-07 |
| SPG11 | NM_025137 | 0,77 | 1,29E-07 |
| NCAPH2 | NM_152299 | -1,20 | 1,30E-07 |
| NRXN2 | NM_138734 | 2,46 | 1,31E-07 |
| GAS2L3 | NM_174942 | -1,35 | 1,32E-07 |
| LRRC2 | NM_024512 | -1,65 | 1,32E-07 |
| RIN1 | NM_004292 | 0,67 | 1,32E-07 |
| ENO3 | NM_053013 | 2,34 | 1,32E-07 |
| TLR3 | NM_003265 | 2,70 | 1,33E-07 |
| TCHP | NM_001143852 | -1,06 | 1,33E-07 |
| EXT1 | NM_000127 | -0,62 | 1,36E-07 |
| ANTXR2 | NM_058172 | 0,66 | 1,41E-07 |
| AFF2 | NM_002025 | 2,40 | 1,44E-07 |
| KIAA1026 | NM_001017999 | -0,76 | 1,44E-07 |
| VPS13C | NM_020821 | 0,95 | 1,44E-07 |
| KLF7 | NM_003709 | -1,35 | 1,45E-07 |
| SH3TC1 | NM_018986 | -1,74 | 1,46E-07 |
| VKORC1L1 | NM_173517 | -1,26 | 1,48E-07 |
| PCDH12 | NM_016580 | -2,60 | 1,53E-07 |
| C17orf68 | NM_025099 | 1,08 | 1,54E-07 |
| LOC284900 | NR_026963 | 1,58 | 1,55E-07 |
| C11orf66 | NM_145017 | 2,87 | 1,55E-07 |
| LONP1 | NM_004793 | -0,88 | 1,56E-07 |
| TNFSF11 | NM_003701 | 4,13 | 1,57E-07 |
| HERC3 | NM_014606 | -0,96 | 1,57E-07 |
| TLK1 | NM_001136554 | 0,73 | 1,59E-07 |
| DDIT3 | NM_004083 | 1,13 | 1,59E-07 |
| MTMR3 | NM_021090 | 0,70 | 1,59E-07 |
| PTDSS1 | NM_014754 | -0,62 | 1,60E-07 |
| ADAP1 | NM_006869 | -1,87 | 1,60E-07 |
| GPR183 | NM_004951 | -3,43 | 1,64E-07 |
| RHBDD2 | NM_001040457 | 0,81 | 1,65E-07 |
| SORD | NM_003104 | -1,10 | 1,66E-07 |
| NPHP1 | NM_001128179 | 2,46 | 1,66E-07 |
| MARCKS | NM_002356 | -0,65 | 1,68E-07 |
| CHURC1 | NM_145165 | 0,81 | 1,70E-07 |
| RPL35 | NM_007209 | -0,66 | 1,72E-07 |
| ABCD4 | NR_003256 | 1,20 | 1,72E-07 |
| PRDM8 | NM_020226 | 1,24 | 1,72E-07 |
| MLKL | NM_152649 | -1,09 | 1,73E-07 |
| CEP152 | NM_014985 | -1,13 | 1,76E-07 |
| ARHGAP18 | NM_033515 | -0,83 | 1,76E-07 |
| ALDH6A1 | NM_005589 | 1,58 | 1,78E-07 |
| SETD3 | NM_032233 | -0,68 | 1,80E-07 |
| HLA-C | NM_002117 | 0,98 | 1,81E-07 |
| CGNL1 | NM_032866 | 1,82 | 1,82E-07 |
| RNF168 | NM_152617 | -0,87 | 1,84E-07 |
| GHDC | NR_024573 | 1,36 | 1,85E-07 |
| GCNT4 | NM_016591 | -2,07 | 1,87E-07 |
| CNNM3 | NM_017623 | 0,95 | 1,87E-07 |
| IPO9 | NM_018085 | -0,73 | 1,89E-07 |
| CCDC92 | NM_025140 | 0,68 | 1,90E-07 |
| SHISA4 | NM_198149 | 1,25 | 1,91E-07 |
| ADORA2B | NM_000676 | -0,89 | 1,97E-07 |
| SEMA7A | NM_001146030 | -0,90 | 1,99E-07 |
| C20orf96 | NM_153269 | 1,25 | 2,00E-07 |
| IMPA1 | NM_001144878 | -0,98 | 2,00E-07 |
| MARS2 | NM_138395 | -1,32 | 2,01E-07 |
| ANKRA2 | NM_023039 | 1,21 | 2,01E-07 |
| VAT1 | NM_006373 | 0,62 | 2,01E-07 |
| C16orf57 | NM_024598 | -0,75 | 2,03E-07 |
| FLJ31306 | NR_029435 | 1,08 | 2,03E-07 |
| RABL2A | NM_013412 | 1,11 | 2,04E-07 |
| HCN3 | NM_020897 | 1,37 | 2,05E-07 |
| SIRT3 | NM_012239 | 0,95 | 2,06E-07 |
| ARID4B | NM_031371 | 0,74 | 2,07E-07 |
| SNHG12 | NR_024127 | -1,42 | 2,09E-07 |
| DDX20 | NM_007204 | -0,87 | 2,12E-07 |
| PTPN1 | NM_002827 | -0,64 | 2,16E-07 |
| ADAMTS8 | NM_007037 | 2,50 | 2,19E-07 |
| ZCWPW1 | NM_017984 | 2,46 | 2,21E-07 |
| KIAA0319L | NM_024874 | 0,69 | 2,24E-07 |
| ZNF32 | NM_006973 | 1,12 | 2,24E-07 |
| UTP18 | NM_016001 | -0,92 | 2,25E-07 |
| SLC26A10 | NM_133489 | 1,97 | 2,25E-07 |
| KRAS | NM_033360 | -0,83 | 2,26E-07 |
| LOC388955 | NR_003131 | -1,32 | 2,29E-07 |
| HHAT | NM_018194 | 1,62 | 2,30E-07 |
| SERPINB9 | NM_004155 | -2,58 | 2,33E-07 |
| KIAA0652 | NR_024589 | 0,63 | 2,34E-07 |
| CASP2 | NM_032982 | -0,83 | 2,34E-07 |
| TMEM143 | NM_018273 | 1,14 | 2,35E-07 |
| CAMK1 | NM_003656 | -1,16 | 2,35E-07 |
| AIG1 | NM_016108 | 1,01 | 2,35E-07 |
| KIF26B | NM_018012 | 2,64 | 2,35E-07 |
| TLE1 | NM_005077 | 1,04 | 2,35E-07 |
| CD9 | NM_001769 | 0,72 | 2,35E-07 |
| LCORL | NM_153686 | -1,19 | 2,37E-07 |
| ZNF192 | NM_006298 | 1,10 | 2,37E-07 |
| TBC1D3C | NM_001001418 | 1,83 | 2,38E-07 |
| DCAF6 | NM_018442 | 0,73 | 2,39E-07 |
| LRRC37A3 | NM_199340 | 1,66 | 2,40E-07 |
| HTR1B | NM_000863 | -3,13 | 2,41E-07 |
| FBXO38 | NM_030793 | 0,73 | 2,43E-07 |
| SEL1L3 | NM_015187 | -1,06 | 2,46E-07 |
| EAF1 | NM_033083 | -0,76 | 2,46E-07 |
| STIM1 | NM_003156 | 0,66 | 2,46E-07 |
| OTUD1 | NM_001145373 | 1,45 | 2,47E-07 |
| NUP160 | NM_015231 | -0,72 | 2,49E-07 |
| DNAL4 | NM_005740 | 1,29 | 2,50E-07 |
| BEND6 | NM_152731 | 1,48 | 2,51E-07 |
| U2AF1 | NM_001025204 | -1,12 | 2,52E-07 |
| LRWD1 | NM_152892 | -0,91 | 2,52E-07 |
| FGD6 | NM_018351 | 1,33 | 2,53E-07 |
| C8orf40 | NM_001135674 | 1,27 | 2,54E-07 |
| NR2C1 | NM_003297 | 0,95 | 2,57E-07 |
| PLCD1 | NM_001130964 | 1,21 | 2,60E-07 |
| NDRG3 | NM_022477 | 1,01 | 2,64E-07 |
| ITGBL1 | NM_004791 | -0,91 | 2,66E-07 |
| CCDC47 | NM_020198 | -0,64 | 2,66E-07 |
| SPNS1 | NM_032038 | 0,64 | 2,68E-07 |
| ZNF514 | NM_032788 | 1,15 | 2,70E-07 |
| TRIOBP | NM_007032 | 0,61 | 2,71E-07 |
| TMEM66 | NM_016127 | 0,71 | 2,73E-07 |
| ANAPC1 | NM_022662 | -0,71 | 2,73E-07 |
| SEMA3F | NM_004186 | -0,91 | 2,77E-07 |
| ACADVL | NM_001033859 | 0,71 | 2,77E-07 |
| MLH1 | NM_000249 | -0,79 | 2,80E-07 |
| HCCS | NM_005333 | -0,95 | 2,82E-07 |
| SLC25A30 | NM_001010875 | -1,14 | 2,82E-07 |
| NLE1 | NM_018096 | -1,03 | 2,82E-07 |
| MAP6 | NM_033063 | 2,04 | 2,84E-07 |
| SSPO | NM_198455 | 4,07 | 2,87E-07 |
| FASTKD2 | NM_001136193 | -0,85 | 2,92E-07 |
| GTF3A | NM_002097 | -0,84 | 2,92E-07 |
| HLA-E | NM_005516 | 0,72 | 2,93E-07 |
| AKT3 | NM_005465 | -0,77 | 2,95E-07 |
| EIF1AD | NM_032325 | -1,00 | 2,95E-07 |
| RGS17 | NM_012419 | 2,41 | 2,97E-07 |
| TAL1 | NM_003189 | -3,13 | 2,97E-07 |
| FAIM2 | NM_012306 | 3,04 | 3,01E-07 |
| DOCK11 | NM_144658 | 0,96 | 3,06E-07 |
| CRY1 | NM_004075 | -0,89 | 3,07E-07 |
| ROM1 | NM_000327 | 2,36 | 3,10E-07 |
| RAB39B | NM_171998 | 3,16 | 3,11E-07 |
| XAF1 | NM_017523 | 1,89 | 3,12E-07 |
| ARPC5L | NM_030978 | -1,03 | 3,12E-07 |
| IFI44 | NM_006417 | 1,79 | 3,12E-07 |
| OBSCN | NM_001098623 | 1,28 | 3,14E-07 |
| SNRPG | NM_003096 | -1,44 | 3,15E-07 |
| CASP8AP2 | NM_001137668 | -0,87 | 3,17E-07 |
| ZFP90 | NM_133458 | 1,01 | 3,18E-07 |
| SCRN2 | NM_001145023 | 1,18 | 3,21E-07 |
| PDGFC | NM_016205 | -0,89 | 3,22E-07 |
| LOC399744 | NR_024497 | 1,18 | 3,28E-07 |
| TBC1D8B | NM_017752 | 1,18 | 3,30E-07 |
| TTC12 | NM_017868 | 1,60 | 3,34E-07 |
| C3orf26 | NM_032359 | -1,17 | 3,37E-07 |
| CXCL16 | NM_022059 | 1,85 | 3,40E-07 |
| GRB2 | NM_002086 | -0,72 | 3,43E-07 |
| PSMA1 | NM_002786 | -0,69 | 3,45E-07 |
| DOCK7 | NM_033407 | -0,74 | 3,47E-07 |
| NRIP3 | NM_020645 | -0,93 | 3,49E-07 |
| FBXO6 | NM_018438 | 2,21 | 3,59E-07 |
| ELF2 | NM_201999 | 0,89 | 3,60E-07 |
| PPM1L | NM_139245 | 1,71 | 3,64E-07 |
| OTUD3 | NM_015207 | -0,94 | 3,65E-07 |
| PRICKLE1 | NM_153026 | 1,22 | 3,68E-07 |
| SLC45A1 | NM_001080397 | 1,82 | 3,70E-07 |
| C13orf31 | NM_001128303 | 1,33 | 3,74E-07 |
| DCP1B | NM_152640 | 1,07 | 3,74E-07 |
| BCAS2 | NM_005872 | -0,97 | 3,81E-07 |
| TGFBI | NM_000358 | 0,72 | 3,83E-07 |
| CFHR1 | NM_002113 | 1,79 | 3,84E-07 |
| IL7R | NM_002185 | -3,80 | 3,84E-07 |
| SNAPC1 | NM_003082 | -1,29 | 3,92E-07 |
| ZNF561 | NM_152289 | 0,82 | 3,98E-07 |
| DUSP8 | NM_004420 | -1,27 | 3,98E-07 |
| GMPS | NM_003875 | -0,63 | 4,03E-07 |
| THTPA | NM_001126339 | 1,24 | 4,06E-07 |
| B4GALT4 | NM_212543 | -0,87 | 4,10E-07 |
| PRDM16 | NM_199454 | 1,06 | 4,12E-07 |
| SRR | NM_021947 | -0,77 | 4,13E-07 |
| SDR39U1 | NM_020195 | 0,82 | 4,16E-07 |
| PRKCH | NM_006255 | -1,28 | 4,17E-07 |
| C20orf3 | NM_020531 | -0,65 | 4,18E-07 |
| LPAR1 | NM_001401 | 0,94 | 4,20E-07 |
| C1orf96 | NM_145257 | -0,92 | 4,22E-07 |
| PARVA | NM_018222 | -0,72 | 4,24E-07 |
| RTCD1 | NM_003729 | -0,76 | 4,24E-07 |
| PHLDB3 | NM_198850 | 1,79 | 4,29E-07 |
| RORA | NM_134261 | 1,57 | 4,31E-07 |
| NOL10 | NM_024894 | -0,87 | 4,32E-07 |
| ZNF362 | NM_152493 | 1,13 | 4,32E-07 |
| AKAP9 | NM_005751 | 0,88 | 4,33E-07 |
| DTX2 | NM_020892 | 0,84 | 4,34E-07 |
| DDX46 | NM_014829 | -0,69 | 4,36E-07 |
| HIBADH | NM_152740 | 0,78 | 4,37E-07 |
| BCL7B | NM_001707 | -0,70 | 4,37E-07 |
| CC2D2A | NM_001080522 | 0,85 | 4,48E-07 |
| RAVER1 | NM_133452 | -0,65 | 4,50E-07 |
| PDCD10 | NM_145859 | -1,00 | 4,51E-07 |
| STK17B | NM_004226 | -0,79 | 4,53E-07 |
| RPUSD1 | NM_058192 | -0,88 | 4,55E-07 |
| RFX5 | NM_000449 | 0,77 | 4,60E-07 |
| SLC1A2 | NM_004171 | 3,63 | 4,61E-07 |
| C5orf26 | NR_015370 | 1,68 | 4,61E-07 |
| UBE2M | NM_003969 | -0,83 | 4,63E-07 |
| DHX40 | NM_001166301 | 0,79 | 4,66E-07 |
| HTR2A | NM_000621 | 4,02 | 4,67E-07 |
| BCL3 | NM_005178 | 1,05 | 4,71E-07 |
| ARL4A | NM_005738 | 1,04 | 4,74E-07 |
| GPR172A | NM_024531 | -0,75 | 4,80E-07 |
| CBLB | NM_170662 | 0,87 | 4,81E-07 |
| MAN2B2 | NM_015274 | 0,72 | 4,81E-07 |
| TTC27 | NM_017735 | -0,97 | 4,85E-07 |
| PIF1 | NM_025049 | -1,28 | 4,87E-07 |
| CTBS | NM_004388 | 0,98 | 4,92E-07 |
| NOC4L | NM_024078 | -0,85 | 4,93E-07 |
| FAM3C | NM_001040020 | -0,67 | 4,94E-07 |
| C2orf64 | NM_001008215 | 1,37 | 4,94E-07 |
| KIF2A | NM_004520 | -0,72 | 4,94E-07 |
| RIC8A | NM_021932 | -0,65 | 4,99E-07 |
| USP39 | NM_006590 | -0,67 | 4,99E-07 |
| GALT | NM_000155 | 1,13 | 5,05E-07 |
| ENPEP | NM_001977 | -2,57 | 5,05E-07 |
| AHNAK2 | NM_138420 | 0,75 | 5,06E-07 |
| CHST11 | NM_018413 | -1,09 | 5,11E-07 |
| ITGB1BP1 | NM_004763 | -0,77 | 5,14E-07 |
| FBLN5 | NM_006329 | 0,79 | 5,19E-07 |
| HIST3H2A | NM_033445 | 1,95 | 5,27E-07 |
| TTC17 | NM_018259 | 0,81 | 5,28E-07 |
| PIGQ | NM_004204 | 0,66 | 5,30E-07 |
| DDX55 | NM_020936 | -0,95 | 5,36E-07 |
| POLS | NM_006999 | -0,74 | 5,39E-07 |
| NT5E | NM_002526 | -0,66 | 5,40E-07 |
| PLAC9 | NM_001012973 | 1,18 | 5,45E-07 |
| FAM134A | NM_024293 | 0,63 | 5,47E-07 |
| MGAT2 | NM_002408 | -0,80 | 5,56E-07 |
| PSMG3 | NM_032302 | -1,14 | 5,58E-07 |
| FGGY | NM_018291 | 1,60 | 5,59E-07 |
| AMZ2 | NM_016627 | 0,64 | 5,61E-07 |
| TCEB1 | NM_005648 | -0,93 | 5,62E-07 |
| TEF | NM_003216 | 1,05 | 5,73E-07 |
| LRCH4 | NM_002319 | 0,69 | 5,73E-07 |
| ARMC1 | NM_018120 | -0,80 | 5,77E-07 |
| RLTPR | NM_001013838 | -1,83 | 5,79E-07 |
| CCT4 | NM_006430 | -0,80 | 5,80E-07 |
| HSD17B11 | NM_016245 | 1,06 | 5,85E-07 |
| SLC7A6OS | NM_032178 | -0,87 | 5,85E-07 |
| MAST3 | NM_015016 | 1,09 | 5,88E-07 |
| LLPH | NM_032338 | -0,87 | 5,89E-07 |
| LIN54 | NM_194282 | -1,03 | 5,92E-07 |
| BRMS1 | NM_015399 | -0,96 | 5,97E-07 |
| CD4 | NM_000616 | 1,23 | 6,07E-07 |
| SH2D3C | NM_001142534 | -1,12 | 6,11E-07 |
| C6orf211 | NM_024573 | -0,87 | 6,13E-07 |
| GNL3L | NM_019067 | -0,72 | 6,16E-07 |
| C17orf39 | NM_024052 | 0,95 | 6,20E-07 |
| SLC9A9 | NM_173653 | 3,70 | 6,21E-07 |
| LARS2 | NM_015340 | -0,86 | 6,23E-07 |
| CRK | NM_016823 | -0,62 | 6,23E-07 |
| USP40 | NM_018218 | 0,70 | 6,24E-07 |
| VILL | NM_015873 | 1,27 | 6,33E-07 |
| ZNF93 | NM_031218 | -2,42 | 6,44E-07 |
| ERAL1 | NM_005702 | -0,73 | 6,45E-07 |
| C8orf30A | NM_016458 | -0,78 | 6,46E-07 |
| SIX4 | NM_017420 | 0,97 | 6,55E-07 |
| FAT3 | NM_001008781 | 1,45 | 6,58E-07 |
| ESM1 | NM_001135604 | -2,49 | 6,63E-07 |
| LPAR2 | NM_004720 | 2,15 | 6,64E-07 |
| EIF4A3 | NM_014740 | -0,77 | 6,65E-07 |
| ELMO1 | NM_014800 | -3,41 | 6,68E-07 |
| MBD3 | NM_003926 | -0,83 | 6,68E-07 |
| CLEC3B | NM_003278 | 1,18 | 6,74E-07 |
| SFRS13A | NM_006625 | -0,76 | 6,76E-07 |
| PI4K2B | NM_018323 | -0,84 | 6,81E-07 |
| FGFRL1 | NM_001004356 | -0,68 | 6,84E-07 |
| CHCHD2 | NM_016139 | -0,81 | 6,89E-07 |
| HSD17B12 | NM_016142 | -0,69 | 6,94E-07 |
| PRDM2 | NM_001135610 | 1,81 | 7,04E-07 |
| ZNF580 | NM_016202 | 0,98 | 7,04E-07 |
| ZBTB41 | NM_194314 | 0,93 | 7,08E-07 |
| ERCC5 | NM_000123 | 0,75 | 7,10E-07 |
| ASL | NM_001024944 | -1,11 | 7,11E-07 |
| LPIN2 | NM_014646 | 0,69 | 7,11E-07 |
| GGN | NM_152657 | 1,81 | 7,24E-07 |
| CYTL1 | NM_018659 | 0,81 | 7,26E-07 |
| ATP7A | NM_000052 | 0,96 | 7,27E-07 |
| HMOX2 | NM_001127205 | -0,95 | 7,28E-07 |
| TACC1 | NM_006283 | -0,60 | 7,29E-07 |
| CDKN2C | NM_001262 | -0,98 | 7,34E-07 |
| C11orf63 | NM_024806 | 1,71 | 7,39E-07 |
| RAB40B | NM_006822 | 1,10 | 7,40E-07 |
| ANKRD27 | NM_032139 | -0,78 | 7,49E-07 |
| SDAD1 | NM_018115 | -0,75 | 7,54E-07 |
| EMP2 | NM_001424 | -0,71 | 7,55E-07 |
| CCM2 | NM_001029835 | -0,95 | 7,61E-07 |
| SPPL3 | NM_139015 | 0,76 | 7,64E-07 |
| MGMT | NM_002412 | 1,19 | 7,68E-07 |
| TRIM36 | NM_018700 | -1,93 | 7,79E-07 |
| CLCN4 | NM_001830 | -1,33 | 7,85E-07 |
| ZNF606 | NM_025027 | 1,19 | 7,85E-07 |
| TMEM205 | NM_198536 | 0,90 | 7,90E-07 |
| ULBP2 | NM_025217 | -1,86 | 7,92E-07 |
| FAM19A5 | NM_001082967 | -1,13 | 7,96E-07 |
| C17orf44 | NR_026951 | 3,41 | 8,03E-07 |
| ANK1 | NM_020480 | 0,86 | 8,07E-07 |
| CROCC | NM_014675 | 1,16 | 8,20E-07 |
| AGTRAP | NM_001040197 | 0,86 | 8,21E-07 |
| UBAC2 | NR_026644 | 0,68 | 8,25E-07 |
| CCDC136 | NM_022742 | 1,86 | 8,27E-07 |
| MAPKAP1 | NM_001006618 | -0,72 | 8,34E-07 |
| UBE2L3 | NM_003347 | -0,77 | 8,48E-07 |
| SYNJ1 | NM_001160306 | 0,91 | 8,51E-07 |
| IQGAP1 | NM_003870 | -0,61 | 8,59E-07 |
| ZNF471 | NM_020813 | 1,89 | 8,61E-07 |
| SEC16B | NM_033127 | 1,74 | 8,66E-07 |
| RALB | NM_002881 | -0,61 | 8,75E-07 |
| CORO1A | NM_007074 | -2,31 | 8,82E-07 |
| CCNJ | NM_001134375 | -1,08 | 8,90E-07 |
| C13orf15 | NM_014059 | -3,54 | 8,90E-07 |
| SIK1 | NM_173354 | -1,29 | 8,98E-07 |
| MPP7 | NM_173496 | -1,58 | 9,13E-07 |
| BRPF1 | NM_001003694 | -0,72 | 9,17E-07 |
| NOL9 | NM_024654 | -0,80 | 9,21E-07 |
| SPATA7 | NM_018418 | 1,72 | 9,27E-07 |
| NALCN | NM_052867 | 1,51 | 9,29E-07 |
| PTK2B | NM_173174 | 2,24 | 9,43E-07 |
| TOE1 | NM_025077 | -1,09 | 9,51E-07 |
| SDC1 | NM_002997 | -0,72 | 9,58E-07 |
| ASCC3 | NM_006828 | -0,64 | 9,63E-07 |
| FAM125A | NM_138401 | 0,86 | 9,64E-07 |
| FAM107A | NM_007177 | -2,62 | 9,78E-07 |
| TSSC1 | NM_003310 | -1,07 | 9,78E-07 |
| FAM177A1 | NM_001079519 | -0,76 | 9,79E-07 |
| DPH3 | NM_001047434 | -0,82 | 9,85E-07 |
| DDX23 | NM_004818 | -0,72 | 9,89E-07 |
| TPBG | NM_006670 | 0,88 | 9,90E-07 |
| HIRIP3 | NM_003609 | -1,01 | 9,93E-07 |
| APOBEC3D | NM_152426 | 2,40 | 9,94E-07 |
| MRPS34 | NM_023936 | -0,63 | 1,00E-06 |
| ATG2B | NM_018036 | 0,77 | 1,00E-06 |
| APBB2 | NM_004307 | -0,76 | 1,01E-06 |
| SLC9A1 | NM_003047 | 0,77 | 1,01E-06 |
| INADL | NM_176877 | 1,62 | 1,02E-06 |
| ATP6V1D | NM_015994 | -0,71 | 1,03E-06 |
| DNAJB6 | NM_005494 | -0,72 | 1,04E-06 |
| SLC35A4 | NM_080670 | -0,61 | 1,05E-06 |
| SOBP | NM_018013 | 0,84 | 1,06E-06 |
| MIR636 | NR_030366 | -1,70 | 1,06E-06 |
| SRP9 | NM_003133 | -0,67 | 1,06E-06 |
| CEBPD | NM_005195 | 1,53 | 1,06E-06 |
| EFNA5 | NM_001962 | 1,09 | 1,08E-06 |
| LEPR | NM_001003679 | 1,14 | 1,08E-06 |
| TMEM25 | NM_032780 | 0,85 | 1,09E-06 |
| RIF1 | NM_018151 | -0,67 | 1,09E-06 |
| BIVM | NM_001159596 | 0,83 | 1,09E-06 |
| GSDMD | NM_024736 | 1,16 | 1,09E-06 |
| HIST2H2AA3 | NM_003516 | 1,30 | 1,10E-06 |
| HIST2H2AA4 | NM_001040874 | 1,30 | 1,10E-06 |
| ACER3 | NM_018367 | -0,88 | 1,10E-06 |
| MLX | NM_170607 | -0,84 | 1,11E-06 |
| HMGA2 | NM_003484 | -0,64 | 1,11E-06 |
| SSSCA1 | NM_006396 | -0,98 | 1,11E-06 |
| SYAP1 | NM_032796 | -0,69 | 1,12E-06 |
| SNRPE | NM_003094 | -1,18 | 1,12E-06 |
| EFNB2 | NM_004093 | -0,60 | 1,14E-06 |
| MPPE1 | NM_023075 | 1,17 | 1,14E-06 |
| FBXO36 | NM_174899 | 1,96 | 1,14E-06 |
| TBC1D9 | NM_015130 | -0,69 | 1,15E-06 |
| TBCB | NM_001281 | -0,64 | 1,15E-06 |
| FAM21A | NM_001005751 | 0,70 | 1,16E-06 |
| KATNB1 | NM_005886 | -0,77 | 1,17E-06 |
| DUSP3 | NM_004090 | -0,67 | 1,18E-06 |
| NAT5 | NM_181527 | -0,75 | 1,18E-06 |
| ZNF295 | NM_001098403 | -1,00 | 1,18E-06 |
| NAGLU | NM_000263 | 0,68 | 1,19E-06 |
| FZD2 | NM_001466 | 0,92 | 1,19E-06 |
| IFT172 | NM_015662 | 1,05 | 1,20E-06 |
| LOC388796 | NR_015366 | -1,39 | 1,21E-06 |
| ZNF546 | NM_178544 | 1,82 | 1,21E-06 |
| DAPK2 | NM_014326 | 2,54 | 1,21E-06 |
| FLJ10038 | NR_026891 | 1,35 | 1,22E-06 |
| ACOT9 | NM_001033583 | -0,73 | 1,22E-06 |
| C11orf35 | NM_173573 | 2,80 | 1,23E-06 |
| CARD6 | NM_032587 | 1,27 | 1,24E-06 |
| PPIH | NM_006347 | -1,27 | 1,24E-06 |
| PGAM2 | NM_000290 | 2,05 | 1,25E-06 |
| MOBKL3 | NM_015387 | -0,79 | 1,25E-06 |
| BAG3 | NM_004281 | -0,82 | 1,26E-06 |
| C18orf8 | NM_013326 | -1,01 | 1,27E-06 |
| IMPAD1 | NM_017813 | -0,70 | 1,27E-06 |
| SLC19A2 | NM_006996 | -1,41 | 1,28E-06 |
| NUDT1 | NM_198953 | -1,40 | 1,30E-06 |
| LOC283050 | NR_024431 | 3,50 | 1,31E-06 |
| GDPD3 | NM_024307 | 1,87 | 1,32E-06 |
| MRPL39 | NM_017446 | -1,17 | 1,33E-06 |
| FAM160A2 | NM_032127 | 0,74 | 1,33E-06 |
| HDAC5 | NM_001015053 | 0,67 | 1,34E-06 |
| CUL4A | NM_003589 | -0,62 | 1,34E-06 |
| RASIP1 | NM_017805 | -2,11 | 1,35E-06 |
| RBM19 | NM_016196 | -0,77 | 1,35E-06 |
| ACSL3 | NM_004457 | -0,71 | 1,36E-06 |
| CDC25B | NM_021873 | 0,60 | 1,38E-06 |
| PLEKHF1 | NM_024310 | 1,16 | 1,38E-06 |
| PBX2 | NM_002586 | 0,78 | 1,38E-06 |
| HADHB | NM_000183 | 0,66 | 1,38E-06 |
| LIF | NM_002309 | 0,77 | 1,39E-06 |
| KCNG1 | NM_002237 | -0,97 | 1,39E-06 |
| FAM49B | NM_016623 | -1,05 | 1,40E-06 |
| HES4 | NM_021170 | -1,31 | 1,42E-06 |
| INHA | NM_002191 | 3,35 | 1,42E-06 |
| TMEM85 | NM_016454 | -0,65 | 1,42E-06 |
| CRIP2 | NM_001312 | -0,83 | 1,43E-06 |
| ZNF397OS | NM_001166012 | 0,98 | 1,44E-06 |
| PVRL2 | NM_001042724 | -0,64 | 1,44E-06 |
| SIGIRR | NM_021805 | 1,22 | 1,45E-06 |
| SLC25A17 | NM_006358 | -0,94 | 1,46E-06 |
| RAMP1 | NM_005855 | 1,77 | 1,46E-06 |
| SYP | NM_003179 | 2,34 | 1,47E-06 |
| WDR89 | NM_080666 | -1,11 | 1,47E-06 |
| ZCCHC11 | NM_001009882 | 0,74 | 1,49E-06 |
| LEPROTL1 | NM_015344 | -0,80 | 1,50E-06 |
| GPRC5B | NM_016235 | -1,90 | 1,51E-06 |
| HEG1 | NM_020733 | -0,74 | 1,53E-06 |
| LOC400931 | NR_027033 | 1,76 | 1,54E-06 |
| CRIP1 | NM_001311 | -2,15 | 1,54E-06 |
| SPOCK1 | NM_004598 | 1,11 | 1,56E-06 |
| VPS8 | NM_001009921 | 0,87 | 1,56E-06 |
| GPR1 | NM_001098199 | -1,64 | 1,56E-06 |
| RBP1 | NM_002899 | 2,82 | 1,57E-06 |
| PCCA | NM_000282 | 1,29 | 1,58E-06 |
| AKAP11 | NM_016248 | 0,71 | 1,61E-06 |
| NCOA7 | NM_181782 | 0,80 | 1,61E-06 |
| IDS | NM_000202 | 0,71 | 1,61E-06 |
| SLC29A1 | NM_001078177 | -0,96 | 1,64E-06 |
| GSTO1 | NM_004832 | -0,78 | 1,64E-06 |
| UTY | NM_007125 | 1,13 | 1,65E-06 |
| SLC25A27 | NM_004277 | 2,56 | 1,67E-06 |
| GPRC5C | NM_022036 | 2,08 | 1,70E-06 |
| GPR137B | NM_003272 | 1,34 | 1,70E-06 |
| TTC8 | NM_198310 | 1,07 | 1,73E-06 |
| FAM72D | NM_207418 | -1,72 | 1,74E-06 |
| C6orf129 | NM_138493 | -1,11 | 1,75E-06 |
| ZNF75D | NM_007131 | 1,26 | 1,77E-06 |
| TCTN1 | NM_001082538 | 1,07 | 1,80E-06 |
| TMED9 | NM_017510 | -0,85 | 1,81E-06 |
| PTCH1 | NM_001083604 | 1,19 | 1,82E-06 |
| GNPTG | NM_032520 | 1,05 | 1,87E-06 |
| SPC24 | NM_182513 | -1,58 | 1,88E-06 |
| C20orf72 | NM_052865 | -0,85 | 1,89E-06 |
| ZNF688 | NM_145271 | 1,38 | 1,89E-06 |
| OBFC2A | NM_001031716 | -0,92 | 1,89E-06 |
| TRIM45 | NM_025188 | 1,30 | 1,91E-06 |
| PPP1R3E | NR_026862 | 1,27 | 1,91E-06 |
| RFT1 | NM_052859 | -0,87 | 1,92E-06 |
| TLR6 | NM_006068 | 1,90 | 1,95E-06 |
| NUFIP1 | NM_012345 | -1,14 | 1,96E-06 |
| CDKN2A | NM_058197 | 0,93 | 2,02E-06 |
| C1orf54 | NM_024579 | 1,39 | 2,03E-06 |
| CCDC103 | NM_213607 | 2,37 | 2,03E-06 |
| DNASE1 | NM_005223 | 1,30 | 2,03E-06 |
| CSRNP3 | NM_024969 | 3,62 | 2,04E-06 |
| NANP | NM_152667 | -1,13 | 2,04E-06 |
| ZNF16 | NM_006958 | 1,22 | 2,05E-06 |
| HAUS2 | NM_018097 | -0,93 | 2,06E-06 |
| CYGB | NM_134268 | 1,44 | 2,06E-06 |
| STAM | NM_003473 | -0,80 | 2,07E-06 |
| WDR6 | NM_018031 | 0,67 | 2,07E-06 |
| FRMD4B | NM_015123 | -2,18 | 2,07E-06 |
| PTHLH | NM_002820 | -2,58 | 2,08E-06 |
| GTF3C6 | NM_138408 | -0,94 | 2,08E-06 |
| SCPEP1 | NM_021626 | 0,89 | 2,10E-06 |
| MYL9 | NM_006097 | -0,89 | 2,10E-06 |
| TSGA14 | NM_018718 | -0,90 | 2,11E-06 |
| NCRNA00085 | NR_024330 | 1,51 | 2,12E-06 |
| PNMAL2 | NM_020709 | 2,82 | 2,12E-06 |
| NRM | NM_007243 | -0,84 | 2,13E-06 |
| CCDC74A | NM_138770 | 1,30 | 2,14E-06 |
| DUSP4 | NM_001394 | 0,80 | 2,15E-06 |
| SLC35C2 | NM_173179 | 0,70 | 2,19E-06 |
| LOC92973 | NR_024006 | 3,62 | 2,19E-06 |
| HDAC10 | NM_001159286 | 1,07 | 2,20E-06 |
| NDUFAF4 | NM_014165 | -1,41 | 2,20E-06 |
| ZNF558 | NM_144693 | 1,06 | 2,22E-06 |
| GRAMD1A | NM_001136199 | 0,71 | 2,23E-06 |
| ITPR1 | NM_002222 | 1,13 | 2,24E-06 |
| MAGEE1 | NM_020932 | 1,30 | 2,25E-06 |
| LZTFL1 | NM_020347 | 1,20 | 2,25E-06 |
| DHX34 | NM_014681 | -0,68 | 2,28E-06 |
| RRP7B | NR_002184 | -1,12 | 2,29E-06 |
| ZNF687 | NM_020832 | 0,67 | 2,32E-06 |
| C7orf44 | NM_018224 | -0,89 | 2,32E-06 |
| SCARF1 | NR_028076 | -1,88 | 2,33E-06 |
| NR2C2AP | NM_176880 | -1,06 | 2,34E-06 |
| ZFP14 | NM_020917 | 1,67 | 2,35E-06 |
| MYLIP | NM_013262 | 1,57 | 2,36E-06 |
| RNF138 | NM_016271 | -1,03 | 2,40E-06 |
| UNK | NM_001080419 | 0,82 | 2,40E-06 |
| LOC645166 | NR_027355 | -1,59 | 2,42E-06 |
| HBS1L | NM_001145158 | -0,70 | 2,45E-06 |
| TNFSF10 | NM_003810 | -3,81 | 2,47E-06 |
| HCFC1 | NM_005334 | -0,72 | 2,48E-06 |
| C4orf21 | NM_018392 | -1,21 | 2,49E-06 |
| TAPBPL | NM_018009 | 1,37 | 2,49E-06 |
| ZNF767 | NR_027788 | 0,98 | 2,50E-06 |
| BOLA1 | NM_016074 | 1,32 | 2,50E-06 |
| RELL1 | NM_001085400 | -0,79 | 2,50E-06 |
| RABL4 | NM_006860 | 1,25 | 2,51E-06 |
| REXO2 | NM_015523 | -0,77 | 2,52E-06 |
| C8orf41 | NM_025115 | -0,88 | 2,53E-06 |
| MRPS15 | NM_031280 | -0,82 | 2,56E-06 |
| SNORA11D | NR_003711 | 2,72 | 2,58E-06 |
| SNORA11E | NR_003712 | 2,72 | 2,58E-06 |
| DALRD3 | NM_001009996 | 0,71 | 2,60E-06 |
| ERAP2 | NM_022350 | 0,73 | 2,60E-06 |
| SC5DL | NM_006918 | -0,83 | 2,63E-06 |
| UBE2QP1 | NR_003661 | 1,68 | 2,66E-06 |
| CLIP1 | NM_002956 | -0,66 | 2,66E-06 |
| APOL3 | NR_027833 | 1,96 | 2,71E-06 |
| C20orf165 | NM_080608 | 2,73 | 2,72E-06 |
| SFRS9 | NM_003769 | -0,79 | 2,76E-06 |
| WDR13 | NM_001166426 | 0,62 | 2,76E-06 |
| KIAA1522 | NM_020888 | 1,03 | 2,77E-06 |
| MTRF1L | NM_001114184 | -0,95 | 2,81E-06 |
| C1orf101 | NM_001130957 | 3,59 | 2,82E-06 |
| DECR1 | NM_001359 | 0,80 | 2,83E-06 |
| ARHGEF12 | NM_015313 | 0,64 | 2,83E-06 |
| MOSPD3 | NM_001040098 | 0,99 | 2,84E-06 |
| RGS7 | NM_002924 | -2,68 | 2,86E-06 |
| EIF2S2 | NM_003908 | -0,65 | 2,87E-06 |
| NOVA1 | NM_002515 | 2,55 | 2,87E-06 |
| LRRFIP2 | NM_006309 | -0,71 | 2,89E-06 |
| RAB37 | NM_175738 | 3,58 | 2,90E-06 |
| C1orf26 | NM_001105518 | 1,74 | 2,92E-06 |
| SLC25A20 | NM_000387 | 1,04 | 2,92E-06 |
| DES | NM_001927 | 0,89 | 2,92E-06 |
| CCDC59 | NM_014167 | -1,02 | 2,93E-06 |
| STT3A | NM_152713 | -0,69 | 2,94E-06 |
| MINA | NM_001042533 | -0,66 | 2,96E-06 |
| USP21 | NM_001014443 | 0,77 | 2,96E-06 |
| PTPRA | NM_002836 | 0,70 | 2,98E-06 |
| RPAIN | NR_027684 | -0,87 | 2,99E-06 |
| FBXO9 | NM_012347 | 0,71 | 3,02E-06 |
| SHQ1 | NM_018130 | -0,97 | 3,02E-06 |
| NKTR | NM_005385 | 0,76 | 3,03E-06 |
| PCTK3 | NM_002596 | 2,44 | 3,03E-06 |
| MAP3K11 | NM_002419 | -0,69 | 3,05E-06 |
| TBCE | NM_001079515 | -0,80 | 3,05E-06 |
| C3orf63 | NM_001112736 | 0,61 | 3,06E-06 |
| SNX29 | NM_001080530 | 0,81 | 3,09E-06 |
| SCML1 | NM_001037535 | -1,02 | 3,09E-06 |
| PHAX | NM_032177 | -0,71 | 3,09E-06 |
| POLD4 | NM_021173 | 0,80 | 3,09E-06 |
| MPV17L2 | NM_032683 | -1,00 | 3,12E-06 |
| FAM98B | NM_173611 | -1,03 | 3,13E-06 |
| DPP3 | NM_005700 | -0,88 | 3,14E-06 |
| SHF | NM_138356 | 2,58 | 3,15E-06 |
| SDHB | NM_003000 | -0,73 | 3,20E-06 |
| EVI5L | NM_145245 | 0,73 | 3,22E-06 |
| FAM21B | NM_018232 | 0,72 | 3,24E-06 |
| C11orf2 | NM_013265 | 0,83 | 3,25E-06 |
| PSMA4 | NM_001102667 | -0,62 | 3,31E-06 |
| PPTC7 | NM_139283 | -0,85 | 3,32E-06 |
| KIAA0240 | NM_015349 | 1,13 | 3,39E-06 |
| METTL3 | NM_019852 | -0,81 | 3,40E-06 |
| INO80 | NM_017553 | -0,70 | 3,42E-06 |
| TOR1A | NM_000113 | -0,62 | 3,43E-06 |
| CPOX | NM_000097 | -0,81 | 3,44E-06 |
| PPFIA4 | NM_015053 | -1,44 | 3,45E-06 |
| PSMB9 | NM_148954 | 2,11 | 3,46E-06 |
| TEX10 | NM_017746 | -0,77 | 3,47E-06 |
| RPN2 | NM_001135771 | -0,61 | 3,48E-06 |
| KCNK3 | NM_002246 | 2,82 | 3,50E-06 |
| BCL7A | NM_020993 | -0,87 | 3,51E-06 |
| CROCCL2 | NR_023386 | 1,21 | 3,54E-06 |
| BCL2L12 | NM_001040668 | -0,98 | 3,54E-06 |
| RUNDC1 | NM_173079 | -0,78 | 3,54E-06 |
| ZNF334 | NM_018102 | 2,53 | 3,55E-06 |
| VMA21 | NM_001017980 | -0,66 | 3,55E-06 |
| GDF6 | NM_001001557 | -1,02 | 3,57E-06 |
| TMEM123 | NM_052932 | -0,77 | 3,59E-06 |
| COL27A1 | NM_032888 | 0,65 | 3,62E-06 |
| MRE11A | NM_005591 | -0,79 | 3,64E-06 |
| ADPGK | NM_031284 | -0,68 | 3,64E-06 |
| TEX264 | NM_015926 | 0,88 | 3,65E-06 |
| NEK1 | NM_012224 | 0,91 | 3,65E-06 |
| PYCR2 | NM_013328 | -0,70 | 3,65E-06 |
| WDR47 | NM_001142550 | -0,80 | 3,67E-06 |
| HFE | NM_139008 | 1,54 | 3,69E-06 |
| IGF2BP1 | NM_006546 | -0,60 | 3,70E-06 |
| SPIRE2 | NM_032451 | 1,85 | 3,75E-06 |
| C6orf225 | NM_001033564 | 1,43 | 3,79E-06 |
| PBLD | NM_022129 | 1,33 | 3,80E-06 |
| TIMM8A | NM_004085 | -1,53 | 3,80E-06 |
| JHDM1D | NM_030647 | 1,08 | 3,80E-06 |
| LYL1 | NM_005583 | -2,68 | 3,81E-06 |
| MTMR2 | NM_201281 | -0,68 | 3,82E-06 |
| CDK2AP2 | NM_005851 | -0,89 | 3,83E-06 |
| SATB2 | NM_015265 | 1,12 | 3,85E-06 |
| MBD6 | NM_052897 | 0,91 | 3,86E-06 |
| OXA1L | NM_005015 | 0,66 | 3,89E-06 |
| ZNF740 | NM_001004304 | 0,80 | 3,89E-06 |
| C12orf4 | NM_020374 | -0,81 | 3,89E-06 |
| AUH | NM_001698 | 1,44 | 3,89E-06 |
| RAP2A | NM_021033 | -0,74 | 3,92E-06 |
| SLC22A5 | NM_003060 | 1,53 | 3,94E-06 |
| DDA1 | NM_024050 | -0,66 | 4,01E-06 |
| MSI2 | NM_138962 | 1,36 | 4,01E-06 |
| PQLC3 | NM_152391 | 1,05 | 4,02E-06 |
| PSMB2 | NM_002794 | -0,81 | 4,04E-06 |
| ALPK2 | NM_052947 | -0,90 | 4,09E-06 |
| HIP1R | NM_003959 | 0,78 | 4,13E-06 |
| RAE1 | NM_003610 | -0,74 | 4,15E-06 |
| ANKRD33B | NM_001164440 | -0,88 | 4,21E-06 |
| SIK3 | NM_025164 | 0,83 | 4,25E-06 |
| MTMR12 | NM_001040446 | -0,69 | 4,26E-06 |
| GNL1 | NM_005275 | 0,79 | 4,30E-06 |
| CEBPG | NM_001806 | -0,70 | 4,33E-06 |
| HCG18 | NR_024052 | -0,85 | 4,36E-06 |
| HK1 | NM_033496 | -0,60 | 4,37E-06 |
| ZNF524 | NM_153219 | 1,28 | 4,38E-06 |
| REM1 | NM_014012 | -1,89 | 4,39E-06 |
| C1orf163 | NM_023077 | -1,00 | 4,41E-06 |
| C6orf48 | NM_001040438 | 0,83 | 4,45E-06 |
| GTF2E2 | NM_002095 | -0,81 | 4,57E-06 |
| MTR | NM_000254 | -0,61 | 4,60E-06 |
| PPP2R2A | NM_002717 | -0,71 | 4,61E-06 |
| KIAA0528 | NM_014802 | 0,72 | 4,61E-06 |
| CLEC11A | NM_002975 | 1,25 | 4,66E-06 |
| MYH7B | NM_020884 | 2,42 | 4,66E-06 |
| LASS4 | NM_024552 | 2,30 | 4,68E-06 |
| SYN1 | NM_006950 | 2,14 | 4,69E-06 |
| SAP30 | NM_003864 | -1,25 | 4,70E-06 |
| ITFG1 | NM_030790 | 0,74 | 4,72E-06 |
| FUK | NM_145059 | 1,13 | 4,80E-06 |
| PNN | NM_002687 | -0,63 | 4,81E-06 |
| MACF1 | NM_033044 | -0,69 | 4,81E-06 |
| POLA1 | NM_016937 | -0,96 | 4,87E-06 |
| GRAMD1C | NM_017577 | 1,79 | 4,87E-06 |
| TBC1D5 | NM_001134381 | 0,69 | 4,89E-06 |
| USP37 | NM_020935 | -0,89 | 4,90E-06 |
| TMEM91 | NM_001098824 | 1,75 | 4,92E-06 |
| LOC100128076 | NR_015444 | 3,53 | 4,95E-06 |
| DHRS12 | NM_024705 | 2,91 | 4,96E-06 |
| APC2 | NM_005883 | 1,46 | 5,03E-06 |
| F11R | NM_016946 | -1,70 | 5,03E-06 |
| ZNF529 | NM_001145649 | 1,03 | 5,09E-06 |
| PHACTR2 | NM_001100164 | 0,80 | 5,11E-06 |
| ZNF248 | NM_021045 | 0,95 | 5,13E-06 |
| PIP5KL1 | NM_173492 | 1,61 | 5,14E-06 |
| VPRBP | NM_014703 | -0,67 | 5,14E-06 |
| PLEKHJ1 | NM_018049 | -1,06 | 5,15E-06 |
| TCEAL8 | NM_153333 | 0,74 | 5,15E-06 |
| ERO1LB | NM_019891 | 1,34 | 5,15E-06 |
| LAMA1 | NM_005559 | -0,99 | 5,16E-06 |
| LPIN3 | NM_022896 | 1,05 | 5,17E-06 |
| NANS | NM_018946 | -0,77 | 5,30E-06 |
| LOC220930 | NR_024284 | 1,22 | 5,33E-06 |
| ANKZF1 | NM_001042410 | 0,76 | 5,33E-06 |
| FAM65A | NM_024519 | -0,89 | 5,37E-06 |
| SLFN11 | NM_001104587 | -0,68 | 5,39E-06 |
| SSBP1 | NM_003143 | -0,82 | 5,39E-06 |
| FAM57A | NM_024792 | -0,70 | 5,40E-06 |
| STC2 | NM_003714 | -0,62 | 5,42E-06 |
| ZNF652 | NM_001145365 | 0,85 | 5,44E-06 |
| ANGPTL5 | NM_178127 | 3,50 | 5,46E-06 |
| MDN1 | NM_014611 | -0,91 | 5,47E-06 |
| ACCN3 | NM_004769 | 2,03 | 5,50E-06 |
| BBX | NM_020235 | 0,74 | 5,51E-06 |
| TAF13 | NM_005645 | -1,22 | 5,52E-06 |
| DYM | NM_017653 | 0,62 | 5,57E-06 |
| C5orf42 | NM_023073 | 0,93 | 5,57E-06 |
| RAI2 | NM_021785 | 2,47 | 5,62E-06 |
| COX17 | NM_005694 | -1,53 | 5,64E-06 |
| TM4SF1 | NM_014220 | -0,67 | 5,65E-06 |
| GATAD1 | NM_021167 | 0,69 | 5,70E-06 |
| FN3KRP | NM_024619 | -0,90 | 5,70E-06 |
| VPS13B | NM_152564 | 0,92 | 5,74E-06 |
| SLC22A18AS | NM_007105 | 2,16 | 5,82E-06 |
| NFKBIB | NM_001001716 | -0,93 | 5,83E-06 |
| POLR1C | NM_004875 | -1,15 | 5,84E-06 |
| DHX38 | NM_014003 | -0,77 | 5,84E-06 |
| TOP1 | NM_003286 | -0,66 | 5,87E-06 |
| CYP4V2 | NM_207352 | 1,73 | 5,91E-06 |
| VEGFB | NM_003377 | 0,69 | 5,91E-06 |
| MAPK3 | NM_001109891 | 0,62 | 5,96E-06 |
| MAPKAPK5 | NM_003668 | -0,95 | 5,99E-06 |
| ZNF33A | NM_006974 | 0,99 | 6,06E-06 |
| SNAPC5 | NM_006049 | -1,14 | 6,09E-06 |
| LSM11 | NM_173491 | -1,12 | 6,13E-06 |
| NME1-NME2 | NM_001018136 | -0,68 | 6,16E-06 |
| CD274 | NM_014143 | -2,99 | 6,18E-06 |
| MRM1 | NM_024864 | -1,33 | 6,18E-06 |
| LSM3 | NM_014463 | -0,79 | 6,21E-06 |
| CFI | NM_000204 | -3,17 | 6,22E-06 |
| CHRD | NM_003741 | 2,64 | 6,22E-06 |
| SNX21 | NM_033421 | 0,78 | 6,35E-06 |
| GRB14 | NM_004490 | -2,27 | 6,38E-06 |
| CREBL2 | NM_001310 | 0,77 | 6,40E-06 |
| NDST2 | NM_003635 | 0,79 | 6,44E-06 |
| NAGPA | NM_016256 | 0,80 | 6,44E-06 |
| MEX3A | NM_001093725 | -0,80 | 6,48E-06 |
| PPARA | NM_001001928 | 0,90 | 6,53E-06 |
| NEK10 | NM_199347 | 2,10 | 6,54E-06 |
| WDR91 | NM_014149 | 0,97 | 6,55E-06 |
| DERL1 | NM_001134671 | -0,66 | 6,58E-06 |
| ANKRD13A | NM_033121 | -0,85 | 6,59E-06 |
| C3orf47 | NR_026991 | 1,38 | 6,64E-06 |
| MYL6 | NM_021019 | -0,98 | 6,66E-06 |
| GPN1 | NM_001145047 | -0,73 | 6,85E-06 |
| VPS28 | NM_183057 | 0,74 | 6,86E-06 |
| PAQR8 | NM_133367 | 1,05 | 6,86E-06 |
| STAMBPL1 | NM_020799 | -1,29 | 6,87E-06 |
| DNASE2 | NM_001375 | 0,88 | 6,89E-06 |
| HDHD3 | NM_031219 | 1,44 | 6,93E-06 |
| MAP3K7IP1 | NM_006116 | 0,76 | 6,96E-06 |
| ADAMTS7 | NM_014272 | 0,63 | 7,04E-06 |
| NEIL1 | NM_024608 | 1,86 | 7,12E-06 |
| MERTK | NM_006343 | 1,74 | 7,12E-06 |
| SLC25A25 | NM_001006643 | -0,70 | 7,16E-06 |
| TCN2 | NM_000355 | 1,12 | 7,24E-06 |
| TP53BP2 | NM_005426 | -0,69 | 7,27E-06 |
| KIAA0141 | NM_014773 | 0,63 | 7,37E-06 |
| VTA1 | NM_016485 | -0,67 | 7,39E-06 |
| MAP4 | NM_030885 | -0,76 | 7,40E-06 |
| BBS5 | NM_152384 | 1,02 | 7,43E-06 |
| SMTNL2 | NM_001114974 | 3,34 | 7,43E-06 |
| C1orf71 | NM_152609 | -0,72 | 7,46E-06 |
| ANGPTL4 | NM_139314 | 1,99 | 7,46E-06 |
| BTN2A2 | NM_006995 | 1,31 | 7,54E-06 |
| FANCE | NM_021922 | -0,78 | 7,56E-06 |
| PDE7B | NM_018945 | 1,50 | 7,60E-06 |
| TGIF1 | NM_173208 | 0,90 | 7,60E-06 |
| PLEKHH3 | NM_024927 | 0,88 | 7,60E-06 |
| C21orf70 | NM_058190 | -1,10 | 7,65E-06 |
| TPRKB | NM_016058 | -1,25 | 7,70E-06 |
| ASB2 | NM_016150 | 2,66 | 7,70E-06 |
| L1CAM | NM_000425 | 1,64 | 7,74E-06 |
| SELE | NM_000450 | -3,39 | 7,75E-06 |
| PANX1 | NM_015368 | -0,69 | 7,75E-06 |
| SUV420H2 | NM_032701 | 1,22 | 7,76E-06 |
| LCLAT1 | NM_182551 | -0,86 | 7,89E-06 |
| C3orf34 | NM_032898 | 1,58 | 7,92E-06 |
| AMOTL1 | NM_130847 | 0,61 | 7,93E-06 |
| SEC13 | NM_183352 | -0,63 | 7,96E-06 |
| LPAR6 | NM_001162497 | 1,68 | 8,01E-06 |
| FILIP1L | NM_014890 | -1,04 | 8,07E-06 |
| CD27 | NM_001242 | 2,59 | 8,11E-06 |
| C7orf68 | NM_001098786 | -1,11 | 8,11E-06 |
| DSTYK | NM_015375 | 0,75 | 8,15E-06 |
| MCF2L | NM_024979 | -2,05 | 8,16E-06 |
| AFG3L2 | NM_006796 | -0,65 | 8,30E-06 |
| COMMD2 | NM_016094 | -0,96 | 8,30E-06 |
| APOBEC3G | NM_021822 | 1,83 | 8,33E-06 |
| CHMP1B | NM_020412 | 0,65 | 8,39E-06 |
| ZNF671 | NM_024833 | 1,16 | 8,41E-06 |
| PHF1 | NM_024165 | 0,72 | 8,42E-06 |
| FAM26E | NM_153711 | -0,87 | 8,43E-06 |
| KCTD21 | NM_001029859 | 0,96 | 8,49E-06 |
| ZNF100 | NM_173531 | -1,35 | 8,51E-06 |
| FGFR3 | NM_022965 | -1,26 | 8,55E-06 |
| DOCK4 | NM_014705 | 1,01 | 8,58E-06 |
| HPS5 | NM_181507 | -0,79 | 8,58E-06 |
| HPS3 | NM_032383 | 0,72 | 8,66E-06 |
| CCRK | NM_001039803 | 1,39 | 8,67E-06 |
| CNTNAP3 | NM_033655 | 1,41 | 8,68E-06 |
| MAPK1IP1L | NM_144578 | -0,86 | 8,69E-06 |
| PRPF40B | NM_012272 | 0,75 | 8,75E-06 |
| KLC2 | NM_022822 | -0,76 | 8,76E-06 |
| TNFAIP1 | NM_021137 | -0,60 | 8,82E-06 |
| RUNX1T1 | NM_175636 | 2,22 | 8,88E-06 |
| WDSUB1 | NM_001128213 | 1,24 | 9,07E-06 |
| C2orf42 | NM_017880 | 1,01 | 9,10E-06 |
| CCDC124 | NM_001136203 | -0,60 | 9,10E-06 |
| C11orf60 | NM_020153 | 1,06 | 9,13E-06 |
| ProSAPiP1 | NM_014731 | 0,98 | 9,17E-06 |
| SESTD1 | NM_178123 | 0,65 | 9,17E-06 |
| CYLD | NM_015247 | 0,71 | 9,22E-06 |
| NPAS3 | NM_001164749 | 2,86 | 9,22E-06 |
| LOC550643 | NR_015367 | 0,68 | 9,34E-06 |
| PDE4A | NM_001111307 | 0,95 | 9,36E-06 |
| TBC1D3H | NM_001123392 | 1,51 | 9,41E-06 |
| EGFLAM | NM_182801 | 1,03 | 9,52E-06 |
| FAM86A | NM_201598 | -0,78 | 9,53E-06 |
| RAP2B | NM_002886 | 0,67 | 9,56E-06 |
| IL1RAP | NM_002182 | -1,03 | 9,65E-06 |
| TMEM67 | NM_153704 | 1,38 | 9,74E-06 |
| PSMD13 | NM_002817 | -0,65 | 9,78E-06 |
| AVIL | NM_006576 | 2,19 | 9,90E-06 |
| MED17 | NM_004268 | -0,77 | 9,91E-06 |
| C6orf226 | NM_001008739 | 1,61 | 9,93E-06 |
| TMTC1 | NM_175861 | 1,15 | 9,95E-06 |
| SCUBE3 | NM_152753 | -0,78 | 9,98E-06 |
| TFAM | NM_003201 | -1,10 | 9,98E-06 |
| CCDC43 | NM_144609 | -0,76 | 9,98E-06 |
| BUD13 | NM_001159736 | -0,83 | 9,98E-06 |
| TRAF4 | NM_004295 | -0,63 | 1,00E-05 |
| DGCR11 | NR_024157 | -1,40 | 1,02E-05 |
| CENPQ | NM_018132 | -1,59 | 1,02E-05 |
| COG4 | NM_015386 | 0,61 | 1,03E-05 |
| CDK5RAP3 | NM_176096 | 0,88 | 1,05E-05 |
| RAD1 | NM_002853 | -0,76 | 1,06E-05 |
| FECH | NM_000140 | 0,75 | 1,07E-05 |
| ZCCHC6 | NM_024617 | 0,68 | 1,07E-05 |
| C16orf63 | NM_144600 | -0,64 | 1,07E-05 |
| TKT | NM_001064 | 0,72 | 1,07E-05 |
| MUM1L1 | NM_152423 | 2,20 | 1,07E-05 |
| CTU2 | NM_001012759 | -0,98 | 1,10E-05 |
| ATP2A3 | NM_005173 | 2,64 | 1,10E-05 |
| C11orf54 | NM_014039 | 0,99 | 1,10E-05 |
| LOC100131434 | NR_027455 | 2,65 | 1,10E-05 |
| C6orf134 | NM_024909 | 0,99 | 1,10E-05 |
| SOCS2 | NM_003877 | -1,53 | 1,11E-05 |
| POLR3GL | NM_032305 | 0,94 | 1,11E-05 |
| AURKAIP1 | NM_001127230 | -0,82 | 1,11E-05 |
| CCDC28A | NM_015439 | 1,37 | 1,12E-05 |
| CRYL1 | NM_015974 | 1,29 | 1,13E-05 |
| PLCB4 | NM_182797 | -0,98 | 1,14E-05 |
| TMEM177 | NM_001105198 | -1,14 | 1,14E-05 |
| SYF2 | NM_015484 | 0,86 | 1,14E-05 |
| CROCCL1 | NR_026752 | 1,02 | 1,15E-05 |
| TMEM198 | NM_001005209 | 1,74 | 1,15E-05 |
| TOB1 | NM_005749 | 0,91 | 1,15E-05 |
| EEF1A2 | NM_001958 | 1,99 | 1,16E-05 |
| CAMK2B | NM_001220 | 3,26 | 1,16E-05 |
| BAT2 | NM_080686 | -0,66 | 1,16E-05 |
| MRPL52 | NM_181304 | -0,88 | 1,17E-05 |
| RBM17 | NM_001145547 | -0,68 | 1,17E-05 |
| TGS1 | NM_024831 | -0,78 | 1,18E-05 |
| ZNF22 | NM_006963 | -0,87 | 1,19E-05 |
| ATG16L2 | NM_033388 | 1,43 | 1,19E-05 |
| TIMM22 | NM_013337 | -0,84 | 1,19E-05 |
| ZNF322A | NM_024639 | 0,90 | 1,19E-05 |
| FAM54B | NM_001099626 | 0,65 | 1,19E-05 |
| C10orf104 | NM_173473 | 0,71 | 1,20E-05 |
| FER1L6 | NM_001039112 | 3,60 | 1,20E-05 |
| SLC2A1 | NM_006516 | -0,78 | 1,23E-05 |
| MAD1L1 | NM_003550 | -1,04 | 1,24E-05 |
| SELM | NM_080430 | 0,80 | 1,25E-05 |
| OSBPL11 | NM_022776 | -0,68 | 1,27E-05 |
| ARD1A | NM_003491 | -0,68 | 1,28E-05 |
| COL18A1 | NM_130444 | 0,63 | 1,28E-05 |
| MPZ | NM_000530 | 1,94 | 1,32E-05 |
| RHOD | NM_014578 | 1,67 | 1,32E-05 |
| LY6E | NM_002346 | 0,77 | 1,33E-05 |
| ATF7IP | NM_018179 | 0,74 | 1,34E-05 |
| COL22A1 | NM_152888 | 1,72 | 1,36E-05 |
| ARID3A | NM_005224 | 0,90 | 1,36E-05 |
| FLAD1 | NM_025207 | -0,78 | 1,36E-05 |
| SAMD11 | NM_152486 | -0,90 | 1,37E-05 |
| SNAP29 | NM_004782 | 0,61 | 1,37E-05 |
| ALPK3 | NM_020778 | -1,22 | 1,38E-05 |
| C9orf150 | NM_203403 | 1,13 | 1,38E-05 |
| ZNF585B | NM_152279 | 1,73 | 1,39E-05 |
| BRCC3 | NM_024332 | -0,95 | 1,40E-05 |
| TICAM1 | NM_182919 | -0,79 | 1,42E-05 |
| PRICKLE4 | NM_013397 | 1,39 | 1,42E-05 |
| GTF2F2 | NM_004128 | -0,79 | 1,43E-05 |
| SERPINF1 | NM_002615 | 1,72 | 1,43E-05 |
| KIAA1217 | NM_001098501 | 1,11 | 1,45E-05 |
| ZNF117 | NM_015852 | 1,01 | 1,45E-05 |
| KDM6B | NM_001080424 | 0,63 | 1,45E-05 |
| BMP4 | NM_130850 | -1,50 | 1,45E-05 |
| USP9Y | NM_004654 | 0,70 | 1,47E-05 |
| CASP9 | NM_001229 | 1,08 | 1,47E-05 |
| ZDHHC13 | NM_019028 | -1,06 | 1,49E-05 |
| XYLB | NM_005108 | -1,28 | 1,52E-05 |
| PTPN3 | NM_002829 | -0,81 | 1,52E-05 |
| AVEN | NM_020371 | -0,95 | 1,53E-05 |
| DNAH10 | NM_207437 | 1,71 | 1,53E-05 |
| C14orf49 | NM_152592 | 0,90 | 1,57E-05 |
| CYTH4 | NM_013385 | 3,22 | 1,57E-05 |
| LOC541471 | NR_015395 | -0,95 | 1,60E-05 |
| DEAF1 | NM_021008 | 0,68 | 1,62E-05 |
| HDGF | NM_001126050 | -0,66 | 1,62E-05 |
| AGER | NM_001136 | 1,27 | 1,62E-05 |
| SFRS17A | NR_027383 | 0,63 | 1,62E-05 |
| HEPH | NM_001130860 | 1,69 | 1,63E-05 |
| NAALAD2 | NM_005467 | 2,13 | 1,63E-05 |
| NRF1 | NM_005011 | -0,84 | 1,63E-05 |
| TSPYL5 | NM_033512 | -0,72 | 1,64E-05 |
| LY75 | NM_002349 | 2,22 | 1,65E-05 |
| ZNF83 | NM_001105549 | 0,83 | 1,66E-05 |
| APPBP2 | NM_006380 | 0,78 | 1,70E-05 |
| PTCH2 | NM_001166292 | 2,46 | 1,70E-05 |
| RPA2 | NM_002946 | -0,91 | 1,71E-05 |
| THOC3 | NM_032361 | -1,13 | 1,72E-05 |
| MYST1 | NM_182958 | 0,92 | 1,72E-05 |
| ADPRHL2 | NM_017825 | -0,80 | 1,73E-05 |
| GPR77 | NM_018485 | 3,37 | 1,74E-05 |
| USP35 | NM_020798 | 0,78 | 1,74E-05 |
| PHLPP2 | NM_015020 | -0,79 | 1,74E-05 |
| CHST15 | NM_015892 | 0,88 | 1,77E-05 |
| NKRF | NM_017544 | -0,76 | 1,79E-05 |
| ST8SIA2 | NM_006011 | -1,96 | 1,81E-05 |
| SEPSECS | NM_001159728 | 1,07 | 1,81E-05 |
| PPFIA2 | NM_003625 | 1,78 | 1,81E-05 |
| HMGCL | NM_000191 | 0,85 | 1,81E-05 |
| RNF146 | NM_030963 | 0,66 | 1,82E-05 |
| SNTA1 | NM_003098 | 0,98 | 1,82E-05 |
| SLIT2 | NM_004787 | -0,72 | 1,84E-05 |
| ZNF788 | NR_027049 | -1,33 | 1,84E-05 |
| FAH | NM_000137 | 0,94 | 1,86E-05 |
| CROT | NM_021151 | 1,14 | 1,88E-05 |
| LCMT2 | NM_014793 | -0,91 | 1,88E-05 |
| ZNF333 | NM_032433 | 0,93 | 1,89E-05 |
| TMEM194B | NM_001142645 | -1,19 | 1,89E-05 |
| PPP2R5E | NM_006246 | -0,61 | 1,89E-05 |
| STARD3 | NM_006804 | 0,66 | 1,90E-05 |
| PSME1 | NM_176783 | 0,80 | 1,90E-05 |
| C14orf156 | NM_031210 | -1,06 | 1,90E-05 |
| KLHL6 | NM_130446 | -3,55 | 1,90E-05 |
| ZNF354A | NM_005649 | 0,97 | 1,90E-05 |
| HEYL | NM_014571 | -1,29 | 1,94E-05 |
| TECTA | NM_005422 | 3,06 | 1,95E-05 |
| VEPH1 | NM_024621 | -1,55 | 1,96E-05 |
| CHAD | NM_001267 | 3,53 | 1,96E-05 |
| NPC1L1 | NM_013389 | 3,53 | 1,96E-05 |
| FKBP7 | NM_001135212 | 0,77 | 1,98E-05 |
| LOC100128288 | NR_024447 | 2,04 | 1,98E-05 |
| TNRC6B | NM_001024843 | 0,71 | 2,00E-05 |
| SERPINA9 | NM_175739 | -3,19 | 2,00E-05 |
| APLP1 | NM_005166 | 0,73 | 2,00E-05 |
| LOC100133161 | NR_028326 | 1,27 | 2,02E-05 |
| POM121 | NM_172020 | -0,69 | 2,02E-05 |
| ANXA3 | NM_005139 | -2,30 | 2,02E-05 |
| C1GALT1 | NM_020156 | -0,85 | 2,02E-05 |
| ZNF10 | NM_015394 | 1,29 | 2,02E-05 |
| LRRC28 | NM_144598 | 1,13 | 2,05E-05 |
| BRD8 | NM_006696 | 0,66 | 2,06E-05 |
| FAM125B | NM_033446 | 0,99 | 2,06E-05 |
| GUCY1A3 | NM_001130683 | 2,08 | 2,06E-05 |
| MRPL19 | NM_014763 | -0,63 | 2,07E-05 |
| UBE2J2 | NM_194315 | -0,76 | 2,07E-05 |
| ZNF280D | NM_001002843 | 0,90 | 2,09E-05 |
| MED27 | NM_004269 | -0,88 | 2,09E-05 |
| PRPF38A | NM_032864 | -0,65 | 2,10E-05 |
| C8orf42 | NM_175075 | 1,54 | 2,11E-05 |
| C1orf212 | NM_001164825 | -0,87 | 2,11E-05 |
| SLC35A2 | NM_005660 | -0,71 | 2,12E-05 |
| MIS12 | NM_024039 | -0,83 | 2,12E-05 |
| LOC441666 | NR_024380 | -2,20 | 2,13E-05 |
| C3orf23 | NM_173826 | 1,05 | 2,13E-05 |
| ESPNL | NM_194312 | 1,48 | 2,14E-05 |
| FBXO3 | NM_012175 | 0,76 | 2,16E-05 |
| C15orf63 | NM_016400 | -1,05 | 2,17E-05 |
| TMEM41A | NM_080652 | -0,88 | 2,19E-05 |
| CLDND1 | NM_001040199 | -0,65 | 2,19E-05 |
| PPAPDC2 | NM_203453 | 1,18 | 2,20E-05 |
| UBTD1 | NM_024954 | 0,68 | 2,21E-05 |
| AMMECR1 | NM_015365 | -0,71 | 2,22E-05 |
| CALML6 | NM_138705 | 2,49 | 2,25E-05 |
| DNAJC28 | NM_001040192 | 2,40 | 2,26E-05 |
| SYT12 | NM_177963 | 2,74 | 2,27E-05 |
| C1orf128 | NM_020362 | -0,76 | 2,27E-05 |
| VANGL1 | NM_138959 | -0,81 | 2,28E-05 |
| LAT | NM_014387 | 1,33 | 2,30E-05 |
| C5orf53 | NM_001007189 | 1,30 | 2,32E-05 |
| GPR3 | NM_005281 | -1,63 | 2,33E-05 |
| RWDD4A | NM_152682 | -0,70 | 2,33E-05 |
| PDXDC2 | NR_003610 | 0,89 | 2,33E-05 |
| LOC643387 | NR_026923 | -1,28 | 2,34E-05 |
| HEMK1 | NM_016173 | 0,78 | 2,35E-05 |
| CREB3L4 | NM_130898 | 1,24 | 2,36E-05 |
| POLR3F | NM_006466 | -1,05 | 2,36E-05 |
| RWDD2A | NM_033411 | 1,19 | 2,37E-05 |
| SHANK2 | NM_012309 | -2,02 | 2,37E-05 |
| DENND2A | NM_015689 | 1,19 | 2,37E-05 |
| AGAP1 | NM_001037131 | -0,64 | 2,37E-05 |
| TRABD | NM_025204 | -0,64 | 2,38E-05 |
| FAM135A | NM_001162529 | 1,00 | 2,43E-05 |
| GGA1 | NM_001001561 | 1,14 | 2,43E-05 |
| ATRNL1 | NM_207303 | 1,16 | 2,44E-05 |
| HSPBP1 | NM_012267 | -0,71 | 2,44E-05 |
| LRBA | NM_006726 | 0,78 | 2,45E-05 |
| RYBP | NM_012234 | 0,69 | 2,46E-05 |
| PHKA2 | NM_000292 | 0,81 | 2,48E-05 |
| Magmas | NM_016069 | -1,30 | 2,48E-05 |
| KTI12 | NM_138417 | -0,89 | 2,49E-05 |
| RNF6 | NM_183044 | -0,66 | 2,51E-05 |
| GTF3C4 | NM_012204 | -0,67 | 2,56E-05 |
| VHL | NM_000551 | 0,61 | 2,56E-05 |
| CPNE2 | NM_152727 | -1,06 | 2,58E-05 |
| LTBP2 | NM_000428 | 0,67 | 2,59E-05 |
| MAP3K5 | NM_005923 | -0,73 | 2,59E-05 |
| ALK | NM_004304 | 3,49 | 2,61E-05 |
| SNRPN | NM_022806 | 0,80 | 2,62E-05 |
| SNURF | NM_005678 | 0,80 | 2,62E-05 |
| ZNF837 | NM_001129730 | 1,85 | 2,64E-05 |
| NCRNA00201 | NR_026778 | -0,61 | 2,67E-05 |
| MDC1 | NM_014641 | -0,91 | 2,68E-05 |
| ZNF224 | NM_013398 | 1,07 | 2,69E-05 |
| ZMYM3 | NM_201599 | 0,61 | 2,69E-05 |
| PKNOX1 | NM_004571 | -0,84 | 2,71E-05 |
| KIF16B | NM_024704 | 1,06 | 2,71E-05 |
| EPC2 | NM_015630 | 0,84 | 2,72E-05 |
| MRPL47 | NM_020409 | -0,90 | 2,73E-05 |
| ATP6V1B1 | NM_001692 | 2,27 | 2,73E-05 |
| LOC541473 | NR_003602 | 2,10 | 2,78E-05 |
| C6orf72 | NM_138785 | 0,69 | 2,80E-05 |
| GFOD1 | NM_018988 | -2,42 | 2,81E-05 |
| GYG2 | NM_003918 | 2,89 | 2,83E-05 |
| LOC375190 | NM_001145710 | 2,16 | 2,85E-05 |
| SLC5A2 | NM_003041 | 0,85 | 2,86E-05 |
| NCRNA00152 | NR_024204 | -1,21 | 2,86E-05 |
| PDIA3P | NR_002305 | -0,70 | 2,88E-05 |
| TRPM7 | NM_017672 | 0,65 | 2,89E-05 |
| PRDX4 | NM_006406 | -0,67 | 2,92E-05 |
| IRAK2 | NM_001570 | 0,87 | 2,93E-05 |
| ZFAND6 | NM_019006 | 0,75 | 2,93E-05 |
| CXCL3 | NM_002090 | -3,05 | 2,94E-05 |
| AGFG1 | NM_001135188 | -0,68 | 2,96E-05 |
| C14orf167 | NR_023921 | 0,94 | 2,96E-05 |
| AATF | NM_012138 | -0,73 | 2,97E-05 |
| CXCR4 | NM_001008540 | -1,88 | 2,98E-05 |
| FTHL3P | NR_002201 | 1,34 | 2,98E-05 |
| ZNF415 | NM_018355 | 1,95 | 2,98E-05 |
| KIAA1632 | NM_020964 | -0,63 | 3,00E-05 |
| C19orf51 | NM_178837 | 2,50 | 3,01E-05 |
| ABHD11 | NM_001145364 | -0,90 | 3,03E-05 |
| ETFB | NM_001014763 | 1,11 | 3,03E-05 |
| DNAJC2 | NM_014377 | -0,80 | 3,05E-05 |
| JOSD2 | NM_138334 | 0,95 | 3,06E-05 |
| SNHG7 | NR_024543 | 0,81 | 3,07E-05 |
| FAM13C | NM_198215 | 2,18 | 3,07E-05 |
| AK3 | NM_016282 | 0,72 | 3,08E-05 |
| POU6F1 | NR_026893 | 1,19 | 3,08E-05 |
| ZCCHC7 | NM_032226 | 0,74 | 3,09E-05 |
| LIFR | NM_001127671 | 0,71 | 3,11E-05 |
| ARMCX4 | NR_028407 | 1,18 | 3,13E-05 |
| DMXL1 | NM_005509 | 0,71 | 3,14E-05 |
| CCDC146 | NM_020879 | 2,27 | 3,15E-05 |
| TRIM14 | NM_014788 | -1,62 | 3,19E-05 |
| FUT10 | NM_032664 | 1,07 | 3,20E-05 |
| TNFSF18 | NM_005092 | -3,29 | 3,22E-05 |
| TMEFF2 | NM_016192 | -1,12 | 3,22E-05 |
| ECHDC2 | NM_018281 | 1,67 | 3,23E-05 |
| HCLS1 | NM_005335 | -1,99 | 3,24E-05 |
| GPT | NM_005309 | 2,03 | 3,27E-05 |
| EEF1A1 | NM_001402 | 0,65 | 3,27E-05 |
| C1orf228 | NM_001145636 | 3,47 | 3,27E-05 |
| CHST6 | NM_021615 | -2,30 | 3,27E-05 |
| F10 | NM_000504 | 2,13 | 3,28E-05 |
| GRAP | NM_006613 | -2,66 | 3,30E-05 |
| INF2 | NM_032714 | -0,61 | 3,33E-05 |
| CDC42EP2 | NM_006779 | -0,81 | 3,33E-05 |
| AIDA | NM_022831 | -0,66 | 3,36E-05 |
| TMEM63B | NM_018426 | 0,65 | 3,36E-05 |
| SLC38A4 | NM_018018 | 2,98 | 3,40E-05 |
| CCDC85C | NM_001144995 | -0,76 | 3,40E-05 |
| C18orf25 | NM_145055 | -0,64 | 3,41E-05 |
| TTC28 | NM_001145418 | 0,75 | 3,44E-05 |
| TSEN15 | NM_052965 | -0,83 | 3,45E-05 |
| PSMC5 | NM_002805 | -0,74 | 3,45E-05 |
| MYEF2 | NM_016132 | -0,89 | 3,47E-05 |
| DISP2 | NM_033510 | 1,58 | 3,49E-05 |
| GUF1 | NM_021927 | -0,75 | 3,50E-05 |
| RAC3 | NM_005052 | -0,89 | 3,52E-05 |
| ZNF287 | NM_020653 | 1,24 | 3,55E-05 |
| BLVRB | NM_000713 | 0,88 | 3,58E-05 |
| OR2A20P | NR_002158 | 1,65 | 3,58E-05 |
| FAM115C | NM_001130025 | -1,37 | 3,59E-05 |
| PLEKHO1 | NM_016274 | -0,72 | 3,60E-05 |
| GNB1L | NM_053004 | -1,27 | 3,60E-05 |
| IFFO2 | NM_001136265 | -0,74 | 3,61E-05 |
| UNKL | NM_001037125 | 1,38 | 3,61E-05 |
| CCNH | NM_001239 | -0,82 | 3,61E-05 |
| BCL2 | NM_000633 | -1,43 | 3,63E-05 |
| ABCA5 | NM_172232 | 1,32 | 3,69E-05 |
| ERN2 | NM_033266 | -2,14 | 3,72E-05 |
| DBF4B | NM_145663 | -1,37 | 3,73E-05 |
| UNC119 | NM_054035 | 0,69 | 3,74E-05 |
| ANTXR1 | NM_032208 | 0,68 | 3,74E-05 |
| OS9 | NM_001017958 | 0,74 | 3,74E-05 |
| MYCL1 | NM_001033082 | -3,26 | 3,74E-05 |
| WRNIP1 | NM_020135 | -0,62 | 3,75E-05 |
| ARHGAP27 | NM_001159330 | 1,28 | 3,76E-05 |
| EPS8L2 | NM_022772 | 0,71 | 3,78E-05 |
| CD34 | NM_001773 | -2,91 | 3,79E-05 |
| CSF2RB | NM_000395 | -3,26 | 3,80E-05 |
| PACSIN3 | NM_016223 | -0,85 | 3,80E-05 |
| SLC24A6 | NM_024959 | 0,80 | 3,81E-05 |
| GLIPR2 | NM_022343 | -0,80 | 3,82E-05 |
| SLC43A1 | NM_003627 | 1,00 | 3,84E-05 |
| GAR1 | NM_018983 | -1,04 | 3,85E-05 |
| SIRT5 | NM_012241 | 1,01 | 3,88E-05 |
| C20orf199 | NR_003605 | 0,74 | 3,89E-05 |
| SNRPB2 | NM_003092 | -0,66 | 3,93E-05 |
| ARSD | NM_001669 | 0,86 | 3,94E-05 |
| TRAF2 | NM_021138 | -0,85 | 3,95E-05 |
| CYC1 | NM_001916 | -0,64 | 3,95E-05 |
| PURG | NM_001015508 | 2,02 | 3,97E-05 |
| TP53I3 | NM_147184 | 0,71 | 3,97E-05 |
| TTC39B | NM_152574 | 1,31 | 3,97E-05 |
| NTN5 | NM_145807 | 2,99 | 4,00E-05 |
| CHST7 | NM_019886 | -0,86 | 4,01E-05 |
| CACNB3 | NM_000725 | 0,64 | 4,03E-05 |
| ALG8 | NM_024079 | -0,87 | 4,04E-05 |
| RASSF4 | NM_032023 | 1,45 | 4,05E-05 |
| PIK3R1 | NM_181504 | 0,69 | 4,06E-05 |
| AKAP3 | NM_006422 | 3,25 | 4,08E-05 |
| LUZP2 | NM_001009909 | -1,48 | 4,10E-05 |
| ACTR1A | NM_005736 | -0,61 | 4,12E-05 |
| CDC14B | NM_003671 | 0,84 | 4,14E-05 |
| GALNTL2 | NM_054110 | 1,94 | 4,17E-05 |
| IFI35 | NM_005533 | 1,27 | 4,18E-05 |
| KIAA0495 | NM_207306 | 0,77 | 4,19E-05 |
| RCL1 | NM_005772 | -0,72 | 4,25E-05 |
| GPR108 | NM_020171 | 1,01 | 4,25E-05 |
| C13orf34 | NM_024808 | -1,22 | 4,33E-05 |
| LOC100131691 | NR_027334 | 1,55 | 4,35E-05 |
| EMILIN1 | NM_007046 | 0,78 | 4,35E-05 |
| DLL4 | NM_019074 | -2,68 | 4,38E-05 |
| BICC1 | NM_001080512 | 0,66 | 4,40E-05 |
| TFAP2C | NM_003222 | 0,84 | 4,40E-05 |
| MRAP2 | NM_138409 | 1,82 | 4,41E-05 |
| SLC25A43 | NM_145305 | -0,78 | 4,46E-05 |
| LFNG | NM_001040167 | 1,08 | 4,55E-05 |
| SFRS2B | NM_032102 | 0,73 | 4,57E-05 |
| NPM3 | NM_006993 | -1,00 | 4,58E-05 |
| FASTKD5 | NM_021826 | -0,87 | 4,59E-05 |
| SNRNP27 | NM_006857 | -0,69 | 4,60E-05 |
| MFSD5 | NM_032889 | -0,66 | 4,60E-05 |
| NAPG | NM_003826 | -0,61 | 4,61E-05 |
| ATF6 | NM_007348 | -0,70 | 4,69E-05 |
| KLF16 | NM_031918 | -0,74 | 4,73E-05 |
| VPS29 | NM_057180 | -0,62 | 4,74E-05 |
| PHLDA3 | NM_012396 | 0,67 | 4,79E-05 |
| C4orf32 | NM_152400 | -1,22 | 4,86E-05 |
| YKT6 | NM_006555 | -0,63 | 4,88E-05 |
| MMP25 | NM_022468 | 1,87 | 4,89E-05 |
| ST20 | NM_001100879 | 1,87 | 4,94E-05 |
| MSTO1 | NM_018116 | -0,73 | 4,94E-05 |
| ABCC6 | NM_001171 | 2,95 | 4,95E-05 |
| ANAPC7 | NM_016238 | -0,64 | 4,96E-05 |
| SF3B4 | NM_005850 | -0,96 | 4,97E-05 |
| GIMAP8 | NM_175571 | -2,72 | 5,00E-05 |
| TBC1D3G | NM_001040282 | 1,49 | 5,00E-05 |
| FAM136A | NM_032822 | -0,67 | 5,02E-05 |
| MGAT3 | NM_001098270 | 1,81 | 5,02E-05 |
| HIST1H2BK | NM_080593 | 1,07 | 5,02E-05 |
| TTC21A | NM_145755 | 1,70 | 5,02E-05 |
| WDR24 | NM_032259 | 0,68 | 5,02E-05 |
| ZNF836 | NM_001102657 | 1,55 | 5,03E-05 |
| PEG3 | NM_001146187 | 2,77 | 5,04E-05 |
| TUBGCP4 | NM_014444 | -0,91 | 5,05E-05 |
| TAF9 | NM_016283 | -0,85 | 5,05E-05 |
| E2F2 | NM_004091 | -1,67 | 5,05E-05 |
| GADD45GIP1 | NM_052850 | -0,81 | 5,11E-05 |
| AGK | NM_018238 | -0,70 | 5,12E-05 |
| CADPS2 | NM_017954 | 1,26 | 5,13E-05 |
| TRPV1 | NM_018727 | 1,62 | 5,14E-05 |
| NDUFAF2 | NM_174889 | -1,36 | 5,19E-05 |
| ERICH1 | NM_207332 | -1,00 | 5,20E-05 |
| C3orf62 | NM_198562 | 1,47 | 5,20E-05 |
| KHNYN | NM_015299 | 0,60 | 5,22E-05 |
| ARL6IP6 | NR_024526 | -0,89 | 5,26E-05 |
| ISLR2 | NM_001130136 | 2,05 | 5,27E-05 |
| PCBP1 | NM_006196 | -0,61 | 5,34E-05 |
| GALNT4 | NM_003774 | -0,89 | 5,37E-05 |
| DLG2 | NM_001142699 | 3,39 | 5,37E-05 |
| GSDMB | NM_001165959 | 1,21 | 5,37E-05 |
| C6orf70 | NM_018341 | 1,08 | 5,42E-05 |
| TOR3A | NM_022371 | -0,81 | 5,43E-05 |
| ZNF281 | NM_012482 | -0,75 | 5,48E-05 |
| MRPL10 | NM_145255 | 0,60 | 5,48E-05 |
| TTC33 | NM_012382 | 0,84 | 5,50E-05 |
| S100A13 | NM_001024210 | 1,06 | 5,52E-05 |
| LOC653501 | NR_003528 | 1,58 | 5,53E-05 |
| ZNF658B | NR_027861 | 1,58 | 5,53E-05 |
| AARS2 | NM_020745 | -0,78 | 5,56E-05 |
| ZNF207 | NM_001032293 | -0,83 | 5,66E-05 |
| CCDC8 | NM_032040 | 0,75 | 5,70E-05 |
| KCNMB4 | NM_014505 | 2,16 | 5,71E-05 |
| TCEAL7 | NM_152278 | 0,95 | 5,73E-05 |
| ZNF596 | NM_001042416 | 1,68 | 5,73E-05 |
| C1GALT1C1 | NM_152692 | -0,81 | 5,74E-05 |
| PSD2 | NM_032289 | 2,85 | 5,74E-05 |
| ARNT | NM_178426 | 0,68 | 5,76E-05 |
| CLCN2 | NM_004366 | 1,30 | 5,83E-05 |
| ALG9 | NM_024740 | -0,65 | 5,85E-05 |
| ZNF397 | NM_001135178 | 1,05 | 5,85E-05 |
| OSR2 | NM_001142462 | 1,51 | 5,89E-05 |
| EFTUD1 | NM_001040610 | -0,65 | 5,89E-05 |
| HIST1H2BD | NM_138720 | 1,95 | 5,94E-05 |
| PRSS27 | NM_031948 | 2,54 | 5,95E-05 |
| ZNF444 | NM_018337 | 0,75 | 5,97E-05 |
| C14orf101 | NM_017799 | 0,80 | 5,98E-05 |
| CCR7 | NM_001838 | 2,46 | 6,00E-05 |
| IMAA | NR_002594 | -1,71 | 6,05E-05 |
| TET3 | NM_144993 | 0,66 | 6,10E-05 |
| SPOCK2 | NM_014767 | 2,74 | 6,12E-05 |
| EGFL8 | NM_030652 | 1,03 | 6,13E-05 |
| MRPS23 | NM_016070 | -0,97 | 6,19E-05 |
| KIAA1267 | NM_015443 | 0,65 | 6,19E-05 |
| DTD1 | NM_080820 | -0,83 | 6,20E-05 |
| RALGPS1 | NM_014636 | 1,52 | 6,20E-05 |
| KLHDC1 | NM_172193 | 2,19 | 6,20E-05 |
| KIAA2026 | NM_001017969 | 0,91 | 6,22E-05 |
| GAS8 | NM_001481 | 0,89 | 6,31E-05 |
| CCT6B | NM_006584 | 2,46 | 6,32E-05 |
| CRLF3 | NM_015986 | -0,85 | 6,49E-05 |
| CAMK1D | NM_153498 | 1,19 | 6,50E-05 |
| EMX2 | NM_004098 | 1,17 | 6,50E-05 |
| PHF5A | NM_032758 | -0,82 | 6,52E-05 |
| ZNF345 | NM_003419 | 1,86 | 6,53E-05 |
| CIAPIN1 | NM_020313 | -0,75 | 6,53E-05 |
| ITPRIPL1 | NM_001163523 | -1,73 | 6,59E-05 |
| SBF2 | NM_030962 | 0,66 | 6,73E-05 |
| MLL2 | NM_003482 | -0,72 | 6,90E-05 |
| PDE2A | NR_026572 | -2,90 | 6,90E-05 |
| CYB5R2 | NM_016229 | 1,29 | 6,91E-05 |
| FHDC1 | NM_033393 | 2,54 | 6,92E-05 |
| NF1 | NM_001042492 | 0,65 | 6,93E-05 |
| EXOC3 | NM_007277 | 0,65 | 6,94E-05 |
| N4BP2L1 | NM_052818 | 2,55 | 6,94E-05 |
| SLC9A3R2 | NM_001130012 | -0,69 | 6,95E-05 |
| HIVEP1 | NM_002114 | -0,73 | 6,95E-05 |
| MAGEH1 | NM_014061 | 0,70 | 6,98E-05 |
| GALNT3 | NM_004482 | -1,45 | 6,99E-05 |
| ARHGEF5 | NM_005435 | 1,44 | 7,12E-05 |
| SNX2 | NM_003100 | 0,68 | 7,19E-05 |
| MMAB | NM_052845 | -0,91 | 7,20E-05 |
| RPP25 | NM_017793 | -1,09 | 7,21E-05 |
| MEF2B | NM_001145785 | 1,18 | 7,22E-05 |
| DNAJC12 | NM_021800 | 1,82 | 7,25E-05 |
| MAP6D1 | NM_024871 | -1,16 | 7,25E-05 |
| LOC221710 | NM_001135575 | -0,67 | 7,27E-05 |
| SMN1 | NM_000344 | -0,80 | 7,29E-05 |
| SMN2 | NM_017411 | -0,80 | 7,29E-05 |
| FAM179B | NM_015091 | 0,74 | 7,29E-05 |
| LOC728743 | NR_027237 | 1,38 | 7,32E-05 |
| CRHBP | NM_001882 | 2,84 | 7,32E-05 |
| LOC728613 | NR_003713 | 1,42 | 7,32E-05 |
| GALNT14 | NM_024572 | -2,11 | 7,33E-05 |
| TMED5 | NM_016040 | -0,66 | 7,34E-05 |
| BHLHB9 | NM_001142525 | 1,04 | 7,34E-05 |
| TSEN2 | NM_025265 | -1,13 | 7,39E-05 |
| GALK1 | NM_000154 | 0,81 | 7,44E-05 |
| C6orf97 | NM_025059 | 2,46 | 7,44E-05 |
| CCDC149 | NM_001130726 | 0,99 | 7,45E-05 |
| ZNF526 | NM_133444 | -0,69 | 7,51E-05 |
| ARL4D | NM_001661 | -0,81 | 7,55E-05 |
| FLYWCH1 | NM_032296 | 0,62 | 7,55E-05 |
| OPCML | NM_002545 | -2,23 | 7,61E-05 |
| ZNF804A | NM_194250 | -1,37 | 7,63E-05 |
| LOC202781 | NR_028090 | 1,26 | 7,63E-05 |
| TRAPPC4 | NM_016146 | -0,84 | 7,67E-05 |
| LOC493754 | NR_002933 | -0,80 | 7,68E-05 |
| MTRR | NM_024010 | -0,66 | 7,71E-05 |
| TIMM44 | NM_006351 | -0,62 | 7,72E-05 |
| C9orf173 | NM_001004353 | 2,81 | 7,75E-05 |
| KHSRP | NM_003685 | -0,78 | 7,84E-05 |
| BFSP1 | NM_001195 | 1,53 | 7,89E-05 |
| HUS1 | NM_004507 | -0,88 | 7,92E-05 |
| NACC2 | NM_144653 | -0,71 | 7,93E-05 |
| DUS2L | NM_017803 | -0,99 | 7,94E-05 |
| TNIP1 | NM_006058 | -0,67 | 7,98E-05 |
| SPIN3 | NM_001010862 | 1,03 | 8,00E-05 |
| BCAP29 | NM_001008405 | -0,75 | 8,01E-05 |
| RBM38 | NM_017495 | -0,80 | 8,02E-05 |
| GOT1 | NM_002079 | -0,71 | 8,19E-05 |
| PEX5 | NM_001131023 | 0,76 | 8,22E-05 |
| PTPN4 | NM_002830 | 1,10 | 8,24E-05 |
| EED | NM_152991 | -0,94 | 8,34E-05 |
| SLC25A19 | NM_021734 | -1,31 | 8,37E-05 |
| LOC284440 | NR_026956 | 2,56 | 8,46E-05 |
| THYN1 | NM_014174 | 0,93 | 8,47E-05 |
| BRWD1 | NM_018963 | 0,74 | 8,50E-05 |
| KIAA1009 | NM_014895 | 1,07 | 8,53E-05 |
| HES1 | NM_005524 | -0,65 | 8,55E-05 |
| C1orf231 | NM_001102601 | 1,65 | 8,59E-05 |
| CALM3 | NM_005184 | -0,62 | 8,59E-05 |
| ANKRD23 | NM_144994 | 1,49 | 8,64E-05 |
| C3orf19 | NM_016474 | 0,85 | 8,65E-05 |
| PTENP1 | NR_023917 | 0,88 | 8,66E-05 |
| SFRS5 | NM_006925 | 0,61 | 8,68E-05 |
| VPS37C | NM_017966 | -0,61 | 8,68E-05 |
| TNFSF12 | NM_003809 | 0,89 | 8,72E-05 |
| LOC643763 | NR_027378 | 2,57 | 8,76E-05 |
| NET1 | NM_001047160 | -0,61 | 8,78E-05 |
| PSPH | NM_004577 | -0,68 | 8,81E-05 |
| EIF4E3 | NM_001134650 | 1,22 | 8,95E-05 |
| IPW | NR_023915 | 0,96 | 9,02E-05 |
| NLGN1 | NM_014932 | 1,21 | 9,03E-05 |
| ANGPTL6 | NM_031917 | 1,48 | 9,05E-05 |
| SMURF1 | NM_181349 | -0,64 | 9,06E-05 |
| PRELID2 | NM_138492 | -1,62 | 9,08E-05 |
| NUDT19 | NM_001105570 | -0,76 | 9,08E-05 |
| TRPC1 | NM_003304 | 0,79 | 9,17E-05 |
| TTLL1 | NR_027779 | 0,88 | 9,19E-05 |
| YJEFN3 | NM_198537 | 1,60 | 9,20E-05 |
| NUDT6 | NM_007083 | -1,09 | 9,21E-05 |
| ZBTB48 | NM_005341 | 0,83 | 9,23E-05 |
| BACE2 | NM_012105 | -1,01 | 9,30E-05 |
| NINL | NM_025176 | 1,23 | 9,33E-05 |
| APBA3 | NM_004886 | 0,79 | 9,35E-05 |
| ANKHD1-EIF4EBP3 | NM_020690 | 0,67 | 9,35E-05 |
| ABT1 | NM_013375 | -0,72 | 9,39E-05 |
| DNAH11 | NM_003777 | -0,94 | 9,43E-05 |
| HSPBL2 | NR_024392 | -1,07 | 9,48E-05 |
| ZNF438 | NM_001143771 | 1,12 | 9,49E-05 |
| RRP8 | NM_015324 | -0,82 | 9,50E-05 |
| ATPGD1 | NM_020811 | 2,46 | 9,50E-05 |
| BCKDHA | NM_001164783 | 0,73 | 9,55E-05 |
| CCDC87 | NM_018219 | 2,58 | 9,56E-05 |
| ABCC9 | NM_020297 | -1,43 | 9,65E-05 |
| CDH8 | NM_001796 | -2,07 | 9,67E-05 |
| ADM | NM_001124 | -0,65 | 9,71E-05 |
| TMEM42 | NM_144638 | 1,07 | 9,72E-05 |
| DMGDH | NM_013391 | 3,30 | 9,74E-05 |
| TRMT61B | NM_017910 | -0,92 | 9,76E-05 |
| MGC70857 | NM_001001795 | 0,88 | 9,76E-05 |
| ZNF695 | NM_020394 | -2,36 | 9,83E-05 |
| ORAOV1 | NM_153451 | -0,75 | 9,87E-05 |
| THRB | NM_001128176 | 1,93 | 9,90E-05 |
| LOC401588 | NR_015378 | -1,27 | 9,99E-05 |
| EFHB | NM_144715 | 2,99 | 1,00E-04 |
| EFHD1 | NM_025202 | -1,05 | 1,01E-04 |
| TSNAX-DISC1 | NR_028393 | 0,89 | 1,01E-04 |
| ZDHHC4 | NM_001134389 | 0,66 | 1,01E-04 |
| FUT4 | NM_002033 | -0,88 | 1,01E-04 |
| DIRAS1 | NM_145173 | -1,07 | 1,01E-04 |
| FBXW4 | NM_022039 | 0,70 | 1,01E-04 |
| ACAD8 | NM_014384 | 0,95 | 1,02E-04 |
| ABTB2 | NM_145804 | 1,37 | 1,02E-04 |
| TMEM229B | NM_182526 | 2,76 | 1,02E-04 |
| FBXO17 | NM_024907 | 0,63 | 1,02E-04 |
| C14orf169 | NM_024644 | -0,72 | 1,03E-04 |
| WDR55 | NM_017706 | -0,60 | 1,03E-04 |
| GFAP | NM_001131019 | 3,29 | 1,03E-04 |
| BCOR | NM_001123385 | -0,65 | 1,04E-04 |
| ZBED5 | NM_001143667 | 0,67 | 1,04E-04 |
| TRAF3 | NM_003300 | -0,85 | 1,05E-04 |
| TUBG2 | NM_016437 | 0,70 | 1,05E-04 |
| ASB8 | NM_024095 | 0,67 | 1,06E-04 |
| TBC1D15 | NM_001146214 | 0,63 | 1,07E-04 |
| ELANE | NM_001972 | 3,28 | 1,07E-04 |
| GIMAP1 | NM_130759 | -3,11 | 1,07E-04 |
| ZEB2 | NM_014795 | -0,63 | 1,07E-04 |
| C10orf140 | NM_207371 | 2,31 | 1,07E-04 |
| STARD3NL | NM_032016 | -0,62 | 1,07E-04 |
| ZNF493 | NM_175910 | 1,66 | 1,08E-04 |
| SDHALP2 | NR_003265 | 0,88 | 1,09E-04 |
| TCF7L2 | NM_001146274 | 0,97 | 1,09E-04 |
| TREX1 | NM_033629 | -0,80 | 1,09E-04 |
| COG5 | NM_181733 | 0,62 | 1,10E-04 |
| FBXO41 | NM_001080410 | 1,61 | 1,10E-04 |
| CDK5 | NM_004935 | 0,84 | 1,11E-04 |
| S100A11 | NM_005620 | -0,66 | 1,11E-04 |
| LOC284441 | NR_003128 | -1,29 | 1,11E-04 |
| PPCS | NM_024664 | 0,81 | 1,11E-04 |
| NT5DC1 | NM_152729 | 0,98 | 1,11E-04 |
| ZNF672 | NM_024836 | -0,62 | 1,12E-04 |
| MRPL13 | NM_014078 | -0,79 | 1,12E-04 |
| LANCL1 | NM_001136574 | 0,62 | 1,12E-04 |
| DHDDS | NM_024887 | -0,65 | 1,12E-04 |
| LIG3 | NM_013975 | -0,66 | 1,12E-04 |
| WDFY2 | NM_052950 | -0,64 | 1,12E-04 |
| DDX26B | NM_182540 | 1,14 | 1,12E-04 |
| MFSD6 | NM_017694 | 0,90 | 1,13E-04 |
| SAP30BP | NM_013260 | -0,61 | 1,14E-04 |
| TMEM64 | NM_001008495 | 0,65 | 1,14E-04 |
| FBXO2 | NM_012168 | 2,60 | 1,14E-04 |
| RALGAPA2 | NM_020343 | 0,95 | 1,15E-04 |
| PASK | NM_015148 | -0,92 | 1,17E-04 |
| METTL7B | NM_152637 | 1,69 | 1,17E-04 |
| POTEE | NM_001083538 | -2,73 | 1,18E-04 |
| LOC201651 | NR_026915 | -3,26 | 1,18E-04 |
| ST8SIA1 | NM_003034 | 2,14 | 1,19E-04 |
| NMNAT3 | NM_178177 | 2,73 | 1,20E-04 |
| VPS37A | NM_152415 | -0,63 | 1,20E-04 |
| PGM1 | NM_002633 | -0,63 | 1,20E-04 |
| TRMT1 | NM_001136035 | -0,61 | 1,20E-04 |
| SLC41A2 | NM_032148 | 1,23 | 1,21E-04 |
| NUP54 | NM_017426 | -0,64 | 1,21E-04 |
| SKP2 | NM_005983 | -0,88 | 1,23E-04 |
| DHODH | NM_001361 | -1,22 | 1,23E-04 |
| NTAN1 | NM_173474 | -0,65 | 1,23E-04 |
| DFNB59 | NM_001042702 | 2,42 | 1,24E-04 |
| AIRE | NM_000383 | 2,83 | 1,24E-04 |
| ZNF615 | NM_198480 | 1,03 | 1,25E-04 |
| BRUNOL6 | NM_052840 | 2,46 | 1,25E-04 |
| PSMB5 | NM_001130725 | -0,62 | 1,26E-04 |
| HSPB2 | NM_001541 | -0,99 | 1,26E-04 |
| GIPC3 | NM_133261 | 1,10 | 1,27E-04 |
| MRPS28 | NM_014018 | -1,04 | 1,27E-04 |
| BEST1 | NM_004183 | 1,69 | 1,27E-04 |
| GAN | NM_022041 | -1,17 | 1,28E-04 |
| OPN1SW | NM_001708 | -2,03 | 1,28E-04 |
| LYRM5 | NM_001001660 | 1,03 | 1,29E-04 |
| OXNAD1 | NM_138381 | -1,09 | 1,29E-04 |
| TTN | NM_133378 | 0,85 | 1,29E-04 |
| IQCD | NM_138451 | 1,74 | 1,30E-04 |
| BIRC3 | NM_001165 | -2,12 | 1,30E-04 |
| PACS1 | NM_018026 | 0,74 | 1,30E-04 |
| PLS1 | NM_001145319 | -1,34 | 1,31E-04 |
| PLGLB2 | NM_002665 | 2,11 | 1,31E-04 |
| PLGLB1 | NM_001032392 | 2,11 | 1,31E-04 |
| ZDHHC6 | NM_022494 | -0,63 | 1,32E-04 |
| C5orf56 | NM_001013717 | 2,95 | 1,33E-04 |
| FBXW7 | NM_018315 | 0,84 | 1,35E-04 |
| CHPT1 | NM_020244 | -0,76 | 1,36E-04 |
| TMEM180 | NM_024789 | 1,47 | 1,36E-04 |
| EMID2 | NM_133457 | 1,73 | 1,37E-04 |
| KIAA1244 | NM_020340 | -1,38 | 1,37E-04 |
| TMEM38A | NM_024074 | 2,07 | 1,38E-04 |
| DUS3L | NM_020175 | -0,92 | 1,38E-04 |
| SEMA6D | NM_024966 | -0,98 | 1,38E-04 |
| PISD | NM_014338 | -0,63 | 1,40E-04 |
| MRPL50 | NM_019051 | -0,89 | 1,41E-04 |
| AMIGO1 | NM_020703 | 2,03 | 1,41E-04 |
| B3GALT1 | NM_020981 | 2,68 | 1,42E-04 |
| SYNE2 | NM_015180 | -0,87 | 1,42E-04 |
| LOC653566 | NR_027268 | -0,72 | 1,42E-04 |
| LOC387647 | NR_003930 | -0,61 | 1,42E-04 |
| ZNF610 | NM_001161427 | 2,08 | 1,42E-04 |
| NARS2 | NM_024678 | -0,86 | 1,43E-04 |
| GAMT | NM_000156 | 0,69 | 1,43E-04 |
| E2F8 | NM_024680 | -1,53 | 1,43E-04 |
| ANO1 | NR_030691 | -2,69 | 1,44E-04 |
| EXTL1 | NM_004455 | 2,14 | 1,44E-04 |
| CAMKK2 | NM_172214 | -0,68 | 1,44E-04 |
| TMEM106C | NM_001143842 | -0,69 | 1,45E-04 |
| MT1E | NM_175617 | -1,01 | 1,47E-04 |
| ABCC4 | NM_005845 | -0,71 | 1,48E-04 |
| HTR1D | NM_000864 | -2,25 | 1,49E-04 |
| EGLN2 | NM_080732 | -0,64 | 1,49E-04 |
| TDRD3 | NM_030794 | 0,87 | 1,50E-04 |
| TTC4 | NM_004623 | -0,84 | 1,51E-04 |
| SGMS1 | NM_147156 | -0,65 | 1,52E-04 |
| C6orf182 | NM_173830 | -0,85 | 1,52E-04 |
| NCRNA00105 | NR_026710 | 0,96 | 1,54E-04 |
| TXNDC11 | NM_015914 | -0,69 | 1,55E-04 |
| CCDC123 | NM_032816 | -0,72 | 1,57E-04 |
| KIAA1683 | NM_001145304 | 2,27 | 1,58E-04 |
| ZNF91 | NM_003430 | 0,87 | 1,58E-04 |
| FAHD1 | NM_031208 | -0,62 | 1,58E-04 |
| PCID2 | NM_001127202 | -0,65 | 1,59E-04 |
| C19orf36 | NM_001031735 | 2,41 | 1,59E-04 |
| NOL7 | NM_016167 | -0,78 | 1,60E-04 |
| COL28A1 | NM_001037763 | 3,22 | 1,60E-04 |
| RNF215 | NM_001017981 | 0,96 | 1,61E-04 |
| GIMAP7 | NM_153236 | -2,78 | 1,61E-04 |
| ALG12 | NM_024105 | 0,63 | 1,62E-04 |
| C16orf86 | NM_001012984 | 2,32 | 1,62E-04 |
| PPPDE2 | NM_015704 | -0,85 | 1,62E-04 |
| CBX2 | NM_005189 | -0,90 | 1,63E-04 |
| KLF11 | NM_003597 | 0,76 | 1,63E-04 |
| CES8 | NM_173815 | 2,19 | 1,63E-04 |
| CEL | NM_001807 | 1,68 | 1,63E-04 |
| ACOT13 | NM_018473 | 0,69 | 1,67E-04 |
| NFAT5 | NM_138713 | 0,88 | 1,68E-04 |
| PI4KAP1 | NR_003563 | 0,82 | 1,69E-04 |
| SERPINB8 | NM_002640 | -0,78 | 1,70E-04 |
| MTHFSD | NM_001159379 | 0,74 | 1,71E-04 |
| TNFRSF21 | NM_014452 | 0,81 | 1,71E-04 |
| NDE1 | NM_017668 | -0,61 | 1,71E-04 |
| NR1D1 | NM_021724 | 0,74 | 1,71E-04 |
| PXMP3 | NM_001079867 | 0,70 | 1,71E-04 |
| DBR1 | NM_016216 | -0,89 | 1,72E-04 |
| VASN | NM_138440 | -0,62 | 1,73E-04 |
| THOC7 | NM_025075 | -0,71 | 1,74E-04 |
| DCST2 | NM_144622 | 3,04 | 1,75E-04 |
| TMEM93 | NM_001014764 | -0,76 | 1,76E-04 |
| PPP2R5B | NM_006244 | -0,61 | 1,76E-04 |
| P4HA3 | NM_182904 | 0,95 | 1,76E-04 |
| NUDCD2 | NM_145266 | -0,85 | 1,77E-04 |
| KBTBD3 | NM_198439 | 2,04 | 1,80E-04 |
| AIM1 | NM_001624 | 1,11 | 1,80E-04 |
| DPYD | NM_000110 | 1,13 | 1,80E-04 |
| C17orf79 | NM_018405 | -0,84 | 1,81E-04 |
| ZNF473 | NM_015428 | -0,83 | 1,81E-04 |
| SRP19 | NM_003135 | -0,73 | 1,81E-04 |
| CENPT | NM_025082 | -0,70 | 1,82E-04 |
| CDK7 | NM_001799 | -0,86 | 1,83E-04 |
| PARP11 | NM_020367 | 0,98 | 1,83E-04 |
| SIRT2 | NM_030593 | 0,64 | 1,83E-04 |
| ZDBF2 | NM_020923 | 0,74 | 1,84E-04 |
| CTNNBIP1 | NM_001012329 | 0,77 | 1,85E-04 |
| NIT1 | NM_005600 | 0,73 | 1,86E-04 |
| PPOX | NM_001122764 | 0,99 | 1,86E-04 |
| ZNF227 | NM_182490 | 1,04 | 1,87E-04 |
| SUMF1 | NM_182760 | 0,60 | 1,87E-04 |
| TSPAN6 | NM_003270 | 0,81 | 1,90E-04 |
| MBOAT2 | NM_138799 | 0,67 | 1,90E-04 |
| ATP11A | NM_015205 | -0,82 | 1,90E-04 |
| SPTBN4 | NM_020971 | 2,77 | 1,90E-04 |
| PPP4R2 | NM_174907 | -0,71 | 1,91E-04 |
| RPL26L1 | NM_016093 | -0,86 | 1,91E-04 |
| STK33 | NM_030906 | 1,44 | 1,92E-04 |
| PEX11G | NM_080662 | 2,55 | 1,92E-04 |
| KLRC2 | NM_002260 | 3,18 | 1,93E-04 |
| RING1 | NM_002931 | 0,68 | 1,93E-04 |
| C19orf28 | NM_174983 | -0,63 | 1,93E-04 |
| ZNF205 | NM_001042428 | 0,71 | 1,93E-04 |
| UBXN1 | NM_015853 | 0,85 | 1,94E-04 |
| MAP9 | NM_001039580 | 0,81 | 1,96E-04 |
| TAF5 | NM_006951 | -0,89 | 1,96E-04 |
| RPUSD2 | NM_152260 | -0,95 | 1,99E-04 |
| HECW2 | NM_020760 | -0,82 | 1,99E-04 |
| TBCEL | NM_001130047 | 0,61 | 1,99E-04 |
| RGS16 | NM_002928 | 2,14 | 2,00E-04 |
| PCLO | NM_014510 | 1,84 | 2,02E-04 |
| ARRDC2 | NM_015683 | 0,78 | 2,03E-04 |
| COL11A2 | NM_080680 | 1,53 | 2,03E-04 |
| HPX | NM_000613 | 2,05 | 2,05E-04 |
| PGM5P2 | NR_002836 | -2,06 | 2,06E-04 |
| EEPD1 | NM_030636 | 1,42 | 2,08E-04 |
| RAB24 | NM_001031677 | 0,81 | 2,09E-04 |
| ZNF540 | NM_152606 | 2,57 | 2,09E-04 |
| AP4E1 | NM_007347 | -0,67 | 2,09E-04 |
| C2orf63 | NM_152385 | 1,58 | 2,10E-04 |
| TOM1 | NM_001135730 | 0,69 | 2,14E-04 |
| SPEF2 | NM_024867 | 2,03 | 2,16E-04 |
| FAM110A | NM_001042353 | -1,12 | 2,19E-04 |
| NDRG2 | NM_201541 | 2,47 | 2,23E-04 |
| PELI3 | NM_001098510 | 0,84 | 2,25E-04 |
| TSNARE1 | NM_145003 | 2,04 | 2,26E-04 |
| ZNF738 | NR_027130 | -1,01 | 2,27E-04 |
| LOC100130987 | NR_024469 | 2,76 | 2,27E-04 |
| CTNNBL1 | NM_030877 | -0,79 | 2,27E-04 |
| SPIN4 | NM_001012968 | -0,79 | 2,31E-04 |
| WDR63 | NM_145172 | 2,05 | 2,32E-04 |
| IGFN1 | NM_001164586 | 1,13 | 2,33E-04 |
| TMEM179B | NM_199337 | 0,63 | 2,33E-04 |
| PTPRCAP | NM_005608 | 2,26 | 2,33E-04 |
| REV3L | NM_002912 | 0,66 | 2,35E-04 |
| MAPT | NM_001123066 | 1,58 | 2,35E-04 |
| SDSL | NM_138432 | 1,49 | 2,36E-04 |
| QPCTL | NM_017659 | -0,86 | 2,40E-04 |
| HAUS3 | NM_024511 | -0,69 | 2,41E-04 |
| TMEM135 | NM_022918 | 0,83 | 2,41E-04 |
| TAZ | NM_181312 | 0,67 | 2,42E-04 |
| KIAA0146 | NM_001080394 | 0,69 | 2,45E-04 |
| POM121C | NM_001099415 | -0,68 | 2,46E-04 |
| CECR5 | NM_017829 | -0,70 | 2,47E-04 |
| GABBR2 | NM_005458 | -2,42 | 2,48E-04 |
| TRIM38 | NM_006355 | 0,72 | 2,50E-04 |
| TXN | NM_003329 | -0,65 | 2,50E-04 |
| PARP8 | NM_024615 | 0,94 | 2,52E-04 |
| BLZF1 | NM_003666 | -0,80 | 2,54E-04 |
| C10orf118 | NM_018017 | 0,61 | 2,55E-04 |
| BMP2 | NM_001200 | -1,26 | 2,56E-04 |
| PLEKHG5 | NM_198681 | 0,76 | 2,58E-04 |
| DCPS | NM_014026 | -0,70 | 2,60E-04 |
| NHSL2 | NM_001013627 | 1,14 | 2,60E-04 |
| DRG1 | NM_004147 | -0,62 | 2,60E-04 |
| ARHGAP19 | NM_032900 | -0,63 | 2,61E-04 |
| ARL10 | NM_173664 | -0,71 | 2,62E-04 |
| NUTF2 | NM_005796 | -0,73 | 2,63E-04 |
| PPIC | NM_000943 | 0,73 | 2,64E-04 |
| POGZ | NM_015100 | 0,64 | 2,64E-04 |
| C10orf110 | NR_024628 | -1,76 | 2,65E-04 |
| RPP40 | NM_006638 | -1,20 | 2,65E-04 |
| TMEM22 | NM_025246 | -1,00 | 2,65E-04 |
| IFT52 | NM_016004 | 0,66 | 2,66E-04 |
| H2AFJ | NR_027716 | 0,81 | 2,67E-04 |
| ANKMY2 | NM_020319 | 0,63 | 2,67E-04 |
| LOC100289341 | NR_027447 | 1,33 | 2,68E-04 |
| C19orf20 | NM_033513 | 1,06 | 2,74E-04 |
| ULK2 | NM_014683 | 0,79 | 2,75E-04 |
| MRPS7 | NM_015971 | -0,76 | 2,76E-04 |
| DNAJB2 | NM_006736 | 0,76 | 2,76E-04 |
| MLYCD | NM_012213 | 1,03 | 2,78E-04 |
| LOC200030 | NM_183372 | 0,68 | 2,79E-04 |
| TXNRD2 | NM_006440 | -0,88 | 2,80E-04 |
| C21orf33 | NM_004649 | 0,70 | 2,83E-04 |
| PARP3 | NM_001003931 | 0,82 | 2,84E-04 |
| ZC3HC1 | NM_016478 | -0,69 | 2,84E-04 |
| RPS27L | NM_015920 | 0,66 | 2,84E-04 |
| RABGAP1L | NM_014857 | 0,76 | 2,85E-04 |
| ANXA4 | NM_001153 | 0,67 | 2,85E-04 |
| MFSD3 | NM_138431 | 0,98 | 2,86E-04 |
| SLC39A3 | NM_213568 | -0,83 | 2,86E-04 |
| BCAP31 | NR_024450 | -0,66 | 2,86E-04 |
| VSNL1 | NM_003385 | 2,39 | 2,87E-04 |
| TRIM11 | NM_145214 | -0,79 | 2,87E-04 |
| PEX26 | NM_017929 | -0,62 | 2,87E-04 |
| ZNF518A | NM_014803 | 0,70 | 2,90E-04 |
| GSTM1 | NM_000561 | 0,81 | 2,92E-04 |
| FAM65C | NM_080829 | 2,84 | 2,92E-04 |
| RAB20 | NM_017817 | -1,11 | 2,92E-04 |
| SHROOM4 | NM_020717 | -1,31 | 2,99E-04 |
| TCF7 | NM_003202 | 1,06 | 3,00E-04 |
| FRY | NM_023037 | -0,93 | 3,00E-04 |
| ALDH8A1 | NM_170771 | 3,10 | 3,00E-04 |
| ZNF177 | NM_003451 | 1,91 | 3,01E-04 |
| OPA3 | NM_001017989 | -1,24 | 3,05E-04 |
| PAX8 | NM_003466 | 1,96 | 3,08E-04 |
| C10orf11 | NM_032024 | 2,72 | 3,10E-04 |
| PSD | NM_002779 | 2,53 | 3,10E-04 |
| COL4A6 | NM_033641 | 1,07 | 3,10E-04 |
| KIF4B | NM_001099293 | -2,25 | 3,11E-04 |
| UNC93B1 | NM_030930 | 0,63 | 3,13E-04 |
| SYTL1 | NM_032872 | 2,35 | 3,14E-04 |
| SLAIN2 | NM_020846 | -0,62 | 3,15E-04 |
| CRYZL1 | NM_145858 | 0,86 | 3,19E-04 |
| PGBD1 | NM_032507 | -0,87 | 3,19E-04 |
| GEMIN7 | NM_001007269 | -0,79 | 3,20E-04 |
| XRN1 | NM_019001 | 0,69 | 3,20E-04 |
| MOAP1 | NM_022151 | 0,78 | 3,21E-04 |
| TRIM6 | NM_001003818 | -1,11 | 3,22E-04 |
| CCDC34 | NM_030771 | -1,01 | 3,23E-04 |
| BTBD16 | NM_144587 | 2,93 | 3,28E-04 |
| RBM15 | NM_022768 | -0,83 | 3,29E-04 |
| KIAA1529 | NM_020893 | 1,21 | 3,29E-04 |
| MRGPRF | NM_145015 | 0,63 | 3,30E-04 |
| UROS | NM_000375 | 0,68 | 3,32E-04 |
| UBA5 | NM_198329 | -0,72 | 3,33E-04 |
| EPHB3 | NM_004443 | 1,79 | 3,34E-04 |
| SEC14L5 | NM_014692 | 2,95 | 3,35E-04 |
| CDO1 | NM_001801 | 1,38 | 3,36E-04 |
| GALNTL4 | NM_198516 | -1,35 | 3,36E-04 |
| TRIM24 | NM_015905 | -0,64 | 3,37E-04 |
| ZMIZ1 | NM_020338 | 0,64 | 3,37E-04 |
| ERN1 | NM_001433 | 1,29 | 3,40E-04 |
| RAB26 | NM_014353 | 2,17 | 3,40E-04 |
| DAG1 | NM_004393 | -0,66 | 3,42E-04 |
| VARS2 | NM_020442 | 0,60 | 3,42E-04 |
| CLEC18B | NM_001011880 | 3,07 | 3,43E-04 |
| RND2 | NM_005440 | 2,03 | 3,44E-04 |
| LGI1 | NM_005097 | 3,08 | 3,45E-04 |
| ADRA2C | NM_000683 | 1,42 | 3,46E-04 |
| POLR3B | NM_001160708 | -0,73 | 3,47E-04 |
| WASH3P | NR_003659 | 0,69 | 3,48E-04 |
| DUSP22 | NM_020185 | 0,88 | 3,49E-04 |
| ZNF488 | NM_153034 | -1,07 | 3,50E-04 |
| USP27X | NM_001145073 | 1,10 | 3,50E-04 |
| SFRS15 | NM_001145444 | -0,65 | 3,52E-04 |
| FAM122C | NM_138819 | 2,33 | 3,52E-04 |
| BVES | NM_147147 | -1,03 | 3,53E-04 |
| CRISPLD2 | NM_031476 | -0,74 | 3,55E-04 |
| WDR44 | NM_019045 | -0,62 | 3,55E-04 |
| WDR59 | NM_030581 | 0,69 | 3,56E-04 |
| SNRPD2 | NM_004597 | -0,68 | 3,56E-04 |
| ZNF771 | NM_016643 | 0,98 | 3,57E-04 |
| NDUFB5 | NM_002492 | 0,61 | 3,58E-04 |
| THBD | NM_000361 | 0,61 | 3,60E-04 |
| TMEM53 | NM_024587 | 1,39 | 3,62E-04 |
| ZNF510 | NM_014930 | 0,80 | 3,63E-04 |
| CST3 | NM_000099 | 0,75 | 3,65E-04 |
| ZNF285A | NM_152354 | 1,51 | 3,67E-04 |
| RGNEF | NM_001080479 | -0,71 | 3,67E-04 |
| KRTCAP3 | NM_173853 | 2,61 | 3,69E-04 |
| A4GALT | NM_017436 | -0,61 | 3,71E-04 |
| JAKMIP3 | NM_001105521 | 2,49 | 3,72E-04 |
| ATXN1 | NM_001128164 | 0,62 | 3,72E-04 |
| RASL11A | NM_206827 | 1,52 | 3,72E-04 |
| IPP | NM_005897 | 0,91 | 3,74E-04 |
| DYNLT1 | NM_006519 | -0,64 | 3,75E-04 |
| NUDT5 | NM_014142 | -0,71 | 3,76E-04 |
| C6orf115 | NM_021243 | -1,05 | 3,76E-04 |
| PDLIM3 | NM_001114107 | -1,68 | 3,80E-04 |
| APCDD1L | NM_153360 | 0,79 | 3,80E-04 |
| ZSCAN2 | NM_181877 | 0,73 | 3,81E-04 |
| CASD1 | NM_022900 | 0,68 | 3,83E-04 |
| SCO1 | NM_004589 | -0,70 | 3,84E-04 |
| LOC440354 | NR_002473 | -1,06 | 3,85E-04 |
| ABAT | NM_020686 | 1,85 | 3,87E-04 |
| MYO1D | NM_015194 | 0,72 | 3,88E-04 |
| LOC100287227 | NR_027954 | 1,68 | 3,88E-04 |
| ZNF486 | NM_052852 | -1,67 | 3,90E-04 |
| ICA1L | NM_138468 | 1,22 | 3,90E-04 |
| DDN | NM_015086 | -1,54 | 3,92E-04 |
| OSBPL6 | NM_032523 | -1,04 | 3,92E-04 |
| WASF3 | NM_006646 | 0,64 | 3,95E-04 |
| ARHGAP20 | NM_020809 | 2,21 | 3,96E-04 |
| HNRNPA0 | NM_006805 | -0,61 | 3,97E-04 |
| KIAA0415 | NM_014855 | 0,62 | 3,98E-04 |
| AGL | NM_000644 | 0,74 | 4,00E-04 |
| FMO4 | NM_002022 | 2,92 | 4,03E-04 |
| INPP5B | NM_005540 | 0,71 | 4,05E-04 |
| TMEM116 | NM_138341 | 1,30 | 4,06E-04 |
| TELO2 | NM_016111 | -0,72 | 4,06E-04 |
| ENGASE | NM_001042573 | 0,90 | 4,08E-04 |
| TMEM70 | NM_001040613 | -0,79 | 4,09E-04 |
| STK11IP | NM_052902 | 0,74 | 4,09E-04 |
| MAGOH | NM_002370 | -0,98 | 4,09E-04 |
| C1orf122 | NM_001142726 | 0,87 | 4,14E-04 |
| CP110 | NM_014711 | -0,63 | 4,18E-04 |
| LSM1 | NM_014462 | -0,69 | 4,18E-04 |
| MCTP2 | NM_018349 | 1,28 | 4,19E-04 |
| ARMC9 | NM_025139 | 0,80 | 4,20E-04 |
| ZNF605 | NM_001164715 | 0,70 | 4,21E-04 |
| PORCN | NM_203476 | 0,88 | 4,26E-04 |
| PYGL | NM_002863 | -0,65 | 4,26E-04 |
| RELN | NM_005045 | -0,76 | 4,26E-04 |
| CLOCK | NM_004898 | -0,82 | 4,31E-04 |
| DUSP12 | NM_007240 | -0,82 | 4,35E-04 |
| ACACB | NM_001093 | 1,23 | 4,36E-04 |
| STX11 | NM_003764 | -1,79 | 4,37E-04 |
| KRT19 | NM_002276 | -1,19 | 4,37E-04 |
| LDHB | NM_002300 | -0,67 | 4,37E-04 |
| XPO4 | NM_022459 | -0,62 | 4,38E-04 |
| MASP2 | NM_006610 | 1,76 | 4,39E-04 |
| C17orf62 | NM_001100408 | 0,81 | 4,41E-04 |
| OR51E2 | NM_030774 | -0,82 | 4,43E-04 |
| PACSIN1 | NM_020804 | 2,38 | 4,46E-04 |
| B4GALT3 | NM_003779 | -0,63 | 4,46E-04 |
| PVRIG | NM_024070 | 1,06 | 4,46E-04 |
| CSRP2BP | NR_028402 | 0,69 | 4,46E-04 |
| ORMDL2 | NM_014182 | -0,79 | 4,54E-04 |
| RGS11 | NM_003834 | 1,79 | 4,63E-04 |
| SLC29A3 | NM_018344 | 1,30 | 4,64E-04 |
| IGSF9B | NM_014987 | 2,31 | 4,66E-04 |
| LRRN3 | NM_001099660 | -1,60 | 4,68E-04 |
| GNB5 | NM_006578 | 0,66 | 4,69E-04 |
| FAM96B | NM_016062 | -0,67 | 4,71E-04 |
| BAIAP2 | NM_001144888 | 0,63 | 4,71E-04 |
| METRNL | NM_001004431 | -0,69 | 4,72E-04 |
| WARS2 | NM_015836 | 0,76 | 4,72E-04 |
| C4orf49 | NM_032623 | 1,59 | 4,76E-04 |
| DOK4 | NM_018110 | 0,66 | 4,76E-04 |
| HAUS5 | NM_015302 | -0,68 | 4,77E-04 |
| RPP14 | NM_007042 | -0,63 | 4,78E-04 |
| LTB4R2 | NM_019839 | 0,91 | 4,80E-04 |
| CCDC74B | NM_207310 | 1,58 | 4,81E-04 |
| DERA | NM_015954 | -0,76 | 4,85E-04 |
| WDR33 | NM_001006622 | -0,91 | 4,85E-04 |
| RUNX3 | NM_001031680 | -1,56 | 4,85E-04 |
| PRPSAP2 | NM_002767 | -0,71 | 4,90E-04 |
| EFCAB7 | NM_032437 | 1,21 | 4,90E-04 |
| TIRAP | NM_001039661 | 0,90 | 4,91E-04 |
| CEP170L | NR_003135 | -1,08 | 4,91E-04 |
| NFIL3 | NM_005384 | -0,64 | 4,95E-04 |
| PTS | NM_000317 | -1,00 | 4,97E-04 |
| TMEM187 | NM_003492 | 1,38 | 4,98E-04 |
| ZNF337 | NM_015655 | 0,62 | 5,00E-04 |
| C16orf91 | NM_001010878 | -1,42 | 5,00E-04 |
| PXK | NM_017771 | 0,76 | 5,03E-04 |
| MRPL32 | NM_031903 | -0,63 | 5,08E-04 |
| LRRC66 | NM_001024611 | 2,38 | 5,09E-04 |
| CRISPLD1 | NM_031461 | 1,59 | 5,13E-04 |
| GPR153 | NM_207370 | 1,42 | 5,15E-04 |
| TMEM182 | NM_144632 | 1,62 | 5,15E-04 |
| BPI | NM_001725 | 2,87 | 5,16E-04 |
| FKBP11 | NM_001143782 | -0,71 | 5,17E-04 |
| C17orf57 | NM_152347 | 1,89 | 5,21E-04 |
| MIR214 | NR_029627 | 1,48 | 5,24E-04 |
| ZNF236 | NM_007345 | 0,69 | 5,24E-04 |
| C21orf2 | NM_004928 | 0,67 | 5,24E-04 |
| SHPK | NM_013276 | -0,66 | 5,26E-04 |
| PGLS | NM_012088 | 0,70 | 5,26E-04 |
| SRD5A3 | NM_024592 | -0,74 | 5,29E-04 |
| SELK | NM_021237 | -0,93 | 5,30E-04 |
| KRT81 | NM_002281 | -2,85 | 5,34E-04 |
| ZNF322B | NM_199005 | 0,73 | 5,35E-04 |
| ARF5 | NM_001662 | 0,75 | 5,36E-04 |
| C21orf91 | NM_001100420 | 0,68 | 5,38E-04 |
| ALS2 | NM_001135745 | -0,81 | 5,38E-04 |
| MAMDC4 | NM_206920 | 0,81 | 5,41E-04 |
| BRE | NM_199191 | 0,77 | 5,44E-04 |
| FAM98C | NM_174905 | 1,11 | 5,44E-04 |
| TP53RK | NM_033550 | -0,66 | 5,53E-04 |
| MSC | NM_005098 | 0,97 | 5,55E-04 |
| SLC15A3 | NM_016582 | 1,35 | 5,56E-04 |
| FAM179A | NM_199280 | 2,49 | 5,57E-04 |
| AKR1B15 | NM_001080538 | 2,72 | 5,58E-04 |
| RAD51L3 | NM_002878 | -0,81 | 5,59E-04 |
| BET1 | NM_005868 | 0,80 | 5,60E-04 |
| CCR10 | NM_016602 | 1,75 | 5,65E-04 |
| FABP5 | NM_001444 | -1,70 | 5,68E-04 |
| MTBP | NM_022045 | -1,14 | 5,71E-04 |
| PELO | NM_015946 | -0,63 | 5,72E-04 |
| CHD1 | NM_001270 | -0,67 | 5,73E-04 |
| CCNJL | NM_024565 | -0,89 | 5,77E-04 |
| CCDC90A | NM_001031713 | -0,98 | 5,82E-04 |
| IRF7 | NM_004029 | 0,94 | 5,84E-04 |
| FMN1 | NM_001103184 | -2,12 | 5,86E-04 |
| SERGEF | NM_012139 | 1,07 | 5,87E-04 |
| ZNF404 | NM_001033719 | 1,30 | 5,90E-04 |
| GABRB1 | NM_000812 | -1,47 | 5,95E-04 |
| LOC100271836 | NR_027155 | -0,94 | 6,00E-04 |
| TG | NM_003235 | 2,51 | 6,06E-04 |
| DGKI | NM_004717 | 0,81 | 6,10E-04 |
| OR2A9P | NR_002157 | 1,34 | 6,11E-04 |
| ZNF414 | NM_001146175 | 0,72 | 6,11E-04 |
| CREBZF | NR_028024 | 0,61 | 6,13E-04 |
| FABP4 | NM_001442 | -1,71 | 6,15E-04 |
| MYSM1 | NM_001085487 | 0,77 | 6,18E-04 |
| PM20D2 | NM_001010853 | -0,74 | 6,19E-04 |
| OSCAR | NM_206818 | 2,72 | 6,22E-04 |
| ZNF843 | NM_001136509 | 2,50 | 6,25E-04 |
| C17orf97 | NM_001013672 | 0,83 | 6,27E-04 |
| C12orf41 | NM_017822 | -0,65 | 6,33E-04 |
| LOC283314 | NR_026947 | 1,62 | 6,34E-04 |
| PRUNE | NM_021222 | 0,78 | 6,35E-04 |
| ZGLP1 | NM_001103167 | 1,63 | 6,44E-04 |
| LRRC48 | NM_001130090 | 2,03 | 6,44E-04 |
| MRPL20 | NM_017971 | -0,69 | 6,46E-04 |
| RELB | NM_006509 | 0,82 | 6,46E-04 |
| EMB | NM_198449 | -0,99 | 6,49E-04 |
| APOBEC3A | NM_145699 | -2,27 | 6,50E-04 |
| RSPH3 | NM_031924 | 1,06 | 6,52E-04 |
| DUSP19 | NM_080876 | 1,26 | 6,54E-04 |
| MYEOV | NM_138768 | -2,60 | 6,54E-04 |
| PHYHIPL | NM_001143774 | 2,60 | 6,54E-04 |
| POLR1D | NM_152705 | 0,71 | 6,56E-04 |
| HAGHL | NM_207112 | 1,25 | 6,57E-04 |
| MAST4 | NM_001164664 | 0,62 | 6,57E-04 |
| ANKRD39 | NM_016466 | -1,11 | 6,58E-04 |
| SH3BGR | NM_001001713 | 2,00 | 6,63E-04 |
| ELMOD3 | NM_001135021 | 0,76 | 6,64E-04 |
| CHST1 | NM_003654 | -1,50 | 6,68E-04 |
| PTPDC1 | NM_177995 | -0,85 | 6,68E-04 |
| TTC18 | NM_145170 | 1,92 | 6,68E-04 |
| RCAN3 | NM_013441 | -1,03 | 6,69E-04 |
| SOX8 | NM_014587 | 1,81 | 6,70E-04 |
| CLUAP1 | NM_024793 | 0,82 | 6,71E-04 |
| CCDC134 | NM_024821 | -1,33 | 6,74E-04 |
| DMAP1 | NM_001034023 | 0,71 | 6,75E-04 |
| MACROD1 | NM_014067 | 1,34 | 6,75E-04 |
| MIOS | NM_019005 | -0,65 | 6,79E-04 |
| SERHL | NR_027786 | -1,00 | 6,79E-04 |
| LOC729082 | NR_026757 | -0,60 | 6,80E-04 |
| FAM58A | NM_152274 | -0,73 | 6,80E-04 |
| FLRT2 | NM_013231 | 0,74 | 6,81E-04 |
| PSMB10 | NM_002801 | 0,85 | 6,84E-04 |
| PSMD9 | NM_002813 | -0,64 | 6,86E-04 |
| BCAN | NM_021948 | 2,94 | 6,89E-04 |
| KCTD19 | NM_001100915 | 2,94 | 6,91E-04 |
| NEDD4 | NM_006154 | -0,60 | 6,95E-04 |
| OSBPL10 | NM_017784 | -0,98 | 6,95E-04 |
| VWCE | NM_152718 | 1,12 | 6,98E-04 |
| ALDOC | NM_005165 | -0,81 | 7,00E-04 |
| SCMH1 | NM_012236 | 0,73 | 7,02E-04 |
| CNTLN | NM_017738 | 0,81 | 7,04E-04 |
| C14orf147 | NM_138288 | -0,66 | 7,05E-04 |
| RPSAP52 | NR_026825 | -2,10 | 7,07E-04 |
| PREX2 | NM_024870 | -2,94 | 7,07E-04 |
| MOCS1 | NM_138928 | -0,82 | 7,08E-04 |
| LOC729991-MEF2B | NR_027307 | 0,81 | 7,09E-04 |
| ANXA9 | NM_003568 | 1,69 | 7,11E-04 |
| FAM13A | NM_001015045 | 0,72 | 7,12E-04 |
| LYN | NM_002350 | -0,73 | 7,17E-04 |
| GFM2 | NM_170681 | -0,66 | 7,17E-04 |
| AKD1 | NM_145025 | 1,60 | 7,26E-04 |
| CNIH3 | NM_152495 | 2,56 | 7,26E-04 |
| TTC23 | NM_001040657 | 0,70 | 7,26E-04 |
| GRPEL2 | NM_152407 | -0,70 | 7,31E-04 |
| ZNF780A | NM_001142579 | 0,96 | 7,39E-04 |
| ZNF658 | NM_033160 | 1,30 | 7,40E-04 |
| ANKDD1A | NM_182703 | 1,53 | 7,40E-04 |
| FLI1 | NM_002017 | -0,77 | 7,42E-04 |
| FBXO10 | NM_012166 | 0,69 | 7,42E-04 |
| BCDIN3D | NM_181708 | 0,99 | 7,42E-04 |
| PDE1A | NM_005019 | 2,94 | 7,42E-04 |
| HPDL | NM_032756 | -1,77 | 7,43E-04 |
| FEZ1 | NM_005103 | 0,66 | 7,47E-04 |
| C19orf50 | NM_024069 | -0,61 | 7,49E-04 |
| C12orf49 | NM_024738 | -0,62 | 7,49E-04 |
| CXCL5 | NM_002994 | -2,79 | 7,50E-04 |
| ORAI1 | NM_032790 | -0,77 | 7,52E-04 |
| PI4KAP2 | NR_003700 | 0,62 | 7,55E-04 |
| AMPD3 | NM_001025389 | 0,92 | 7,56E-04 |
| PICK1 | NM_012407 | 0,78 | 7,57E-04 |
| TFB2M | NM_022366 | -0,78 | 7,62E-04 |
| HGSNAT | NM_152419 | 0,71 | 7,63E-04 |
| PLB1 | NM_153021 | 2,81 | 7,65E-04 |
| PHYH | NM_001037537 | 0,89 | 7,81E-04 |
| LGR6 | NM_021636 | 2,70 | 7,86E-04 |
| AKR7L | NM_201252 | 1,47 | 7,89E-04 |
| ADAM23 | NM_003812 | -0,93 | 7,92E-04 |
| C5orf39 | NM_001014279 | 1,77 | 8,05E-04 |
| RIOK2 | NM_018343 | -0,61 | 8,10E-04 |
| PTGER2 | NM_000956 | 0,85 | 8,10E-04 |
| C6orf81 | NM_145028 | 2,48 | 8,11E-04 |
| UBL4A | NM_014235 | -0,62 | 8,23E-04 |
| GIMAP5 | NM_018384 | -2,47 | 8,23E-04 |
| FCRLA | NM_032738 | -2,91 | 8,24E-04 |
| LOC220594 | NR_003554 | 1,18 | 8,25E-04 |
| TTC25 | NM_031421 | 2,06 | 8,26E-04 |
| RUNX2 | NM_001015051 | -0,81 | 8,28E-04 |
| PRKRIP1 | NM_024653 | 0,65 | 8,34E-04 |
| SYDE2 | NM_032184 | -1,87 | 8,37E-04 |
| FAIM | NM_018147 | -0,85 | 8,39E-04 |
| LOC154761 | NR_015421 | -1,50 | 8,43E-04 |
| RASGRP3 | NM_170672 | -1,89 | 8,44E-04 |
| NAF1 | NM_138386 | -0,77 | 8,48E-04 |
| WNT16 | NM_057168 | 1,65 | 8,51E-04 |
| CCDC45 | NM_138363 | 0,67 | 8,51E-04 |
| KRBA1 | NM_032534 | 0,75 | 8,52E-04 |
| EIF1B | NM_005875 | 0,62 | 8,55E-04 |
| TBL3 | NM_006453 | -0,77 | 8,63E-04 |
| C12orf57 | NM_138425 | 0,70 | 8,70E-04 |
| TMEM20 | NM_001134658 | -1,36 | 8,70E-04 |
| NRIP2 | NM_031474 | 1,61 | 8,71E-04 |
| C9orf93 | NM_173550 | 1,40 | 8,71E-04 |
| MRPL27 | NM_016504 | -0,67 | 8,72E-04 |
| ZIK1 | NM_001010879 | -0,91 | 8,72E-04 |
| ZNF821 | NM_017530 | 1,01 | 8,78E-04 |
| SLFN12 | NM_018042 | -0,87 | 8,79E-04 |
| TMEM55A | NM_018710 | 0,74 | 8,85E-04 |
| IFT20 | NM_174887 | 0,67 | 8,91E-04 |
| GOLGA6L5 | NR_003246 | 1,24 | 9,02E-04 |
| STK16 | NR_026909 | 0,64 | 9,05E-04 |
| H3F3C | NM_001013699 | -0,65 | 9,13E-04 |
| ILKAP | NM_030768 | -0,71 | 9,17E-04 |
| KLRC4 | NM_013431 | 2,89 | 9,18E-04 |
| GCLC | NM_001498 | 0,68 | 9,19E-04 |
| C10orf12 | NM_015652 | -0,81 | 9,22E-04 |
| SPHK1 | NM_182965 | -0,79 | 9,28E-04 |
| ZNF497 | NM_198458 | 0,94 | 9,31E-04 |
| KCP | NM_001135914 | 2,76 | 9,31E-04 |
| KLHDC4 | NM_017566 | -0,89 | 9,34E-04 |
| TUBGCP5 | NM_052903 | -0,65 | 9,38E-04 |
| MIR656 | NR_030392 | 2,56 | 9,38E-04 |
| DERL2 | NM_016041 | -0,64 | 9,39E-04 |
| AGAP11 | NM_133447 | 1,64 | 9,43E-04 |
| ST3GAL4 | NM_006278 | -0,75 | 9,44E-04 |
| ICT1 | NM_001545 | -0,76 | 9,44E-04 |
| FAM198B | NM_016613 | -0,75 | 9,46E-04 |
| EPSTI1 | NM_001002264 | 1,33 | 9,48E-04 |
| WASL | NM_003941 | 0,64 | 9,56E-04 |
| AADAC | NM_001086 | -1,99 | 9,62E-04 |
| ZNF267 | NM_003414 | -0,82 | 9,63E-04 |
| POM121L10P | NR_024593 | 0,96 | 9,63E-04 |
| C7orf51 | NM_173564 | 1,35 | 9,63E-04 |
| GFRA2 | NM_001495 | 1,56 | 9,64E-04 |
| MORN1 | NM_024848 | 1,68 | 9,64E-04 |
| ATP6AP1L | NM_001017971 | 1,86 | 9,65E-04 |
| NDUFS6 | NM_004553 | -0,66 | 9,70E-04 |
| KLHL3 | NM_017415 | 1,44 | 9,70E-04 |
| INSRR | NM_014215 | 2,87 | 9,75E-04 |
| FMO5 | NM_001144829 | 2,76 | 9,77E-04 |
| SLC25A34 | NM_207348 | 1,63 | 9,78E-04 |
| C2orf44 | NM_025203 | -0,68 | 9,81E-04 |
| ADH1B | NM_000668 | 2,87 | 9,83E-04 |
| FAM176B | NM_018166 | 0,66 | 9,83E-04 |
| NLGN3 | NM_018977 | 2,04 | 9,88E-04 |
| PIWIL4 | NM_152431 | 2,15 | 9,88E-04 |
| MDK | NM_001012334 | 0,62 | 1,00E-03 |
| MPP3 | NR_003562 | -1,05 | 1,01E-03 |
| MMAA | NM_172250 | 1,01 | 1,01E-03 |
| CPNE3 | NM_003909 | 0,61 | 1,01E-03 |
| ZNF189 | NM_197977 | 0,64 | 1,01E-03 |
| ZNF197 | NM_006991 | 0,67 | 1,02E-03 |
| NGEF | NM_001114090 | 1,24 | 1,02E-03 |
| TNFAIP8L3 | NM_207381 | -2,42 | 1,03E-03 |
| SOX17 | NM_022454 | -1,98 | 1,03E-03 |
| ANKRD44 | NM_153697 | 1,23 | 1,04E-03 |
| CLYBL | NM_206808 | 2,52 | 1,04E-03 |
| NIPA1 | NM_001142275 | -0,64 | 1,05E-03 |
| SURF1 | NM_003172 | 1,00 | 1,06E-03 |
| CCDC107 | NM_174923 | -0,91 | 1,06E-03 |
| STEAP1 | NM_012449 | -2,04 | 1,06E-03 |
| TDRD7 | NM_014290 | 0,82 | 1,07E-03 |
| GRHL3 | NM_198173 | 2,51 | 1,07E-03 |
| WRN | NM_000553 | -0,65 | 1,07E-03 |
| LOC399815 | NR_027282 | -1,90 | 1,08E-03 |
| C6orf170 | NM_152730 | 1,23 | 1,08E-03 |
| C14orf45 | NM_025057 | 1,10 | 1,08E-03 |
| AGBL2 | NM_024783 | 2,52 | 1,09E-03 |
| WTIP | NM_001080436 | -0,81 | 1,09E-03 |
| TMEM134 | NM_001078650 | 0,78 | 1,09E-03 |
| MYPOP | NM_001012643 | 0,72 | 1,09E-03 |
| CAPN12 | NM_144691 | -0,74 | 1,09E-03 |
| NXNL2 | NM_001161625 | 2,62 | 1,10E-03 |
| SERINC4 | NM_001033517 | 1,00 | 1,10E-03 |
| POP5 | NM_015918 | -0,72 | 1,10E-03 |
| GLT8D3 | NM_173601 | -0,61 | 1,10E-03 |
| ZNF202 | NM_003455 | -0,70 | 1,12E-03 |
| TMEM102 | NM_178518 | 1,06 | 1,12E-03 |
| NSUN5B | NM_001039575 | 0,61 | 1,12E-03 |
| MAPK8IP2 | NM_016431 | 1,44 | 1,13E-03 |
| NMNAT2 | NM_015039 | -0,75 | 1,13E-03 |
| LONRF1 | NM_152271 | 0,83 | 1,13E-03 |
| OSCP1 | NM_145047 | 1,28 | 1,14E-03 |
| SHBG | NM_001040 | 2,60 | 1,15E-03 |
| EXOC6 | NM_019053 | -0,90 | 1,15E-03 |
| TRIML2 | NM_173553 | -2,27 | 1,15E-03 |
| GALNTL1 | NM_020692 | 0,61 | 1,16E-03 |
| HERC6 | NM_017912 | 1,58 | 1,16E-03 |
| MAGIX | NM_001099680 | 2,04 | 1,17E-03 |
| LOC401093 | NR_027037 | -0,78 | 1,17E-03 |
| NMNAT1 | NM_022787 | 0,71 | 1,17E-03 |
| BSN | NM_003458 | 1,37 | 1,17E-03 |
| PUSL1 | NM_153339 | -0,69 | 1,18E-03 |
| C17orf100 | NM_001105520 | 1,30 | 1,18E-03 |
| LOC652276 | NR_015441 | -0,90 | 1,18E-03 |
| TIMM8B | NM_012459 | -0,68 | 1,18E-03 |
| LOC100129534 | NR_024489 | 1,87 | 1,19E-03 |
| GDF1 | NM_001492 | 1,15 | 1,19E-03 |
| LASS1 | NM_021267 | 1,15 | 1,19E-03 |
| TTF1 | NM_007344 | -0,73 | 1,20E-03 |
| C14orf37 | NM_001001872 | 1,00 | 1,20E-03 |
| ZNF607 | NM_032689 | 0,96 | 1,20E-03 |
| MRPL23 | NM_021134 | -0,72 | 1,20E-03 |
| WNT7B | NM_058238 | -1,81 | 1,21E-03 |
| TATDN3 | NM_001042552 | 1,05 | 1,21E-03 |
| NGFR | NM_002507 | 1,89 | 1,22E-03 |
| FBXO4 | NM_012176 | 1,16 | 1,22E-03 |
| NEURL1B | NM_001142651 | -1,07 | 1,22E-03 |
| RTTN | NM_173630 | -0,75 | 1,22E-03 |
| MOXD1 | NM_015529 | 1,24 | 1,22E-03 |
| GLCCI1 | NM_138426 | 0,80 | 1,22E-03 |
| THAP9 | NM_024672 | 1,08 | 1,23E-03 |
| GPLD1 | NM_001503 | 1,44 | 1,23E-03 |
| RCBTB1 | NM_018191 | 0,62 | 1,23E-03 |
| ZYG11A | NM_001004339 | -1,43 | 1,24E-03 |
| TTC30A | NM_152275 | 0,80 | 1,24E-03 |
| CCDC11 | NM_145020 | 2,31 | 1,25E-03 |
| CTAGE5 | NM_203355 | 0,70 | 1,25E-03 |
| TNXB | NM_019105 | 0,83 | 1,25E-03 |
| CCDC30 | NM_001080850 | 2,71 | 1,25E-03 |
| MRP63 | NM_024026 | -0,61 | 1,27E-03 |
| UCHL3 | NM_006002 | -0,79 | 1,27E-03 |
| SURF6 | NM_006753 | -0,67 | 1,28E-03 |
| TNFRSF25 | NM_148967 | 1,20 | 1,29E-03 |
| APTX | NM_175072 | -0,66 | 1,29E-03 |
| ELF1 | NM_172373 | 0,68 | 1,30E-03 |
| ZNF213 | NM_004220 | 0,63 | 1,30E-03 |
| LOC283663 | NR_024433 | 2,17 | 1,30E-03 |
| PNPLA3 | NM_025225 | -1,26 | 1,31E-03 |
| FRAS1 | NM_025074 | 2,07 | 1,31E-03 |
| FAM66C | NR_026788 | 2,40 | 1,31E-03 |
| SCN1B | NM_001037 | 1,07 | 1,32E-03 |
| NHLRC4 | NM_176677 | 2,48 | 1,32E-03 |
| ZNF585A | NM_199126 | 1,23 | 1,32E-03 |
| XRCC4 | NM_003401 | -0,93 | 1,33E-03 |
| ARRB2 | NM_199004 | 0,75 | 1,33E-03 |
| ZNF530 | NM_020880 | -1,24 | 1,33E-03 |
| ZNF571 | NM_016536 | 1,86 | 1,33E-03 |
| C2orf7 | NM_032319 | -0,69 | 1,33E-03 |
| GLDC | NM_000170 | 1,27 | 1,34E-03 |
| COLQ | NM_080538 | 1,81 | 1,34E-03 |
| GTPBP6 | NM_012227 | -0,70 | 1,34E-03 |
| CNOT6L | NM_144571 | 0,68 | 1,34E-03 |
| TFR2 | NM_003227 | -1,86 | 1,34E-03 |
| LOC147804 | NR_003148 | -1,21 | 1,35E-03 |
| LMBR1L | NM_018113 | 0,65 | 1,35E-03 |
| AMPH | NM_139316 | 0,62 | 1,35E-03 |
| MBOAT1 | NM_001080480 | -1,34 | 1,36E-03 |
| C9orf79 | NM_178828 | 2,80 | 1,36E-03 |
| HSPB6 | NM_144617 | 0,72 | 1,36E-03 |
| HOMER1 | NM_004272 | -0,70 | 1,37E-03 |
| CABYR | NM_012189 | -1,27 | 1,37E-03 |
| CDKN2AIPNL | NM_080656 | -0,72 | 1,38E-03 |
| GCSH | NM_004483 | -0,95 | 1,39E-03 |
| NSUN5C | NM_032158 | 0,66 | 1,39E-03 |
| A1BG | NM_130786 | 1,03 | 1,40E-03 |
| MIF4GD | NM_020679 | 0,73 | 1,40E-03 |
| LOC282997 | NR_026932 | 1,39 | 1,40E-03 |
| CHCHD7 | NM_001011670 | 0,84 | 1,40E-03 |
| LRRC20 | NM_207119 | -0,66 | 1,41E-03 |
| GALC | NM_000153 | 0,70 | 1,41E-03 |
| DOK3 | NM_001144875 | -1,37 | 1,41E-03 |
| WDR46 | NM_001164267 | -0,62 | 1,41E-03 |
| FAM151B | NM_205548 | 1,72 | 1,42E-03 |
| ZNF655 | NM_024061 | -0,64 | 1,42E-03 |
| KIAA1530 | NM_020894 | 0,64 | 1,42E-03 |
| NAIP | NM_022892 | 1,55 | 1,43E-03 |
| UCN | NM_003353 | 2,50 | 1,43E-03 |
| GARNL3 | NM_032293 | 1,66 | 1,44E-03 |
| ZMYND15 | NM_001136046 | 2,56 | 1,44E-03 |
| CKB | NM_001823 | -0,77 | 1,44E-03 |
| TRDMT1 | NM_004412 | -1,05 | 1,44E-03 |
| LEO1 | NM_138792 | -0,68 | 1,45E-03 |
| PALB2 | NM_024675 | -0,84 | 1,46E-03 |
| ITIH3 | NM_002217 | 1,66 | 1,47E-03 |
| LRCH2 | NM_020871 | 0,86 | 1,47E-03 |
| C9orf114 | NM_016390 | -0,64 | 1,48E-03 |
| LRIG2 | NM_014813 | 0,77 | 1,49E-03 |
| MIR198 | NR_029584 | -0,68 | 1,49E-03 |
| MAP7 | NM_003980 | 1,47 | 1,49E-03 |
| RHBDF2 | NM_001005498 | 0,79 | 1,50E-03 |
| ZCCHC17 | NM_016505 | -0,64 | 1,50E-03 |
| SLC38A3 | NM_006841 | 2,49 | 1,50E-03 |
| C15orf54 | NM_207445 | -2,78 | 1,53E-03 |
| RNF130 | NM_018434 | 0,63 | 1,54E-03 |
| RHOJ | NM_020663 | 0,62 | 1,54E-03 |
| RICH2 | NM_014859 | 0,80 | 1,54E-03 |
| GLA | NM_000169 | -0,75 | 1,54E-03 |
| PLP2 | NM_002668 | -0,64 | 1,54E-03 |
| RNLS | NM_001031709 | 1,28 | 1,55E-03 |
| LRMP | NM_006152 | 2,41 | 1,55E-03 |
| ZBTB9 | NM_152735 | -0,62 | 1,56E-03 |
| C16orf75 | NM_152308 | -1,08 | 1,57E-03 |
| PTGS2 | NM_000963 | -0,73 | 1,57E-03 |
| CTHRC1 | NM_138455 | 0,78 | 1,57E-03 |
| CCDC39 | NM_181426 | 1,50 | 1,57E-03 |
| MGC3771 | NR_024167 | 1,29 | 1,58E-03 |
| PCDHB14 | NM_018934 | 1,94 | 1,61E-03 |
| ADAT2 | NM_182503 | -0,91 | 1,63E-03 |
| FBXL13 | NM_145032 | 1,99 | 1,64E-03 |
| MTERFD1 | NM_015942 | -0,72 | 1,64E-03 |
| C8orf73 | NM_001100878 | 0,89 | 1,66E-03 |
| CYTSB | NM_001033555 | 0,69 | 1,66E-03 |
| TOX | NM_014729 | 0,80 | 1,66E-03 |
| AIFM2 | NM_032797 | -0,74 | 1,66E-03 |
| FBXL4 | NM_012160 | 0,67 | 1,67E-03 |
| FAM86D | NR_024241 | -0,64 | 1,67E-03 |
| C11orf51 | NM_014042 | -0,81 | 1,69E-03 |
| WDR5B | NM_019069 | 0,74 | 1,70E-03 |
| DRAP1 | NM_006442 | -0,64 | 1,72E-03 |
| C4orf12 | NR_015359 | 1,45 | 1,73E-03 |
| TRADD | NM_003789 | 0,87 | 1,74E-03 |
| FLT4 | NM_002020 | -1,65 | 1,74E-03 |
| PDDC1 | NM_182612 | 0,67 | 1,75E-03 |
| MTF2 | NM_001164391 | -0,64 | 1,75E-03 |
| PCSK1N | NM_013271 | 1,83 | 1,75E-03 |
| FASTKD3 | NM_024091 | -0,89 | 1,76E-03 |
| LOC286367 | NR_024011 | 2,05 | 1,76E-03 |
| PRKCDBP | NM_145040 | 0,70 | 1,82E-03 |
| KRBA2 | NM_213597 | 1,39 | 1,82E-03 |
| SLC34A3 | NM_080877 | 2,21 | 1,82E-03 |
| DUSP6 | NM_001946 | -0,64 | 1,83E-03 |
| HCG11 | NR_026790 | 0,85 | 1,83E-03 |
| BOLA2 | NM_001031827 | -1,08 | 1,84E-03 |
| BOLA2B | NM_001039182 | -1,08 | 1,84E-03 |
| SNCB | NM_003085 | 2,19 | 1,84E-03 |
| NTRK3 | NM_001007156 | 2,75 | 1,86E-03 |
| POM121L8P | NR_024583 | 2,26 | 1,86E-03 |
| SLC6A16 | NM_014037 | 1,78 | 1,86E-03 |
| SLC26A1 | NM_022042 | 1,19 | 1,88E-03 |
| RAD52 | NM_134424 | 0,80 | 1,89E-03 |
| ZDHHC2 | NM_016353 | -0,68 | 1,89E-03 |
| KIAA1875 | NR_024207 | 1,98 | 1,89E-03 |
| C17orf65 | NM_178542 | 0,76 | 1,90E-03 |
| AMACR | NM_014324 | 0,88 | 1,90E-03 |
| COG6 | NR_026745 | 0,63 | 1,91E-03 |
| USP12 | NM_182488 | -0,61 | 1,91E-03 |
| BRWD3 | NM_153252 | 0,64 | 1,91E-03 |
| NCRNA00181 | NR_015380 | 0,81 | 1,92E-03 |
| COMMD3 | NM_012071 | 0,69 | 1,94E-03 |
| ACTC1 | NM_005159 | -2,52 | 1,95E-03 |
| FNIP2 | NM_020840 | -0,61 | 1,95E-03 |
| HIPK3 | NM_005734 | 0,63 | 1,97E-03 |
| SLC25A45 | NM_182556 | 1,19 | 1,97E-03 |
| EFCAB6 | NM_022785 | 2,52 | 1,97E-03 |
| C12orf29 | NM_001009894 | -0,79 | 1,99E-03 |
| NUP62CL | NM_017681 | 2,51 | 1,99E-03 |
| SCIN | NM_033128 | 2,51 | 2,00E-03 |
| CLCA2 | NM_006536 | 2,72 | 2,00E-03 |
| SERF2 | NM_001018108 | 0,64 | 2,00E-03 |
| GEMIN6 | NM_024775 | -0,94 | 2,01E-03 |
| ZNF350 | NM_021632 | 0,93 | 2,02E-03 |
| ZNF234 | NM_001144824 | 0,83 | 2,02E-03 |
| C9orf21 | NM_153698 | -1,01 | 2,02E-03 |
| SLC5A10 | NM_001042450 | -1,52 | 2,02E-03 |
| IKZF4 | NM_022465 | 0,73 | 2,02E-03 |
| HIST1H1B | NM_005322 | -2,64 | 2,04E-03 |
| ZNF490 | NM_020714 | 0,75 | 2,04E-03 |
| GCAT | NM_014291 | -0,64 | 2,05E-03 |
| ZMYM6 | NM_007167 | 0,65 | 2,06E-03 |
| CXorf26 | NM_016500 | -0,68 | 2,06E-03 |
| LOC646762 | NR_024278 | 0,72 | 2,06E-03 |
| TBC1D19 | NM_018317 | 0,97 | 2,06E-03 |
| SEPP1 | NM_005410 | 1,06 | 2,07E-03 |
| C16orf55 | NM_153025 | -0,84 | 2,09E-03 |
| SRGAP3 | NM_014850 | 2,71 | 2,10E-03 |
| SPAG8 | NM_001039592 | 1,94 | 2,10E-03 |
| C1QL1 | NM_006688 | 1,32 | 2,10E-03 |
| TBX6 | NM_004608 | 2,28 | 2,11E-03 |
| EML6 | NM_001039753 | 2,05 | 2,11E-03 |
| ACD | NM_022914 | -0,63 | 2,11E-03 |
| FHIT | NM_002012 | 2,71 | 2,12E-03 |
| KIF27 | NM_017576 | 1,15 | 2,13E-03 |
| TRIM16L | NM_001037330 | 0,70 | 2,13E-03 |
| HHEX | NM_002729 | -0,99 | 2,14E-03 |
| IGF2 | NM_000612 | 0,81 | 2,15E-03 |
| PODNL1 | NM_024825 | 1,86 | 2,15E-03 |
| HSPA14 | NM_016299 | -0,73 | 2,16E-03 |
| C5orf36 | NM_001145678 | 1,54 | 2,18E-03 |
| MRPL1 | NM_020236 | -0,73 | 2,19E-03 |
| C2orf56 | NM_144736 | 0,71 | 2,19E-03 |
| MAB21L2 | NM_006439 | 2,51 | 2,21E-03 |
| C20orf177 | NM_022106 | 0,74 | 2,21E-03 |
| ZDHHC14 | NM_153746 | 0,80 | 2,22E-03 |
| E2F6 | NR_003095 | -0,67 | 2,22E-03 |
| ZC3H12C | NM_033390 | -0,64 | 2,23E-03 |
| ZNF462 | NM_021224 | 0,62 | 2,23E-03 |
| FAM108C1 | NM_021214 | -0,97 | 2,24E-03 |
| GPAM | NM_020918 | -0,63 | 2,25E-03 |
| ZFP37 | NM_003408 | 1,41 | 2,26E-03 |
| MKNK1 | NR_024176 | 0,63 | 2,28E-03 |
| ACAA1 | NM_001607 | 0,60 | 2,28E-03 |
| COCH | NM_004086 | -0,84 | 2,29E-03 |
| LOC100126784 | NR_015384 | -0,98 | 2,29E-03 |
| WASH2P | NR_024077 | 0,89 | 2,30E-03 |
| ZBTB12 | NM_181842 | 0,84 | 2,30E-03 |
| IPPK | NM_022755 | -0,63 | 2,31E-03 |
| ENTPD1 | NM_001164181 | -0,68 | 2,33E-03 |
| LOC149134 | NR_015422 | 2,61 | 2,34E-03 |
| FAM161B | NM_152445 | 0,95 | 2,34E-03 |
| NBR2 | NR_003108 | 1,47 | 2,35E-03 |
| COX6B2 | NM_144613 | 2,48 | 2,37E-03 |
| GPR17 | NM_001161416 | -1,79 | 2,37E-03 |
| ROGDI | NM_024589 | 0,66 | 2,38E-03 |
| NLRX1 | NM_170722 | 0,74 | 2,39E-03 |
| WDR31 | NM_001012361 | 1,56 | 2,39E-03 |
| COQ3 | NM_017421 | -1,17 | 2,42E-03 |
| RPPH1 | NR_002312 | 1,39 | 2,43E-03 |
| C14orf179 | NM_052873 | 0,90 | 2,43E-03 |
| C1orf159 | NM_017891 | -0,73 | 2,43E-03 |
| FLJ35776 | NR_024101 | 1,30 | 2,47E-03 |
| B4GALNT1 | NM_001478 | -1,03 | 2,47E-03 |
| LMNA | NM_005572 | -0,64 | 2,48E-03 |
| LOC728855 | NR_024510 | 0,86 | 2,48E-03 |
| TMEM216 | NM_016499 | 0,90 | 2,50E-03 |
| RNF39 | NM_025236 | 1,25 | 2,51E-03 |
| SPSB2 | NM_001146316 | 0,81 | 2,52E-03 |
| EFNA1 | NM_182685 | -1,47 | 2,52E-03 |
| LAMC2 | NM_005562 | -1,34 | 2,55E-03 |
| FAM109A | NM_144671 | 0,72 | 2,58E-03 |
| ASMTL | NM_004192 | 0,64 | 2,59E-03 |
| NBPF10 | NM_001039703 | 0,76 | 2,59E-03 |
| LRRTM3 | NM_178011 | 2,66 | 2,59E-03 |
| OAS2 | NM_002535 | -2,08 | 2,60E-03 |
| EVC2 | NM_147127 | 0,83 | 2,60E-03 |
| GLRX2 | NM_016066 | -0,89 | 2,61E-03 |
| BTN2A3 | NR_027795 | 1,17 | 2,62E-03 |
| IQCK | NM_153208 | 0,97 | 2,62E-03 |
| MND1 | NM_032117 | -1,86 | 2,62E-03 |
| TSSK6 | NM_032037 | 1,70 | 2,63E-03 |
| COPG2 | NM_012133 | 0,67 | 2,64E-03 |
| LYPD1 | NM_001077427 | -0,93 | 2,65E-03 |
| RBM34 | NR_027762 | -0,63 | 2,67E-03 |
| ACRC | NM_052957 | 1,42 | 2,68E-03 |
| RP9P | NR_003500 | -0,85 | 2,71E-03 |
| GATSL2 | NM_001145064 | 1,87 | 2,72E-03 |
| CCDC88B | NM_032251 | 1,81 | 2,73E-03 |
| ZNF366 | NM_152625 | -2,48 | 2,74E-03 |
| ARHGAP4 | NM_001164741 | 1,56 | 2,74E-03 |
| QDPR | NM_000320 | -0,69 | 2,75E-03 |
| PRKAR1B | NM_001164762 | 0,68 | 2,76E-03 |
| C1orf51 | NM_144697 | -0,95 | 2,76E-03 |
| INS-IGF2 | NR_003512 | 0,78 | 2,76E-03 |
| MID1 | NM_000381 | -0,75 | 2,78E-03 |
| ZNF121 | NM_001008727 | -0,77 | 2,78E-03 |
| DHFRL1 | NM_176815 | 0,74 | 2,79E-03 |
| UNC5C | NM_003728 | 2,28 | 2,79E-03 |
| PCGF6 | NM_001011663 | -0,79 | 2,79E-03 |
| RABL2B | NM_001130921 | 0,69 | 2,79E-03 |
| SYPL2 | NM_001040709 | 1,97 | 2,82E-03 |
| ZNF577 | NM_032679 | 1,13 | 2,84E-03 |
| FRZB | NM_001463 | 1,46 | 2,84E-03 |
| MARCH2 | NM_016496 | 0,62 | 2,84E-03 |
| FNDC1 | NM_032532 | 1,40 | 2,86E-03 |
| CACNA2D3 | NM_018398 | 2,54 | 2,86E-03 |
| THSD7A | NM_015204 | -2,19 | 2,87E-03 |
| MFSD11 | NM_024311 | 0,69 | 2,87E-03 |
| NOV | NM_002514 | -0,90 | 2,93E-03 |
| SLC25A26 | NM_001164796 | 0,84 | 2,94E-03 |
| LOC653653 | NR_027408 | -1,55 | 2,95E-03 |
| CA5BP | NR_026551 | -0,90 | 2,96E-03 |
| KLHL23 | NM_144711 | -0,85 | 2,96E-03 |
| SLC30A1 | NM_021194 | -0,62 | 2,97E-03 |
| FUZ | NM_025129 | 0,73 | 2,97E-03 |
| CLMN | NM_024734 | 1,03 | 2,99E-03 |
| OMA1 | NM_145243 | 0,81 | 3,00E-03 |
| GPR4 | NM_005282 | -2,15 | 3,00E-03 |
| KCNJ12 | NM_021012 | 1,02 | 3,00E-03 |
| ABCG1 | NM_207627 | -2,13 | 3,01E-03 |
| LOC729603 | NR_003288 | 1,28 | 3,02E-03 |
| RNF150 | NM_020724 | 1,27 | 3,02E-03 |
| PTPLAD2 | NM_001010915 | 1,31 | 3,05E-03 |
| ASPSCR1 | NM_024083 | 0,71 | 3,05E-03 |
| TCEA3 | NM_003196 | 1,78 | 3,09E-03 |
| SLC7A7 | NM_001126106 | 1,49 | 3,09E-03 |
| CHN1 | NM_001822 | -0,89 | 3,11E-03 |
| ZNF204 | NR_002722 | 1,75 | 3,12E-03 |
| ZNF223 | NM_013361 | 1,25 | 3,12E-03 |
| PECI | NR_028588 | -0,61 | 3,12E-03 |
| DNAH7 | NM_018897 | 2,53 | 3,12E-03 |
| LOC100130932 | NM_001146693 | -0,85 | 3,14E-03 |
| HSD17B2 | NM_002153 | -2,36 | 3,15E-03 |
| EIF1AY | NM_004681 | -0,68 | 3,17E-03 |
| CDH18 | NM_004934 | 2,15 | 3,18E-03 |
| NRG1 | NM_001159995 | -1,43 | 3,20E-03 |
| CNR1 | NM_001160260 | 2,15 | 3,21E-03 |
| USP3 | NM_006537 | 0,65 | 3,21E-03 |
| ARRDC1 | NM_152285 | 0,83 | 3,24E-03 |
| FLJ45244 | NR_015415 | 1,99 | 3,26E-03 |
| GIPC2 | NM_017655 | -2,41 | 3,26E-03 |
| LOC100130015 | NR_027336 | 0,97 | 3,26E-03 |
| DENND2C | NM_198459 | 1,66 | 3,26E-03 |
| GDPD1 | NM_001165994 | 2,30 | 3,27E-03 |
| RAET1L | NM_130900 | -2,01 | 3,27E-03 |
| ADAM8 | NM_001164490 | 1,68 | 3,27E-03 |
| ADCY1 | NM_021116 | -1,01 | 3,29E-03 |
| LSM8 | NM_016200 | -0,66 | 3,30E-03 |
| RNF135 | NM_032322 | 0,69 | 3,30E-03 |
| CD58 | NM_001779 | -1,18 | 3,31E-03 |
| CCDC77 | NM_001130148 | -0,68 | 3,33E-03 |
| C3orf31 | NM_138807 | -0,82 | 3,33E-03 |
| FAM162A | NM_014367 | -0,64 | 3,34E-03 |
| CHCHD1 | NM_203298 | -0,71 | 3,34E-03 |
| FLJ13197 | NR_026804 | 1,65 | 3,35E-03 |
| C7orf31 | NM_138811 | 1,39 | 3,36E-03 |
| KIAA1598 | NM_018330 | 0,84 | 3,38E-03 |
| NOVA2 | NM_002516 | -2,50 | 3,39E-03 |
| TANK | NM_004180 | 0,67 | 3,39E-03 |
| TMEM59L | NM_012109 | 1,19 | 3,39E-03 |
| C9orf64 | NM_032307 | -1,06 | 3,42E-03 |
| RSPH9 | NM_152732 | 2,00 | 3,44E-03 |
| MYOM1 | NM_019856 | 1,48 | 3,47E-03 |
| FBXO43 | NM_001029860 | -1,62 | 3,48E-03 |
| LSM5 | NM_001130710 | -0,71 | 3,49E-03 |
| NUP210 | NM_024923 | 1,04 | 3,50E-03 |
| PEX12 | NM_000286 | 0,72 | 3,52E-03 |
| LOC100134259 | NR_024452 | 1,57 | 3,52E-03 |
| DNAJC27 | NM_016544 | 0,96 | 3,53E-03 |
| HTR7P | NR_002774 | 0,81 | 3,54E-03 |
| ZNF702P | NR_003578 | -0,97 | 3,55E-03 |
| SEC22C | NM_032970 | -0,69 | 3,55E-03 |
| PAPD5 | NM_001040285 | -0,64 | 3,58E-03 |
| NEFH | NM_021076 | -1,19 | 3,60E-03 |
| SUSD2 | NM_019601 | 0,84 | 3,61E-03 |
| TNFRSF10A | NM_003844 | -1,03 | 3,62E-03 |
| ENOX1 | NM_017993 | 1,30 | 3,64E-03 |
| ZNF627 | NM_145295 | 0,64 | 3,64E-03 |
| THAP2 | NM_031435 | 0,89 | 3,64E-03 |
| SH2B2 | NM_020979 | 0,93 | 3,65E-03 |
| SEMA4A | NM_022367 | 1,63 | 3,65E-03 |
| LOC100271831 | NR_027081 | 0,67 | 3,65E-03 |
| C20orf160 | NM_080625 | -2,40 | 3,65E-03 |
| FLJ37453 | NR_024279 | 1,00 | 3,67E-03 |
| CARD14 | NM_024110 | 2,03 | 3,67E-03 |
| TMEM90B | NM_024893 | 2,48 | 3,68E-03 |
| MIR621 | NR_030352 | -1,85 | 3,68E-03 |
| SRRD | NM_001013694 | -0,69 | 3,68E-03 |
| ZNF815 | NR_023382 | 2,14 | 3,70E-03 |
| C22orf25 | NM_152906 | -0,63 | 3,70E-03 |
| S100A3 | NM_002960 | -0,89 | 3,72E-03 |
| ZNF226 | NM_001032372 | 0,84 | 3,74E-03 |
| TM2D3 | NM_025141 | 0,76 | 3,74E-03 |
| HNMT | NM_006895 | 1,68 | 3,75E-03 |
| HSN2 | NM_213655 | 1,47 | 3,77E-03 |
| PTPLB | NM_198402 | -0,99 | 3,79E-03 |
| ZNF34 | NM_030580 | 0,80 | 3,79E-03 |
| PSIMCT-1 | NR_003677 | -1,04 | 3,80E-03 |
| PLIN4 | NM_001080400 | 1,66 | 3,80E-03 |
| TRPA1 | NM_007332 | 1,72 | 3,81E-03 |
| ZNF420 | NM_144689 | 1,01 | 3,81E-03 |
| ST7OT2 | NR_002331 | -0,97 | 3,82E-03 |
| IL17RE | NM_153481 | -1,12 | 3,83E-03 |
| SLC4A4 | NM_001134742 | 0,62 | 3,84E-03 |
| CASP1 | NM_033293 | 2,19 | 3,87E-03 |
| TM7SF4 | NM_030788 | 2,23 | 3,87E-03 |
| C16orf48 | NM_032140 | 0,66 | 3,88E-03 |
| C6orf154 | NM_001012974 | 2,11 | 3,91E-03 |
| LOC284233 | NR_026756 | 2,49 | 3,93E-03 |
| RASSF5 | NM_182663 | 1,29 | 3,93E-03 |
| C14orf118 | NM_017926 | -0,63 | 3,94E-03 |
| NR5A2 | NM_205860 | -2,47 | 3,95E-03 |
| FAM27B | NR_027422 | -1,32 | 3,95E-03 |
| CYB5RL | NM_001031672 | 0,97 | 3,95E-03 |
| RHOU | NM_021205 | 1,68 | 3,96E-03 |
| SNRNP25 | NM_024571 | -0,71 | 3,96E-03 |
| TMTC2 | NM_152588 | 0,83 | 3,98E-03 |
| LOC145820 | NR_027133 | 1,86 | 3,99E-03 |
| LOC100132287 | NR_028322 | 1,04 | 4,00E-03 |
| LOC100132062 | NR_028325 | 1,04 | 4,00E-03 |
| SMPDL3A | NM_006714 | 1,25 | 4,05E-03 |
| FAM113B | NM_138371 | 1,23 | 4,05E-03 |
| CNIH4 | NM_014184 | -0,78 | 4,05E-03 |
| ERCC4 | NM_005236 | 0,69 | 4,06E-03 |
| CYP2D7P1 | NR_002570 | 1,73 | 4,07E-03 |
| KLHL38 | NM_001081675 | 2,47 | 4,07E-03 |
| DMD | NM_004006 | 0,62 | 4,08E-03 |
| MGC16703 | NR_003608 | 1,77 | 4,08E-03 |
| TLR9 | NM_017442 | 2,39 | 4,08E-03 |
| PMCH | NM_002674 | -1,36 | 4,09E-03 |
| SLC38A7 | NM_018231 | 0,65 | 4,09E-03 |
| LOC100288730 | NR_029383 | 0,91 | 4,09E-03 |
| BCL9 | NM_004326 | 0,67 | 4,13E-03 |
| LOC388588 | NM_001163724 | 2,55 | 4,13E-03 |
| C9orf167 | NM_017723 | -0,63 | 4,13E-03 |
| ARSE | NM_000047 | 1,27 | 4,13E-03 |
| STC1 | NM_003155 | -0,67 | 4,13E-03 |
| IFI6 | NM_022872 | 0,75 | 4,15E-03 |
| PCDHB16 | NM_020957 | 2,14 | 4,17E-03 |
| MYLK4 | NM_001012418 | 2,01 | 4,17E-03 |
| sep-01 | NM_052838 | 1,24 | 4,19E-03 |
| LOC653390 | NR_003369 | -1,17 | 4,19E-03 |
| ZNF167 | NM_018651 | 1,01 | 4,24E-03 |
| GIMAP2 | NM_015660 | -2,45 | 4,24E-03 |
| CCDC78 | NM_001031737 | 1,31 | 4,26E-03 |
| C14orf28 | NM_001017923 | 1,01 | 4,26E-03 |
| C5 | NM_001735 | 1,20 | 4,27E-03 |
| ZNF568 | NM_198539 | 1,04 | 4,29E-03 |
| MGST1 | NM_020300 | -0,75 | 4,29E-03 |
| U2AF1L4 | NM_001040425 | 0,91 | 4,31E-03 |
| ZNF329 | NM_024620 | 0,62 | 4,32E-03 |
| MAPK12 | NM_002969 | -0,70 | 4,32E-03 |
| MARK1 | NM_018650 | 0,79 | 4,35E-03 |
| ZNF135 | NM_001164530 | 1,28 | 4,38E-03 |
| PABPC5 | NM_080832 | 1,58 | 4,39E-03 |
| WNK4 | NM_032387 | -1,09 | 4,42E-03 |
| C14orf21 | NM_174913 | -0,66 | 4,43E-03 |
| LAGE3 | NM_006014 | 0,68 | 4,43E-03 |
| C9orf95 | NM_001127603 | 0,93 | 4,45E-03 |
| TFAP4 | NM_003223 | -0,67 | 4,46E-03 |
| TUFT1 | NM_020127 | -0,63 | 4,47E-03 |
| FAM40B | NM_020704 | -1,27 | 4,48E-03 |
| CYP2R1 | NM_024514 | 1,00 | 4,49E-03 |
| NDUFAB1 | NM_005003 | -0,60 | 4,49E-03 |
| SLC36A4 | NM_152313 | -0,64 | 4,49E-03 |
| NPAT | NM_002519 | -0,71 | 4,49E-03 |
| UBL4B | NM_203412 | 2,45 | 4,49E-03 |
| POM121L1P | NR_024591 | 1,93 | 4,51E-03 |
| LGR5 | NM_003667 | 0,71 | 4,54E-03 |
| ZNF461 | NM_153257 | 0,95 | 4,54E-03 |
| LOC100129387 | NR_024490 | 0,90 | 4,56E-03 |
| FLJ45445 | NR_028324 | 1,11 | 4,56E-03 |
| SNX11 | NM_152244 | -0,72 | 4,60E-03 |
| RBX1 | NM_014248 | -0,61 | 4,61E-03 |
| ZNF680 | NM_178558 | -1,06 | 4,61E-03 |
| ZC3HAV1L | NM_080660 | -0,73 | 4,63E-03 |
| YY2 | NM_206923 | -1,09 | 4,65E-03 |
| TRIM69 | NM_182985 | 1,91 | 4,65E-03 |
| GOLSYN | NM_001099756 | -1,93 | 4,65E-03 |
| FABP3 | NM_004102 | -1,23 | 4,65E-03 |
| C5orf25 | NM_198567 | -0,68 | 4,66E-03 |
| C1orf89 | NM_030907 | 1,25 | 4,73E-03 |
| C18orf1 | NM_181482 | 2,16 | 4,78E-03 |
| GULP1 | NM_016315 | 0,67 | 4,83E-03 |
| LRRC16A | NM_017640 | 0,83 | 4,83E-03 |
| DHRS13 | NM_144683 | 0,92 | 4,83E-03 |
| PAQR3 | NM_001040202 | -0,61 | 4,85E-03 |
| TMEM86A | NM_153347 | 1,51 | 4,85E-03 |
| MMP17 | NM_016155 | -0,77 | 4,87E-03 |
| BIRC7 | NM_139317 | 2,51 | 4,88E-03 |
| HLA-G | NM_002127 | 0,72 | 4,88E-03 |
| MDH1B | NM_001039845 | 1,68 | 4,91E-03 |
| FLJ43663 | NR_015431 | 1,70 | 4,92E-03 |
| SBK1 | NM_001024401 | 2,01 | 4,94E-03 |
| PDRG1 | NM_030815 | -0,68 | 4,95E-03 |
| MRPL14 | NM_032111 | -0,65 | 4,96E-03 |
| KCNK12 | NM_022055 | 2,32 | 4,99E-03 |
| NAP1L3 | NM_004538 | 0,85 | 4,99E-03 |
| ZNF578 | NM_001099694 | 1,20 | 5,01E-03 |
| C1QTNF2 | NM_031908 | -2,23 | 5,01E-03 |
| COL9A2 | NM_001852 | 2,16 | 5,05E-03 |
| SCNN1A | NM_001159576 | 1,84 | 5,07E-03 |
| HBA2 | NM_000517 | 2,19 | 5,09E-03 |
| PRAM1 | NM_032152 | 2,42 | 5,10E-03 |
| RALGAPA1 | NM_014990 | 0,70 | 5,11E-03 |
| FKBP1P1 | NR_024162 | -0,60 | 5,11E-03 |
| EDARADD | NM_080738 | -1,43 | 5,12E-03 |
| C1orf56 | NM_017860 | 0,74 | 5,16E-03 |
| PTPRQ | NM_001145026 | -2,30 | 5,17E-03 |
| FAM50B | NM_012135 | 0,82 | 5,19E-03 |
| GLIPR1L2 | NM_152436 | 2,41 | 5,24E-03 |
| SVEP1 | NM_153366 | 0,99 | 5,26E-03 |
| ABCC6P2 | NR_023387 | 2,41 | 5,27E-03 |
| SERPINB7 | NM_001040147 | -2,49 | 5,27E-03 |
| RTN4RL1 | NM_178568 | 2,35 | 5,28E-03 |
| C2orf89 | NM_001080824 | -1,22 | 5,28E-03 |
| JPH4 | NM_001146028 | 2,41 | 5,28E-03 |
| TNFSF15 | NM_005118 | 1,86 | 5,29E-03 |
| SLC6A1 | NM_003042 | 2,49 | 5,31E-03 |
| PLK5P | NR_026557 | 2,49 | 5,31E-03 |
| A4GNT | NM_016161 | 2,32 | 5,33E-03 |
| C1orf66 | NM_015997 | 0,74 | 5,33E-03 |
| ZFP3 | NM_153018 | 0,86 | 5,35E-03 |
| CDNF | NM_001029954 | 2,09 | 5,37E-03 |
| PYCARD | NM_013258 | 1,25 | 5,37E-03 |
| MC1R | NM_002386 | 0,64 | 5,37E-03 |
| CYorf15B | NM_032576 | 0,77 | 5,38E-03 |
| CXorf23 | NM_198279 | 0,83 | 5,41E-03 |
| SGCD | NM_000337 | 2,33 | 5,42E-03 |
| DKFZp686O24166 | NR_026750 | -0,71 | 5,42E-03 |
| LOC646214 | NR_027053 | -0,69 | 5,42E-03 |
| POSTN | NM_001135934 | 1,29 | 5,43E-03 |
| ARNTL | NM_001030273 | 0,74 | 5,45E-03 |
| ECHDC3 | NM_024693 | 1,01 | 5,50E-03 |
| SUPT3H | NM_181356 | 0,81 | 5,50E-03 |
| TMCC3 | NM_020698 | -1,61 | 5,53E-03 |
| WDYHV1 | NM_018024 | -0,80 | 5,53E-03 |
| CCDC142 | NM_032779 | 0,66 | 5,54E-03 |
| HESX1 | NM_003865 | 2,40 | 5,57E-03 |
| SFMBT2 | NM_001029880 | 1,35 | 5,58E-03 |
| LOC643837 | NR_015368 | -0,96 | 5,58E-03 |
| METTL6 | NM_152396 | -0,70 | 5,61E-03 |
| C2orf74 | NM_001143960 | 0,67 | 5,62E-03 |
| QRICH2 | NM_032134 | 0,73 | 5,63E-03 |
| KLRAQ1 | NM_001135630 | 0,68 | 5,63E-03 |
| LOC654433 | NR_015377 | 1,98 | 5,64E-03 |
| TMEM190 | NM_139172 | 2,41 | 5,66E-03 |
| MRPS17 | NM_015969 | -0,81 | 5,68E-03 |
| MACROD2 | NM_001033087 | 2,40 | 5,70E-03 |
| FGF20 | NM_019851 | -2,39 | 5,71E-03 |
| ELK4 | NM_021795 | 0,62 | 5,72E-03 |
| EPB41L3 | NM_012307 | -0,68 | 5,74E-03 |
| PSG4 | NM_002780 | -2,26 | 5,76E-03 |
| ABI3 | NM_001135186 | -1,42 | 5,77E-03 |
| ZNF133 | NM_003434 | 0,63 | 5,77E-03 |
| ZNF20 | NM_021143 | 0,96 | 5,79E-03 |
| SNHG4 | NR_003141 | -1,52 | 5,79E-03 |
| ZSCAN12L1 | NR_024063 | 1,81 | 5,82E-03 |
| MORN4 | NM_178832 | 0,80 | 5,82E-03 |
| LRRC6 | NM_012472 | 2,47 | 5,82E-03 |
| PPIL3 | NM_032472 | 0,70 | 5,84E-03 |
| TNFRSF1B | NM_001066 | -1,47 | 5,85E-03 |
| EIF2C4 | NM_017629 | 0,67 | 5,89E-03 |
| CILP | NM_003613 | 2,38 | 5,89E-03 |
| BCS1L | NM_001079866 | -0,64 | 5,89E-03 |
| EFCAB4B | NM_001144958 | -1,31 | 5,92E-03 |
| SEC11C | NM_033280 | -0,77 | 5,96E-03 |
| CPNE5 | NM_020939 | -1,81 | 5,96E-03 |
| RAB27A | NM_183234 | -0,76 | 5,96E-03 |
| FIG4 | NM_014845 | 0,62 | 5,98E-03 |
| LOC100131193 | NR_024580 | 1,14 | 5,98E-03 |
| DPP4 | NM_001935 | -2,01 | 6,00E-03 |
| TSPAN31 | NM_005981 | 0,64 | 6,01E-03 |
| AR | NM_000044 | 0,76 | 6,02E-03 |
| LOC285696 | NR_027253 | 2,13 | 6,07E-03 |
| USE1 | NM_018467 | 0,71 | 6,08E-03 |
| SMCR7 | NM_148886 | 0,71 | 6,08E-03 |
| FBXO24 | NM_012172 | 1,79 | 6,11E-03 |
| ZNF491 | NM_152356 | 1,36 | 6,11E-03 |
| FAM70B | NM_182614 | -1,64 | 6,12E-03 |
| ZNRF2 | NM_147128 | 0,84 | 6,12E-03 |
| C1QTNF5 | NM_015645 | -1,35 | 6,14E-03 |
| MFRP | NM_031433 | -1,35 | 6,14E-03 |
| PIH1D2 | NM_138789 | 1,80 | 6,15E-03 |
| DDIT4L | NM_145244 | 2,16 | 6,17E-03 |
| ZNF790 | NM_206894 | 1,08 | 6,17E-03 |
| SPATA1 | NM_001081472 | 1,24 | 6,19E-03 |
| C16orf46 | NM_001100873 | 1,59 | 6,23E-03 |
| NLRP10 | NM_176821 | -2,11 | 6,26E-03 |
| LOC730101 | NR_024403 | 0,61 | 6,27E-03 |
| CCDC138 | NM_144978 | -1,12 | 6,29E-03 |
| HTR6 | NM_000871 | 2,45 | 6,29E-03 |
| NDN | NM_002487 | 1,09 | 6,29E-03 |
| C9orf169 | NM_199001 | 1,83 | 6,30E-03 |
| DDI2 | NM_032341 | -0,77 | 6,31E-03 |
| SNORD57 | NR_002738 | -2,07 | 6,34E-03 |
| C10orf88 | NM_024942 | -0,66 | 6,34E-03 |
| CCDC114 | NM_144577 | 1,97 | 6,41E-03 |
| MANEAL | NM_001113482 | 0,87 | 6,43E-03 |
| CRLF1 | NM_004750 | 0,72 | 6,43E-03 |
| OFD1 | NM_003611 | 0,63 | 6,48E-03 |
| TNNI1 | NM_003281 | 2,44 | 6,50E-03 |
| PRAF2 | NM_007213 | 1,12 | 6,51E-03 |
| PARD3B | NM_152526 | 0,81 | 6,53E-03 |
| TADA2A | NM_001166105 | -0,79 | 6,57E-03 |
| RET | NM_020630 | 1,80 | 6,58E-03 |
| KRTAP2-4 | NM_033184 | -2,43 | 6,60E-03 |
| KRT15 | NM_002275 | -2,11 | 6,62E-03 |
| PCDHB5 | NM_015669 | 1,77 | 6,67E-03 |
| SEMA3B | NM_004636 | 0,88 | 6,78E-03 |
| SERPINA3 | NM_001085 | 1,26 | 6,85E-03 |
| TMCO4 | NM_181719 | 0,71 | 6,90E-03 |
| TFEC | NM_001018058 | -2,19 | 6,90E-03 |
| RSC1A1 | NM_006511 | -0,65 | 6,92E-03 |
| THAP10 | NM_020147 | -0,93 | 6,93E-03 |
| ALG10 | NM_032834 | -1,04 | 6,96E-03 |
| C7orf63 | NM_001039706 | 1,83 | 7,01E-03 |
| NAPSA | NM_004851 | 2,41 | 7,15E-03 |
| WDR88 | NM_173479 | 1,90 | 7,15E-03 |
| ARPP-21 | NM_016300 | 1,79 | 7,17E-03 |
| ABCB7 | NM_004299 | 0,70 | 7,21E-03 |
| ZNF107 | NM_001013746 | -0,72 | 7,21E-03 |
| PHEX | NM_000444 | 1,76 | 7,21E-03 |
| PSMA3 | NM_002788 | -0,61 | 7,28E-03 |
| C15orf58 | NM_001013657 | 1,52 | 7,28E-03 |
| TSSK2 | NM_053006 | -1,37 | 7,29E-03 |
| TNNI3K | NM_015978 | 2,34 | 7,30E-03 |
| DPY19L2 | NM_173812 | 0,86 | 7,31E-03 |
| GFRA1 | NM_005264 | -0,69 | 7,42E-03 |
| C17orf106 | NM_001113324 | 0,66 | 7,44E-03 |
| ATP9B | NM_198531 | 0,61 | 7,45E-03 |
| HSPC159 | NM_014181 | 0,70 | 7,52E-03 |
| LOC644538 | NM_001163438 | -0,92 | 7,59E-03 |
| C2 | NM_000063 | 1,81 | 7,60E-03 |
| KLHL15 | NM_030624 | 0,63 | 7,62E-03 |
| LOC729234 | NR_003698 | 1,60 | 7,62E-03 |
| C2orf16 | NM_032266 | 1,29 | 7,62E-03 |
| KLF8 | NM_007250 | 1,85 | 7,63E-03 |
| LRRN4CL | NM_203422 | 1,71 | 7,63E-03 |
| REC8 | NM_005132 | 1,19 | 7,66E-03 |
| TP53TG1 | NR_015381 | 1,25 | 7,69E-03 |
| IL26 | NM_018402 | -1,46 | 7,70E-03 |
| ARSG | NM_014960 | 1,04 | 7,73E-03 |
| RASAL3 | NM_022904 | -2,14 | 7,74E-03 |
| C17orf61 | NM_152766 | 0,62 | 7,77E-03 |
| ZNF383 | NM_152604 | 1,09 | 7,77E-03 |
| NRG2 | NM_013982 | 1,80 | 7,86E-03 |
| POP4 | NR_027368 | -0,60 | 7,90E-03 |
| RENBP | NM_002910 | 2,00 | 7,90E-03 |
| SH2D4A | NM_022071 | -1,02 | 7,93E-03 |
| FOS | NM_005252 | -0,61 | 7,93E-03 |
| TMSB10 | NM_021103 | -0,61 | 7,95E-03 |
| HIST1H4B | NM_003544 | -2,38 | 7,96E-03 |
| CAND2 | NM_001162499 | 0,66 | 7,99E-03 |
| IQCB1 | NM_001023571 | -0,65 | 8,02E-03 |
| LIN52 | NM_001024674 | -0,83 | 8,03E-03 |
| FAS | NR_028034 | 0,77 | 8,03E-03 |
| ZFP30 | NM_014898 | 0,65 | 8,03E-03 |
| KIAA1984 | NM_001039374 | 1,51 | 8,04E-03 |
| CCIN | NM_005893 | -1,77 | 8,05E-03 |
| TEX9 | NM_198524 | 1,74 | 8,05E-03 |
| A2LD1 | NM_033110 | 1,09 | 8,05E-03 |
| RPS6KL1 | NM_031464 | -1,87 | 8,11E-03 |
| ZNF214 | NM_013249 | 2,23 | 8,13E-03 |
| MFSD7 | NM_032219 | 1,33 | 8,13E-03 |
| LOC151534 | NR_024606 | 1,30 | 8,15E-03 |
| LAMB2L | NR_004405 | 2,15 | 8,15E-03 |
| ANKRD53 | NM_001115116 | 1,69 | 8,18E-03 |
| ZDHHC24 | NM_207340 | 0,69 | 8,23E-03 |
| ZNF19 | NM_006961 | 1,30 | 8,23E-03 |
| COX16 | NM_016468 | -0,63 | 8,23E-03 |
| NOXA1 | NM_006647 | 2,13 | 8,23E-03 |
| SNHG5 | NR_003038 | 0,77 | 8,24E-03 |
| ADC | NM_052998 | 1,32 | 8,27E-03 |
| LOC283174 | NR_024344 | 1,87 | 8,28E-03 |
| FLJ39653 | NR_027697 | 1,58 | 8,31E-03 |
| C16orf53 | NM_024516 | 0,60 | 8,31E-03 |
| ABHD14A | NM_015407 | 0,78 | 8,35E-03 |
| LPXN | NM_001143995 | 1,10 | 8,37E-03 |
| MIR17HG | NR_027350 | -0,92 | 8,38E-03 |
| TCF15 | NM_004609 | -2,37 | 8,44E-03 |
| ANO2 | NM_020373 | -2,12 | 8,45E-03 |
| RGS2 | NM_002923 | 1,00 | 8,45E-03 |
| CMTM7 | NM_181472 | -0,68 | 8,46E-03 |
| SHANK1 | NM_016148 | 1,71 | 8,47E-03 |
| INCA1 | NM_213726 | 1,07 | 8,52E-03 |
| MAGOHB | NM_018048 | -0,79 | 8,52E-03 |
| FLJ39739 | NR_027468 | 1,39 | 8,55E-03 |
| DMRTA1 | NM_022160 | 2,12 | 8,67E-03 |
| TFB1M | NM_016020 | -0,83 | 8,69E-03 |
| ST7OT3 | NR_002332 | -1,17 | 8,71E-03 |
| IQCH | NM_022784 | 1,29 | 8,75E-03 |
| ATXN7L2 | NM_153340 | 0,63 | 8,75E-03 |
| WDR27 | NM_182552 | 0,85 | 8,80E-03 |
| ACOT8 | NM_005469 | 0,70 | 8,81E-03 |
| BAI3 | NM_001704 | 2,36 | 8,81E-03 |
| C7orf52 | NM_198571 | 2,36 | 8,82E-03 |
| PTPN20A | NM_001042392 | -2,11 | 8,82E-03 |
| PTPN20B | NM_001042360 | -2,11 | 8,82E-03 |
| SLC7A5P1 | NR_002593 | -1,93 | 8,83E-03 |
| PARD6A | NM_016948 | 1,61 | 8,83E-03 |
| SLC44A3 | NM_001114106 | 1,89 | 8,84E-03 |
| EXOSC4 | NM_019037 | -0,70 | 8,87E-03 |
| C1orf201 | NM_178122 | -0,64 | 8,89E-03 |
| FN3K | NM_022158 | 1,03 | 8,90E-03 |
| FAM86B1 | NR_003494 | -1,01 | 8,91E-03 |
| HIST1H2AL | NM_003511 | -2,24 | 8,92E-03 |
| RAPGEF6 | NM_001164390 | -0,85 | 8,93E-03 |
| C3orf14 | NM_020685 | -0,91 | 8,98E-03 |
| WHAMML2 | NR_026589 | 0,82 | 9,00E-03 |
| EXOSC5 | NM_020158 | -0,67 | 9,11E-03 |
| GCET2 | NM_152785 | -2,02 | 9,13E-03 |
| SPRY3 | NM_005840 | 1,12 | 9,18E-03 |
| TMEM232 | NM_001039763 | 2,06 | 9,19E-03 |
| TDRD1 | NM_198795 | -1,21 | 9,19E-03 |
| GTF2A1L | NM_006872 | 2,11 | 9,21E-03 |
| HIST1H1E | NM_005321 | -1,89 | 9,24E-03 |
| KILLIN | NM_001126049 | 1,59 | 9,34E-03 |
| PRG4 | NM_001127710 | 0,92 | 9,36E-03 |
| CYP2E1 | NM_000773 | 1,65 | 9,36E-03 |
| RPIA | NM_144563 | -0,69 | 9,39E-03 |
| DCLRE1A | NM_014881 | -0,62 | 9,39E-03 |
| RPA3 | NM_002947 | -0,91 | 9,44E-03 |
| GTF2H2 | NM_001515 | -0,70 | 9,46E-03 |
| ADAMTS4 | NM_005099 | -1,18 | 9,47E-03 |
| BMPER | NM_133468 | -1,70 | 9,47E-03 |
| LOC645431 | NR_024334 | 1,71 | 9,48E-03 |
| GNG7 | NM_052847 | 1,43 | 9,50E-03 |
| FBXO15 | NM_152676 | 2,27 | 9,51E-03 |
| FOXD4L1 | NM_012184 | 2,27 | 9,51E-03 |
| C20orf7 | NM_001039375 | -0,88 | 9,52E-03 |
| TRIL | NM_014817 | 1,71 | 9,53E-03 |
| C8orf51 | NR_026785 | 1,86 | 9,54E-03 |
| GTF2H2C | NM_001098728 | -1,01 | 9,57E-03 |
| TMEM106A | NM_145041 | -0,86 | 9,60E-03 |
| C17orf90 | NM_001039842 | 0,74 | 9,63E-03 |
| FBXL2 | NM_012157 | 0,62 | 9,69E-03 |
| GLTPD2 | NM_001014985 | 2,12 | 9,82E-03 |
| ABCG4 | NM_022169 | 2,03 | 9,83E-03 |
| C8orf37 | NM_177965 | 1,03 | 9,89E-03 |
| C9orf72 | NM_145005 | -1,21 | 9,91E-03 |
| SNORD17 | NR_003045 | -1,58 | 9,93E-03 |
| LOC219347 | NR_027431 | 1,49 | 9,95E-03 |
| HIATL2 | NR_002894 | -0,82 | 1,00E-02 |
| TTC30B | NM_152517 | 0,91 | 1,00E-02 |
| CRELD2 | NM_001135101 | -0,67 | 1,01E-02 |
| FICD | NM_007076 | -0,79 | 1,01E-02 |
| MITF | NM_198159 | 0,80 | 1,01E-02 |
| WSCD1 | NM_015253 | -1,21 | 1,01E-02 |
| SIRT4 | NM_012240 | 1,67 | 1,01E-02 |
| AMH | NM_000479 | 1,12 | 1,02E-02 |
| APLF | NM_173545 | 1,08 | 1,02E-02 |
| MRPL22 | NM_001014990 | -0,72 | 1,02E-02 |
| SFXN2 | NM_178858 | -0,87 | 1,02E-02 |
| IQUB | NM_178827 | 2,08 | 1,03E-02 |
| MT1F | NM_005949 | 1,29 | 1,03E-02 |
| CNKSR2 | NM_014927 | -1,94 | 1,03E-02 |
| WBSCR22 | NM_017528 | -0,66 | 1,03E-02 |
| ZNF784 | NM_203374 | 0,78 | 1,03E-02 |
| WBSCR27 | NM_152559 | 0,98 | 1,03E-02 |
| KLHDC9 | NM_152366 | 1,75 | 1,03E-02 |
| FAHD2B | NM_199336 | 0,80 | 1,03E-02 |
| METTL4 | NM_022840 | -0,66 | 1,04E-02 |
| XIST | NR_001564 | 1,14 | 1,05E-02 |
| FAM24B | NM_152644 | -1,22 | 1,05E-02 |
| MT1L | NR_001447 | -1,03 | 1,05E-02 |
| DNAH5 | NM_001369 | -1,93 | 1,05E-02 |
| KEL | NM_000420 | 2,31 | 1,05E-02 |
| AQP3 | NM_004925 | 1,21 | 1,06E-02 |
| LOC146336 | NR_027242 | 2,30 | 1,07E-02 |
| SH3GL3 | NM_003027 | 2,17 | 1,07E-02 |
| CLEC18A | NM_001136214 | 2,23 | 1,08E-02 |
| CDON | NM_016952 | 0,96 | 1,08E-02 |
| ZNF184 | NM_007149 | 0,68 | 1,08E-02 |
| KAZALD1 | NM_030929 | 1,20 | 1,08E-02 |
| XKR6 | NM_173683 | 1,32 | 1,09E-02 |
| ZNF624 | NM_020787 | 0,78 | 1,10E-02 |
| CACNA1B | NM_000718 | 2,10 | 1,10E-02 |
| RAVER2 | NM_018211 | 0,66 | 1,10E-02 |
| MIR614 | NR_030345 | -1,86 | 1,10E-02 |
| SLC4A5 | NM_133478 | -0,67 | 1,10E-02 |
| CIB2 | NM_006383 | 0,79 | 1,11E-02 |
| MUC1 | NM_001018016 | 1,31 | 1,11E-02 |
| FAM184B | NM_015688 | 1,44 | 1,11E-02 |
| PADI2 | NM_007365 | 1,95 | 1,12E-02 |
| CCDC112 | NM_152549 | -0,67 | 1,12E-02 |
| GJD4 | NM_153368 | 2,22 | 1,12E-02 |
| IL6R | NM_000565 | 0,61 | 1,12E-02 |
| RPL32P3 | NR_003111 | 1,01 | 1,12E-02 |
| GNG2 | NM_053064 | 0,80 | 1,13E-02 |
| LOC388242 | NR_002556 | 1,34 | 1,13E-02 |
| LOC613038 | NR_002557 | 1,34 | 1,13E-02 |
| RIBC1 | NM_144968 | 2,07 | 1,13E-02 |
| RNASET2 | NM_003730 | 0,66 | 1,14E-02 |
| KIAA1644 | NM_001099294 | 0,69 | 1,15E-02 |
| ZSCAN23 | NM_001012455 | 1,53 | 1,15E-02 |
| TMEM223 | NM_001080501 | -0,62 | 1,15E-02 |
| HSCB | NM_172002 | 0,95 | 1,15E-02 |
| PAOX | NM_207127 | 1,01 | 1,17E-02 |
| KCNA1 | NM_000217 | 2,02 | 1,17E-02 |
| ZCWPW2 | NM_001040432 | 1,91 | 1,17E-02 |
| LOC100286844 | NR_027501 | 0,95 | 1,17E-02 |
| IL17RD | NM_017563 | 0,97 | 1,18E-02 |
| CCDC23 | NM_199342 | 0,71 | 1,18E-02 |
| TMEM170B | NM_001100829 | 0,89 | 1,18E-02 |
| SIP1 | NM_003616 | -0,99 | 1,19E-02 |
| PCDHA10 | NM_031860 | 1,80 | 1,19E-02 |
| FLJ42709 | NR_015369 | -0,66 | 1,20E-02 |
| IL11RA | NM_147162 | 0,61 | 1,20E-02 |
| CYP21A2 | NM_000500 | 1,52 | 1,21E-02 |
| PLEKHG1 | NM_001029884 | -1,26 | 1,21E-02 |
| C12orf76 | NM_207435 | 0,93 | 1,22E-02 |
| HES6 | NM_018645 | -1,41 | 1,22E-02 |
| HIST1H3E | NM_003532 | 1,93 | 1,23E-02 |
| MTRF1 | NM_004294 | 0,76 | 1,24E-02 |
| BCO2 | NM_001037290 | 2,05 | 1,24E-02 |
| ALDH1A1 | NM_000689 | -2,21 | 1,24E-02 |
| LOC150381 | NR_027034 | -1,52 | 1,24E-02 |
| FAM185A | NM_001145268 | 1,13 | 1,25E-02 |
| MLL5 | NM_182931 | 0,64 | 1,25E-02 |
| LIME1 | NM_017806 | 0,62 | 1,25E-02 |
| LGI4 | NM_139284 | 1,97 | 1,26E-02 |
| ACOXL | NM_001142807 | 2,25 | 1,27E-02 |
| LINGO1 | NM_032808 | -1,46 | 1,27E-02 |
| TF | NM_001063 | 2,25 | 1,28E-02 |
| PLEKHB1 | NM_001130033 | 1,44 | 1,28E-02 |
| ARMC8 | NM_014154 | -0,61 | 1,28E-02 |
| MGAT4A | NM_012214 | -1,45 | 1,29E-02 |
| TMC8 | NM_152468 | 2,20 | 1,29E-02 |
| TCTEX1D4 | NM_001013632 | 1,26 | 1,29E-02 |
| LSM7 | NM_016199 | -0,70 | 1,29E-02 |
| SDHALP1 | NR_003264 | 0,65 | 1,30E-02 |
| HIPK4 | NM_144685 | 1,88 | 1,30E-02 |
| SLAMF8 | NM_020125 | 1,65 | 1,30E-02 |
| RDH13 | NR_027382 | -0,68 | 1,30E-02 |
| GCDH | NM_000159 | -0,67 | 1,31E-02 |
| TIGD7 | NM_033208 | 0,67 | 1,31E-02 |
| C5AR1 | NM_001736 | 1,70 | 1,31E-02 |
| ZNF323 | NM_030899 | 1,25 | 1,31E-02 |
| ANO9 | NM_001012302 | 2,19 | 1,32E-02 |
| PTRH1 | NM_001002913 | -0,92 | 1,32E-02 |
| GPR39 | NM_001508 | -0,74 | 1,33E-02 |
| MCEE | NM_032601 | 0,84 | 1,33E-02 |
| KIF19 | NM_153209 | -2,11 | 1,33E-02 |
| RASSF9 | NM_005447 | 1,15 | 1,33E-02 |
| SPRY1 | NM_199327 | 0,68 | 1,34E-02 |
| CASP12 | NR_000035 | 2,19 | 1,34E-02 |
| PHYHD1 | NM_001100876 | 0,94 | 1,35E-02 |
| ANKMY1 | NM_016552 | 0,93 | 1,35E-02 |
| PLXNC1 | NM_005761 | 1,89 | 1,35E-02 |
| DLX1 | NM_001038493 | -1,79 | 1,35E-02 |
| AARSD1 | NM_001136042 | -0,68 | 1,36E-02 |
| SCD5 | NM_024906 | -0,62 | 1,37E-02 |
| ANO7 | NM_001001891 | -1,30 | 1,37E-02 |
| C9orf116 | NM_144654 | 1,00 | 1,37E-02 |
| SNORD28 | NR_002562 | -2,11 | 1,38E-02 |
| PDGFRL | NM_006207 | 1,06 | 1,38E-02 |
| ZNF124 | NM_003431 | -1,33 | 1,39E-02 |
| C9orf47 | NM_001001938 | 1,15 | 1,40E-02 |
| ZNF311 | NM_001010877 | 1,88 | 1,40E-02 |
| GAS2 | NM_177553 | 2,18 | 1,40E-02 |
| HAPLN4 | NM_023002 | 2,10 | 1,40E-02 |
| CPEB1 | NM_001079534 | 1,89 | 1,41E-02 |
| CENPP | NM_001012267 | -0,64 | 1,41E-02 |
| C17orf69 | NR_026906 | 1,84 | 1,41E-02 |
| LOC285847 | NR_027117 | 2,17 | 1,43E-02 |
| CD72 | NM_001782 | 1,59 | 1,44E-02 |
| KLHL35 | NM_001039548 | 1,07 | 1,44E-02 |
| RERGL | NM_024730 | 2,22 | 1,44E-02 |
| NGDN | NM_001042635 | -0,68 | 1,45E-02 |
| SNORD26 | NR_002564 | -1,82 | 1,46E-02 |
| SPATA17 | NM_138796 | 2,15 | 1,46E-02 |
| DLEU1 | NR_002605 | -0,73 | 1,47E-02 |
| PPAPDC3 | NM_032728 | 0,72 | 1,47E-02 |
| C14orf174 | NM_001010860 | 1,05 | 1,47E-02 |
| ABI3BP | NM_015429 | -0,66 | 1,47E-02 |
| ZBTB24 | NM_014797 | -0,60 | 1,48E-02 |
| ZNF772 | NM_001144068 | 0,69 | 1,48E-02 |
| USP43 | NM_153210 | 2,16 | 1,48E-02 |
| ZNF79 | NM_007135 | 0,76 | 1,49E-02 |
| C1orf92 | NM_144702 | 1,96 | 1,49E-02 |
| DKKL1 | NM_014419 | 2,20 | 1,51E-02 |
| HAUS1 | NR_026978 | -0,65 | 1,51E-02 |
| GATSL1 | NM_001145063 | 1,60 | 1,51E-02 |
| LOC439994 | NR_029408 | 0,63 | 1,52E-02 |
| PCDHB15 | NM_018935 | 1,78 | 1,52E-02 |
| ZNF714 | NM_182515 | -0,75 | 1,53E-02 |
| PXMP4 | NM_007238 | 0,73 | 1,53E-02 |
| PCDHA4 | NM_018907 | 1,75 | 1,55E-02 |
| BATF3 | NM_018664 | -1,43 | 1,55E-02 |
| MYRIP | NM_015460 | -2,15 | 1,56E-02 |
| TRNT1 | NM_182916 | -0,64 | 1,56E-02 |
| C19orf47 | NM_178830 | -0,70 | 1,56E-02 |
| ZNF844 | NM_001136501 | 0,93 | 1,56E-02 |
| LGALS2 | NM_006498 | 1,92 | 1,56E-02 |
| PABPC3 | NM_030979 | 0,89 | 1,57E-02 |
| WDR67 | NM_145647 | -0,67 | 1,57E-02 |
| EAF2 | NM_018456 | -1,93 | 1,57E-02 |
| MATN3 | NM_002381 | -1,39 | 1,57E-02 |
| ZNF789 | NM_213603 | -1,14 | 1,57E-02 |
| RNFT1 | NM_016125 | 0,69 | 1,57E-02 |
| DNMT3B | NM_006892 | -0,67 | 1,58E-02 |
| DHDPSL | NM_138413 | 1,83 | 1,58E-02 |
| TRIMP1 | NR_002777 | -0,63 | 1,59E-02 |
| OR2W3 | NM_001001957 | -1,23 | 1,59E-02 |
| ERI2 | NM_001142725 | -0,64 | 1,62E-02 |
| ZFP28 | NM_020828 | 0,64 | 1,62E-02 |
| KIAA1161 | NM_020702 | -1,01 | 1,62E-02 |
| ZNF467 | NM_207336 | 1,79 | 1,63E-02 |
| KCTD16 | NM_020768 | 1,06 | 1,63E-02 |
| CACNA2D2 | NM_001005505 | 1,55 | 1,63E-02 |
| TRAF1 | NM_005658 | 0,91 | 1,64E-02 |
| DMBT1 | NM_007329 | 1,83 | 1,64E-02 |
| F2RL3 | NM_003950 | -1,99 | 1,64E-02 |
| C17orf42 | NM_024683 | -0,74 | 1,64E-02 |
| MPP6 | NM_016447 | -0,75 | 1,66E-02 |
| FOXD2 | NM_004474 | 0,88 | 1,66E-02 |
| PKIB | NM_032471 | 1,50 | 1,67E-02 |
| CCDC17 | NM_001114938 | 1,87 | 1,67E-02 |
| NAPB | NM_022080 | 0,62 | 1,67E-02 |
| C14orf176 | NM_001146683 | 1,97 | 1,68E-02 |
| ZMYND17 | NM_001024593 | 1,10 | 1,68E-02 |
| INPP5J | NM_001002837 | 1,12 | 1,68E-02 |
| FCGBP | NM_003890 | 2,01 | 1,68E-02 |
| SH3BGRL2 | NM_031469 | 0,82 | 1,68E-02 |
| GTF2H3 | NM_001516 | -0,61 | 1,69E-02 |
| NME5 | NM_003551 | 2,13 | 1,69E-02 |
| RIMBP3 | NM_015672 | 1,30 | 1,69E-02 |
| MECOM | NM_001164000 | -1,03 | 1,69E-02 |
| TRIM7 | NM_203295 | -1,08 | 1,69E-02 |
| HSP90AB2P | NR_003132 | -0,61 | 1,69E-02 |
| STEAP2 | NM_152999 | 1,47 | 1,70E-02 |
| CCDC41 | NM_001042399 | -0,64 | 1,71E-02 |
| CHCHD5 | NM_032309 | 0,73 | 1,71E-02 |
| LSP1 | NM_001013253 | -0,74 | 1,71E-02 |
| GTF2H2D | NM_001042490 | -0,97 | 1,71E-02 |
| TFDP2 | NM_006286 | 0,64 | 1,71E-02 |
| PPARGC1A | NM_013261 | 2,12 | 1,72E-02 |
| P2RY2 | NM_176071 | -2,12 | 1,72E-02 |
| PCDHA13 | NM_018904 | 1,72 | 1,73E-02 |
| LRRC15 | NM_130830 | -2,16 | 1,73E-02 |
| ZNF749 | NM_001023561 | -0,79 | 1,73E-02 |
| LOC554202 | NR_027054 | 1,10 | 1,75E-02 |
| MFAP5 | NM_003480 | -2,16 | 1,75E-02 |
| ZNF2 | NM_001017396 | 0,66 | 1,75E-02 |
| MSTN | NM_005259 | 2,16 | 1,76E-02 |
| DRD4 | NM_000797 | 2,16 | 1,76E-02 |
| SLC22A4 | NM_003059 | 1,09 | 1,76E-02 |
| C1QTNF4 | NM_031909 | 2,11 | 1,76E-02 |
| HVCN1 | NM_032369 | 1,31 | 1,77E-02 |
| C9orf139 | NM_207511 | 2,15 | 1,77E-02 |
| C3orf24 | NM_173472 | -2,11 | 1,78E-02 |
| HSH2D | NM_032855 | -1,85 | 1,79E-02 |
| TMC6 | NM_007267 | 0,91 | 1,79E-02 |
| SCN11A | NM_014139 | 2,15 | 1,80E-02 |
| ZNF474 | NM_207317 | -2,10 | 1,81E-02 |
| OSBPL3 | NM_145320 | -0,68 | 1,81E-02 |
| SAMD13 | NM_001134664 | 2,03 | 1,82E-02 |
| UBQLNL | NM_145053 | 1,89 | 1,82E-02 |
| ZNF793 | NM_001013659 | 1,47 | 1,83E-02 |
| RINL | NM_198445 | 1,46 | 1,83E-02 |
| LRRC46 | NM_033413 | 1,63 | 1,83E-02 |
| NOS3 | NM_000603 | -1,61 | 1,84E-02 |
| EBF2 | NM_022659 | 0,83 | 1,84E-02 |
| LOC100128239 | NR_027276 | 1,97 | 1,84E-02 |
| LHX4 | NM_033343 | 1,34 | 1,84E-02 |
| RGS3 | NM_017790 | -1,13 | 1,84E-02 |
| LOC113230 | NR_024282 | 1,80 | 1,84E-02 |
| sep-04 | NM_080415 | 1,53 | 1,84E-02 |
| SIVA1 | NM_021709 | -0,67 | 1,84E-02 |
| ZNF284 | NM_001037813 | 0,98 | 1,84E-02 |
| ART5 | NM_001079536 | 1,89 | 1,85E-02 |
| KIAA1328 | NM_020776 | 0,86 | 1,85E-02 |
| NEFL | NM_006158 | 0,91 | 1,85E-02 |
| HSFX2 | NM_001164415 | 1,67 | 1,86E-02 |
| HSFX1 | NM_016153 | 1,67 | 1,86E-02 |
| PUS10 | NM_144709 | 0,81 | 1,87E-02 |
| AP3B2 | NM_004644 | 2,14 | 1,88E-02 |
| C14orf128 | NR_027263 | 1,42 | 1,89E-02 |
| LOC728875 | NR_024584 | 0,82 | 1,91E-02 |
| CAMK2N2 | NM_033259 | 1,11 | 1,91E-02 |
| ZNF717 | NM_001128223 | 0,83 | 1,91E-02 |
| SLC16A7 | NM_004731 | -0,74 | 1,92E-02 |
| DNAH12 | NM_178504 | 2,13 | 1,92E-02 |
| C9orf117 | NM_001012502 | -1,12 | 1,92E-02 |
| LOC284023 | NR_024349 | 1,06 | 1,92E-02 |
| SCN4B | NR_024527 | 2,13 | 1,93E-02 |
| NUDT7 | NR_024154 | 1,37 | 1,93E-02 |
| ACVR1C | NM_001111032 | 1,84 | 1,94E-02 |
| PNKD | NM_001077399 | 0,71 | 1,95E-02 |
| XPA | NM_000380 | 0,76 | 1,95E-02 |
| CD163L1 | NM_174941 | -0,83 | 1,97E-02 |
| PHF7 | NM_173341 | 0,87 | 1,98E-02 |
| SLC17A7 | NM_020309 | 2,08 | 1,99E-02 |
| HOPX | NM_001145460 | -1,74 | 1,99E-02 |
| PARP16 | NM_017851 | 0,73 | 2,00E-02 |
| EFNA4 | NM_182690 | 0,67 | 2,01E-02 |
| MGAM | NM_004668 | -2,07 | 2,01E-02 |
| HOXB13 | NM_006361 | 1,86 | 2,02E-02 |
| LOC100216001 | NR_024475 | -1,73 | 2,03E-02 |
| ETV7 | NM_016135 | 2,11 | 2,04E-02 |
| THSD1P | NR_002816 | 0,73 | 2,04E-02 |
| NTF4 | NM_006179 | 2,07 | 2,05E-02 |
| FLJ35024 | NR_015375 | -1,93 | 2,06E-02 |
| LRRC29 | NM_001004055 | 1,26 | 2,07E-02 |
| MPP2 | NM_005374 | 0,67 | 2,08E-02 |
| SNAI3 | NM_178310 | 1,28 | 2,08E-02 |
| LMO2 | NM_001142315 | -1,05 | 2,08E-02 |
| USHBP1 | NM_031941 | -2,10 | 2,09E-02 |
| ZNF221 | NM_013359 | 1,14 | 2,09E-02 |
| FAAH | NM_001441 | 1,62 | 2,10E-02 |
| LOC151174 | NR_026925 | 1,99 | 2,10E-02 |
| TLE6 | NM_024760 | 2,00 | 2,10E-02 |
| SYTL3 | NM_001009991 | -1,02 | 2,10E-02 |
| LOC158381 | NR_003582 | 0,88 | 2,10E-02 |
| HSPB11 | NM_016126 | -0,71 | 2,11E-02 |
| LONRF2 | NM_198461 | 1,16 | 2,11E-02 |
| MGC21881 | NR_015363 | 1,34 | 2,11E-02 |
| SNHG8 | NR_003584 | 0,67 | 2,12E-02 |
| BBS12 | NM_152618 | 0,79 | 2,13E-02 |
| CCDC89 | NM_152723 | 1,25 | 2,14E-02 |
| C19orf18 | NM_152474 | 2,05 | 2,14E-02 |
| SFXN4 | NM_213649 | -0,82 | 2,14E-02 |
| ZNF85 | NM_003429 | -0,96 | 2,15E-02 |
| IL1RAPL1 | NM_014271 | 1,84 | 2,15E-02 |
| PCDHA1 | NM_018900 | 1,63 | 2,15E-02 |
| LOC221442 | NR_026938 | 0,97 | 2,15E-02 |
| SNORA61 | NR_002987 | -1,65 | 2,15E-02 |
| MGC45800 | NR_027107 | 0,80 | 2,17E-02 |
| C2CD4B | NM_001007595 | -1,99 | 2,19E-02 |
| CCL14 | NM_032962 | -1,99 | 2,19E-02 |
| CDYL2 | NM_152342 | -0,60 | 2,19E-02 |
| PIGP | NR_028352 | 0,76 | 2,20E-02 |
| SYTL2 | NM_206930 | 1,10 | 2,21E-02 |
| TDRKH | NM_001083965 | -0,99 | 2,21E-02 |
| SERPINI1 | NM_005025 | 0,89 | 2,21E-02 |
| TSIX | NR_003255 | 1,10 | 2,21E-02 |
| SLC39A8 | NM_001135148 | -1,00 | 2,22E-02 |
| CLDN22 | NM_001111319 | -1,24 | 2,23E-02 |
| LOC150786 | NM_001077637 | -0,80 | 2,24E-02 |
| NUP37 | NM_024057 | -0,61 | 2,24E-02 |
| TMEM88 | NM_203411 | 1,04 | 2,24E-02 |
| GOLGA6L10 | NM_001164465 | 1,60 | 2,26E-02 |
| SAC3D1 | NM_013299 | -0,65 | 2,27E-02 |
| RPLP0P2 | NR_002775 | 0,94 | 2,28E-02 |
| C6orf192 | NM_052831 | 0,92 | 2,28E-02 |
| B9D1 | NM_015681 | 0,75 | 2,28E-02 |
| NIPAL1 | NM_207330 | -1,65 | 2,28E-02 |
| FER | NM_005246 | -0,67 | 2,28E-02 |
| C10orf107 | NM_173554 | 2,05 | 2,28E-02 |
| C9orf103 | NM_001001551 | 1,31 | 2,29E-02 |
| RASL10B | NM_033315 | 0,68 | 2,30E-02 |
| GOLGA6L9 | NM_198181 | 0,76 | 2,31E-02 |
| ALG13 | NM_018466 | -0,71 | 2,31E-02 |
| ASTL | NM_001002036 | 2,07 | 2,31E-02 |
| LOC81691 | NM_001144924 | -0,82 | 2,32E-02 |
| OPLAH | NM_017570 | 0,76 | 2,33E-02 |
| GPR132 | NM_013345 | 2,07 | 2,34E-02 |
| SLC16A8 | NM_013356 | 1,40 | 2,34E-02 |
| EXOSC7 | NR_023353 | -0,79 | 2,34E-02 |
| LOC731275 | NR_029401 | 1,08 | 2,35E-02 |
| PLEK2 | NM_016445 | -1,13 | 2,35E-02 |
| VN1R1 | NM_020633 | 1,97 | 2,35E-02 |
| TSPYL3 | NR_002781 | 1,02 | 2,36E-02 |
| DYNC2LI1 | NM_016008 | 0,85 | 2,36E-02 |
| PCDHA9 | NM_031857 | 1,66 | 2,36E-02 |
| FAM168A | NM_015159 | 0,60 | 2,37E-02 |
| SFT2D1 | NM_145169 | -0,71 | 2,37E-02 |
| EBF4 | NM_001110514 | 1,55 | 2,38E-02 |
| RNMTL1 | NM_018146 | -0,67 | 2,39E-02 |
| FAM86C | NM_001099653 | -0,73 | 2,40E-02 |
| NCRNA00107 | NR_027232 | 1,17 | 2,40E-02 |
| RFX8 | NM_001145664 | -1,35 | 2,41E-02 |
| MRPL33 | NM_004891 | -0,65 | 2,41E-02 |
| HOXD1 | NM_024501 | -2,02 | 2,44E-02 |
| ADCK1 | NM_020421 | 0,63 | 2,44E-02 |
| C19orf57 | NM_024323 | 1,15 | 2,44E-02 |
| ST6GALNAC3 | NM_152996 | -0,91 | 2,45E-02 |
| ACOT2 | NM_006821 | 0,70 | 2,46E-02 |
| CA5B | NM_007220 | 0,65 | 2,46E-02 |
| LDHD | NM_153486 | 1,91 | 2,47E-02 |
| LOC100129726 | NR_027251 | 1,91 | 2,47E-02 |
| LOC158376 | NR_024283 | -2,01 | 2,47E-02 |
| CADM1 | NM_014333 | 0,84 | 2,48E-02 |
| P11 | NM_006025 | 1,81 | 2,48E-02 |
| CDH29 | NM_001007540 | 2,05 | 2,48E-02 |
| HIST1H2BI | NM_003525 | -2,02 | 2,49E-02 |
| MRPS11 | NM_176805 | -0,66 | 2,49E-02 |
| ASTN2 | NM_198187 | 0,82 | 2,49E-02 |
| PPIAL4G | NM_001123068 | -0,81 | 2,50E-02 |
| ALKBH7 | NM_032306 | 0,67 | 2,50E-02 |
| NRN1L | NM_198443 | 2,04 | 2,50E-02 |
| LOC100270804 | NR_026885 | 1,28 | 2,50E-02 |
| LBX2 | NM_001009812 | 1,26 | 2,52E-02 |
| C17orf89 | NM_001086521 | -0,75 | 2,53E-02 |
| SPATA5L1 | NR_027635 | -0,62 | 2,53E-02 |
| CCDC150 | NM_001080539 | -0,84 | 2,54E-02 |
| SENP8 | NM_145204 | 1,04 | 2,55E-02 |
| C1orf70 | NM_001114748 | 1,30 | 2,55E-02 |
| C21orf57 | NM_058181 | 0,75 | 2,57E-02 |
| ANKRD30B | NM_001145029 | -1,84 | 2,57E-02 |
| ZNF385B | NM_152520 | 1,87 | 2,57E-02 |
| JMJD7 | NM_001114632 | 0,88 | 2,59E-02 |
| ZNF547 | NM_173631 | 0,88 | 2,60E-02 |
| CILP2 | NM_153221 | 1,24 | 2,60E-02 |
| LOC100133545 | NR_024471 | 1,87 | 2,60E-02 |
| PHKA1 | NM_001122670 | 0,71 | 2,61E-02 |
| FAM22D | NM_001009610 | 1,14 | 2,61E-02 |
| FBLL1 | NR_024356 | 2,03 | 2,62E-02 |
| WDR69 | NM_178821 | -2,03 | 2,62E-02 |
| STYK1 | NM_018423 | -1,63 | 2,63E-02 |
| SERPING1 | NM_000062 | 1,78 | 2,64E-02 |
| LRRC4C | NM_020929 | 2,02 | 2,64E-02 |
| GLB1L | NM_024506 | 0,79 | 2,65E-02 |
| PTGES | NM_004878 | 1,39 | 2,66E-02 |
| FAM184A | NM_024581 | 1,80 | 2,67E-02 |
| MST4 | NM_016542 | -0,62 | 2,68E-02 |
| TSPAN10 | NM_031945 | 1,08 | 2,69E-02 |
| AUTS2 | NM_015570 | -0,69 | 2,69E-02 |
| SULT1E1 | NM_005420 | -1,28 | 2,69E-02 |
| FAM132A | NM_001014980 | 1,39 | 2,71E-02 |
| ATPBD4 | NM_080650 | -0,87 | 2,72E-02 |
| LOC728411 | NR_027026 | 1,32 | 2,73E-02 |
| MX1 | NM_002462 | 1,01 | 2,74E-02 |
| PGBD4 | NM_152595 | 0,77 | 2,74E-02 |
| JAM2 | NM_021219 | 1,55 | 2,75E-02 |
| CCDC28B | NM_024296 | 0,62 | 2,76E-02 |
| CRYGS | NM_017541 | 1,56 | 2,79E-02 |
| DHX40P | NR_002924 | 0,90 | 2,80E-02 |
| ZSWIM3 | NM_080752 | 0,78 | 2,80E-02 |
| PCDHA11 | NM_018902 | 1,54 | 2,80E-02 |
| ALX3 | NM_006492 | 0,89 | 2,81E-02 |
| HSPC157 | NR_023918 | 0,64 | 2,81E-02 |
| MED11 | NM_001001683 | 0,66 | 2,81E-02 |
| D4S234E | NM_014392 | -1,52 | 2,82E-02 |
| ZNF487 | NR_026693 | 1,22 | 2,82E-02 |
| C6orf165 | NM_001031743 | 1,73 | 2,82E-02 |
| SNORA62 | NR_002324 | -1,58 | 2,82E-02 |
| LPAR5 | NM_001142961 | 2,00 | 2,87E-02 |
| GRRP1 | NM_024869 | -1,77 | 2,88E-02 |
| TMEM200B | NM_001003682 | 0,63 | 2,89E-02 |
| HOXB3 | NM_002146 | -1,29 | 2,90E-02 |
| TM6SF1 | NM_023003 | -0,98 | 2,91E-02 |
| KIF5A | NM_004984 | 1,37 | 2,91E-02 |
| SLC13A3 | NM_022829 | 1,38 | 2,91E-02 |
| GPR35 | NM_005301 | 1,68 | 2,93E-02 |
| ZNF326 | NM_182975 | 0,80 | 2,95E-02 |
| CP | NM_000096 | 1,05 | 2,95E-02 |
| LOC401052 | NM_001008737 | 1,30 | 2,98E-02 |
| TAS2R5 | NM_018980 | 1,89 | 3,01E-02 |
| GPC2 | NM_152742 | -0,78 | 3,04E-02 |
| DSG2 | NM_001943 | 0,72 | 3,04E-02 |
| NOSTRIN | NM_052946 | -1,71 | 3,04E-02 |
| LOC641298 | NR_027154 | -0,66 | 3,06E-02 |
| SLC29A2 | NM_001532 | 0,75 | 3,06E-02 |
| CNGA4 | NM_001037329 | 1,97 | 3,06E-02 |
| LOC100294362 | NR_029376 | 0,95 | 3,07E-02 |
| UCKL1AS | NR_027287 | 0,74 | 3,07E-02 |
| CCDC88C | NM_001080414 | -1,19 | 3,08E-02 |
| VAT1L | NM_020927 | -1,56 | 3,09E-02 |
| DDX31 | NM_138620 | -0,61 | 3,10E-02 |
| F3 | NM_001993 | 1,27 | 3,10E-02 |
| NOX5 | NM_024505 | 1,90 | 3,11E-02 |
| RGS9 | NM_001165933 | 1,85 | 3,11E-02 |
| LOC100302640 | NR_028303 | 1,75 | 3,11E-02 |
| LRP1B | NM_018557 | 1,37 | 3,13E-02 |
| C16orf74 | NM_206967 | -1,25 | 3,13E-02 |
| ANKRD37 | NM_181726 | -0,88 | 3,13E-02 |
| NUDT17 | NM_001012758 | 0,91 | 3,14E-02 |
| C1orf110 | NM_178550 | -0,86 | 3,15E-02 |
| PODN | NM_153703 | 1,18 | 3,16E-02 |
| PCSK6 | NM_138323 | 1,49 | 3,16E-02 |
| HRCT1 | NM_001039792 | -1,50 | 3,16E-02 |
| PRR15 | NM_175887 | -1,68 | 3,17E-02 |
| RANBP17 | NM_022897 | 0,70 | 3,18E-02 |
| TBC1D10C | NM_198517 | 1,94 | 3,19E-02 |
| KBTBD7 | NM_032138 | 0,62 | 3,19E-02 |
| SNORD30 | NR_002561 | -1,75 | 3,19E-02 |
| ACYP1 | NM_203488 | -0,87 | 3,19E-02 |
| NLK | NM_016231 | -0,60 | 3,20E-02 |
| REP15 | NM_001029874 | -1,51 | 3,22E-02 |
| APOD | NM_001647 | -1,71 | 3,22E-02 |
| GPR135 | NM_022571 | 1,03 | 3,22E-02 |
| SLC26A8 | NM_052961 | 1,96 | 3,23E-02 |
| YBX2 | NM_015982 | 1,96 | 3,23E-02 |
| CSN1S1 | NM_001890 | -1,96 | 3,24E-02 |
| PNPLA4 | NM_001142389 | 0,76 | 3,24E-02 |
| CCDC122 | NM_144974 | 1,01 | 3,25E-02 |
| STAT5A | NM_003152 | 0,84 | 3,25E-02 |
| ZKSCAN4 | NM_019110 | 0,75 | 3,25E-02 |
| PCDHA2 | NM_018905 | 1,51 | 3,26E-02 |
| PCDHA7 | NM_018910 | 1,54 | 3,26E-02 |
| RASGRP1 | NM_001128602 | -1,51 | 3,27E-02 |
| SLC23A3 | NM_001144890 | 0,88 | 3,27E-02 |
| SEMA3G | NM_020163 | -1,73 | 3,28E-02 |
| FAM78A | NM_033387 | 0,96 | 3,28E-02 |
| MFSD4 | NM_181644 | 1,70 | 3,29E-02 |
| IGSF3 | NM_001542 | 0,97 | 3,29E-02 |
| PSTK | NM_153336 | 1,00 | 3,30E-02 |
| SYCP2 | NM_014258 | 1,08 | 3,31E-02 |
| STK32C | NM_173575 | 0,62 | 3,31E-02 |
| GMPR | NM_006877 | 0,99 | 3,31E-02 |
| SELP | NM_003005 | -1,95 | 3,32E-02 |
| LOC255512 | NR_029409 | 1,49 | 3,33E-02 |
| FAM103A1 | NM_031452 | -0,81 | 3,34E-02 |
| KIF21A | NM_017641 | -0,81 | 3,34E-02 |
| WNT11 | NM_004626 | 1,80 | 3,35E-02 |
| BPHL | NR_026648 | 0,63 | 3,36E-02 |
| GBGT1 | NM_021996 | 0,78 | 3,37E-02 |
| ZBTB3 | NM_024784 | 0,60 | 3,38E-02 |
| BTBD8 | NM_183242 | 1,88 | 3,38E-02 |
| GRHL1 | NM_198182 | 1,74 | 3,40E-02 |
| TCTEX1D2 | NM_152773 | 0,91 | 3,42E-02 |
| SNORD49A | NR_002744 | -1,88 | 3,43E-02 |
| NPAS4 | NM_178864 | -1,94 | 3,43E-02 |
| C9orf96 | NM_153710 | 1,59 | 3,43E-02 |
| ZBTB8OS | NM_178547 | -0,73 | 3,44E-02 |
| ZNF225 | NM_013362 | 0,74 | 3,45E-02 |
| PPP1R3F | NM_033215 | 0,78 | 3,46E-02 |
| ZNF816A | NM_001031665 | 0,80 | 3,47E-02 |
| ANKRD19 | NR_026868 | 0,92 | 3,49E-02 |
| ZNF765 | NM_001040185 | -0,65 | 3,50E-02 |
| FMNL1 | NM_005892 | 1,41 | 3,50E-02 |
| SNORD22 | NR_000008 | -1,07 | 3,51E-02 |
| SPAG16 | NM_024532 | 0,95 | 3,52E-02 |
| RASGRF2 | NM_006909 | -1,87 | 3,53E-02 |
| KIAA1324 | NM_020775 | 1,33 | 3,54E-02 |
| C18orf45 | NM_032933 | -0,70 | 3,54E-02 |
| CCDC110 | NM_001145411 | 1,35 | 3,54E-02 |
| RBM47 | NM_019027 | 1,33 | 3,55E-02 |
| UNC13A | NM_001080421 | 1,15 | 3,56E-02 |
| ZNF396 | NM_145756 | 1,56 | 3,57E-02 |
| C8G | NM_000606 | 1,90 | 3,57E-02 |
| TOX2 | NM_001098798 | -1,07 | 3,58E-02 |
| KCNF1 | NM_002236 | -1,03 | 3,58E-02 |
| ZNF670 | NM_033213 | -1,11 | 3,59E-02 |
| PSTPIP2 | NM_024430 | -0,76 | 3,59E-02 |
| GPR63 | NM_030784 | -1,45 | 3,59E-02 |
| OGN | NM_033014 | 1,70 | 3,59E-02 |
| MYH15 | NM_014981 | 1,84 | 3,60E-02 |
| CMAH | NR_002174 | 1,17 | 3,62E-02 |
| SOX2 | NM_003106 | 1,91 | 3,64E-02 |
| LOC100132163 | NR_029379 | 0,94 | 3,65E-02 |
| C22orf34 | NR_026997 | -1,90 | 3,65E-02 |
| HOXB7 | NM_004502 | -1,42 | 3,65E-02 |
| PCDHA6 | NM_018909 | 1,53 | 3,66E-02 |
| TMEM90A | NM_001105579 | 1,91 | 3,66E-02 |
| DIRC3 | NR_026597 | 1,91 | 3,67E-02 |
| DNAJC25 | NM_001015882 | -0,64 | 3,67E-02 |
| KATNAL2 | NM_031303 | 1,52 | 3,72E-02 |
| SYT8 | NM_138567 | 1,90 | 3,72E-02 |
| MRPL42P5 | NR_002208 | 1,71 | 3,73E-02 |
| NCRNA00176 | NR_027687 | 1,51 | 3,75E-02 |
| CCDC62 | NM_201435 | 1,79 | 3,75E-02 |
| C12orf39 | NM_030572 | 1,77 | 3,76E-02 |
| DNAH17 | NM_173628 | 0,74 | 3,76E-02 |
| MGC12916 | NR_026880 | -1,70 | 3,77E-02 |
| FZD5 | NM_003468 | -0,92 | 3,77E-02 |
| C8orf31 | NM_173687 | 1,55 | 3,78E-02 |
| TNFRSF4 | NM_003327 | -1,90 | 3,79E-02 |
| OR51E1 | NM_152430 | -1,90 | 3,79E-02 |
| SLC15A2 | NM_021082 | 1,77 | 3,79E-02 |
| LOC90784 | NR_026984 | -0,62 | 3,80E-02 |
| IL18BP | NM_001145057 | 0,92 | 3,80E-02 |
| C16orf71 | NM_139170 | 1,35 | 3,81E-02 |
| RSPH10B | NM_173565 | 1,90 | 3,82E-02 |
| CYP2J2 | NM_000775 | 1,90 | 3,82E-02 |
| RSPH10B2 | NM_001099697 | 1,90 | 3,82E-02 |
| RPS6KA1 | NM_001006665 | -0,76 | 3,82E-02 |
| ATG4A | NM_178270 | -0,60 | 3,88E-02 |
| ZNF429 | NM_001001415 | 0,95 | 3,88E-02 |
| ZSWIM5 | NM_020883 | 1,18 | 3,88E-02 |
| CASC1 | NM_001082973 | 1,82 | 3,90E-02 |
| MIR199A2 | NR_029618 | 1,82 | 3,91E-02 |
| ZDHHC23 | NM_173570 | 1,43 | 3,91E-02 |
| PYGM | NM_005609 | 1,58 | 3,91E-02 |
| LOC100130581 | NR_027413 | 1,11 | 3,93E-02 |
| CYP24A1 | NM_001128915 | 1,89 | 3,93E-02 |
| FAM92B | NM_198491 | 1,89 | 3,93E-02 |
| LOC441089 | NR_003665 | -0,93 | 3,99E-02 |
| MYO1H | NM_001101421 | -1,87 | 4,00E-02 |
| MYLK2 | NM_033118 | -1,19 | 4,00E-02 |
| LYRM4 | NM_001164840 | -0,94 | 4,03E-02 |
| ZG16B | NM_145252 | 1,65 | 4,03E-02 |
| ACRV1 | NM_001612 | -1,88 | 4,03E-02 |
| STK19 | NM_032454 | 0,77 | 4,05E-02 |
| HLA-F | NM_018950 | 0,86 | 4,07E-02 |
| MIOX | NM_017584 | 1,86 | 4,08E-02 |
| CD96 | NM_005816 | 1,87 | 4,10E-02 |
| ZNF709 | NM_001145647 | 0,85 | 4,11E-02 |
| PEX11A | NM_003847 | 1,13 | 4,13E-02 |
| FAM10A4 | NR_002183 | 0,60 | 4,13E-02 |
| KANK3 | NM_198471 | -0,80 | 4,16E-02 |
| CCDC109B | NM_017918 | -0,70 | 4,17E-02 |
| ZNF613 | NM_024840 | 0,83 | 4,17E-02 |
| SLC25A33 | NM_032315 | -0,66 | 4,17E-02 |
| NKAPL | NM_001007531 | 1,11 | 4,20E-02 |
| HEY1 | NM_012258 | 0,75 | 4,20E-02 |
| ACP6 | NM_016361 | 0,80 | 4,20E-02 |
| FAM173B | NM_199133 | -0,60 | 4,21E-02 |
| C8orf44 | NM_019607 | 1,07 | 4,21E-02 |
| BNIPL | NM_001159642 | 1,48 | 4,22E-02 |
| PRCD | NM_001077620 | 1,81 | 4,22E-02 |
| NT5M | NM_020201 | 1,01 | 4,25E-02 |
| STRBP | NM_018387 | -0,70 | 4,27E-02 |
| RADIL | NM_018059 | -0,80 | 4,32E-02 |
| C10orf116 | NM_006829 | 1,07 | 4,33E-02 |
| FAM13AOS | NR_002806 | 0,78 | 4,35E-02 |
| KLKB1 | NM_000892 | 1,84 | 4,36E-02 |
| LOC644936 | NR_004845 | -1,85 | 4,37E-02 |
| LOC100271722 | NR_027036 | 1,01 | 4,38E-02 |
| SYN2 | NM_003178 | 1,81 | 4,38E-02 |
| C9orf46 | NM_018465 | -0,88 | 4,40E-02 |
| HIST1H3I | NM_003533 | -1,84 | 4,42E-02 |
| BCKDHB | NM_000056 | 0,76 | 4,42E-02 |
| C15orf37 | NR_028330 | 0,76 | 4,43E-02 |
| CASP10 | NM_032977 | -0,91 | 4,48E-02 |
| MIR155HG | NR_001458 | -1,24 | 4,51E-02 |
| DNLZ | NM_001080849 | -0,73 | 4,51E-02 |
| LOC100288778 | NR_028269 | 0,61 | 4,53E-02 |
| CD7 | NM_006137 | 1,77 | 4,54E-02 |
| HLX | NM_021958 | -0,62 | 4,54E-02 |
| OCIAD2 | NM_152398 | -0,80 | 4,55E-02 |
| ARID3B | NM_006465 | -0,71 | 4,56E-02 |
| ULBP1 | NM_025218 | -0,94 | 4,56E-02 |
| CACHD1 | NM_020925 | -0,61 | 4,57E-02 |
| NEK5 | NM_199289 | 1,83 | 4,59E-02 |
| RIBC2 | NM_015653 | -1,56 | 4,60E-02 |
| RASD2 | NM_014310 | 1,70 | 4,61E-02 |
| C3AR1 | NM_004054 | 1,70 | 4,63E-02 |
| ASPN | NM_017680 | 1,49 | 4,64E-02 |
| CHD5 | NM_015557 | 1,71 | 4,65E-02 |
| AGAP4 | NM_133446 | 0,89 | 4,65E-02 |
| CCL14-CCL15 | NR_027921 | -1,73 | 4,66E-02 |
| PIPOX | NM_016518 | 1,78 | 4,66E-02 |
| PROM2 | NM_001165978 | 1,61 | 4,68E-02 |
| C22orf26 | NM_018280 | 1,23 | 4,69E-02 |
| IMPG2 | NM_016247 | 1,70 | 4,70E-02 |
| FAM7A1 | NR_026858 | 1,82 | 4,72E-02 |
| FAM7A2 | NR_027470 | 1,82 | 4,72E-02 |
| GPR75 | NM_006794 | -1,12 | 4,74E-02 |
| HBA1 | NM_000558 | 1,77 | 4,76E-02 |
| ZFP2 | NM_030613 | 1,81 | 4,79E-02 |
| CCDC157 | NM_001017437 | 0,73 | 4,81E-02 |
| SLC38A6 | NM_153811 | 0,69 | 4,81E-02 |
| C7orf53 | NM_182597 | 1,45 | 4,81E-02 |
| FAM81A | NM_152450 | -1,81 | 4,85E-02 |
| CCDC75 | NM_174931 | -0,69 | 4,87E-02 |
| CLDN1 | NM_021101 | -1,05 | 4,88E-02 |
| RIMBP3C | NM_001128633 | 1,22 | 4,89E-02 |
| RIMBP3B | NM_001128635 | 1,22 | 4,89E-02 |
| DPM3 | NM_018973 | 0,77 | 4,89E-02 |
| LIPT1 | NM_145197 | 0,77 | 4,90E-02 |
| LOH12CR1 | NM_058169 | 0,65 | 4,91E-02 |
| AVPI1 | NM_021732 | 0,71 | 4,91E-02 |
| ALX4 | NM_021926 | 0,90 | 4,92E-02 |
| INPP4B | NM_003866 | 0,68 | 4,92E-02 |
| FLJ25006 | NM_144610 | -1,05 | 4,93E-02 |
| BMP8A | NM_181809 | 0,95 | 4,96E-02 |
| ERBB3 | NM_001982 | -1,33 | 4,96E-02 |
| ZC3H12B | NM_001010888 | 1,37 | 4,97E-02 |
| IRAK1BP1 | NM_001010844 | 1,15 | 4,97E-02 |
| HIST1H2BO | NM_003527 | -1,80 | 4,97E-02 |

**Supplemental Table 4:** Differential expression of TP53-dependent target genes in co-cultured pericytes

| Gene ID | Refseq ID | Log2FC | P Adjusted | TP53 Prediction |
| --- | --- | --- | --- | --- |
| CX3CL1 | NM_002996 | -4,75 | 3,8E-18 | Inhibited |
| RARRES3 | NM_004585 | -4,21 | 1,6E-13 | Inhibited |
| MST1 | NM_020998 | -3,95 | 9,1E-27 | Inhibited |
| BDKRB2 | NM_000623 | -3,92 | 8,4E-10 | Inhibited |
| PRKN | NM_013987 | -3,79 | 1,1E-08 | Inhibited |
| DBP | NM_001352 | -3,78 | 8,5E-15 | Inhibited |
| PLTP | NM_182676 | -3,66 | 1,2E-178 | Inhibited |
| YPEL3 | NM_031477 | -3,65 | 5,9E-87 | Inhibited |
| DUSP2 | NM_004418 | -3,50 | 4,9E-26 | Inhibited |
| LRRC17 | NM_001031692 | -3,45 | 2,1E-149 | Inhibited |
| IGFBP5 | NM_000599 | -3,38 | 2,3E-68 | Inhibited |
| CAMK2B | NM_001220 | -3,26 | 1,2E-05 | Inhibited |
| GDF15 | NM_004864 | -3,13 | 1,9E-56 | Inhibited |
| SEMA6A | NM_020796 | -2,87 | 2,2E-09 | Inhibited |
| TP53INP1 | NM_001135733 | -2,86 | 8,7E-63 | Inhibited |
| PEG3 | NM_001146186 | -2,77 | 5,0E-05 | Inhibited |
| CLCA2 | NM_006536 | -2,72 | 2,0E-03 | Inhibited |
| SRGAP3 | NM_014850 | -2,71 | 2,1E-03 | Inhibited |
| CTSF | NM_003793 | -2,57 | 1,8E-39 | Inhibited |
| CMBL | NM_138809 | -2,56 | 5,3E-39 | Inhibited |
| NDRG2 | NM_016250 | -2,47 | 2,2E-04 | Inhibited |
| ENPP2 | NM_006209 | -2,38 | 9,4E-37 | Inhibited |
| CRACR2B | NM_173584 | -2,36 | 2,2E-21 | Inhibited |
| ADGRB3 | NM_001704 | -2,36 | 8,8E-03 | Inhibited |
| NYNRIN | NM_025081 | -2,33 | 6,2E-39 | Inhibited |
| PDGFRA | NM_006206 | -2,31 | 2,0E-74 | Inhibited |
| FBLN2 | NM_001165035 | -2,30 | 8,2E-93 | Inhibited |
| VDR | NM_000376 | -2,23 | 3,0E-28 | Inhibited |
| CASP1 | NM_033293 | -2,19 | 3,9E-03 | Inhibited |
| RGS16 | NM_002928 | -2,14 | 2,0E-04 | Inhibited |
| SYN1 | NM_006950 | -2,14 | 4,7E-06 | Inhibited |
| TRIM22 | NM_006074 | -2,12 | 5,3E-53 | Inhibited |
| MAPRE3 | NM_012326 | -2,10 | 5,4E-14 | Inhibited |
| SPATA18 | NM_145263 | -2,09 | 1,9E-09 | Inhibited |
| HMOX1 | NM_002133 | -2,09 | 1,8E-24 | Inhibited |
| EDA2R | NM_021783 | -2,09 | 2,4E-12 | Inhibited |
| WDR63 | NM_145172 | -2,05 | 2,3E-04 | Inhibited |
| ATG7 | NM_001144912 | -2,00 | 6,5E-55 | Inhibited |
| PADI2 | NM_007365 | -1,95 | 1,1E-02 | Inhibited |
| NEO1 | NM_002499 | -1,95 | 8,5E-26 | Inhibited |
| SOX2 | NM_003106 | -1,91 | 3,6E-02 | Inhibited |
| BBC3 | NM_001127242 | -1,91 | 3,0E-12 | Inhibited |
| BCAS3 | NM_001099432 | -1,90 | 3,0E-24 | Inhibited |
| SAT1 | NM_002970 | -1,90 | 3,2E-19 | Inhibited |
| CYP24A1 | NM_000782 | -1,89 | 3,9E-02 | Inhibited |
| CCNG2 | NM_004354 | -1,87 | 2,4E-33 | Inhibited |
| EPHX1 | NM_001136018 | -1,87 | 2,0E-29 | Inhibited |
| ABAT | NM_001127448 | -1,85 | 3,9E-04 | Inhibited |
| mir-199 | NR_029618 | -1,82 | 3,9E-02 | Inhibited |
| ZMAT3 | NM_152240 | -1,81 | 9,8E-21 | Inhibited |
| PHLDB3 | NM_198850 | -1,79 | 4,3E-07 | Inhibited |
| TCEA3 | NM_003196 | -1,78 | 3,1E-03 | Inhibited |
| PDGFRB | NM_002609 | -1,75 | 4,3E-52 | Inhibited |
| DDR1 | NM_013993 | -1,72 | 1,3E-45 | Inhibited |
| COQ8A | NM_020247 | -1,68 | 4,8E-17 | Inhibited |
| CLU | NM_203339 | -1,65 | 4,8E-36 | Inhibited |
| CTSK | NM_000396 | -1,65 | 8,4E-15 | Inhibited |
| ECH1 | NM_001398 | -1,63 | 2,6E-20 | Inhibited |
| FDXR | NM_004110 | -1,63 | 1,8E-11 | Inhibited |
| TRPV1 | NM_018727 | -1,62 | 5,1E-05 | Inhibited |
| HIC1 | NM_006497 | -1,60 | 1,1E-10 | Inhibited |
| NDRG1 | NM_001135242 | -1,59 | 2,5E-51 | Inhibited |
| KLLN | NM_001126049 | -1,59 | 9,3E-03 | Inhibited |
| ZEB1 | NR_024286 | -1,57 | 2,0E-19 | Inhibited |
| SNAI1 | NM_005985 | -1,47 | 2,5E-09 | Inhibited |
| PSAP | NM_002778 | -1,42 | 3,0E-28 | Inhibited |
| IRF9 | NM_006084 | -1,40 | 3,1E-16 | Inhibited |
| SESN1 | NM_014454 | -1,40 | 1,3E-08 | Inhibited |
| CTSD | NM_001909 | -1,34 | 2,1E-37 | Inhibited |
| BTG2 | NM_006763 | -1,34 | 1,6E-11 | Inhibited |
| PIDD1 | NM_145886 | -1,34 | 6,0E-12 | Inhibited |
| JUNB | NM_002229 | -1,32 | 3,2E-20 | Inhibited |
| CAT | NM_001752 | -1,30 | 2,9E-15 | Inhibited |
| BTG1 | NM_001731 | -1,29 | 2,9E-20 | Inhibited |
| PCCA | NM_000282 | -1,29 | 1,6E-06 | Inhibited |
| XPC | NM_004628 | -1,28 | 8,8E-16 | Inhibited |
| CDKN1A | NM_000389 | -1,28 | 1,8E-30 | Inhibited |
| CSF1 | NM_172210 | -1,27 | 2,1E-15 | Inhibited |
| F3 | NM_001993 | -1,27 | 3,1E-02 | Inhibited |
| SERPINA3 | NM_001085 | -1,26 | 6,8E-03 | Inhibited |
| PYCARD | NM_013258 | -1,25 | 5,4E-03 | Inhibited |
| CD82 | NM_001024844 | -1,24 | 1,2E-09 | Inhibited |
| IGFBP3 | NM_001013398 | -1,23 | 1,0E-18 | Inhibited |
| PHLDA1 | NM_007350 | -1,21 | 9,8E-32 | Inhibited |
| COL1A1 | NM_000088 | -1,20 | 8,9E-17 | Inhibited |
| PTCH1 | NM_001083604 | -1,19 | 1,8E-06 | Inhibited |
| GSN | NM_000177 | -1,19 | 6,5E-29 | Inhibited |
| MGMT | NM_002412 | -1,19 | 7,7E-07 | Inhibited |
| NRP1 | NM_003873 | -1,19 | 2,1E-23 | Inhibited |
| STEAP3 | NM_182915 | -1,17 | 4,9E-16 | Inhibited |
| EDIL3 | NM_005711 | -1,15 | 2,2E-11 | Inhibited |
| DDIT3 | NM_004083 | -1,13 | 1,6E-07 | Inhibited |
| ISCU | NM_014301 | -1,13 | 5,9E-13 | Inhibited |
| VWCE | NM_152718 | -1,12 | 7,0E-04 | Inhibited |
| CASP9 | NM_032996 | -1,10 | 1,5E-05 | Inhibited |
| COL6A2 | NM_001849 | -1,10 | 1,1E-22 | Inhibited |
| NINJ1 | NM_004148 | -1,09 | 2,7E-09 | Inhibited |
| KCNMA1 | NM_001161353 | -1,09 | 3,3E-12 | Inhibited |
| IFI16 | NM_005531 | -1,08 | 2,5E-16 | Inhibited |
| CES2 | NM_198061 | -1,08 | 7,3E-15 | Inhibited |
| CP | NM_000096 | -1,05 | 3,0E-02 | Inhibited |
| ROBO1 | NM_001145844 | -1,04 | 4,1E-18 | Inhibited |
| TSC2 | NM_001077183 | -1,04 | 4,8E-23 | Inhibited |
| TNFAIP8 | NM_014350 | -1,02 | 1,6E-09 | Inhibited |
| MX1 | NM_002462 | -1,01 | 2,7E-02 | Inhibited |
| RRM2B | NM_015713 | -1,01 | 5,9E-10 | Inhibited |
| ULK1 | NM_003565 | -1,00 | 3,5E-20 | Inhibited |
| C9orf116 | NM_144654 | -1,00 | 1,4E-02 | Inhibited |
| DDB2 | NM_000107 | -0,97 | 3,6E-08 | Inhibited |
| COL5A2 | NM_000393 | -0,97 | 2,4E-16 | Inhibited |
| THBS2 | NM_003247 | -0,97 | 4,3E-15 | Inhibited |
| CCNG1 | NM_199246 | -0,97 | 1,5E-12 | Inhibited |
| PTEN | NM_000314 | -0,94 | 7,2E-17 | Inhibited |
| IRF7 | NM_004029 | -0,94 | 5,8E-04 | Inhibited |
| LTBP1 | NM_001166266 | -0,93 | 3,2E-20 | Inhibited |
| TRAF1 | NM_005658 | -0,91 | 1,6E-02 | Inhibited |
| RB1CC1 | NM_014781 | -0,90 | 1,4E-14 | Inhibited |
| PML | NM_033238 | -0,87 | 1,3E-09 | Inhibited |
| ANK1 | NM_020480 | -0,87 | 8,1E-07 | Inhibited |
| ASTN2 | NM_198186 | -0,82 | 2,5E-02 | Inhibited |
| OMA1 | NM_145243 | -0,81 | 3,0E-03 | Inhibited |
| PERP | NM_022121 | -0,81 | 4,1E-08 | Inhibited |
| GSTM1 | NM_146421 | -0,81 | 2,9E-04 | Inhibited |
| TSPAN6 | NM_003270 | -0,81 | 1,9E-04 | Inhibited |
| IGF2 | NM_000612 | -0,81 | 2,1E-03 | Inhibited |
| ULK2 | NM_001142610 | -0,80 | 2,7E-04 | Inhibited |
| COL4A1 | NM_001845 | -0,79 | 3,2E-15 | Inhibited |
| PLXNB2 | NM_012401 | -0,78 | 5,0E-12 | Inhibited |
| LIF | NM_002309 | -0,77 | 1,4E-06 | Inhibited |
| FAS | NR_028034 | -0,77 | 8,0E-03 | Inhibited |
| ATG2B | NM_018036 | -0,77 | 1,0E-06 | Inhibited |
| CDKN1B | NM_004064 | -0,77 | 1,0E-08 | Inhibited |
| ZNF79 | NM_007135 | -0,76 | 1,5E-02 | Inhibited |
| ERCC5 | NM_000123 | -0,76 | 7,1E-07 | Inhibited |
| FLRT2 | NM_013231 | -0,75 | 6,8E-04 | Inhibited |
| PTPRM | NM_001105244 | -0,74 | 5,8E-09 | Inhibited |
| TP53I3 | NM_004881 | -0,74 | 4,0E-05 | Inhibited |
| PPIC | NM_000943 | -0,73 | 2,6E-04 | Inhibited |
| NOTCH1 | NM_017617 | -0,72 | 2,5E-09 | Inhibited |
| ACADVL | NM_001033859 | -0,71 | 2,8E-07 | Inhibited |
| TOM1 | NM_001135730 | -0,69 | 2,1E-04 | Inhibited |
| ANTXR1 | NM_032208 | -0,68 | 3,7E-05 | Inhibited |
| FOXO3 | NM_201559 | -0,68 | 4,5E-08 | Inhibited |
| PHLDA3 | NM_012396 | -0,67 | 4,8E-05 | Inhibited |
| HDAC5 | NM_001015053 | -0,67 | 1,3E-06 | Inhibited |
| ANXA4 | NM_001153 | -0,67 | 2,9E-04 | Inhibited |
| HADHB | NM_000183 | -0,66 | 1,4E-06 | Inhibited |
| RPS27L | NM_015920 | -0,66 | 2,8E-04 | Inhibited |
| COL18A1 | NM_130444 | -0,63 | 1,3E-05 | Inhibited |
| ATXN1 | NM_000332 | -0,62 | 3,7E-04 | Inhibited |
| FOS | NM_005252 | 0,61 | 7,9E-03 | Inhibited |
| DSTN | NM_001011546 | 0,62 | 1,1E-08 | Inhibited |
| TIMM44 | NM_006351 | 0,62 | 7,7E-05 | Inhibited |
| ZEB2 | NM_014795 | 0,63 | 1,1E-04 | Inhibited |
| IQCB1 | NM_001023571 | 0,65 | 8,0E-03 | Inhibited |
| FAM3C | NM_014888 | 0,67 | 4,9E-07 | Inhibited |
| PDRG1 | NM_030815 | 0,68 | 5,0E-03 | Inhibited |
| IL4R | NM_000418 | 0,68 | 1,6E-08 | Inhibited |
| TP53BP2 | NM_005426 | 0,69 | 7,3E-06 | Inhibited |
| ACSL3 | NM_004457 | 0,71 | 1,4E-06 | Inhibited |
| PARVA | NM_018222 | 0,72 | 4,2E-07 | Inhibited |
| MYOF | NM_133337 | 0,72 | 6,9E-11 | Inhibited |
| GRB2 | NM_002086 | 0,72 | 3,4E-07 | Inhibited |
| PTGS2 | NM_000963 | 0,73 | 1,6E-03 | Inhibited |
| SERPINH1 | NM_001235 | 0,73 | 1,2E-08 | Inhibited |
| PTP4A1 | NM_003463 | 0,73 | 4,9E-08 | Inhibited |
| IPO9 | NM_018085 | 0,74 | 1,9E-07 | Inhibited |
| MAP4 | NM_030885 | 0,76 | 7,4E-06 | Inhibited |
| YBX1 | NM_004559 | 0,76 | 2,4E-09 | Inhibited |
| HSPA8 | NM_153201 | 0,77 | 1,0E-16 | Inhibited |
| DNM1L | NM_012063 | 0,77 | 2,7E-10 | Inhibited |
| XPO1 | NM_003400 | 0,77 | 5,5E-14 | Inhibited |
| PGM3 | NM_015599 | 0,78 | 4,1E-08 | Inhibited |
| SLC2A1 | NM_006516 | 0,78 | 1,2E-05 | Inhibited |
| SPHK1 | NM_001142601 | 0,80 | 9,3E-04 | Inhibited |
| PSMD8 | NM_002812 | 0,80 | 5,1E-12 | Inhibited |
| SMC3 | NM_005445 | 0,81 | 1,9E-12 | Inhibited |
| RUNX2 | NM_001015051 | 0,81 | 8,3E-04 | Inhibited |
| ILK | NM_001014795 | 0,83 | 5,2E-11 | Inhibited |
| IPO7 | NM_006391 | 0,84 | 3,5E-11 | Inhibited |
| CDK7 | NM_001799 | 0,86 | 1,8E-04 | Inhibited |
| PSMD3 | NM_002809 | 0,86 | 1,2E-14 | Inhibited |
| HSP90AB1 | NM_007355 | 0,87 | 1,1E-11 | Inhibited |
| ACTN4 | NM_004924 | 0,87 | 2,1E-08 | Inhibited |
| AKT3 | NM_181690 | 0,88 | 2,9E-07 | Inhibited |
| TIMP3 | NM_000362 | 0,90 | 8,8E-11 | Inhibited |
| MIR17HG | NR_027350 | 0,92 | 8,4E-03 | Inhibited |
| TGFBR1 | NM_001130916 | 0,94 | 4,1E-11 | Inhibited |
| HSPA1A/HSPA1B | NM_005345 | 0,95 | 1,0E-09 | Inhibited |
| PSMD1 | NM_002807 | 0,95 | 5,1E-17 | Inhibited |
| POLA1 | NM_016937 | 0,96 | 4,9E-06 | Inhibited |
| SLC16A1 | NM_001166496 | 0,96 | 5,6E-14 | Inhibited |
| PPP2CA | NM_002715 | 1,00 | 7,7E-23 | Inhibited |
| P4HA1 | NM_000917 | 1,04 | 7,3E-19 | Inhibited |
| MAD1L1 | NM_001013837 | 1,04 | 1,2E-05 | Inhibited |
| HIF1A | NM_001530 | 1,05 | 9,6E-13 | Inhibited |
| POLD2 | NM_006230 | 1,06 | 3,0E-17 | Inhibited |
| ME2 | NM_002396 | 1,06 | 1,4E-11 | Inhibited |
| NUP153 | NM_005124 | 1,11 | 1,5E-15 | Inhibited |
| CD44 | NM_001001392 | 1,12 | 6,2E-26 | Inhibited |
| NECTIN3 | NM_015480 | 1,12 | 5,9E-11 | Inhibited |
| CHUK | NM_001278 | 1,13 | 7,1E-11 | Inhibited |
| AKAP12 | NM_144497 | 1,13 | 2,0E-21 | Inhibited |
| ARL6IP1 | NM_015161 | 1,15 | 2,2E-28 | Inhibited |
| TDG | NM_003211 | 1,20 | 2,7E-17 | Inhibited |
| PSME3 | NM_176863 | 1,21 | 1,0E-21 | Inhibited |
| HMGB1 | NM_002128 | 1,22 | 1,2E-30 | Inhibited |
| UNG | NM_080911 | 1,24 | 5,0E-11 | Inhibited |
| PARP2 | NM_001042618 | 1,24 | 1,3E-12 | Inhibited |
| AHSA1 | NM_012111 | 1,26 | 4,6E-18 | Inhibited |
| PSMD12 | NM_174871 | 1,27 | 1,0E-15 | Inhibited |
| HSPH1 | NM_006644 | 1,30 | 2,4E-29 | Inhibited |
| PSMD2 | NM_002808 | 1,31 | 3,3E-33 | Inhibited |
| TFDP1 | NM_007111 | 1,34 | 8,3E-25 | Inhibited |
| DSN1 | NM_001145316 | 1,34 | 1,7E-10 | Inhibited |
| H19 | NR_002196 | 1,35 | 8,4E-09 | Inhibited |
| KPNB1 | NM_002265 | 1,35 | 2,0E-42 | Inhibited |
| KRT18 | NM_199187 | 1,38 | 1,6E-15 | Inhibited |
| FUBP1 | NM_003902 | 1,39 | 8,4E-16 | Inhibited |
| CDC7 | NM_001134419 | 1,40 | 1,1E-11 | Inhibited |
| DEK | NM_003472 | 1,40 | 4,5E-33 | Inhibited |
| DHFR | NM_000791 | 1,41 | 9,0E-18 | Inhibited |
| BCL2 | NM_000633 | 1,43 | 3,6E-05 | Inhibited |
| TOP2A | NM_001067 | 1,51 | 7,7E-33 | Inhibited |
| LBR | NM_002296 | 1,52 | 2,0E-24 | Inhibited |
| CAP1 | NM_001105530 | 1,53 | 1,8E-37 | Inhibited |
| SOCS2 | NM_003877 | 1,53 | 1,1E-05 | Inhibited |
| UMPS | NM_000373 | 1,55 | 4,8E-15 | Inhibited |
| UGDH | NM_003359 | 1,58 | 9,5E-41 | Inhibited |
| DNMT1 | NM_001130823 | 1,58 | 2,0E-49 | Inhibited |
| SMC2 | NM_001042551 | 1,60 | 5,2E-31 | Inhibited |
| STIP1 | NM_006819 | 1,60 | 5,7E-47 | Inhibited |
| TMEM97 | NM_014573 | 1,60 | 2,5E-23 | Inhibited |
| NOS3 | NM_000603 | 1,61 | 1,8E-02 | Inhibited |
| RECQL4 | NM_004260 | 1,61 | 7,0E-24 | Inhibited |
| CHEK1 | NM_001274 | 1,62 | 1,9E-16 | Inhibited |
| MYC | NM_002467 | 1,63 | 2,7E-38 | Inhibited |
| SFRP1 | NM_003012 | 1,63 | 2,4E-10 | Inhibited |
| PRIM1 | NM_000946 | 1,64 | 4,3E-08 | Inhibited |
| ACLY | NM_001096 | 1,67 | 2,3E-52 | Inhibited |
| HSPD1 | NM_199440 | 1,70 | 3,7E-66 | Inhibited |
| PCLAF | NM_014736 | 1,70 | 4,7E-20 | Inhibited |
| EZH2 | NM_152998 | 1,71 | 2,7E-22 | Inhibited |
| SMC4 | NM_005496 | 1,75 | 9,6E-50 | Inhibited |
| POLD1 | NM_002691 | 1,75 | 1,4E-31 | Inhibited |
| FKBP4 | NM_002014 | 1,76 | 3,6E-58 | Inhibited |
| WNT5B | NM_032642 | 1,76 | 1,4E-21 | Inhibited |
| ACTN1 | NM_001102 | 1,79 | 1,4E-51 | Inhibited |
| SRSF3 | NM_003017 | 1,80 | 4,1E-36 | Inhibited |
| CCL2 | NM_002982 | 1,83 | 7,8E-08 | Inhibited |
| STMN1 | NM_203401 | 1,84 | 7,2E-30 | Inhibited |
| CDT1 | NM_030928 | 1,85 | 8,2E-33 | Inhibited |
| SREBF1 | NM_004176 | 1,88 | 2,0E-33 | Inhibited |
| CCND3 | NM_001760 | 1,88 | 5,4E-44 | Inhibited |
| PDK1 | NM_002610 | 1,90 | 2,1E-30 | Inhibited |
| HSP90AA1 | NM_005348 | 1,90 | 6,7E-51 | Inhibited |
| PFKFB3 | NM_004566 | 1,92 | 1,3E-26 | Inhibited |
| SELP | NM_003005 | 1,95 | 3,3E-02 | Inhibited |
| MCM7 | NM_182776 | 1,96 | 1,4E-56 | Inhibited |
| LDHA | NM_001165415 | 1,96 | 5,4E-64 | Inhibited |
| PLOD2 | NM_182943 | 1,98 | 1,4E-74 | Inhibited |
| PLAUR | NM_001005376 | 1,99 | 4,4E-40 | Inhibited |
| TTK | NM_003318 | 2,00 | 2,9E-32 | Inhibited |
| MCM6 | NM_005915 | 2,01 | 1,9E-47 | Inhibited |
| CENPF | NM_016343 | 2,03 | 6,5E-57 | Inhibited |
| CDC25C | NM_001790 | 2,03 | 6,1E-16 | Inhibited |
| CKS1B | NM_001826 | 2,06 | 4,4E-30 | Inhibited |
| PODXL | NM_001018111 | 2,09 | 1,5E-30 | Inhibited |
| ESPL1 | NM_012291 | 2,09 | 5,2E-37 | Inhibited |
| MCM2 | NM_004526 | 2,12 | 8,5E-37 | Inhibited |
| BRCA1 | NM_007297 | 2,12 | 6,5E-28 | Inhibited |
| RAD54B | NM_012415 | 2,13 | 1,4E-14 | Inhibited |
| H2AFX | NM_002105 | 2,15 | 1,8E-89 | Inhibited |
| CDK1 | NM_033379 | 2,16 | 7,9E-46 | Inhibited |
| NDC80 | NM_006101 | 2,17 | 1,3E-27 | Inhibited |
| PFKP | NM_002627 | 2,22 | 6,6E-73 | Inhibited |
| MCM4 | NM_182746 | 2,24 | 1,0E-71 | Inhibited |
| WDHD1 | NM_001008396 | 2,24 | 4,8E-30 | Inhibited |
| CDKN3 | NM_005192 | 2,25 | 3,0E-24 | Inhibited |
| MCM3 | NM_002388 | 2,27 | 8,6E-33 | Inhibited |
| BNIP3 | NM_004052 | 2,28 | 1,5E-81 | Inhibited |
| CCNE2 | NM_057749 | 2,29 | 7,6E-15 | Inhibited |
| ANXA3 | NM_005139 | 2,30 | 2,0E-05 | Inhibited |
| SLC19A1 | NM_194255 | 2,31 | 7,3E-51 | Inhibited |
| HMMR | NM_001142557 | 2,33 | 3,6E-27 | Inhibited |
| NCAPG | NM_022346 | 2,34 | 9,2E-55 | Inhibited |
| HK2 | NM_000189 | 2,37 | 3,4E-79 | Inhibited |
| MKI67 | NM_001145966 | 2,38 | 6,9E-35 | Inhibited |
| KPNA2 | NM_002266 | 2,39 | 1,2E-100 | Inhibited |
| LOX | NM_002317 | 2,43 | 9,2E-35 | Inhibited |
| NEK2 | NM_002497 | 2,43 | 1,3E-29 | Inhibited |
| MCM5 | NM_006739 | 2,46 | 3,6E-75 | Inhibited |
| POLE2 | NM_002692 | 2,49 | 1,5E-09 | Inhibited |
| DBF4 | NM_006716 | 2,50 | 6,1E-37 | Inhibited |
| CCNB2 | NM_004701 | 2,51 | 6,9E-50 | Inhibited |
| TPX2 | NM_012112 | 2,57 | 1,2E-62 | Inhibited |
| FEN1 | NM_004111 | 2,58 | 5,5E-51 | Inhibited |
| UBE2C | NM_181800 | 2,65 | 1,3E-46 | Inhibited |
| FOXM1 | NM_021953 | 2,69 | 1,6E-82 | Inhibited |
| PRC1 | NM_003981 | 2,69 | 1,5E-114 | Inhibited |
| MET | NM_001127500 | 2,70 | 5,2E-37 | Inhibited |
| VRK1 | NM_003384 | 2,77 | 1,2E-26 | Inhibited |
| ID3 | NM_002167 | 2,78 | 2,6E-101 | Inhibited |
| MAD2L1 | NM_002358 | 2,80 | 5,2E-49 | Inhibited |
| CCNB1 | NM_031966 | 2,80 | 1,9E-78 | Inhibited |
| BRCA2 | NM_000059 | 2,80 | 8,2E-39 | Inhibited |
| AURKB | NM_004217 | 2,80 | 1,2E-60 | Inhibited |
| RFC3 | NM_002915 | 2,83 | 2,1E-28 | Inhibited |
| CDC20 | NM_001255 | 2,83 | 5,1E-92 | Inhibited |
| BIRC5 | NM_001012270 | 2,83 | 3,8E-60 | Inhibited |
| MYBL2 | NM_002466 | 2,83 | 2,4E-99 | Inhibited |
| BUB1 | NM_004336 | 2,84 | 3,8E-74 | Inhibited |
| MMP1 | NM_002421 | 2,85 | 2,5E-47 | Inhibited |
| KIF23 | NM_004856 | 2,90 | 9,4E-94 | Inhibited |
| E2F1 | NM_005225 | 2,93 | 3,2E-60 | Inhibited |
| PLK1 | NM_005030 | 2,99 | 6,8E-91 | Inhibited |
| AURKA | NM_198436 | 3,00 | 8,4E-83 | Inhibited |
| BUB1B | NM_001211 | 3,03 | 2,3E-83 | Inhibited |
| CDC25A | NM_201567 | 3,07 | 8,0E-38 | Inhibited |
| PBK | NM_018492 | 3,08 | 8,3E-55 | Inhibited |
| CCNA2 | NM_001237 | 3,10 | 8,0E-114 | Inhibited |
| RRM2 | NM_001165931 | 3,12 | 1,1E-116 | Inhibited |
| MELK | NM_014791 | 3,16 | 6,1E-56 | Inhibited |
| PDGFB | NM_002608 | 3,30 | 1,2E-21 | Inhibited |
| CDC6 | NM_001254 | 3,36 | 1,6E-67 | Inhibited |
| CEP55 | NM_001127182 | 3,38 | 1,0E-117 | Inhibited |
| RAD51 | NM_002875 | 3,53 | 8,4E-39 | Inhibited |
| GBX2 | NM_001485 | 4,04 | 3,9E-25 | Inhibited |
| IL1B | NM_000576 | 4,14 | 7,3E-11 | Inhibited |
| IL6 | NM_000600 | 4,19 | 3,5E-21 | Inhibited |

**Supplemental Table 5:** Differential expression of E2F1- and/or E2F3-dependent target genes in co-cultured pericytes

| Gene ID | Refseq ID | Log2FC | P Adjusted | E2F1 | E2F3 | E2F Prediction |
| --- | --- | --- | --- | --- | --- | --- |
| AKR1C1/AKR1C2 | NM_001354 | -4,54 | 6,22E-39 |  | x | Activated |
| AKR1C3 | NM_003739 | -4,08 | 1,72E-27 |  | x | Activated |
| PDK4 | NM_002612 | -3,96 | 1,56E-17 | x |  | Activated |
| VCAM1 | NM_001078 | -3,03 | 2,99E-08 | x |  | Activated |
| PPARGC1A | NM_013261 | -2,12 | 1,72E-02 | x |  | Activated |
| FAM102A | NM_203305 | -1,97 | 1,30E-40 |  | x | Activated |
| NEAT1 | NR_028272 | -1,84 | 2,12E-16 |  | x | Activated |
| VEGFA | NM_001025367 | -1,83 | 3,99E-56 | x |  | Activated |
| ECHDC2 | NM_018281 | -1,67 | 3,23E-05 |  | x | Activated |
| LAMA4 | NM_002290 | -1,62 | 3,95E-32 |  | x | Activated |
| MALAT1 | NR_002819 | -1,24 | 4,97E-17 |  | x | Activated |
| DDIT3 | NM_004083 | -1,13 | 1,59E-07 | x |  | Activated |
| OGT | NM_181672 | -1,12 | 1,74E-22 | x |  | Activated |
| AKNA | NM_030767 | -1,02 | 1,03E-08 |  | x | Activated |
| CDKN2A | NM_058197 | -0,93 | 2,02E-06 |  | x | Activated |
| AR | NM_000044 | -0,76 | 6,02E-03 | x |  | Activated |
| SMG6 | NM_017575 | -0,75 | 3,40E-09 |  | x | Activated |
| XPO4 | NM_022459 | 0,62 | 4,38E-04 |  | x | Activated |
| ZNF672 | NM_024836 | 0,62 | 1,12E-04 | x |  | Activated |
| RAB1A | NM_004161 | 0,64 | 7,64E-09 | x |  | Activated |
| E2F4 | NM_001950 | 0,66 | 7,06E-08 | x |  | Activated |
| SIVA1 | NM_021709 | 0,67 | 1,84E-02 | x |  | Activated |
| EI24 | NM_004879 | 0,69 | 3,91E-10 | x |  | Activated |
| TP53BP2 | NM_005426 | 0,69 | 7,27E-06 | x |  | Activated |
| NPAT | NM_002519 | 0,71 | 4,49E-03 | x | x | Activated |
| MAP3K5 | NM_005923 | 0,73 | 2,59E-05 | x |  | Activated |
| CKB | NM_001823 | 0,77 | 1,44E-03 | x |  | Activated |
| NUCKS1 | NM_022731 | 0,79 | 8,19E-15 |  | x | Activated |
| MLH1 | NM_000249 | 0,80 | 2,80E-07 | x | x | Activated |
| AKT1 | NM_001014431 | 0,83 | 2,79E-16 | x |  | Activated |
| KRAS | NM_033360 | 0,83 | 2,26E-07 | x | x | Activated |
| PIN1 | NM_006221 | 0,83 | 2,55E-09 | x | x | Activated |
| CRIP2 | NM_001312 | 0,83 | 1,43E-06 | x |  | Activated |
| ILK | NM_001014795 | 0,83 | 5,21E-11 | x |  | Activated |
| ADIPOR2 | NM_024551 | 0,84 | 3,98E-11 | x |  | Activated |
| TRAF3 | NM_003300 | 0,85 | 1,05E-04 | x |  | Activated |
| TIMP3 | NM_000362 | 0,90 | 8,82E-11 |  | x | Activated |
| RPA2 | NM_002946 | 0,91 | 1,71E-05 | x |  | Activated |
| NFKBIB | NM_001001716 | 0,93 | 5,83E-06 | x |  | Activated |
| RSL1D1 | NM_015659 | 0,93 | 1,51E-17 | x |  | Activated |
| POLA1 | NM_016937 | 0,96 | 4,87E-06 | x |  | Activated |
| BAK1 | NM_001188 | 0,96 | 2,07E-08 | x |  | Activated |
| PTMA | NM_002823 | 0,97 | 3,93E-11 |  | x | Activated |
| CDKN2C | NM_001262 | 0,98 | 7,34E-07 | x |  | Activated |
| RALGPS2 | NM_152663 | 1,03 | 4,57E-08 |  | x | Activated |
| MMP16 | NM_005941 | 1,04 | 2,95E-17 | x |  | Activated |
| BID | NM_197966 | 1,06 | 2,62E-08 | x |  | Activated |
| VCP | NM_007126 | 1,10 | 2,35E-13 | x |  | Activated |
| CAV2 | NM_001233 | 1,12 | 4,78E-20 |  | x | Activated |
| PDCD5 | NM_004708 | 1,12 | 1,90E-08 | x |  | Activated |
| MTBP | NM_022045 | 1,14 | 5,71E-04 | x |  | Activated |
| HSP90B1 | NM_003299 | 1,14 | 1,23E-21 | x |  | Activated |
| PHB | NM_002634 | 1,16 | 3,41E-17 | x |  | Activated |
| RRP1B | NM_015056 | 1,18 | 5,39E-22 | x |  | Activated |
| PLPPR4 | NM_001166252 | 1,21 | 4,13E-11 | x |  | Activated |
| FGF2 | NM_002006 | 1,23 | 1,11E-11 | x | x | Activated |
| E2F3 | NM_001949 | 1,23 | 5,76E-15 |  | x | Activated |
| UNG | NM_080911 | 1,24 | 5,03E-11 | x |  | Activated |
| FGFR3 | NM_022965 | 1,26 | 8,55E-06 | x |  | Activated |
| PSMD2 | NM_002808 | 1,31 | 3,34E-33 | x |  | Activated |
| H19 | NR_002196 | 1,35 | 8,38E-09 | x |  | Activated |
| DHFR | NM_000791 | 1,41 | 9,03E-18 | x |  | Activated |
| BCL2 | NM_000633 | 1,43 | 3,63E-05 | x |  | Activated |
| TOP2A | NM_001067 | 1,51 | 7,65E-33 | x |  | Activated |
| LBR | NM_002296 | 1,52 | 1,96E-24 |  | x | Activated |
| TOPBP1 | NM_007027 | 1,55 | 2,00E-31 | x | x | Activated |
| PCNA | NM_002592 | 1,56 | 3,53E-21 |  | x | Activated |
| CHEK1 | NM_001274 | 1,62 | 1,90E-16 | x |  | Activated |
| MYC | NM_002467 | 1,63 | 2,67E-38 | x |  | Activated |
| UCHL5 | NM_015984 | 1,66 | 8,98E-17 | x |  | Activated |
| E2F2 | NM_004091 | 1,67 | 5,05E-05 | x | x | Activated |
| PRIM2 | NM_000947 | 1,68 | 1,31E-13 | x |  | Activated |
| HSPD1 | NM_199440 | 1,70 | 3,73E-66 |  | x | Activated |
| PCLAF | NM_014736 | 1,70 | 4,68E-20 |  | x | Activated |
| EZH2 | NM_152998 | 1,71 | 2,73E-22 | x |  | Activated |
| POLD1 | NM_002691 | 1,75 | 1,41E-31 | x |  | Activated |
| DUSP1 | NM_004417 | 1,78 | 1,92E-53 | x |  | Activated |
| CDCA4 | NM_145701 | 1,81 | 2,53E-25 | x | x | Activated |
| CCNE1 | NM_001238 | 1,85 | 8,29E-10 | x | x | Activated |
| CCND3 | NM_001760 | 1,88 | 5,39E-44 | x | x | Activated |
| HELLS | NM_018063 | 1,92 | 7,36E-13 | x |  | Activated |
| CSE1L | NM_001316 | 1,94 | 4,89E-52 | x |  | Activated |
| HMGB2 | NM_001130689 | 1,96 | 1,60E-48 |  | x | Activated |
| MCM7 | NM_182776 | 1,96 | 1,39E-56 | x |  | Activated |
| MCM6 | NM_005915 | 2,01 | 1,91E-47 | x |  | Activated |
| RFC4 | NM_002916 | 2,04 | 2,62E-20 | x |  | Activated |
| THBS1 | NM_003246 | 2,05 | 1,14E-30 | x | x | Activated |
| ENO2 | NM_001975 | 2,07 | 8,80E-41 | x |  | Activated |
| FBXO5 | NM_012177 | 2,09 | 7,65E-37 | x | x | Activated |
| BRCA1 | NM_007297 | 2,12 | 6,53E-28 | x |  | Activated |
| MCM2 | NM_004526 | 2,12 | 8,50E-37 | x |  | Activated |
| CDK1 | NM_033379 | 2,16 | 7,95E-46 | x | x | Activated |
| RBL1 | NM_183404 | 2,16 | 1,46E-21 | x |  | Activated |
| RRM1 | NM_001033 | 2,17 | 2,25E-70 | x |  | Activated |
| CDK2 | NM_052827 | 2,17 | 1,55E-52 | x | x | Activated |
| MCM4 | NM_182746 | 2,24 | 9,98E-72 | x |  | Activated |
| MCM3 | NM_002388 | 2,27 | 8,60E-33 | x |  | Activated |
| BNIP3 | NM_004052 | 2,28 | 1,47E-81 | x |  | Activated |
| CCNE2 | NM_057749 | 2,29 | 7,65E-15 | x |  | Activated |
| PTTG1 | NM_004219 | 2,35 | 1,45E-29 |  | x | Activated |
| HIST1H4B | NM_003544 | 2,38 | 7,96E-03 |  | x | Activated |
| POLA2 | NM_002689 | 2,38 | 7,38E-40 | x |  | Activated |
| GMNN | NM_015895 | 2,40 | 3,01E-30 | x |  | Activated |
| MCM5 | NM_006739 | 2,46 | 3,59E-75 | x |  | Activated |
| DBF4 | NM_006716 | 2,50 | 6,14E-37 | x |  | Activated |
| CCND1 | NM_053056 | 2,53 | 4,67E-123 |  | x | Activated |
| TMPO | NM_003276 | 2,55 | 2,99E-80 |  | x | Activated |
| TPX2 | NM_012112 | 2,57 | 1,25E-62 |  | x | Activated |
| TYMS | NM_001071 | 2,57 | 8,67E-87 | x |  | Activated |
| CDCA3 | NM_031299 | 2,58 | 1,64E-52 |  | x | Activated |
| FEN1 | NM_004111 | 2,58 | 5,47E-51 | x |  | Activated |
| TK1 | NM_003258 | 2,59 | 1,59E-58 | x |  | Activated |
| NCAPG2 | NM_017760 | 2,63 | 1,70E-76 |  | x | Activated |
| UBE2C | NM_181800 | 2,65 | 1,28E-46 |  | x | Activated |
| ECT2 | NM_018098 | 2,66 | 3,56E-80 | x |  | Activated |
| ORC1 | NM_004153 | 2,75 | 1,31E-27 | x |  | Activated |
| MAD2L1 | NM_002358 | 2,80 | 5,24E-49 |  | x | Activated |
| RFC3 | NM_002915 | 2,83 | 2,10E-28 | x |  | Activated |
| BIRC5 | NM_001012270 | 2,83 | 3,76E-60 | x | x | Activated |
| MYBL2 | NM_002466 | 2,83 | 2,40E-99 | x | x | Activated |
| MMP1 | NM_002421 | 2,85 | 2,48E-47 | x |  | Activated |
| CXCL1 | NM_001511 | 2,89 | 6,21E-12 |  | x | Activated |
| E2F1 | NM_005225 | 2,93 | 3,15E-60 | x | x | Activated |
| PLK1 | NM_005030 | 2,99 | 6,81E-91 |  | x | Activated |
| AURKA | NM_198436 | 3,00 | 8,40E-83 | x |  | Activated |
| KRT80 | NM_001081492 | 3,09 | 1,57E-50 |  | x | Activated |
| CCNA2 | NM_001237 | 3,10 | 8,01E-114 | x | x | Activated |
| RRM2 | NM_001165931 | 3,12 | 1,11E-116 | x | x | Activated |
| CDC45 | NM_003504 | 3,27 | 6,69E-42 | x | x | Activated |
| CDC6 | NM_001254 | 3,36 | 1,63E-67 | x | x | Activated |
| CRYAB | NM_001885 | 3,36 | 6,59E-94 | x |  | Activated |
| INHBA | NM_002192 | 3,37 | 1,41E-158 |  | x | Activated |
| KDR | NM_002253 | 3,44 | 2,33E-28 | x |  | Activated |
| FST | NM_006350 | 3,45 | 2,79E-52 |  | x | Activated |
| RAD51 | NM_002875 | 3,53 | 8,43E-39 | x | x | Activated |
| EDN1 | NM_001955 | 4,06 | 1,03E-13 |  | x | Activated |
| MCM10 | NM_182751 | 4,67 | 5,39E-89 | x | x | Activated |
| FLT1 | NM_001159920 | 4,69 | 6,34E-35 | x |  | Activated |
| SERPINE1 | NM_000602 | 4,86 | 0,00E+00 | x |  | Activated |

**Supplemental Table 6:** Differentially expressed genes involved in axon guiding in co-cultured pericytes

| Gene ID | Refseq ID | Log2FC | P Adjusted |
| --- | --- | --- | --- |
| EPHA7 | NM_004440 | -5,52 | 7,28E-151 |
| EPHA3 | NM_005233 | -4,72 | 2,63E-26 |
| NTNG2 | NM_032536 | -3,81 | 7,75E-13 |
| MMP11 | NM_005940 | -3,43 | 1,54E-75 |
| ADAMTS13 | NM_139026 | -3,31 | 2,12E-22 |
| NTN5 | NM_145807 | -2,99 | 4,00E-05 |
| SEMA3D | NM_152754 | -2,96 | 2,35E-23 |
| SEMA6A | NM_020796 | -2,87 | 2,15E-09 |
| NTRK3 | NM_001007156 | -2,75 | 1,86E-03 |
| SRGAP3 | NM_014850 | -2,71 | 2,10E-03 |
| GLI1 | NM_005269 | -2,68 | 3,88E-19 |
| EFNB3 | NM_001406 | -2,64 | 7,92E-30 |
| NTN1 | NM_004822 | -2,59 | 4,18E-24 |
| PLCL1 | NM_006226 | -2,54 | 3,39E-09 |
| ADAMTS8 | NM_007037 | -2,51 | 2,19E-07 |
| PTCH2 | NM_001166292 | -2,46 | 1,70E-05 |
| TLR9 | NM_017442 | -2,39 | 4,08E-03 |
| KEL | NM_000420 | -2,31 | 1,05E-02 |
| UNC5C | NM_003728 | -2,28 | 2,79E-03 |
| NFATC4 | NM_001136022 | -2,24 | 9,65E-51 |
| UNC5B | NM_170744 | -2,18 | 2,23E-26 |
| MYL5 | NM_002477 | -2,10 | 8,84E-10 |
| NTF4 | NM_006179 | -2,07 | 2,05E-02 |
| LRRC4C | NM_020929 | -2,02 | 2,64E-02 |
| ITGA2 | NM_002203 | -2,02 | 2,59E-15 |
| PLCH2 | NM_014638 | -2,00 | 6,00E-31 |
| GLIS1 | NM_147193 | -1,97 | 1,45E-24 |
| SEMA3E | NM_012431 | -1,96 | 4,07E-08 |
| PLCD4 | NM_032726 | -1,95 | 1,52E-11 |
| PLXNC1 | NM_005761 | -1,89 | 1,35E-02 |
| NGFR | NM_002507 | -1,89 | 1,22E-03 |
| VEGFA | NM_001025367 | -1,83 | 3,47E-55 |
| WNT11 | NM_004626 | -1,80 | 3,35E-02 |
| EPHB3 | NM_004443 | -1,79 | 3,34E-04 |
| MMP2 | NM_004530 | -1,69 | 1,30E-30 |
| ADAM8 | NM_001164490 | -1,68 | 3,27E-03 |
| PLCE1 | NM_001165979 | -1,67 | 1,25E-21 |
| RHOD | NM_014578 | -1,67 | 1,32E-05 |
| WNT16 | NM_057168 | -1,65 | 8,51E-04 |
| L1CAM | NM_001143963 | -1,64 | 7,74E-06 |
| SEMA4A | NM_022367 | -1,63 | 3,65E-03 |
| SEMA4G | NM_017893 | -1,50 | 3,85E-09 |
| GAB1 | NM_207123 | -1,50 | 6,76E-13 |
| GNG7 | NM_052847 | -1,43 | 9,50E-03 |
| ROBO3 | NM_022370 | -1,37 | 6,22E-14 |
| GNG11 | NM_004126 | -1,34 | 3,94E-17 |
| ROBO2 | NM_001128929 | -1,32 | 6,10E-12 |
| RASSF5 | NM_182664 | -1,29 | 3,93E-03 |
| SEMA6C | NM_030913 | -1,27 | 3,53E-08 |
| PIK3R2 | NM_005027 | -1,27 | 8,98E-24 |
| STK36 | NM_015690 | -1,26 | 1,22E-17 |
| NGEF | NM_001114090 | -1,24 | 1,02E-03 |
| ARHGEF6 | NM_004840 | -1,24 | 4,23E-15 |
| PLCD3 | NM_133373 | -1,23 | 1,56E-17 |
| FZD1 | NM_003505 | -1,23 | 3,71E-18 |
| PLCD1 | NR_024071 | -1,21 | 2,80E-07 |
| FZD8 | NM_031866 | -1,19 | 3,25E-08 |
| PTCH1 | NM_001083604 | -1,19 | 1,82E-06 |
| NRP1 | NM_003873 | -1,19 | 2,08E-23 |
| SMO | NM_005631 | -1,16 | 1,14E-13 |
| SEMA4B | NM_198925 | -1,15 | 7,08E-09 |
| IRS2 | NM_003749 | -1,13 | 4,29E-13 |
| GLIS2 | NM_032575 | -1,12 | 1,93E-13 |
| SUFU | NM_016169 | -1,11 | 1,06E-11 |
| EFNA5 | NM_001962 | -1,09 | 1,08E-06 |
| ATM | NM_138292 | -1,07 | 1,55E-09 |
| GLI3 | NM_000168 | -1,06 | 5,35E-08 |
| PLXNB1 | NM_001130082 | -1,06 | 9,51E-11 |
| ROBO1 | NM_001145844 | -1,04 | 2,19E-17 |
| PPP3CA | NM_001130692 | -1,02 | 9,65E-19 |
| PIK3R3 | NM_003629 | -0,99 | 2,98E-08 |
| BMP8A | NM_181809 | -0,95 | 4,96E-02 |
| FZD2 | NM_001466 | -0,92 | 1,19E-06 |
| EFNB1 | NM_004429 | -0,91 | 9,46E-09 |
| RAC2 | NM_002872 | -0,91 | 9,61E-08 |
| SEMA3B | NM_001005914 | -0,88 | 6,78E-03 |
| NFAT5 | NM_006599 | -0,88 | 1,68E-04 |
| CDK5 | NM_004935 | -0,84 | 1,11E-04 |
| GNG2 | NM_053064 | -0,81 | 1,13E-02 |
| ADAM17 | NM_003183 | -0,80 | 2,47E-08 |
| PLXNB2 | NM_012401 | -0,78 | 4,96E-12 |
| MYSM1 | NM_001085487 | -0,77 | 6,18E-04 |
| ERAP2 | NM_001130140 | -0,74 | 2,75E-06 |
| PIK3R1 | NM_181504 | -0,69 | 4,06E-05 |
| VEGFB | NM_003377 | -0,69 | 5,91E-06 |
| EFNA4 | NM_182689 | -0,68 | 2,39E-02 |
| PRKAR1B | NM_001164762 | -0,68 | 2,76E-03 |
| GNB5 | NM_016194 | -0,66 | 4,69E-04 |
| ARHGEF12 | NM_015313 | -0,64 | 2,83E-06 |
| WASL | NM_003941 | -0,64 | 9,56E-04 |
| MKNK1 | NR_024176 | -0,63 | 2,28E-03 |
| ADAMTS7 | NM_014272 | -0,63 | 7,04E-06 |
| BAIAP2 | NM_006340 | -0,63 | 4,71E-04 |
| MAPK3 | NM_001040056 | -0,62 | 8,72E-05 |
| EFNB2 | NM_004093 | 0,60 | 1,14E-06 |
| CRK | NM_016823 | 0,62 | 6,23E-07 |
| ADAM9 | NR_027638 | 0,63 | 5,14E-08 |
| ADAM19 | NM_033274 | 0,64 | 1,07E-07 |
| RAP1B | NM_015646 | 0,64 | 6,21E-08 |
| AFG3L2 | NM_006796 | 0,65 | 8,30E-06 |
| RHOA | NM_001664 | 0,66 | 8,97E-11 |
| SLIT2 | NM_004787 | 0,72 | 1,84E-05 |
| GRB2 | NM_002086 | 0,72 | 3,43E-07 |
| CDC42 | NM_044472 | 0,72 | 2,43E-06 |
| GNG5 | NM_005274 | 0,74 | 2,68E-08 |
| PDIA3 | NM_005313 | 0,79 | 2,09E-14 |
| GNAI3 | NM_006496 | 0,80 | 1,83E-11 |
| PAK4 | NM_001014835 | 0,81 | 1,24E-07 |
| AKT1 | NM_001014431 | 0,83 | 3,02E-16 |
| KRAS | NM_033360 | 0,83 | 2,26E-07 |
| ARPC1A | NM_006409 | 0,84 | 2,98E-13 |
| AKT3 | NM_181690 | 0,88 | 2,99E-07 |
| MYL12B | NM_033546 | 0,88 | 6,55E-12 |
| BCAR1 | NM_014567 | 0,88 | 1,32E-13 |
| PDGFC | NM_016205 | 0,89 | 3,22E-07 |
| MYL9 | NM_006097 | 0,89 | 2,10E-06 |
| RAC3 | NM_005052 | 0,89 | 3,52E-05 |
| SEMA7A | NM_001146030 | 0,90 | 1,99E-07 |
| KLC1 | NM_005552 | 0,90 | 1,37E-14 |
| SEMA3F | NM_004186 | 0,91 | 2,77E-07 |
| ARPC4 | NM_001024960 | 0,92 | 1,11E-11 |
| FZD5 | NM_003468 | 0,93 | 3,77E-02 |
| ADAMTS2 | NM_014244 | 0,93 | 1,66E-11 |
| ADAM23 | NM_003812 | 0,93 | 7,92E-04 |
| BRCC3 | NM_024332 | 0,95 | 1,40E-05 |
| PTPN11 | NM_002834 | 0,97 | 1,57E-21 |
| PLCB4 | NM_000933 | 0,98 | 1,14E-05 |
| SEMA6D | NM_024966 | 0,98 | 1,38E-04 |
| MYL6 | NM_079423 | 0,98 | 7,43E-06 |
| PPP3CC | NM_005605 | 1,00 | 1,10E-09 |
| RTN4 | NM_207521 | 1,01 | 6,72E-14 |
| ITGA3 | NM_002204 | 1,03 | 8,84E-20 |
| ARPC5L | NM_030978 | 1,03 | 3,12E-07 |
| GNB1 | NM_002074 | 1,08 | 5,43E-33 |
| CFL2 | NR_028132 | 1,10 | 2,87E-22 |
| GNA11 | NM_002067 | 1,12 | 9,47E-18 |
| RGS3 | NM_017790 | 1,13 | 1,84E-02 |
| ARPC2 | NM_005731 | 1,14 | 1,34E-24 |
| GNB4 | NM_021629 | 1,15 | 4,13E-26 |
| ACTR2 | NM_005722 | 1,17 | 2,73E-36 |
| ADAMTS4 | NM_005099 | 1,18 | 9,47E-03 |
| PFN1 | NM_005022 | 1,19 | 3,81E-16 |
| ITGA5 | NM_002205 | 1,21 | 1,84E-28 |
| BMP2 | NM_001200 | 1,26 | 2,56E-04 |
| FGFR3 | NM_022965 | 1,26 | 8,55E-06 |
| GNB1L | NM_053004 | 1,27 | 3,60E-05 |
| MYL12A | NM_006471 | 1,27 | 5,24E-14 |
| ADAMTS5 | NM_007038 | 1,27 | 5,15E-10 |
| CFL1 | NM_005507 | 1,28 | 1,18E-25 |
| PRKCH | NM_006255 | 1,28 | 4,17E-07 |
| HRAS | NM_001130442 | 1,32 | 1,38E-09 |
| NRAS | NM_002524 | 1,35 | 9,88E-28 |
| NRP2 | NM_018534 | 1,36 | 1,85E-07 |
| GLI2 | NM_005270 | 1,37 | 1,01E-12 |
| NTN4 | NM_021229 | 1,37 | 1,64E-36 |
| MAP2K1 | NM_002755 | 1,42 | 8,80E-26 |
| PLCB3 | NM_000932 | 1,44 | 1,71E-21 |
| EIF4E | NM_001968 | 1,45 | 3,78E-21 |
| LINGO1 | NM_032808 | 1,46 | 1,27E-02 |
| TUBB3 | NM_006086 | 1,46 | 2,24E-40 |
| EFNA1 | NM_182685 | 1,47 | 2,52E-03 |
| VASP | NM_003370 | 1,48 | 1,46E-18 |
| BMP4 | NM_130851 | 1,51 | 1,45E-05 |
| ADAMTS6 | NM_197941 | 1,51 | 5,31E-09 |
| PGF | NM_002632 | 1,54 | 1,87E-11 |
| BMP6 | NM_001718 | 1,56 | 8,95E-09 |
| TUBG1 | NM_001070 | 1,57 | 2,08E-23 |
| ACTR3 | NM_005721 | 1,64 | 1,09E-63 |
| ITGA4 | NM_000885 | 1,64 | 2,09E-27 |
| ARPC5 | NM_005717 | 1,67 | 5,43E-33 |
| PSMD14 | NM_005805 | 1,72 | 7,67E-26 |
| SEMA3G | NM_020163 | 1,73 | 3,28E-02 |
| SEMA6B | NM_032108 | 1,74 | 1,94E-08 |
| WNT5B | NM_032642 | 1,76 | 1,36E-21 |
| RRAS2 | NM_012250 | 1,81 | 1,46E-31 |
| WNT7B | NM_058238 | 1,81 | 1,21E-03 |
| CXCR4 | NM_003467 | 1,88 | 2,98E-05 |
| PRKAG2 | NM_024429 | 1,88 | 2,83E-33 |
| NTF3 | NM_002527 | 1,90 | 1,46E-08 |
| TUBA1A | NM_006009 | 1,93 | 3,38E-44 |
| TUBB | NM_178014 | 1,99 | 3,14E-35 |
| OPN1SW | NM_001708 | 2,03 | 1,28E-04 |
| SHANK2 | NM_133266 | 2,21 | 3,74E-05 |
| PLXNA2 | NM_025179 | 2,31 | 2,97E-45 |
| NGF | NM_002506 | 2,32 | 4,70E-10 |
| VEGFC | NM_005429 | 2,44 | 1,23E-56 |
| TUBB2B | NM_178012 | 2,45 | 4,34E-44 |
| KALRN | NM_007064 | 2,47 | 5,00E-34 |
| BDNF | NM_170733 | 2,51 | 1,95E-27 |
| ENPEP | NM_001977 | 2,58 | 5,05E-07 |
| TUBA1C | NM_032704 | 2,64 | 8,52E-57 |
| TUBB2A | NM_001069 | 2,65 | 1,15E-49 |
| MET | NM_001127500 | 2,70 | 5,17E-37 |
| TUBA1B | NM_006082 | 2,72 | 7,51E-119 |
| TUBB4B | NM_006088 | 2,75 | 7,49E-177 |
| ADAMTS1 | NM_006988 | 3,08 | 4,71E-102 |
| TUBB6 | NM_032525 | 3,12 | 2,20E-144 |
| PDGFB | NM_002608 | 3,30 | 1,19E-21 |
| ARHGEF15 | NM_173728 | 3,94 | 3,84E-17 |
| HHIP | NM_022475 | 5,02 | 2,06E-262 |

**Supplemental Table 7:** Differential expression of TGFB1-dependent target genes in co-cultured pericytes

| Gene ID | Refseq ID | Log2FC | P Adjusted | TGFB Prediction |
| --- | --- | --- | --- | --- |
| GRIN2A | NM_001134407 | -6,22 | 3,58E-45 | Activated |
| HGF | NM_000601 | -4,92 | 4,67E-100 | Activated |
| KLRK1 | NM_007360 | -4,38 | 8,20E-10 | Activated |
| BDKRB2 | NM_000623 | -3,92 | 8,45E-10 | Activated |
| DBP | NM_001352 | -3,78 | 8,52E-15 | Activated |
| PTGDS | NM_000954 | -3,70 | 3,58E-27 | Activated |
| SLC1A2 | NM_004171 | -3,63 | 4,61E-07 | Activated |
| TGFB3 | NM_003239 | -3,58 | 7,83E-61 | Activated |
| IGFBP5 | NM_000599 | -3,38 | 2,33E-68 | Activated |
| IFIH1 | NM_022168 | -3,30 | 2,02E-12 | Activated |
| GFAP | NM_001131019 | -3,29 | 1,03E-04 | Activated |
| FNDC5 | NM_153756 | -3,19 | 1,65E-11 | Activated |
| VCAM1 | NM_001078 | -3,03 | 2,99E-08 | Activated |
| PSD2 | NM_032289 | -2,85 | 5,74E-05 | Activated |
| SELENBP1 | NM_003944 | -2,78 | 3,59E-32 | Activated |
| CLCA2 | NM_006536 | -2,72 | 2,00E-03 | Activated |
| CPT1B | NM_001145135 | -2,58 | 8,49E-09 | Activated |
| AMY2B | NM_020978 | -2,58 | 1,02E-09 | Activated |
| TG | NM_003235 | -2,51 | 6,06E-04 | Activated |
| SLC22A18 | NM_183233 | -2,51 | 2,15E-35 | Activated |
| S1PR2 | NM_004230 | -2,42 | 6,43E-47 | Activated |
| LGALS3 | NR_003225 | -2,39 | 5,45E-52 | Activated |
| DCN | NM_133504 | -2,36 | 4,81E-88 | Activated |
| TGFBR3 | NM_003243 | -2,31 | 1,83E-39 | Activated |
| SALL2 | NM_005407 | -2,24 | 3,64E-49 | Activated |
| ANGPT1 | NM_001146 | -2,14 | 1,32E-11 | Activated |
| PPARGC1A | NM_013261 | -2,12 | 1,72E-02 | Activated |
| GPT | NM_005309 | -2,03 | 3,27E-05 | Activated |
| PAX8 | NM_003466 | -1,96 | 3,08E-04 | Activated |
| MPZ | NM_000530 | -1,94 | 1,32E-05 | Activated |
| ACSS1 | NM_032501 | -1,93 | 3,75E-08 | Activated |
| C1R | NM_001733 | -1,87 | 4,22E-15 | Activated |
| NPTXR | NM_014293 | -1,86 | 1,08E-23 | Activated |
| H6PD | NM_004285 | -1,82 | 7,58E-32 | Activated |
| MGAT3 | NM_002409 | -1,81 | 5,02E-05 | Activated |
| GRHL1 | NM_198182 | -1,74 | 3,40E-02 | Activated |
| SERPINF1 | NM_002615 | -1,72 | 1,43E-05 | Activated |
| ITIH3 | NM_002217 | -1,66 | 1,47E-03 | Activated |
| SLC26A11 | NM_173626 | -1,60 | 6,20E-14 | Activated |
| IRF1 | NM_002198 | -1,59 | 1,13E-17 | Activated |
| DISP2 | NM_033510 | -1,58 | 3,49E-05 | Activated |
| CRYGS | NM_017541 | -1,57 | 2,79E-02 | Activated |
| CYP21A2 | NM_000500 | -1,52 | 1,21E-02 | Activated |
| EDNRA | NR_028596 | -1,51 | 2,18E-23 | Activated |
| GNG7 | NM_052847 | -1,43 | 9,50E-03 | Activated |
| PPFIBP2 | NM_003621 | -1,40 | 2,32E-15 | Activated |
| PTGES | NM_004878 | -1,39 | 2,66E-02 | Activated |
| ALDH2 | NM_000690 | -1,37 | 1,66E-15 | Activated |
| ROBO3 | NM_022370 | -1,37 | 6,22E-14 | Activated |
| FTH1 | NM_002032 | -1,37 | 1,69E-21 | Activated |
| CAT | NM_001752 | -1,30 | 2,92E-15 | Activated |
| XPC | NM_004628 | -1,28 | 8,77E-16 | Activated |
| FTL | NM_000146 | -1,27 | 5,97E-31 | Activated |
| CD4 | NM_000616 | -1,23 | 6,07E-07 | Activated |
| GSN | NM_000177 | -1,19 | 6,75E-29 | Activated |
| CELSR2 | NM_001408 | -1,18 | 2,66E-10 | Activated |
| DNMT3A | NM_175630 | -1,18 | 7,06E-03 | Activated |
| HEXA | NM_000520 | -1,17 | 3,19E-22 | Activated |
| ITPR1 | NM_002222 | -1,14 | 2,24E-06 | Activated |
| IFI16 | NM_005531 | -1,08 | 2,53E-16 | Activated |
| ATM | NM_138292 | -1,07 | 1,55E-09 | Activated |
| SGSH | NM_000199 | -1,04 | 8,25E-12 | Activated |
| PTEN | NM_000314 | -0,94 | 7,19E-17 | Activated |
| LPAR1 | NM_001401 | -0,94 | 4,20E-07 | Activated |
| TRAF1 | NM_005658 | -0,91 | 1,64E-02 | Activated |
| ANPEP | NM_001150 | -0,91 | 4,08E-13 | Activated |
| STAT5A | NM_003152 | -0,84 | 3,25E-02 | Activated |
| IGF2 | NM_000612 | -0,81 | 2,15E-03 | Activated |
| MITF | NM_198158 | -0,80 | 1,57E-02 | Activated |
| TRPC1 | NM_003304 | -0,79 | 9,17E-05 | Activated |
| LIPT1 | NM_145197 | -0,77 | 4,90E-02 | Activated |
| FAS | NR_028034 | -0,77 | 8,03E-03 | Activated |
| CDKN1B | NM_004064 | -0,77 | 1,01E-08 | Activated |
| NIT1 | NM_005600 | -0,73 | 1,86E-04 | Activated |
| GCLC | NM_001498 | -0,68 | 9,19E-04 | Activated |
| RIN1 | NM_004292 | -0,67 | 1,32E-07 | Activated |
| GNB5 | NM_016194 | -0,66 | 4,69E-04 | Activated |
| KMT2E | NM_182931 | -0,64 | 1,25E-02 | Activated |
| THBD | NM_000361 | -0,61 | 3,60E-04 | Activated |
| FOS | NM_005252 | 0,61 | 7,93E-03 | Activated |
| RALB | NM_002881 | 0,61 | 8,75E-07 | Activated |
| NET1 | NM_001047160 | 0,61 | 8,78E-05 | Activated |
| PCBP1 | NM_006196 | 0,61 | 5,34E-05 | Activated |
| RPN2 | NM_001135771 | 0,61 | 3,48E-06 | Activated |
| EXT1 | NM_000127 | 0,62 | 1,36E-07 | Activated |
| ZEB2 | NM_014795 | 0,63 | 1,07E-04 | Activated |
| EIF4H | NM_022170 | 0,63 | 5,23E-11 | Activated |
| CYC1 | NM_001916 | 0,64 | 3,95E-05 | Activated |
| SMURF1 | NM_020429 | 0,64 | 9,06E-05 | Activated |
| RAB1A | NM_004161 | 0,64 | 7,64E-09 | Activated |
| HMGA2 | NM_003484 | 0,64 | 1,11E-06 | Activated |
| ADAM19 | NM_033274 | 0,64 | 1,07E-07 | Activated |
| HES1 | NM_005524 | 0,65 | 8,55E-05 | Activated |
| RHOA | NM_001664 | 0,66 | 8,97E-11 | Activated |
| HDGF | NM_001126050 | 0,66 | 1,62E-05 | Activated |
| S100A11 | NM_005620 | 0,66 | 1,11E-04 | Activated |
| NT5E | NM_002526 | 0,66 | 5,40E-07 | Activated |
| IARS | NM_013417 | 0,67 | 4,35E-12 | Activated |
| FAM3C | NM_014888 | 0,67 | 4,94E-07 | Activated |
| IL4R | NM_000418 | 0,68 | 1,65E-08 | Activated |
| ARF4 | NM_001660 | 0,68 | 4,61E-08 | Activated |
| PLOD1 | NM_000302 | 0,69 | 4,66E-12 | Activated |
| ASNS | NM_001673 | 0,71 | 2,09E-08 | Activated |
| PPP2R2A | NM_002717 | 0,71 | 4,61E-06 | Activated |
| LSM5 | NM_001130710 | 0,71 | 3,49E-03 | Activated |
| SDC1 | NM_002997 | 0,72 | 9,58E-07 | Activated |
| DNAJB6 | NM_005494 | 0,72 | 1,04E-06 | Activated |
| PTGS2 | NM_000963 | 0,73 | 1,57E-03 | Activated |
| SERPINH1 | NM_001235 | 0,73 | 1,24E-08 | Activated |
| JAG1 | NM_000214 | 0,74 | 3,43E-13 | Activated |
| GARS | NM_002047 | 0,74 | 9,46E-11 | Activated |
| ST3GAL4 | NM_006278 | 0,75 | 9,44E-04 | Activated |
| SLC52A2 | NM_024531 | 0,75 | 4,80E-07 | Activated |
| MPP6 | NM_016447 | 0,75 | 1,66E-02 | Activated |
| NMNAT2 | NM_170706 | 0,75 | 1,13E-03 | Activated |
| MAP4 | NM_030885 | 0,76 | 7,40E-06 | Activated |
| SLC4A2 | NM_003040 | 0,77 | 1,23E-10 | Activated |
| SRR | NM_021947 | 0,77 | 4,13E-07 | Activated |
| SLC2A1 | NM_006516 | 0,78 | 1,23E-05 | Activated |
| SPHK1 | NM_001142601 | 0,80 | 1,10E-03 | Activated |
| S100A10 | NM_002966 | 0,80 | 6,48E-09 | Activated |
| NDST1 | NM_001543 | 0,81 | 5,44E-11 | Activated |
| RUNX2 | NM_001015051 | 0,81 | 8,28E-04 | Activated |
| ABCF1 | NM_001025091 | 0,82 | 3,60E-13 | Activated |
| AKT1 | NM_001014431 | 0,83 | 3,02E-16 | Activated |
| ILK | NM_001014795 | 0,83 | 5,21E-11 | Activated |
| LASP1 | NM_006148 | 0,84 | 1,64E-13 | Activated |
| ZYX | NM_001010972 | 0,84 | 3,90E-13 | Activated |
| PI4K2B | NM_018323 | 0,85 | 6,81E-07 | Activated |
| COL8A1 | NM_001850 | 0,85 | 7,40E-19 | Activated |
| TAX1BP3 | NM_014604 | 0,85 | 4,54E-08 | Activated |
| KPNA3 | NM_002267 | 0,87 | 3,86E-09 | Activated |
| PLS3 | NM_005032 | 0,88 | 1,95E-14 | Activated |
| ADK | NM_006721 | 0,88 | 2,58E-08 | Activated |
| PSMC1 | NM_002802 | 0,88 | 1,06E-11 | Activated |
| PDGFC | NM_016205 | 0,89 | 3,22E-07 | Activated |
| FLNA | NM_001110556 | 0,89 | 2,83E-12 | Activated |
| SEMA7A | NM_001146030 | 0,90 | 1,99E-07 | Activated |
| TIMP3 | NM_000362 | 0,90 | 8,82E-11 | Activated |
| ITGBL1 | NM_004791 | 0,91 | 2,66E-07 | Activated |
| HSPB1 | NM_001540 | 0,91 | 5,22E-09 | Activated |
| CAV1 | NM_001753 | 0,92 | 1,06E-12 | Activated |
| ADAMTS2 | NM_014244 | 0,93 | 1,66E-11 | Activated |
| MYO1C | NM_001080950 | 0,94 | 1,44E-18 | Activated |
| TGFBR1 | NM_001130916 | 0,94 | 4,32E-11 | Activated |
| PSMD1 | NM_002807 | 0,95 | 5,11E-17 | Activated |
| VCL | NM_014000 | 0,96 | 4,67E-14 | Activated |
| CNN2 | NM_004368 | 0,98 | 1,44E-10 | Activated |
| MYL6 | NM_079423 | 0,98 | 7,43E-06 | Activated |
| HSPB2 | NM_001541 | 0,99 | 1,26E-04 | Activated |
| ENPP1 | NM_006208 | 1,00 | 9,67E-09 | Activated |
| PPP2CA | NM_002715 | 1,00 | 7,75E-23 | Activated |
| GDF6 | NM_001001557 | 1,02 | 3,57E-06 | Activated |
| ITGA3 | NM_002204 | 1,03 | 8,84E-20 | Activated |
| FILIP1L | NM_001042459 | 1,04 | 8,41E-06 | Activated |
| SSRP1 | NM_003146 | 1,04 | 6,12E-13 | Activated |
| P4HA1 | NM_000917 | 1,04 | 7,27E-19 | Activated |
| XBP1 | NM_005080 | 1,04 | 7,31E-24 | Activated |
| MAD1L1 | NM_001013837 | 1,04 | 1,24E-05 | Activated |
| HIF1A | NM_001530 | 1,05 | 1,32E-12 | Activated |
| MSN | NM_002444 | 1,06 | 6,57E-15 | Activated |
| ME2 | NM_002396 | 1,06 | 1,41E-11 | Activated |
| FSCN1 | NM_003088 | 1,06 | 3,22E-26 | Activated |
| RHOC | NM_001042678 | 1,07 | 7,40E-25 | Activated |
| CHST11 | NM_018413 | 1,09 | 5,11E-07 | Activated |
| IER3 | NM_003897 | 1,09 | 2,63E-11 | Activated |
| C20orf24 | NM_199483 | 1,11 | 4,42E-11 | Activated |
| ELAVL1 | NM_001419 | 1,11 | 2,49E-18 | Activated |
| VCAN | NM_001126336 | 1,12 | 2,25E-19 | Activated |
| CAV2 | NM_001233 | 1,12 | 7,34E-20 | Activated |
| OSTF1 | NM_012383 | 1,13 | 1,05E-08 | Activated |
| PLEK2 | NM_016445 | 1,13 | 2,35E-02 | Activated |
| ARPC2 | NM_005731 | 1,14 | 1,34E-24 | Activated |
| GNB4 | NM_021629 | 1,15 | 4,13E-26 | Activated |
| ADAMTS4 | NM_005099 | 1,18 | 9,47E-03 | Activated |
| ITGA5 | NM_002205 | 1,21 | 1,84E-28 | Activated |
| CHST3 | NM_004273 | 1,22 | 2,02E-25 | Activated |
| FGF2 | NM_002006 | 1,23 | 1,11E-11 | Activated |
| CD59 | NM_001127223 | 1,23 | 7,37E-37 | Activated |
| PSMC3 | NM_002804 | 1,26 | 3,28E-17 | Activated |
| BMP2 | NM_001200 | 1,26 | 2,56E-04 | Activated |
| MYL12A | NM_006471 | 1,27 | 5,24E-14 | Activated |
| TAGLN | NM_001001522 | 1,27 | 8,25E-12 | Activated |
| CFL1 | NM_005507 | 1,28 | 1,18E-25 | Activated |
| THY1 | NM_006288 | 1,30 | 9,81E-08 | Activated |
| CNN3 | NM_001839 | 1,32 | 5,36E-39 | Activated |
| PIM1 | NM_002648 | 1,32 | 1,55E-14 | Activated |
| LAMC2 | NM_005562 | 1,34 | 2,55E-03 | Activated |
| NOP58 | NM_015934 | 1,34 | 1,53E-21 | Activated |
| CALM1 | NM_001166106 | 1,34 | 2,20E-35 | Activated |
| HNRNPH1 | NM_005520 | 1,35 | 1,18E-27 | Activated |
| GLI2 | NM_005270 | 1,37 | 1,01E-12 | Activated |
| KRT18 | NM_199187 | 1,38 | 1,59E-15 | Activated |
| FERMT2 | NM_001135000 | 1,41 | 4,36E-42 | Activated |
| MAP2K1 | NM_002755 | 1,42 | 8,80E-26 | Activated |
| BCL2 | NM_000633 | 1,43 | 3,63E-05 | Activated |
| GJA1 | NM_000165 | 1,44 | 7,84E-42 | Activated |
| RSU1 | NM_012425 | 1,45 | 5,66E-13 | Activated |
| GADD45B | NM_015675 | 1,45 | 6,12E-21 | Activated |
| ID1 | NM_002165 | 1,45 | 1,83E-18 | Activated |
| MYOCD | NM_001146312 | 1,46 | 5,02E-16 | Activated |
| TUBB3 | NM_006086 | 1,46 | 2,24E-40 | Activated |
| CSRP2 | NM_001321 | 1,46 | 3,95E-08 | Activated |
| VASP | NM_003370 | 1,48 | 1,46E-18 | Activated |
| CDC42EP3 | NM_006449 | 1,49 | 2,30E-21 | Activated |
| NR4A1 | NM_002135 | 1,51 | 3,99E-08 | Activated |
| RASGRP1 | NM_005739 | 1,51 | 3,27E-02 | Activated |
| CAP1 | NM_001105530 | 1,53 | 1,84E-37 | Activated |
| HSPA5 | NM_005347 | 1,53 | 1,04E-34 | Activated |
| AXL | NM_001699 | 1,54 | 1,27E-34 | Activated |
| SRF | NM_003131 | 1,55 | 1,13E-30 | Activated |
| RUNX3 | NM_004350 | 1,56 | 4,85E-04 | Activated |
| BMP6 | NM_001718 | 1,56 | 8,95E-09 | Activated |
| RNF152 | NM_173557 | 1,59 | 8,39E-11 | Activated |
| NOS3 | NM_000603 | 1,61 | 1,84E-02 | Activated |
| CDK5R1 | NM_003885 | 1,63 | 5,24E-08 | Activated |
| TNC | NM_002160 | 1,64 | 1,66E-37 | Activated |
| IL11 | NM_000641 | 1,66 | 2,55E-26 | Activated |
| ACLY | NM_001096 | 1,67 | 2,25E-52 | Activated |
| FABP5 | NM_001444 | 1,70 | 5,68E-04 | Activated |
| SRM | NM_003132 | 1,71 | 3,90E-45 | Activated |
| FLNB | NM_001164317 | 1,72 | 2,97E-55 | Activated |
| WNT5B | NM_032642 | 1,76 | 1,36E-21 | Activated |
| DUSP1 | NM_004417 | 1,78 | 1,92E-53 | Activated |
| TPM1 | NM_000366 | 1,78 | 1,25E-27 | Activated |
| SLC7A1 | NM_003045 | 1,79 | 1,06E-28 | Activated |
| ACTN1 | NM_001102 | 1,79 | 1,41E-51 | Activated |
| SRSF3 | NM_003017 | 1,80 | 4,13E-36 | Activated |
| CCL2 | NM_002982 | 1,83 | 7,83E-08 | Activated |
| RHOB | NM_004040 | 1,84 | 5,55E-40 | Activated |
| GCNT1 | NM_001097633 | 1,86 | 6,44E-10 | Activated |
| CORO1C | NM_014325 | 1,86 | 1,99E-93 | Activated |
| ETS1 | NM_001143820 | 1,86 | 2,48E-39 | Activated |
| HAPLN3 | NM_178232 | 1,87 | 1,13E-29 | Activated |
| RASGRF2 | NM_006909 | 1,88 | 3,53E-02 | Activated |
| CXCR4 | NM_003467 | 1,88 | 2,98E-05 | Activated |
| RASGRP3 | NM_170672 | 1,89 | 8,44E-04 | Activated |
| HSP90AA1 | NM_005348 | 1,90 | 6,74E-51 | Activated |
| HAS2 | NM_005328 | 1,92 | 6,57E-26 | Activated |
| TUBA1A | NM_006009 | 1,93 | 3,38E-44 | Activated |
| PRPS1 | NM_002764 | 1,95 | 3,59E-27 | Activated |
| LDHA | NM_001165415 | 1,96 | 1,85E-43 | Activated |
| CCT5 | NM_012073 | 1,97 | 3,30E-59 | Activated |
| PLOD2 | NM_182943 | 1,98 | 1,37E-74 | Activated |
| GREM1 | NM_013372 | 1,98 | 1,23E-28 | Activated |
| CSPG4 | NM_001897 | 1,98 | 3,23E-84 | Activated |
| PLAUR | NM_001005376 | 1,99 | 4,42E-40 | Activated |
| TPM3 | NM_152263 | 2,02 | 1,76E-24 | Activated |
| HNRNPAB | NM_004499 | 2,03 | 3,26E-57 | Activated |
| CDC25C | NM_001790 | 2,03 | 6,07E-16 | Activated |
| THBS1 | NM_003246 | 2,05 | 1,14E-30 | Activated |
| PCOLCE2 | NM_013363 | 2,06 | 1,29E-15 | Activated |
| CSRP1 | NM_001144773 | 2,06 | 3,73E-33 | Activated |
| PODXL | NM_001018111 | 2,09 | 1,53E-30 | Activated |
| SMURF2 | NM_022739 | 2,11 | 4,46E-44 | Activated |
| ABCG1 | NM_207629 | 2,13 | 3,01E-03 | Activated |
| CDK1 | NM_033379 | 2,16 | 1,02E-43 | Activated |
| CNN1 | NM_001299 | 2,17 | 2,25E-23 | Activated |
| TNFRSF12A | NM_016639 | 2,18 | 5,35E-41 | Activated |
| CDH2 | NM_001792 | 2,19 | 8,60E-55 | Activated |
| NME1 | NM_198175 | 2,21 | 3,94E-43 | Activated |
| RFLNB | NM_182705 | 2,23 | 1,26E-49 | Activated |
| CCNE2 | NM_057749 | 2,29 | 7,65E-15 | Activated |
| FGF5 | NM_004464 | 2,31 | 7,08E-55 | Activated |
| NGF | NM_002506 | 2,32 | 4,70E-10 | Activated |
| IDI1 | NM_004508 | 2,32 | 3,61E-40 | Activated |
| FSTL3 | NM_005860 | 2,39 | 2,09E-59 | Activated |
| LOX | NM_002317 | 2,43 | 9,20E-35 | Activated |
| VEGFC | NM_005429 | 2,44 | 1,23E-56 | Activated |
| TNFAIP3 | NM_006290 | 2,45 | 1,12E-42 | Activated |
| TGM2 | NM_004613 | 2,48 | 1,77E-34 | Activated |
| BDNF | NM_170733 | 2,51 | 1,95E-27 | Activated |
| ACTA2 | NM_001613 | 2,51 | 1,46E-47 | Activated |
| ACTC1 | NM_005159 | 2,52 | 1,95E-03 | Activated |
| CCND1 | NM_053056 | 2,53 | 4,67E-123 | Activated |
| PTHLH | NM_002820 | 2,58 | 2,08E-06 | Activated |
| ACTG2 | NM_001615 | 2,63 | 5,87E-62 | Activated |
| TUBB2A | NM_001069 | 2,65 | 1,15E-49 | Activated |
| SMTN | NM_134270 | 2,67 | 7,70E-98 | Activated |
| HDAC9 | NM_014707 | 2,69 | 3,05E-14 | Activated |
| MET | NM_001127500 | 2,70 | 5,17E-37 | Activated |
| CTPS1 | NM_001905 | 2,72 | 1,89E-75 | Activated |
| IFI27 | NM_005532 | 2,72 | 8,39E-12 | Activated |
| LYVE1 | NM_006691 | 2,74 | 2,37E-28 | Activated |
| ORC1 | NM_004153 | 2,75 | 1,31E-27 | Activated |
| SOCS1 | NM_003745 | 2,76 | 1,28E-14 | Activated |
| CCNB1 | NM_031966 | 2,80 | 1,92E-78 | Activated |
| MYBL2 | NM_002466 | 2,83 | 2,40E-99 | Activated |
| NNMT | NM_006169 | 2,84 | 3,53E-11 | Activated |
| KRT81 | NM_002281 | 2,85 | 5,34E-04 | Activated |
| E2F1 | NM_005225 | 2,93 | 3,15E-60 | Activated |
| KIT | NM_000222 | 3,03 | 4,87E-39 | Activated |
| ESAM | NM_138961 | 3,04 | 2,40E-21 | Activated |
| SLC7A5 | NM_003486 | 3,11 | 5,26E-143 | Activated |
| CFI | NM_000204 | 3,17 | 6,22E-06 | Activated |
| PDGFB | NM_002608 | 3,30 | 1,19E-21 | Activated |
| IL32 | NM_001012635 | 3,32 | 1,37E-18 | Activated |
| ACVRL1 | NM_001077401 | 3,32 | 1,63E-28 | Activated |
| ITGA6 | NM_001079818 | 3,35 | 4,86E-81 | Activated |
| INHBA | NM_002192 | 3,37 | 1,41E-158 | Activated |
| ELMO1 | NM_014800 | 3,41 | 6,68E-07 | Activated |
| RGCC | NM_014059 | 3,54 | 8,90E-07 | Activated |
| VWF | NM_000552 | 3,62 | 5,88E-56 | Activated |
| NEDD9 | NM_006403 | 3,69 | 3,28E-57 | Activated |
| ICAM2 | NM_000873 | 3,83 | 4,74E-23 | Activated |
| ACAN | NM_001135 | 3,89 | 3,43E-70 | Activated |
| KRT7 | NM_005556 | 3,98 | 2,17E-92 | Activated |
| HBEGF | NM_001945 | 4,04 | 5,98E-66 | Activated |
| EDN1 | NM_001955 | 4,06 | 1,03E-13 | Activated |
| IL6 | NM_000600 | 4,19 | 3,54E-21 | Activated |
| CTGF | NM_001901 | 4,40 | 9,96E-203 | Activated |
| CYR61 | NM_001554 | 4,68 | 9,92E-222 | Activated |
| FLT1 | NM_001159920 | 4,69 | 6,34E-35 | Activated |
| SERPINE1 | NM_000602 | 4,86 | 0,00E+00 | Activated |
| ANKRD1 | NM_014391 | 4,90 | 2,02E-112 | Activated |
